# Trajectories tracking of maternal and neonatal health in eastern China from 2010 to 2021: A multicentre cross-sectional study

**DOI:** 10.7189/jogh.14.04069

**Published:** 2024-03-22

**Authors:** Hui Zhu, Jie Cai, Hongyi Liu, Zhijia Zhao, Yanming Chen, Penghao Wang, Tao Chen, Da He, Xiang Chen, Jin Xu, Lindan Ji

**Affiliations:** 1 Department of Internal Medicine, Health Science Center, Ningbo University, Ningbo city, Zhejiang province, China; 2 Center for Reproductive Medicine, Ningbo Women and Children’s Hospital, Ningbo city, Zhejiang province, China; 3 School of Public Health, Health Science Center, Ningbo University, Ningbo city, Zhejiang province, China; 4 Department of Medical Records and Statistics, Beilun People's Hospital, Ningbo city, Zhejiang province, China; 5 Department of Obstetrics and Gynecology, Yinzhou District Maternal and Child Health Care Institute, Ningbo city, Zhejiang province, China; 6 Zhejiang Key Laboratory of Pathophysiology, Health Science Center, Ningbo University, Ningbo city, Zhejiang province, China; 7 Department of Biochemistry and Molecular Biology, School of Basic Medical Sciences, Health Science Center, Ningbo University, Ningbo city, Zhejiang province, China

## Abstract

**Background:**

China’s fertility policy has dramatically changed in the past decade with the successive promulgation of the partial two-child policy, universal two-child policy and three-child policy. The trajectories of maternal and neonatal health accompanied the changes in fertility policy are unknown.

**Methods:**

We obtained data of 280 203 deliveries with six common pregnancy complications and thirteen perinatal outcomes between 2010 and 2021 in eastern China. The average annual percent change (AAPC) was calculated to evaluated the temporal trajectories of obstetric characteristics and adverse outcomes during this period. Then, the autoregressive integrated moving average (ARIMA) models were constructed to project future trend of obstetric characteristics and outcomes until 2027.

**Results:**

The proportion of advanced maternal age (AMA), assisted reproduction technology (ART) treatment, gestational diabetes mellitus (GDM), anaemia, thrombocytopenia, thyroid dysfunction, oligohydramnios, placental abruption, small for gestational age (SGA) infants, and congenital malformation significantly increased from 2010 to 2021. However, the placenta previa, large for gestational age (LGA) infants and stillbirth significantly decreased during the same period. The AMA and ART treatment were identified as independent risk factors for the uptrends of pregnancy complications and adverse perinatal outcomes. The overall caesarean section rate remained above 40%. Importantly, among multiparas, a previous caesarean section was found to be associated with a significantly reduced risk of hypertensive disorders of pregnancy (HDP), premature rupture of membranes (PROM), placenta previa, placental abruption, perinatal asphyxia, LGA infants, stillbirths, and preterm births. In addition, the ARIMA time series models predicted increasing trends in the ART treatment, GDM, anaemia, thrombocytopenia, postpartum haemorrhage, congenital malformation, and caesarean section until 2027. Conversely, a decreasing trend was predicted for HDP, PROM, and placental abruption premature, LGA infants, SGA infants, perinatal asphyxia, and stillbirth.

**Conclusions:**

Maternal and neonatal adverse outcomes became more prevalent from 2010 to 2021 in China. Maternal age and ART treatment were independent risk factors for adverse obstetric outcomes. The findings offered comprehensive trajectories for monitoring pregnancy complications and perinatal outcomes in China, and provided robust intervention targets in obstetric safety. The development of early prediction models and the implementation of prevention efforts for adverse obstetric events are necessary to enhance obstetric safety.

The rapidly declining fertility rate has been an urgent issue of national concern in China. In this context, China’s fertility policy has dramatically changed in the past decade with the successive promulgation of the partial two-child policy (November 2013), universal two-child policy (October 2015) and three-child policy (May 2021) [1,2]. Under the impetus of the universal two-child policy, there was an increase in the number of births in China during 2016 and 2017 [3,4]. However, this trend appears to have been short-lived. According to China's Seventh National Census (2020), the total fertility rate (TFR) has fallen to 1.3 children per woman of childbearing age (15–49 years), which is even lower than the global warning line of 1.5 [5]. Moreover, the birth rate in 2022 declined to 6.77‰, and the natural growth rate of the population was −0.6 ‰, which was the first negative growth rate since 1962 [6]. Chinese women of reproductive age are facing a fertility paradox: while the state encourages childbirth, various factors have led to declined fertility desires and delayed childbearing. Currently, obstetric health and safety is not only a medical concern but also an important social issue in China.

Pregnancy induces intense physiological changes in all maternal organs, particularly profound alterations in the hormonal milieu, systemic metabolism, hemodynamic parameters, the haemostatic system, the cardiovascular system and renal function, to meet the robust energy demands of the developing foetus [7–9]. Several common pregnancy complications, such as gestational diabetes mellitus (GDM), gestational hypertension (GH) and preeclampsia (PE), arise from the system-wide metabolic reprogramming that exceeds the maternal compensatory capacity due to insulin resistance, elevated lipid profiles or placental hormones and cytokines, leading to the deterioration of β-cell secretion function and endothelial damage [10]. In recent decades, there has been an increasing prevalence of pregnancy complications, and their associated adverse maternal and neonatal outcomes have gained recognition. According to the International Diabetes Federation (IDF) 2021 report, the global prevalence of GDM is estimated to be 13.41%, affecting approximately 17 million pregnant women aged 20–49 years and exhibiting a rapid global increase of over 30% during the past decade [10]. Hypertensive disorders of pregnancy (HDPs) include chronic hypertension, gestational hypertension, preeclampsia and eclampsia. The prevalence of HDPs is approximately 116.4 per 100 000 women of childbearing age by WHO region [10], but it remains one of the most common obstetric causes of maternal and infant mortality [11]. These two complications result in a range of short- and long-term adverse consequences for mothers and their offspring [12,13]. Pregnant women with complications of GDM and/or HDP experience greater rates of abortion, amniotic fluid volume abnormalities, placental diseases, caesarean section and postpartum haemorrhage in the perinatal stage, and their offspring experience greater rates of foetal malformations, intrauterine growth restriction (IUGR), neonatal hypoglycaemia, macrosomia, low birthweight, and premature delivery [12,14–17]. Furthermore, women with a history of GDM are at a 7-fold increased risk for developing type 2 diabetes mellitus (T2DM), and their offspring face an 8-fold increased risk of diabetes [18]. HDPs are also responsible for increased risks of cardiovascular, cerebrovascular and metabolic diseases in mothers and their offspring, as well as reduced life expectancy [13,19,20]. Simultaneously, pregnant women with HDPs have increased risks of IUGR, bleeding events, admission to the intensive care unit (ICU) and even maternal or foetal mortality due to several haematological issues that commonly arise during pregnancy, including anaemia, thrombocytopenia and coagulation abnormalities [21–23]. Specifically, anaemia is the most widespread condition, with rates reaching as high as 40%, and the rate of thrombocytopenia during pregnancy ranges from 5–10% [21,22]. These pregnancy complications may manifest as comorbidities or exhibit intricate interactions, posing substantial challenges to obstetric health and imposing a substantial disease burden despite the provision of advanced medical care and interventions.

Furthermore, advanced maternal age (AMA) and assisted reproductive technology (ART) interventions have become increasingly prevalent in past decades, leading to medical and social challenges that pose a higher risk for both maternal and perinatal complications. AMA generally refers to childbearing in women who are 35 years of age or older. The prevalence of AMA is on the rise worldwide, with rates reaching 20% in the United States and 33.4% in Korea [24,25]. In China, the reported proportion of women with AMA ranges from 8 to 15% of all births, especially that of first births among women of AMA, which are progressively increasing [6,26,27]. Age-related infertility often coexists with delayed childbearing, leading to increased demands for ART treatment among women of reproductive age [28]. It is estimated that there are over 10 million children conceived through ART worldwide [29]. Moreover, over 300 000 babies conceived through ART are born annually in China, accounting for 2.7% of the total births [23]. It appears that obstetric and perinatal complications are significantly higher in women who undergo ART treatments [30,31]. However, the effects of ART treatment, AMA and their interactions on adverse pregnancy complications and outcomes are not adequately understood, which might further complicate the obstetric challenges.

We conducted a retrospective, multicentre cross-sectional study of 280 203 women in Zhejiang, China, based on the surveillance system of deliveries from 2010–2021. We aimed to investigate the temporal trajectories of obstetric characteristics and adverse outcomes over the past 12 years in eastern China. Furthermore, we evaluated the independent and joint risk effects of AMA and ART treatment on major pregnancy complications and adverse maternal as well as foetal outcomes. Our ultimate objectives were to offer appropriate guidance for women’s reproductive decisions while promoting maternal and infant health.

## METHODS

### Study population and data collection

This multicentre retrospective cross-sectional study was conducted using the electronic medical records of obstetric hospitalisations from four medical centres in Zhejiang Province, China, between January 2010 and December 2021. Zhejiang is a province in eastern China with a resident population of 6540 million in 2021. Pregnancy complications and outcomes were ascertained through manual assessments of obstetrical hospitalisation diagnoses. The data also included information about delivery year, maternal age, gestational age, ART treatment, hepatitis B virus (HBV) infection and past medical history.

**Figure 1:**
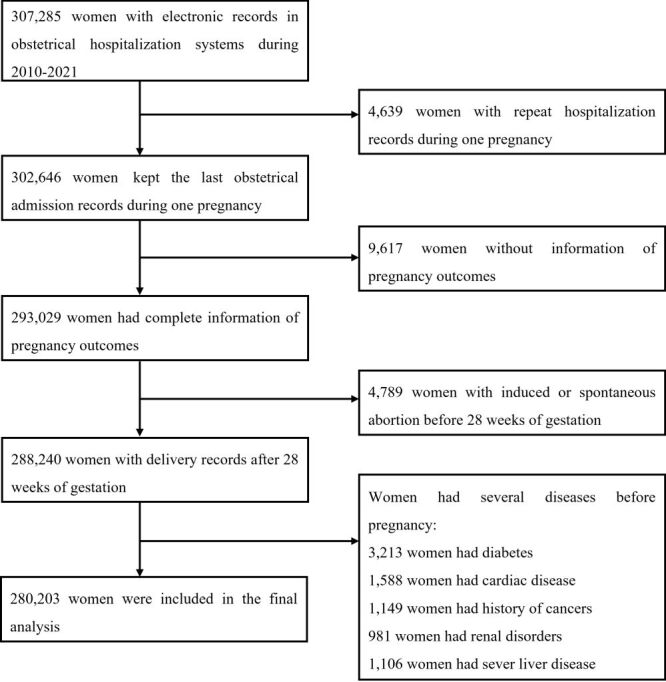
Flowchart for eligible participants of this study.

The inclusion criteria for this study encompassed maternal age ranging from 15 to 49 years, gestational age of at least 28 weeks, availability of diagnostic information for both the mother and newborn, and Chinese Han population. We excluded participants with a previous diagnosis of diabetes, cardiac disease, renal disorders, severe liver disease and cancer before pregnancy. A total of 280 203 eligible participants were included in the final analysis (**Figure 1**).

According to the information security technology guide for health data security of China, the sensitive information fields of all participants (such as name, ID number, medical insurance account, address, telephone number, work unit, etc.) would undergo desensitisation and deformation through predefined rules in order to ensure the protection of sensitive privacy data prior to data analysis [32].

### Criteria for pregnancy complications and adverse perinatal outcomes

Six common pregnancy complications, including GDM, HDPs, hypothyroidism, hyperthyroidism, thrombocytopenia, and anaemia, and thirteen maternal and neonatal outcomes, including polyhydramnios, oligohydramnios, premature rupture of membranes (PROM), placenta previa, placental abruption, caesarean section, perinatal asphyxia, premature birth, small for gestational age (SGA), large for gestational age (LGA), stillbirth, postpartum haemorrhage, and congenital malformations, were included in this analysis. The detailed diagnostic criteria are presented in the Appendix S1 in the **Online Supplementary Document**.

### Ethics approval

This study was approved by the Ningbo University Medical Science Research Ethics Committee.

### Statistical analysis

Descriptive statistics were utilised to present the characteristics of prevalent pregnancy complications and perinatal outcomes from 2010 to 2021. Continuous variables are presented as the means and standard deviations (SDs), and categorical variables are presented as the frequency (n) and proportion (%). Poisson regression models were employed to examine the impact of calendar year on obstetric characteristics, with results presented as prevalence rate ratios (PRRs) and 95% confidence intervals (CIs). The calendar year was included as a dummy variable in these models.

The detailed temporal trends with years for AMA, the ART rate, the prevalence of pregnancy complications and adverse perinatal outcomes were determined using Joinpoint regression analysis. The Joinpoint model established all possible connection points (i.e. Joinpoint points) between interval segments using the grid search method (GSM) method, and calculated the corresponding sum of squared errors (SSE) and mean squared errors (MSE) for each potential case [33]. The grid point with the smallest MSE was selected as the connection point for the piecewise function. The permutation test was used to optimise the Joinpoint model and estimated the optimal join point k. In this study, the value for the maximum number of Joinpoints depended on the number of data points, and the temporal trend analysis from 2010–2021 allowed a maximum of two Joinpoints. Finally, the average annual percent change (AAPC), annual percent change (APC), and 95% confidence intervals (CI) were calculated by Joinpoint model.

Moreover, autoregressive integrated moving average (ARIMA) model was also used to analyse and forecast the time series data [34]. The ARIMA model consisted of three components: the autoregression (AR), moving average (MA), and integration (I). AR captured the correlation between a given time period and its previous periods. MA used past forecasting errors to predict future variables. I was utilised to induce the non-stationary time series into a stationary state, thereby eliminating the trend factor in the time series through first or second order differencing.

Based on the yearly data, we constructed sample ARIMA (p, d, q) models for prediction of each obstetric adverse event. The parameter p represented the order of autoregressive that captures the interdependence between current and historical values, the parameter q was the number of moving average terms, and parameter d was the order of difference to form a stationary time series. The process of constructing an ARIMA model typically involved four steps. First, the stationarity of time series data was test using Augmented Dickey-Fuller (ADF) unit-root test, and the corresponding d-difference would be applied to address non-stationarity time series. Second, the plots of Autocorrelation Function (ACF) and Partial Autocorrelation (PACF) were performed to determine the p and q values. Third, the optimal model was selected by the lowest Akaike information criterion (AIC) and Bayesian information criterion (BIC) values, and the model evaluation was conducted using white noise by Ljung-Box test. Finally, we used the established ARIMA model to make predictions.

Advanced maternal age, ART treatment and prior caesarean section were previously reported as independent risk factors for several pregnancy complications and adverse perinatal outcomes. Poisson regression models were also used to examine the association between maternal age, ART treatment or prior caesarean section and pregnancy complications as well as adverse perinatal outcomes. Age was categorised into six groups: <20, 20–24, 25–29, 30–34, 35–39 and ≥40 years in the Poisson models, and 20–24 years was set as the reference group. Additionally, we carried out a series of sensitivity analyses to assess the robustness of the associations of age and ART with the aforementioned adverse obstetric events. First, restricted cubic spline analysis was applied to explore the dose-response relationship between maternal age (15–49 years) and the risk of pregnancy complications as well as adverse perinatal outcomes. Second, the dose relationships between maternal age and adverse obstetric events were examined in women with ART treatment and spontaneous conception (SC). Third, cross stratification analysis was conducted to analyse the associations between ART treatment as well as twin or multiple gestations and adverse obstetric events.

The comorbid pregnancy complications of each participant were calculated and a new variable, ‘comorbid pregnancy complications’, was generated, which was categorised into four groups: no pregnancy complications, one complication, two complications and three or more complications. We ultimately analysed the cumulative effect of multiple pregnancy complications on adverse perinatal outcomes.

These statistical analyses were conducted using R software (version 4.2.0), the Joinpoint Regression Program (version 4.8.0.1) and GraphPad Prism (version 8.0.2). *P* < 0.05 (two-tailed) was considered significant.

## RESULTS

### Obstetric characteristics and prevalence of adverse obstetric events

**Figure 2:**
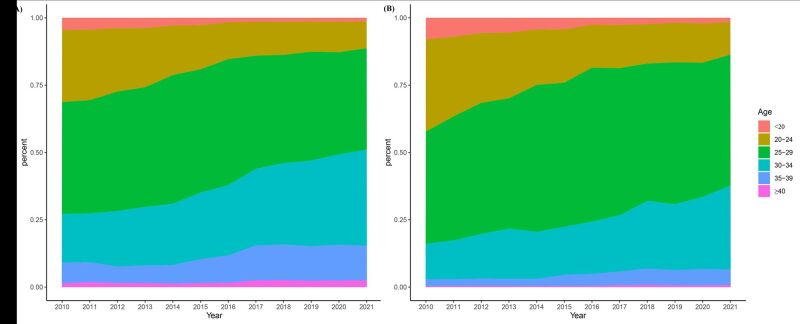
Stacked area graph of the trend in maternal age groups from 2010 − 2021. **Panel A. **The age distribution in the overall maternal population.** Panel B.** The age distribution in the primiparas

A total of 280 203 pregnant women who delivered between 2010 and 2021 were eligible for this study, and the obstetric characteristics and annual prevalence of adverse obstetric events are presented in Table S1 in the **Online Supplementary Document**. The average maternal age at delivery was 28.52 ± 4.86 years, and the proportion of pregnant women aged 30 years or older increased over time (**Figure 2**, panel A). Furthermore, a similar pattern was noted among primiparas (**Figure 2**, panel B). 

Overall, the prevalence rates of GDM, HDP, hypothyroidism, hyperthyroidism, anaemia and thrombocytopenia were 13.2, 6.1, 9.1, 0.4, 21.6, and 2.35%, respectively. Simultaneously, the prevalence of thirteen perinatal adverse outcomes was analysed. In terms of adverse maternal outcomes, the rates of caesarean section and postpartum haemorrhage remained stable at approximately 40 and 8.7%, respectively. Premature birth (10.8%), perinatal asphyxia (5.7%), LGA (5.5%) and SGA (3.7%) were still common adverse infant outcomes. Notably, the ratio of women conceived by ART treatment increased remarkably from 0.78 to 6.09% between 2011 and 2021 (Table S1 in the **Online Supplementary Document**).

### Temporal trajectories of obstetric characteristics and adverse outcomes during 2010–2021

**Figure 3:**
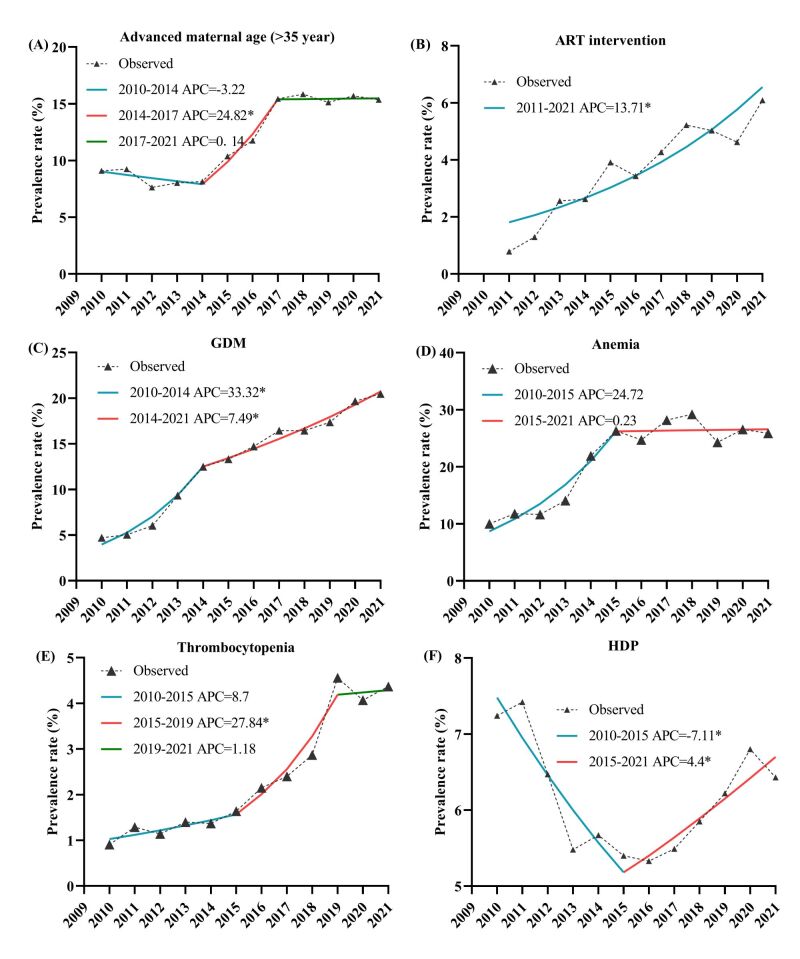
The temporal trends in prevalence of advanced maternal age, ART treatment and several pregnancy complications from 2010 to 2021. **Panel A.** Advanced maternal age. **Panel B.** ART treatment. **Panel C.** GDM. **Panel D.** Anaemia during pregnancy. **Panel E.** Thrombocytopenia during pregnancy. **Panel F.** HDP. The number of Joinpoints were selected by the optimal models from Joinpoint regression analysis. *Indicates that the annual percent change (APC) is significantly different from zero at the α = 0.05 level. ART – assisted reproductive technology, GDM – gestational diabetes mellitus, HDP – hypertensive disorders in pregnancy

All the detailed AAPC and segment annual percent changes (APC) in obstetrics characteristics and adverse events during 2010–2021 were shown in Table S2 in the **Online Supplementary Document**. The Joinpoint regression analysis showed that the rate of AMA (≥35 years) rose significantly by 5.03% (95% CI = 3.93, 6.26%) from 2010–2021 overall. In particular, this trend increased sharply with an annual percent change (APC) of 24.82% (95% CI = 17.86, 29.87%) from 2014–2017 before and after the universal two-child policy, but the trend after 2017 was relatively stable (**Figure 3**, panel A). The rate of women who conceived with ART intervention continuously increased over time, with an AAPC of 13.71% (95% CI = 7.83, 19.91%) per year from 2011 − 2021 (**Figure 3**, panel B).

The rates of several pregnancy complications, including GDM, anaemia and thrombocytopenia, increased nonlinearly. The prevalence of GDM increased by 33.32% (95% CI = 25.60, 47.58%) per year during 2010–2014 and then by 7.49% (95% CI = 5.47, 9.40%) per year during 2014–2021 (**Figure 3**, panel C). The prevalence of anaemia during pregnancy increased by 24.73% (95% CI = 15.85, 65.46%) per year during 2010–2015 but was stable after 2015 (**Figure 3**, panel D). The prevalence of thrombocytopenia exhibited three consequent trends, and the second period (2015–2019) showed a significant increasing trend with an APC of 27.84% (95% CI = 21.43, 41.52%) (**Figure 3**, panel E). The prevalence of HDP showed a bidirectional trend, with a declining trend of −7.11% (95% CI = −13.54, −3.85%)) per year during 2010–2015 but a rising trend of 4.40% (95% C I = 1.68, 9.61%) per year during 2015–2021 (**Figure 3**, panel F). In addition, the prevalence of thyroid dysfunction, including hypothyroidism and hyperthyroidism, also presented a significant upward trend and was maintained at a steady high-plateau level after 2015 (Table S2 in the **Online Supplementary Document**). This might be associated with an increase in the clinical detection of thyroid dysfunction during pregnancy. The detailed results of the APC and AAPC identified by Joinpoint regression analysis are presented in Table S2 in the **Online Supplementary Document**.

**Figure 4:**
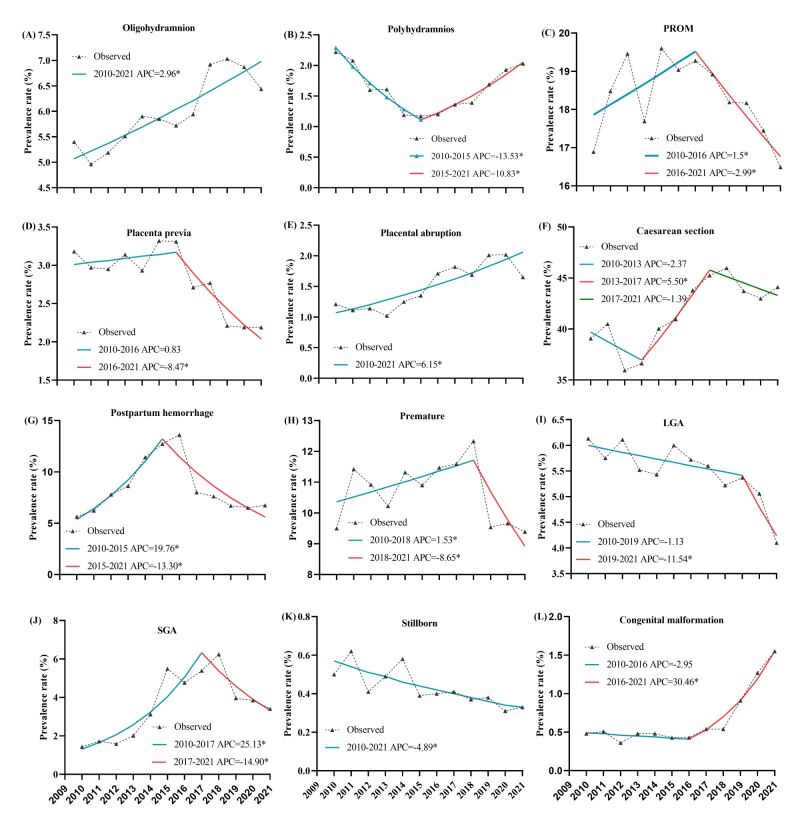
The temporal trends in prevalence of maternal and foetal adverse outcomes from 2010 to 2021. **Panel A.** Oligohydramnios. **Panel B.** Polyhydramnios. **Panel C.** PROM. **Panel D.** Placenta previa. **Panel E.** Placental abruption. **Panel F**. Caesarean section. **Panel G.** Postpartum haemorrhage. **Panel H.** Premature. **Panel I.** LGA. **Panel J.** SGA. **Panel K.** Stillbirth. **Panel L.** Congenital malformation. The number of Joinpoints were selected by the optimal models from Joinpoint regression analysis. *Indicates that the annual percent change (APC) is significantly different from zero at the α = 0.05 level. PROM – premature rupture of membranes, LGA – Large-for-gestational-age infants, SGA – small-for-gestational-age infants

The variation trends of different perinatal adverse outcomes are shown in **Figure 4**. The prevalence of oligohydramnios and placental abruption increased during 2010–2021, with AAPCs of 2.96% (95% CI = 1.39, 4.72%) and 6.15% (95% CI = 2.96, 10.25%), respectively (**Figure 4**, panels A and E). The prevalence of polyhydramnios and congenital malformations mainly increased after 2015 and 2016, respectively, and the APCs were 10.83% (95% CI = 5.38, 22.00%) and 30.46% (95% CI = 23.59, 44.05%), respectively (**Figure 4**, panels B and L). The rate of caesarean section showed a significantly increasing trend during 2013–2017, with an APC of 5.50% (95% CI = 3.44, 9.16%), and then slightly declined during 2017–2021 (**Figure 4**, panel F). The prevalence of stillbirth presented prominent downward trends of −4.89% (95% CI = −7.52, −2.39%) per year (**Figure 4**, panel K). The prevalence of placenta previa and LGA significantly declined by −8.47% (−23.15, −4.75%) and −11.54% (95% CI = −22.66, −1.70%) per year since 2016 and 2019, respectively (**Figure 4**, panels D and I). The prevalence of PROM, postpartum haemorrhage, prematurity and SGA all showed bidirectional trends, first increasing and then decreasing. These four adverse outcomes declined in 2016, 2015, 2018 and 2017, respectively, which could be attributed to the implementation of advanced obstetric technologies and standardised antenatal interventions (**Figure 4**, panels C, G, H and J).

**Figure 5:**
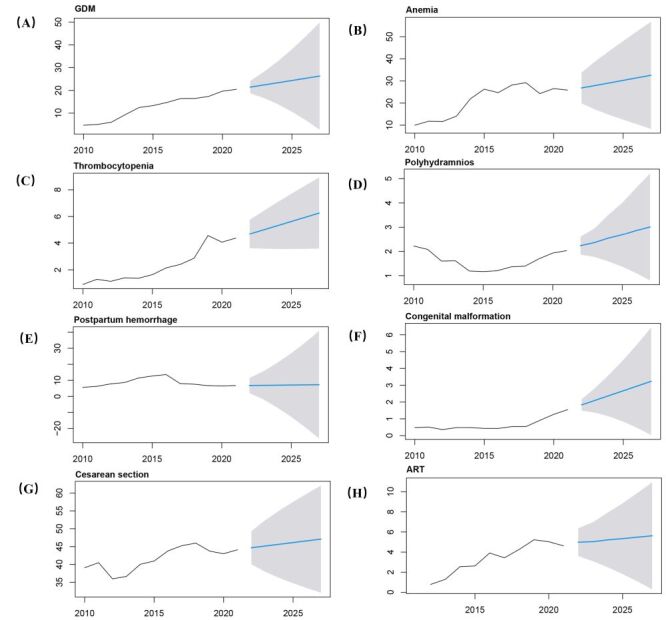
The predictions of the increased trend of adverse obstetric events in China from the ARIMA model. **Panel A.** The prevalence trend of GDM. **Panel B.** The prevalence trend of anaemia. **Panel C.** The prevalence trend of thrombocytopenia. **Panel D.** The prevalence trend of polyhydramnios. **Panel E.** The prevalence trend of postpartum haemorrhage. **Panel F.** The prevalence trend of congenital malformation. **Panel G.** The prevalence trend of caesarean section. **Panel H.** The prevalence trend of ART. The solid black line indicates the current prevalence of outcome events, the blue line indicates the predicted trend, and the shadow indicates the 95% confidence interval. GDM – gestational diabetes mellitus, ART – assisted reproductive technology

**Figure 6:**
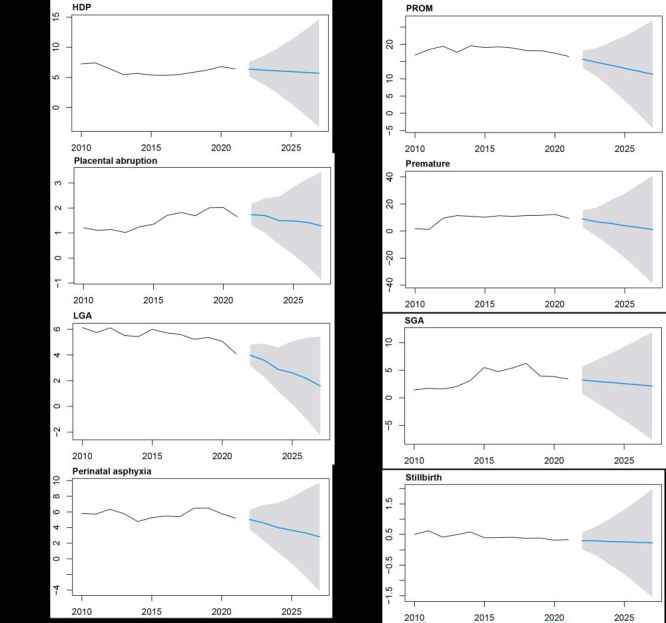
The predictions of the decreased trend of adverse obstetric events in China from the ARIMA model. **Panel A.** The prevalence trend of HDP. **Panel B.** The prevalence trend of PROM. **Panel C.** The prevalence trend of placental abruption. **Panel D.** The prevalence trend of premature. **Panel E.** The prevalence trend of LGA. **Panel F.** The prevalence trend of SGA. **Panel G.** The prevalence trend of perinatal asphyxia. **Panel H.** The prevalence trend of stillbirth. The solid black line indicates the current prevalence of outcome events, the blue line indicates the predicted trend, and the shadow indicates the 95% confidence interval. HDP – hypertensive disorders in pregnancy, PROM – premature rupture of membranes, LGA – Large-for-gestational-age infants, SGA – small-for-gestational-age infants

Furthermore, the ARIMA time series models were constructed using the yearly prevalences data from 2010 to 2021, and to predict the future obstetric characteristics and outcomes. The models predicted increasing trends in the GDM, anaemia, thrombocytopenia, polyhydramnios, postpartum haemorrhage, congenital malformation, caesarean section and ART intervention (**Figure 5**, panels A−H). Conversely, the ARIMA model exhibited declining trends in terms of HDP, PROM, placental abruption, premature, LGA, SGA, perinatal asphyxia and stillbirth (**Figure 6, **panels A−H). The optimal ARIMA models and detailed predicted prevalence of these adverse outcomes were presented in Table S3 in the **Online Supplementary Document**. Moreover, the sensitivity analysis conducted using time series data from 2010 to 2020 demonstrated the robustness of the ARIMA model in accurately predicting adverse obstetric outcomes trends (Table S4 in the **Online Supplementary Document**). Particularly noteworthy were the results indicating that the estimated prevalences of adverse outcomes align closely with the actual reported data for 2021(Figure S1 in the **Online Supplementary Document**).

### The independent and joint effects of advanced maternal age and ART treatment on adverse obstetric events

**Figure 7:**
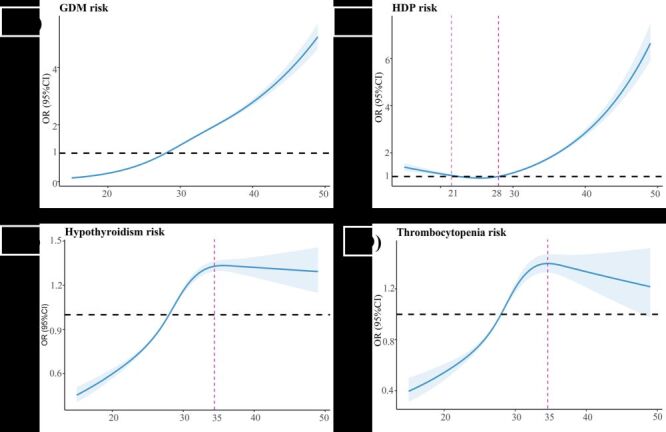
The dose-response relationship of maternal age and risk of pregnancy complications. **Panel A.** GDM. **Panel B.** HDP. **Panel C.** Hypothyroidism. **Panel D.** Thrombocytopenia. The blue lines were the fitted linear trend, and the shaded areas were 95% confidence interval. The red dotted lines indicate the point of inflection where a statistically significant difference occurs. GDM – gestational diabetes mellitus, HDP – hypertensive disorders in pregnancy

**Figure 8:**
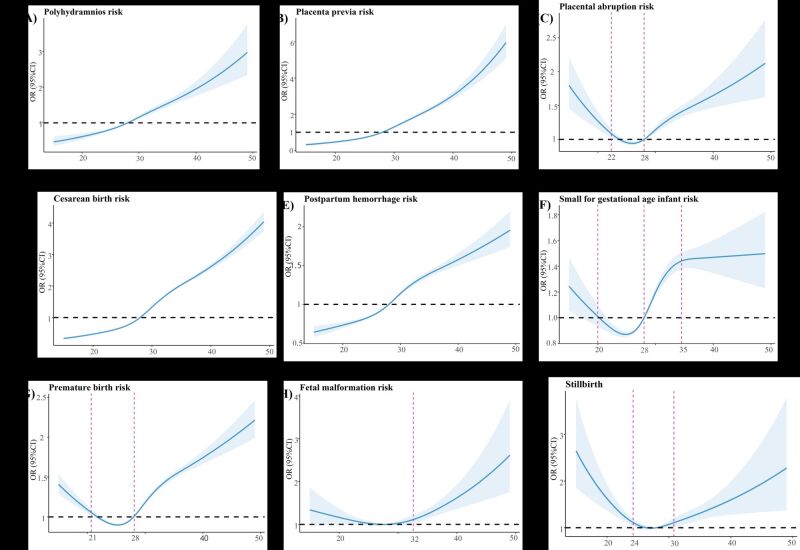
The dose-response relationship of maternal age and risk of maternal as well as foetal adverse outcomes. **Panel A.** Polyhydramnios. **Panel B.** Placenta previa. **Panel C.** Placental abruption. **Panel D.** Caesarean section. **Panel E.** Postpartum haemorrhage. **Panel F.** SGA. **Panel G.** Premature. **Panel H.** Congenital malformation. **Panel I.** Stillbirth. The blue lines were the fitted linear trend, and the shaded areas were 95% confidence interval. The red dotted lines indicate the point of inflection where a statistically significant difference occurs. SGA – small-for-gestational-age infants

The prevalence of several pregnancy complications and adverse perinatal outcomes exhibited significant variation across age subgroups (Table S5 in the **Online Supplementary Document**). Specifically, the PRR of hypothyroidism, GDM, HDP, anaemia, thrombocytopenia, placenta previa, placental abruption, caesarean section, postpartum haemorrhage, prematurity, and congenital malformations substantially increased after the maternal age of 30 years compared with 20–24 years (Table S6 in the **Online Supplementary Document**). Moreover, the restricted cubic spline model showed a nonlinear association of advanced maternal age with these pregnancy complications or adverse perinatal outcomes (Table S7 in the **Online Supplementary Document**). For example, the risks of GDM, HDP, polyhydramnios, placenta previa, caesarean section and postpartum haemorrhage increased with advanced maternal age (**Figure 7**, panels A and B, **Figure 8**, panels A, B, D, and E). The risk of hypothyroidism and thrombocytopenia showed an inverted L-shape pattern with maternal age, whereby the risk increased between 15 and 35 years of maternal age before plateauing after 35 years (**Figure 7**, panels C and D). However, several adverse perinatal outcomes, including placental abruption, SGA, premature, fetal malformations, and stillbirth, displayed bidirectional trends with increasing maternal age (**Figure 8**, panels C, F−I), and pregnant women who were younger or had AMA were at a higher risk of these adverse pregnancy outcomes.

In this retrospective study, 3.39% of pregnant women conceived through ART treatment. The average age of women who conceived through ART was significantly higher than that of women with SC (31.77 ± 3.91 vs 28.4 ± 4.85 years, *P* < 0.001). Moreover, women who conceived through ART were more frequently primiparous (60.97%) and had twin or multiple gestations (53.53%) than women with SC (Table S8 in the **Online Supplementary Document**). The prevalence of GDM, HDP, hypothyroidism, hyperthyroidism, anaemia, placenta previa, caesarean section, postpartum haemorrhage and SGA were significantly higher in women who conceived through ART in comparison to those with SC (**Table 1**). After adjusting for age, gravidity, parity and twin or multiple gestations, the risk of GDM (adjusted PRR = 1.29; 95% CI = 1.24, 1.34), HDP (adjusted PRR = 1.17; 95% CI = 1.10, 1.24), hypothyroidism (adjusted PRR = 1.48; 95% CI = 1.42, 1.53), hyperthyroidism (adjusted PRR = 1.38; 95% CI = 1.10, 1.67), anaemia (adjusted PRR = 1.19; 95% CI = 1.15, 1.24), placenta previa (adjusted PRR = 1.12; 95% CI = 1.02, 1.23), caesarean section (adjusted PRR = 1.14; 95% CI = 1.11, 1.17), postpartum haemorrhage (adjusted PRR = 1.42; 95% CI = 1.37, 1.47), and SGA (adjusted PRR = 1.26; 95% CI = 1.19, 1.32) was increased for women who conceived through ART compared with those with SC (**Table 1**). Stratification analysis revealed that ART treatment remained an independent risk factor for the aforementioned pregnancy complications and adverse perinatal outcomes in women with singleton gestation after adjustment for age, gravidity and parity (Table S9 in the **Online Supplementary Document**).

**Table 1:** The prevalence rate ratio (PRR) of pregnancy complications and perinatal outcomes of ART women compared to SC women

Adverse events	ART (n = 9494)		SC (n = 270 709)		Adjusted PRR* (95%CI)	*P*-value
	**Count**	**Incidence rate (%)**	**Count**	**Incidence rate (%)**		
Pregnancy complications						
GDM	2363	24.9	34 606	12.8	1.29 (1.24, 1.34)	<0.001
HDP	1373	14.5	15 661	5.8	1.17 (1.10, 1.24)	<0.001
Hypothyroidism	1910	20.1	23 621	8.7	1.48 (1.42, 1.53)	<0.001
Hyperthyroidism	81	0.9	1009	0.4	1.38 (1.10, 1.67)	0.025
Anaemia	2859	30.1	57 778	21.3	1.19 (1.15, 1.24)	<0.001
Thrombocytopenia	325	3.4	5434	2.0	1.11 (0.99, 1.23)	0.099
Perinatal outcomes						
Polyhydramnios	333	3.5	4109	1.5	1.12 (0.99, 1.26)	0.084
Oligohydramnios	401	4.2	16 361	6.0	1.1 (0.98, 1.21)	0.117
PROM	1503	15.8	50 024	18.5	0.8 (0.74, 0.86)	<0.001
Placenta previa	507	5.3	7435	2.7	1.12 (1.02, 1.23)	0.031
Placental abruption	201	2.1	4027	1.5	0.94 (0.78, 1.1)	0.468
Caesarean section	7708	81.2	109 175	40.3	1.14 (1.11, 1.17)	<0.001
Postpartum haemorrhage	2597	27.4	21 777	8.0	1.42 (1.37, 1.47)	<0.001
Perinatal asphyxia	438	4.6	15 060	5.6	0.98 (0.87, 1.09)	<0.001
LGA	240	2.5	15 194	5.6	0.94 (0.8, 1.08)	0.425
SGA	1498	15.8	6847	2.5	1.26 (1.19, 1.32)	<0.001
Premature	3651	38.5	26 496	9.8	0.96 (0.92, 1.00)	0.051
Stillbirth	50	0.5	1159	0.4	0.41 (0.09, 0.73)	<0.001
Congenital malformation	67	0.7	1753	0.6	1.01 (0.72, 1.31)	0.929

ART – assisted reproductive technology, SC – spontaneous conception, GDM – gestational diabetes mellitus, HDP – hypertensive disorders in pregnancy, PROM – premature rupture of membranes, LGA – large-for-gestational-age, SGA – small-for-gestational-age

*The Poisson regression models were used to examine the association between ART intervention and pregnancy complications as well as adverse perinatal outcomes. The maternal age, gravidity, parity, twin or multiple gestations were adjusted.

**Figure 9:**
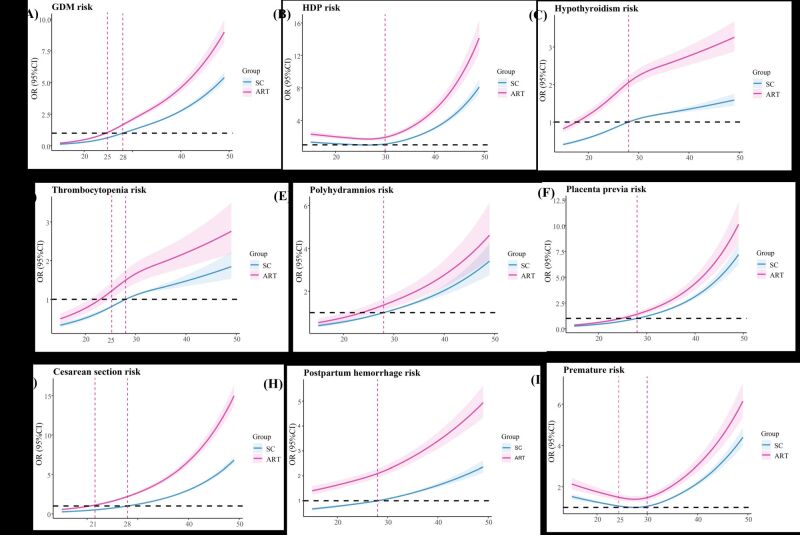
The dose-response relationship of maternal age and pregnancy complications as well as adverse perinatal outcomes in ART and SC groups. **Panel A.** Risk of GDM with maternal age in ART and SC women. **Panel B.** Risk of HDP with maternal age in ART and SC women. **Panel C.** Risk of hypothyroidism with maternal age in ART and SC women. **Panel D.** Risk of thrombocytopenia with maternal age in ART and SC women. **Panel E.** Risk of polyhydramnios with maternal age in ART and SC women. **Panel F.** Risk of placenta previa with maternal age in ART and SC women. **Panel G.** Risk of caesarean section with maternal age in ART and SC women. **Panel H.** Risk of postpartum haemorrhage with maternal age in ART and SC women. **Panel I**. Risk of premature with maternal age and risk of stillbirth. The blue lines were the fitted linear trend of SC group, red lines were the fitted linear trend of ART group the and the shaded areas were 95% confidence interval. The red dotted lines indicate the point of inflection where a statistically significant difference occurs. ART – assisted reproductive technology, SC – spontaneous conception, GDM – gestational diabetes mellitus, HDP – hypertensive disorders in pregnancy

The dose-response relationship between maternal age and pregnancy complications as well as adverse perinatal outcomes in the ART and SC subgroups were further analysed using the restricted cubic spline model. The results indicated that maternal age was nonlinearly associated with adverse outcomes, such as GDM, HDP, hypothyroidism, thrombocytopenia, polyhydramnios, placenta previa, caesarean section, postpartum haemorrhage and stillbirth, in both the ART and SC groups, but the risk effect of advanced maternal age was more pronounced among women undergoing ART treatment (**Figure 9**, panels A−I, Table S10 in the **Online Supplementary Document**). Further interactive analysis observed no significant interaction between the two factors in relation to adverse outcomes (Table S11 in the **Online Supplementary Document**).

### The effect of prior caesarean section on next pregnancy complications and adverse perinatal outcomes

In this study, a total of 60.42% (16 925 / 280 203) participants provided comprehensive information regarding their gravidity and parity (Table S1 in the **Online Supplementary Document**). Among these participants, there were 74 780 multiparas identified, out of which 29.14% (21 793 / 74 780) had previously undergone caesarean sections. We further analysed the effect of prior caesarean section on pregnancy complications on adverse perinatal outcomes (Table S12 in the **Online Supplementary Document**). The findings revealed a significantly higher risk of hypothyroidism (adjusted PRR = 1.69; 95% CI = 1.62, 1.77), hyperthyroidism (adjusted PRR = 1.55; 95% CI = 1.21, 1.98), GDM (adjusted PRR = 1.48; 95% CI = 1.42, 1.55), thrombocytopenia (adjusted PRR = 1.31; 95% CI = 1.19, 1.43), and anaemia (adjusted PRR = 1.76; 95% CI = 1.7, 1.83) in multiparas with a history of caesarean section compared to those without such history. However, the risk of HDP (adjusted PRR = 0.78; 95% CI = 0.73, 0.83), PROM (adjusted PRR = 0.42; 95% CI = 0.4, 0.44), placenta previa (adjusted PRR = 0.47; 95% CI = 0.43, 0.51), placental abruption (adjusted PRR = 0.38; 95% CI = 0.33, 0.44), perinatal asphyxia (adjusted PRR = 0.66; 95% CI = 0.60, 0.72), LGA (adjusted PRR = 0.69; 95% CI = 0.64, 0.74), stillbirth (adjusted PRR = 0.37; 95% CI = 0.27, 0.50), and premature (adjusted PRR = 0.86; 95% CI = 0.82, 0.90) significantly decreased in multiparas with a history of caesarean section. The relationship between caesarean section history and adverse outcomes remained consistent with that of the general population, irrespective of age group (above or below 35 years), as demonstrated by stratified analysis (Table S13 in the **Online Supplementary Document**).

### The cumulative effect of comorbid pregnancy complications on adverse perinatal outcomes

**Figure 10:**
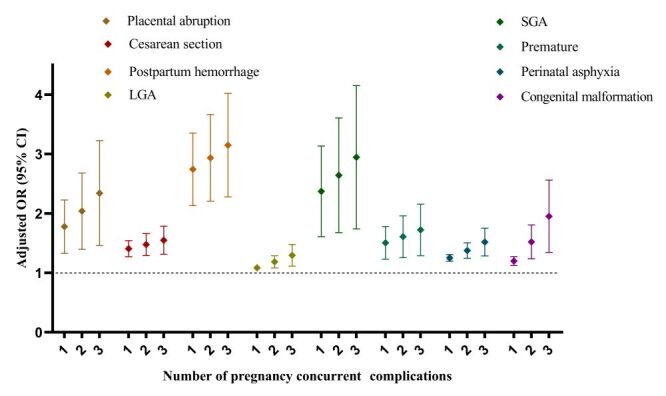
The cumulative effect of comorbid pregnancy complications for adverse perinatal outcomes. LGA – large for gestational age infants, SGA – small for gestational age infants

To evaluate the cumulative effect of comorbid pregnancy complications on perinatal outcomes, the complication status was categorised into four tiers: none, one complication, two complications and three or more complications. The findings revealed that 32.1% of pregnant women had one complication, 8.7% had two complications and 1.5% had three or more complications during pregnancy (Table S14 in the **Online Supplementary Document**). Furthermore, the prevalence of comorbid pregnancy complications significantly increased with AMA, particularly among women over 40 years of age, where it could reach up to 20% (Table S14 in the **Online Supplementary Document**). Compared to pregnant women without pregnancy complications, in those with complications, the risk of placental abruption, caesarean section, perinatal asphyxia, LGA, SGA, postpartum haemorrhage, prematurity and congenital malformations significantly increased with the number of concurrent pregnancy complications after adjustment for maternal age, ART treatment, gravidity and parity (**Figure 10**, Table S15 in the **Online Supplementary Document**).

## DISCUSSION

Obstetric safety is a crucial long-term strategic issue that is closely linked to maternal and neonatal health as well as population quality, and warrants great attention. In this retrospective, multicentre cross-sectional study, we provided a comprehensive overview of trends in obstetric population characteristics and nineteen adverse obstetric events from 2010 to 2021 in eastern China. The main findings indicated significantly increased trends of several adverse maternal and neonatal outcomes, despite the continuous improvement in Chinese maternal and child health care during the past decade.

HDP and GDM were two important pregnancy complications that imposed a significant disease burden worldwide. HDP affected up to 10% of pregnancies worldwide [35]. The prevalence of HDP in China was about 5−10%, and it was one of the top three causes of maternal death [36]. The global prevalence of GDM had also steadily increased in the past four decades, and approximately 9−25% of pregnancies were affected by GDM [37]. A systematic review and meta-analysis of 25 studies in China found that the prevalence of GDM was 14.8% (95% CI = 12.8, 16.7%) according to International Association of Diabetes and Pregnancy Study Groups (IADPSG) criteria [38]. The prevalence rates of HDP and GDM consistently observed in our data were 6.1 and 13.2%, respectively. Moreover, the prevalence of anaemia, thrombocytopenia and hypothyroidism were 21.6, 2.35, and 9.1% respectively. The prevalence of anaemia among 18 948 443 pregnant females aged 15 to 49 years, as recorded in the Chinese Hospital Quality Monitoring System (HQMS) established by the National Health Commission, was found to be 17.77% during the period from 2016 to 2019 [21]. Previous studies also found that approximately 5 to 10% of women develop thrombocytopenia during pregnancy or immediately after delivery [39]. Low platelet count is often an occasional feature of pregnancy, but it might also provide a biomarker for concurrent systemic or gestational disease. The prevalence of thrombocytopenia during pregnancy was relatively lower according to our data. This disparity was attributed to variations in population demographics and the frequency of platelet monitoring during pregnancy and prenatal care. Notably, we further highlighted the cumulative effects of above concurrent complications on the risk of adverse perinatal outcomes, which added to the intricacy of obstetric health issues.

The neonatal outcomes of premature, perinatal asphyxia, LGA infants, SGA infants, stillbirth and congenital malformation were significant clinical concerns regarding the health problems of offspring. Preterm birth is the leading cause of death for children under five worldwide. The global preterm birth rate varied from 8.7 to 13.4% by worldwide region in 2014 [40]. Furthermore, the age-standardised incidence rates of preterm birth increased by a mean of 0.25% in high sociodemographic index regions from 1990 to 2019 [41]. China's National Maternal Near Miss Surveillance System (NMNMSS) reported that the overall preterm birth rate increased from 5.9 to 6.4% from 2012 to 2018 [42]. Meanwhile, the global prevalence of neonatal asphyxia in full-term newborns ranges from 0.5 to 1.0% [43], and a multicentre retrospective cohort study conducted in China reported a prevalence rate of 1.3% for neonatal asphyxia [44]. The present study, however, revealed a prevalence rate of perinatal asphyxia reaching up to 5.7%, primarily due to the inclusion of both cases of foetal distress and neonatal asphyxia. Currently, abnormal foetal growth still presented vital clinical and social implications, and 10 and 9% pregnancies were affected by SGA and LGA, respectively [45,46]. Moreover, the lack of accurate examination technologies for detecting SGA and LGA infants, as well as effective interventions to reverse these abnormal foetal growth conditions, further exacerbates the situation. The global stillbirth rate was estimated to be 18.4 per 1000 births; it was projected to decrease to 12 per 1000 births by 2030. However, China continued to face a persistently high stillbirth rate, ranking among the top five globally, with significant regional disparities ranging from approximately 1.5 to 5.5% [47]. The prevalence of stillbirth was lower in our study, which may be attributed to the solid foundation of economic development and health care provision in eastern China. However, this study observed significant increase in the prevalence of congenital malformation since 2017. The trend was consistent with another observational study using data from birth defects surveillance system in Zhejiang Province, China [26]. Overall, the main findings were generally consistent with the prevailing trend of adverse obstetric outcomes reported both domestically and internationally in recent years. However, it was important to take into account variations in regional economic development levels, maternal and child health care systems, and diagnostic criteria when considering the generalisability of the findings in this study.

In this study, we observed that AMA and ART treatment were independent risk factors for several adverse outcomes. The underlying mechanism explaining the association between AMA and adverse pregnancy outcomes remained incompletely elucidated. Previous evidence indicated that AMA was associated with dysfunctional trophoblast cell function and premature senescence of the placenta. Age-related abnormal placental aging, vascular differences or placental hypoxia could lead to a reduced maximum uteroplacental blood supply as well adverse outcomes, such as early-onset preeclampsia, premature birth, intrauterine growth restriction and even stillbirth [48,49]. Moreover, AMA is generally recognised as a pancreatic hit before pregnancy that contributes to age-associated deterioration of pancreatic β-cells [50]. Consistent with previous studies, our findings suggested that the prevalence of HDPs, GDM, polyhydramnios, placenta previa and foetal malformations increased with maternal age. In addition, the use of ART treatment was often increased due to the negative impact of advanced maternal age (AMA) on fertility [51]. The increasing prevalence of AMA and the corresponding rise in ART treatment had emerged as a burgeoning public health concern in China. It was well established that pregnancies conceived through ART were associated with a higher risk of pregnancy complications and adverse perinatal outcomes than pregnancies conceived spontaneously [52,53]. In fact, it was imperative to consider the inherent characteristics of ART patients, including underlying infertility, other preexisting medical conditions and advanced maternal age, before evaluating the substantial risk of ART interventions for maternal and foetal outcomes in future studies.

In this study, the ARIMA models offered a predictive overview of common pregnancy complications and perinatal outcomes trends. The ARIMA model is a renowned and versatile class of forecasting model that utilises historical data to generate predictions. The ARIMA model offers the advantage of effectively predicting time series data and accommodating nonlinear relationships and unconventional data. Additionally, it can be utilised for stationarity testing and outlier detection in time series analysis. By considering lagged values and differencing, the ARIMA model excels at capturing underlying trends within the data. In previous studies, the ARIMA model was commonly used to study the trend of infectious diseases and chronic non-infectious disease, such as the varicella [54], pulmonary tuberculosis [55], malaria [56], pertussis [57], Corona Virus Disease 2019 (COVID-19) [58], chronic kidney disease [59], diabetes [60], cardiovascular diseases [61] and economic burden of these diseases. In this study, the Ljung-Box test and sensitivity analysis demonstrated that the model exhibited a good fit and accurately predicted outcomes. However, the capability of ARIMA models for long-term prediction is limited, as it solely relies on lag and differencing of data while neglecting other potential influencing factors. Moreover, the ARIMA model generally exhibited suboptimal fitting performance when dealing with non-stationary and nonlinear data, necessitating additional data processing and transformation that inevitably impact the accuracy of predicted values to a certain extent. Consequently, the well-fitted ARIMA models constructed basing on regularly updated could provide valuable insight for preemptive measures and public health guidance for maternal and neonatal Health.

Furthermore, the diagnostic criteria of GDM were changed during 2010 and 2021. The diagnostic criteria for GDM using the International Association of Diabetes and Pregnancy Study Groups (IADPSG) guidelines from 2010 have gradually gained recommendation in China since 2011. The GDM health industry standards issued by the Ministry of Health of China in 2011, the Chinese Guidelines for the Diagnosis and Treatment of Diabetes Mellitus from 2013, and the Obstetrics and Gynecology Subcommittee of the Chinese Medical Association in 2014 all adopted the IADPSG standard. These criteria advocate a ‘one-step’ approach (75 g oral glucose tolerance test) for screening at 24 − 28 weeks of gestation, leading to increased detection rates of GDM among pregnant women [62,63]. Consistently, the Joinpoint model revealed that the GDM increased by 33.32% per year during 2010-2014 and then by 7.49% per year during 2014 − 2021. The observable uptrend during 2010-2014 indicated the high detection rate of new diagnosis. The prevalence of GDM continued to rise steadily even after the implementation of one-step diagnosis.

Pregnancy complications and adverse perinatal outcomes are complex public health issues that not only lead to a dramatic increase in the national disease burden, but also have a profound impact on the long-term health of pregnant women and their offspring [64]. The primary public health implication of this study is to visually depict the current alarming obstetric safety for health care facility decision-makers and providers. With the rise in maternal age and increased ART treatment, obstetric safety will encounter increasingly complex challenges. The health care decision-makers and providers should enhance their awareness of these potential risks. In addition to addressing individual lifestyle and behavioural factors that impact health, it is imperative to develop multi-level public health strategies that encompass prevention and treatment at the individual level [37]. These strategies should also involve collaborative partnerships across multiple sectors to enhance social and community conditions for pregnant women.

The study’s notable strength lies in its comprehensive examination of the prevalences and temporal trends of common pregnancy complications and perinatal outcomes in China from 2010 to 2021 based on a relatively large multicentre sample. An additional strength of this study is the innovative analysis of independent risk effects and synergistic effects of maternal age and ART on various obstetric events, providing profound insights into the obstetrical safety of delayed childbearing and ART treatment, which are widely concerning in modern times. Furthermore, this study also stands out as one of the few to investigate the cumulative impact of comorbid pregnancy complications on diverse adverse perinatal outcomes. This highlights the need for obstetrical experts and researchers to prioritise the prevention and management of such coexisting pregnancy complications.

Nevertheless, some limitations must be considered in this study. First, we collected obstetric delivery data from four Tier 3 hospitals in Zhejiang Province, China. However, it was important to note that our access to delivery information from community hospitals was limited, which could introduce potential selection bias, such as health care access bias and admission rate bias. Second, the present study was a multi-centre retrospective cross-sectional study. The completeness and accuracy of disease diagnosis might vary among different electronic medical record systems, and International Classification of Diseases (ICD) coding also changed between 2010 − 2021. These factors collectively had the potential to introduce information bias. Third, the adverse perinatal outcomes were multifactorial, and the socioeconomic status, environmental exposure, genetic predispositions, life style, physiological characteristics, and antenatal care could be important confounding factors influencing maternal and neonatal health. Therefore, a prospective cohort study is warranted to ascertain the condition of obstetric safety and identify associated risk factors.

## CONCLUSIONS

In conclusion, this study provided a panoramic view of the current state of obstetric characteristics and adverse outcomes in eastern China during the decade marked by drastic changes in the fertility policy (from 2010 to 2021). The findings revealed significant increases in the prevalence of AMA and ART treatment during this period. The prevalences of GDM, anaemia, thrombocytopenia, oligohydramnios and placental abruption increased nonlinearly and concurrently. The prevalence of PROM, placenta previa, postpartum haemorrhage, premature birth and LGA presented bidirectional trends and showed a recent decline. AMA and ART treatment could be independent risk factors for pregnancy complications and adverse perinatal outcomes. Twin or multiple gestations and comorbid pregnancy complications exacerbate the complexity of pregnancy status and pose more challenges to maternal and foetal safety. Accurate early prediction models are warranted to promote early prediction of adverse obstetric events and enhance individualised management for high-risk pregnant women.

## Additional material


Online Supplementary Document
