# Supplementary material for: Trajectories tracking of maternal and neonatal health in eastern China from 2010 to 2021: A multicentre cross-sectional study
Source: J Glob Health. 2024 Mar 22;14:04069. doi: 10.7189/jogh.14.04069 (PMC10958191; doi:10.7189/jogh.14.04069)
Supplement: Online Supplementary Document [file jogh-14-04069-s001.pdf]

Appendix S1

Criteria of pregnancy complications and adverse perinatal outcomes

The fasting plasma glucose (FPG) test was carried out during the first trimester of gestation to leave out the pre-pregnancy diabetes, and the overt diabetes mellitus (DM) of FPG>7.0 mmol/L was excluded. The gestational diabetes mellitus was diagnosed by two-step 50-g glucose challenge test (GCT) or one-step 75-g oral glucose tolerance test (OGTT) at 24-28 weeks of gestation. The 1-hour glucose value of 50-g GCT ≥11.2 mmol/L or 1-h glucose value ≥ 7.8 but subsequent 75-g OGTT had two or more items reached the standard criterions: FPG ≥ 5.6, 1h value ≥10.3, 2h value ≥8.6 or 3h value ≥ 6.7 was identified as GDM. The glucose value of one-step 75-g OGTT meeting or exceeding one of the following criteria: 0 h value ≥ 5.1 mmol/L, 1 h value ≥10.0 mmol/L, or 2 h value ≥ 8.5 mmol/L was diagnosed as GDM [1,2].

Hypertensive disorders of pregnancy were defined as systolic blood pressure (SBP) ≥140 mmHg and/or diastolic blood pressure (DBP) ≥90 mmHg, and it included four categories: 1) gestational hypertension; 2) pre-eclampsia (PE)/eclampsia; 3) chronic hypertension; and 4) pre-eclampsia/ eclampsia variants superimposed on chronic hypertension [3,4].Gestational hypertension that develops after the 20th week of pregnancy in women who have had no high BP values before. Blood pressure usually returns to normal during the first 42 days postpartum. Chronic hypertension which is pre-existing hypertension in most of the cases but could also be secondary due to various causes and may be associated with proteinuria. PE/eclampsia is GH associated with significant proteinuria (>0.3 g/24 h or ⩾30 mg/mmol urinary protein) or even seizures (eclampsia).

Hypothyroidism was defined by high serum levels of thyroid stimulating hormone (TSH) with low levels of free T4 [5]. Hyperthyroidism was defined as low serum levels of TSH with elevated levels of free T4[5].

Thrombocytopenia during pregnancy was defined as a platelet count less than 150 $x10^9$/L [6].

Anaemia was defined as hemoglobin concentrations of less than 110 g/L (to convert g/L to g/dL, divide by 10.0) in pregnant women[7].

Polyhydramnios could be defined by ultrasonography, and when the maximum depth of amniotic fluid (MVP)>8 cm or amniotic fluid index (AFI)>24 cm was defined as

polyhydramnios[8]. Oligohydramnion was defined as MVP<2 cm or AFI<5 cm.

Premature rupture of the membranes (PROM), defined as rupture of the membranes before the onset of labor

Placenta previa was defined as the placenta abnormally covered the endocervical os [9].

Placental abruption was the premature separation of the placenta from its uterine attachment before the delivery of a fetus [10].

Perinatal asphyxia included fetal distress and neonatal asphyxia in this study.

Premature birth was defined preterm birth as births before 37 completed weeks of gestation or fewer than 259 days from the first date of a woman's last menstrual period according to World Health Organization (WHO) criterion

Small-for-gestational-age infant (SGA) was defined as birth weight weight<2500 g according to World Health Organization (WHO) criterion

Large-for-gestational-age infant (LGA) was defined as birth weight ≥4000g according to World Health Organization (WHO) criterion

Stillborn defined as fetal death in utero or at delivery during perinatal period.

Postpartum hemorrhage were traditionally defined as blood loss of more than 500 ml after a vaginal delivery or more than 1000 ml after a cesarean delivery.

A variety of fetal/neonatal birth malformations described in clinical reports were included in this study. Fetal malformations in this study included these located in limbs, fingers, toes, heart, kidneys, urethra, spinal brain, ear, eye, visceral transposition, Down syndrome, cleft lip and palate, chromosomal abnormalities, and other unspecified fetal malformations.

Reference

1. Sweeting A, Wong J, Murphy HR, Ross GP. A Clinical Update on Gestational Diabetes Mellitus. *Endocr Rev*. Sep 26 2022;43(5):763-793. doi:10.1210/endrev/bnac003
2. Zhu H, Zhao Z, Xu J, et al. The prevalence of gestational diabetes mellitus before and after the implementation of the universal two-child policy in China. *Front Endocrinol (Lausanne)*. 2022;13:960877. doi:10.3389/fendo.2022.960877
3. Garovic VD, White WM, Vaughan L, et al. Incidence and Long-Term Outcomes of Hypertensive Disorders of Pregnancy. *J Am Coll Cardiol*. May 12 2020;75(18):2323-2334. doi:10.1016/j.jacc.2020.03.028
4. Antza C, Cifkova R, Kotsis V. Hypertensive complications of pregnancy: A clinical overview. *Metabolism*. Sep 2018;86:102-111. doi:10.1016/j.metabol.2017.11.011
5. Lee SY, Pearce EN. Assessment and treatment of thyroid disorders in pregnancy and the

postpartum period. *Nat Rev Endocrinol*. Mar 2022;18(3):158-171. doi:10.1038/s41574-021-00604-z
6. Pishko AM, Levine LD, Cines DB. Thrombocytopenia in pregnancy: Diagnosis and approach to management. *Blood Rev*. Mar 2020;40:100638. doi:10.1016/j.blre.2019.100638
7. Shi H, Chen L, Wang Y, et al. Severity of Anemia During Pregnancy and Adverse Maternal and Fetal Outcomes. *JAMA Netw Open*. Feb 1 2022;5(2):e2147046. doi:10.1001/jamanetworkopen.2021.47046
8. Society for Maternal-Fetal Medicine . Electronic address pso, Dashe JS, Pressman EK, Hibbard JU. SMFM Consult Series #46: Evaluation and management of polyhydramnios. *Am J Obstet Gynecol*. Oct 2018;219(4):B2-B8. doi:10.1016/j.ajog.2018.07.016
9. Svanvik T, Jacobsson AK, Carlsson Y. Prenatal detection of placenta previa and placenta accreta spectrum: Evaluation of the routine mid-pregnancy obstetric ultrasound screening between 2013 and 2017. *Int J Gynaecol Obstet*. Jun 2022;157(3):647-653. doi:10.1002/ijgo.13876
10. Brandt JS, Ananth CV. Placental abruption at near-term and term gestations: pathophysiology, epidemiology, diagnosis, and management. *Am J Obstet Gynecol*. May 2023;228(5S):S1313-S1329. doi:10.1016/j.ajog.2022.06.059

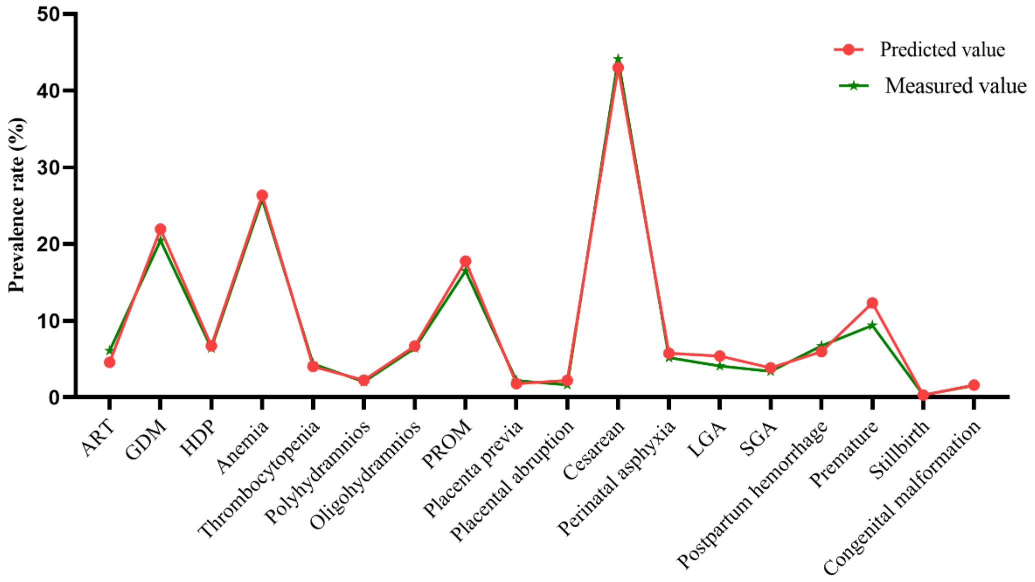

Figure S1 The rate of adverse obstetric outcomes from actual recorded data and estimated data using ARIMA models in 2021

ART, assisted reproductive technology; GDM, gestational diabetes mellitus; HDP, hypertensive disorders in pregnancy; PROM, premature rupture of membranes; LGA, Large-for-gestational-age infants; SGA, small-for-gestational-age infants.

Table S1 The Characteristics of pregnant women and obstetric events profiles from 2010 to 2021

Characteristics		Total	2010	2011	2012	2013	2014	2015	2016	2017	2018	2019	2020	2021
**Maternal age (year)**		28.52±4.86	27.13±5.019	27.3±5.018	27.32±4.718	27.44±4.734	27.75±4.524	28.24±4.738	28.77±4.615	29.33±4.859	29.46±4.816	29.54±4.679	29.68±4.769	29.85±4.681
	20-24	46802 (16.7)	4943 (26.8)	5445 (26.1)	5395 (23.5)	4987 (22)	4673 (18.4)	3976 (16.4)	3936 (13.6)	3403 (12.5)	2944 (12.2)	2722 (11.3)	2269 (11.3)	2109 (9.94)
	<20	7036 (2.5)	822 (4.5)	921 (4.4)	881 (3.8)	863 (3.8)	721 (2.8)	656 (2.7)	493 (1.7)	415 (1.5)	359 (1.5)	320 (1.3)	301 (1.5)	284 (1.34)
	25-29	119964 (42.8)	7679 (41.6)	8777 (42.1)	10180 (44.4)	10108 (44.5)	12126 (47.8)	11105 (45.8)	13504 (46.8)	11437 (42.1)	9667 (40.2)	9768 (40.4)	7626 (37.9)	7987 (37.65)
	30-34	73167 (26.1)	3327 (18)	3770 (18.1)	4735 (20.6)	4937 (21.7)	5787 (22.8)	5997 (24.7)	7543 (26.1)	7740 (28.5)	7259 (30.2)	7716 (31.9)	6778 (33.7)	7578 (35.72)
	35-39	27821 (9.9)	1411 (7.6)	1535 (7.4)	1408 (6.1)	1475 (6.5)	1741 (6.9)	2152 (8.9)	2915 (10.1)	3520 (12.9)	3189 (13.3)	3087 (12.8)	2674 (13.3)	2714 (12.79)
	≥40	5409 (1.9)	268 (1.5)	391 (1.9)	342 (1.5)	347 (1.5)	328 (1.3)	362 (1.5)	475 (1.6)	677 (2.5)	617 (2.6)	573 (2.4)	487 (2.4)	542 (2.55)
**Gravidity**	One	64152 (37.9)	3794 (36.95)	4933 (40.46)	4939 (40.7)	5397 (41.99)	7139 (42.19)	5961 (37.79)	5810 (37.18)	5107 (33.65)	4820 (36)	5756 (35.94)	5013 (34.79)	5483 (37.34)
	Two	46435 (27.4)	2799 (27.26)	3272 (26.84)	3331 (27.45)	3394 (26.4)	4581 (27.07)	4336 (27.49)	4310 (27.58)	4285 (28.24)	3625 (27.08)	4501 (28.1)	3995 (27.72)	4006 (27.28)
	Three or more	58861 (34.7)	3675 (35.79)	3987 (32.7)	3865 (31.85)	4063 (31.61)	5202 (30.74)	5477 (34.72)	5507 (35.24)	5783 (38.11)	4943 (36.92)	5760 (35.96)	5403 (37.49)	5196 (35.38)
**Parity**	Primipara	94516 (55.9)	5958 (58.41)	7434 (61.64)	7502 (62.36)	8057 (62.94)	10628 (62.81)	8968 (56.85)	8364 (53.52)	7232 (47.66)	6624 (49.48)	8306 (51.86)	7446 (51.67)	7997 (54.46)
	Multipara	74577 (44.1)	4243 (41.59)	4627 (38.36)	4529 (37.64)	4744 (37.06)	6294 (37.19)	6806 (43.15)	7263 (46.48)	7943 (52.34)	6764 (50.52)	7711 (48.14)	6965 (48.33)	6688 (45.54)
**ART intervention**	Yes	9494 (3.4)	/	162 (0.78)	295 (1.29)	582 (2.56)	665 (2.62)	948 (3.91)	989 (3.43)	1161 (4.27)	1255 (5.22)	1216 (5.03)	930 (4.62)	1291 (6.09)
	No	270709 (96.6)	18451 (100)	20678 (99.22)	22646 (98.71)	22136 (97.44)	24711 (97.38)	23301 (96.09)	27877 (96.57)	26031 (95.73)	22780 (94.78)	22970 (94.97)	19205 (95.38)	19923 (93.91)
**Twin or multiple gestations**	Yes	17658 (6.3)	1003 (5.44)	1468 (7.04)	1366 (5.95)	1269 (5.59)	1440 (5.67)	1654 (6.82)	2107 (7.3)	2113 (7.77)	2056 (8.55)	1173 (4.85)	895 (4.44)	1114 (5.25)
	No	262545 (93.7)	17448 (94.56)	19372 (92.96)	21575 (94.05)	21449 (94.41)	23936 (94.33)	22595 (93.18)	26759 (92.7)	25079 (92.23)	21979 (91.45)	23013 (95.15)	19240 (95.56)	20100 (94.75)
**HBV infection**	Yes	4059 (1.4)	157 (0.85)	309 (1.48)	290 (1.26)	361 (1.59)	389 (1.53)	470 (1.94)	638 (2.21)	416 (1.53)	236 (0.98)	214 (0.88)	223 (1.11)	356 (1.68)
	No	276144 (98.6)	18294 (99.15)	20531 (98.52)	22651 (98.74)	22357 (98.41)	24987 (98.47)	23779 (98.06)	28228 (97.79)	26776 (98.47)	23799 (99.02)	23972 (99.12)	19912 (98.89)	20858 (98.32)
**Hypothyroidism**	Yes	25531 (9.1)	4 (0.02)	3 (0.01)	12 (0.05)	55 (0.24)	956 (3.77)	3163 (13.04)	4758 (16.48)	2957 (10.87)	3524 (14.66)	3697 (15.29)	2794 (13.88)	3608 (17.01)
	No	254672 (90.9)	18447 (99.98)	20837 (99.99)	22929 (99.95)	22663 (99.76)	24420 (96.23)	21086 (86.96)	24108 (83.52)	24235 (89.13)	20511 (85.34)	20489 (84.71)	17341 (86.12)	17606 (82.99)
**Hyperthyroidism**	Yes	1090 (0.4)	5 (0.03)	8 (0.04)	8 (0.03)	19 (0.08)	152 (0.6)	116 (0.48)	110 (0.38)	116 (0.43)	128 (0.53)	156 (0.65)	107 (0.53)	165 (0.78)
	No	279113 (99.6)	18446 (99.97)	20832 (99.96)	22933 (99.97)	22699 (99.92)	25224 (99.4)	24133 (99.52)	28756 (99.62)	27076 (99.57)	23907 (99.47)	24030 (99.35)	20028 (99.47)	21049 (99.22)
**GDM**	Yes	36969 (13.2)	867 (4.7)	1049 (5.03)	1380 (6.02)	2119 (9.33)	3169 (12.49)	3227 (13.31)	4243 (14.7)	4468 (16.43)	3951 (16.44)	4197 (17.35)	3961 (19.67)	4338 (20.45)
	No	243234 (86.8)	17584 (95.3)	19791 (94.97)	21561 (93.98)	20599 (90.67)	22207 (87.51)	21022 (86.69)	24623 (85.3)	22724 (83.57)	20084 (83.56)	19989 (82.65)	16174 (80.33)	16876 (79.55)
**HDP**	Yes	17034 (6.1)	1336 (7.24)	1546 (7.42)	1485 (6.47)	1244 (5.48)	1438 (5.67)	1309 (5.4)	1539 (5.33)	1493 (5.49)	1407 (5.85)	1504 (6.22)	1369 (6.8)	1364 (6.43)
	No	263169 (93.9)	17115 (92.76)	19294 (92.58)	21456 (93.53)	21474 (94.52)	23938 (94.33)	22940 (94.6)	27327 (94.67)	25699 (94.51)	22628 (94.15)	22682 (93.78)	18766 (93.2)	19850 (93.57)
**Anemia**	Yes	60637 (21.6)	1847 (10.01)	2456 (11.79)	2672 (11.65)	3199 (14.08)	5565 (21.93)	6374 (26.29)	7132 (24.71)	7665 (28.19)	7021 (29.21)	5872 (24.28)	5347 (26.56)	5487 (25.86)
	No	219566 (78.4)	16604 (89.99)	18384 (88.21)	20269 (88.35)	19519 (85.92)	19811 (78.07)	17875 (73.71)	21734 (75.29)	19527 (71.81)	17014 (70.79)	18314 (75.72)	14788 (73.44)	15727 (74.14)
**Thrombocytopenia**	Yes	6575 (2.35)	168 (0.91)	269 (1.29)	261 (1.14)	317 (1.4)	348 (1.37)	397 (1.64)	621 (2.15)	653 (2.4)	691 (2.87)	1104 (4.56)	819 (4.07)	927 (4.37)
	No	273628 (97.65)	18283 (99.09)	20571 (98.71)	22680 (98.86)	22401 (98.6)	25028 (98.63)	23852 (98.36)	28245 (97.85)	26539 (97.6)	23344 (97.13)	23082 (95.44)	19316 (95.93)	20287 (95.63)
**Polyhydramnios**	Yes	4442 (1.6)	410 (2.22)	433 (2.08)	368 (1.6)	366 (1.61)	301 (1.19)	284 (1.17)	347 (1.2)	371 (1.36)	334 (1.39)	409 (1.69)	389 (1.93)	430 (2.03)
	No	275761 (98.4)	18041 (97.78)	20407 (97.92)	22573 (98.4)	22352 (98.39)	25075 (98.81)	23965 (98.83)	28519 (98.8)	26821 (98.64)	23701 (98.61)	23777 (98.31)	19746 (98.07)	20784 (97.97)
**Oligohydramnios**	Yes	16762 (6)	996 (5.4)	1033 (4.96)	1190 (5.19)	1251 (5.51)	1496 (5.9)	1418 (5.85)	1651 (5.72)	1614 (5.94)	1663 (6.92)	1700 (7.03)	1383 (6.87)	1367 (6.44)
	No	263441 (94)	17455 (94.6)	19807 (95.04)	21751 (94.81)	21467 (94.49)	23880 (94.1)	22831 (94.15)	27215 (94.28)	25578 (94.06)	22372 (93.08)	22486 (92.97)	18752 (93.13)	19847 (93.56)

**PROM**	Yes	51527 (18.4)	3116 (16.89)	3851 (18.48)	4464 (19.46)	4018 (17.69)	4973 (19.6)	4616 (19.04)	5564 (19.28)	5145 (18.92)	4373 (18.19)	4395 (18.17)	3514 (17.45)	3498 (16.49)
	No	228676 (81.6)	15335 (83.11)	16989 (81.52)	18477 (80.54)	18700 (82.31)	20403 (80.4)	19633 (80.96)	23302 (80.72)	22047 (81.08)	19662 (81.81)	19791 (81.83)	16621 (82.55)	17716 (83.51)
**Placenta previa**	Yes	7942 (2.8)	587 (3.18)	618 (2.97)	676 (2.95)	714 (3.14)	743 (2.93)	806 (3.32)	955 (3.31)	738 (2.71)	665 (2.77)	534 (2.21)	441 (2.19)	465 (2.19)
	No	272261 (97.2)	17864 (96.82)	20222 (97.03)	22265 (97.05)	22004 (96.86)	24633 (97.07)	23443 (96.68)	27911 (96.69)	26454 (97.29)	23370 (97.23)	23652 (97.79)	19694 (97.81)	20749 (97.81)
**Placental abruption**	Yes	4228 (1.5)	223 (1.21)	232 (1.11)	261 (1.14)	231 (1.02)	318 (1.25)	327 (1.35)	494 (1.71)	494 (1.82)	406 (1.69)	485 (2.01)	407 (2.02)	350 (1.65)
	No	275975 (98.5)	18228 (98.79)	20608 (98.89)	22680 (98.86)	22487 (98.98)	25058 (98.75)	23922 (98.65)	28372 (98.29)	26698 (98.18)	23629 (98.31)	23701 (97.99)	19728 (97.98)	20864 (98.35)
**Cesarean section**	Yes	116883 (41.7)	7208 (39.07)	8438 (40.49)	8248 (35.95)	8319 (36.62)	10157 (40.03)	9931 (40.95)	12640 (43.79)	12304 (45.25)	11049 (45.97)	10575 (43.72)	8657 (42.99)	9357 (44.11)
	No	163320 (58.3)	11243 (60.93)	12402 (59.51)	14693 (64.05)	14399 (63.38)	15219 (59.97)	14318 (59.05)	16226 (56.21)	14888 (54.75)	12986 (54.03)	13611 (56.28)	11478 (57.01)	11857 (55.89)
**Perinatal asphyxia**	Yes	15942 (5.70)	1073 (5.82)	1191 (5.71)	1454 (6.34)	1314 (5.78)	1208 (4.76)	1279 (5.27)	1576 (5.46)	1463 (5.38)	1552 (6.46)	1572 (6.5)	1161 (5.77)	1099 (5.18)
	No	264261 (94.31)	17378 (94.18)	19649 (94.29)	21487 (93.66)	21404 (94.22)	24168 (95.24)	22970 (94.73)	27290 (94.54)	25729 (94.62)	22483 (93.54)	22614 (93.5)	18974 (94.23)	20115 (94.82)
**LGA**	Yes	15434 (5.5)	1131 (6.13)	1199 (5.75)	1402 (6.11)	1255 (5.52)	1378 (5.43)	1454 (6)	1650 (5.72)	1523 (5.6)	1254 (5.22)	1299 (5.37)	1019 (5.06)	870 (4.1)
	No	264769 (94.5)	17320 (93.87)	19641 (94.25)	21539 (93.89)	21463 (94.48)	23998 (94.57)	22795 (94)	27216 (94.28)	25669 (94.4)	22781 (94.78)	22887 (94.63)	19116 (94.94)	20344 (95.9)
**SGA**	Yes	10360 (3.70)	264 (1.43)	358 (1.72)	365 (1.59)	456 (2.01)	792 (3.12)	1331 (5.49)	1374 (4.76)	1465 (5.39)	1500 (6.24)	957 (3.96)	775 (3.85)	723 (3.41)
	No	269843 (96.30)	18187 (98.57)	20482 (98.28)	22576 (98.41)	22262 (97.99)	24584 (96.88)	22918 (94.51)	27492 (95.24)	25727 (94.61)	22535 (93.76)	23229 (96.04)	19360 (96.15)	20491 (96.59)
**Postpartum hemorrhage**	Yes	24374 (8.7)	1040 (5.64)	1298 (6.23)	1789 (7.8)	1959 (8.62)	2900 (11.43)	3084 (12.72)	3930 (13.61)	2179 (8.01)	1832 (7.62)	1622 (6.71)	1311 (6.51)	1430 (6.74)
	No	255829 (91.3)	17411 (94.36)	19542 (93.77)	21152 (92.2)	20759 (91.38)	22476 (88.57)	21165 (87.28)	24936 (86.39)	25013 (91.99)	22203 (92.38)	22564 (93.29)	18824 (93.49)	19784 (93.26)
**Premature**	Yes	30147 (10.8)	318 (1.72)	253 (1.21)	1752 (9.5)	2380 (11.42)	2505 (10.92)	2322 (10.22)	2872 (11.32)	2644 (10.9)	3312 (11.47)	3151 (11.59)	2963 (12.33)	1993 (9.39)
	No	250056 (89.2)	16699 (90.5)	18460 (88.58)	20436 (89.08)	20396 (89.78)	22504 (88.68)	21605 (89.1)	25554 (88.53)	24041 (88.41)	21072 (87.67)	21878 (90.46)	18190 (90.34)	19221 (90.61)
**Stillbirth**	Yes	1209 (0.4)	92 (0.5)	129 (0.62)	94 (0.41)	111 (0.49)	148 (0.58)	95 (0.39)	115 (0.4)	111 (0.41)	90 (0.37)	92 (0.38)	63 (0.31)	69 (0.33)
	No	278994 (99.6)	18359 (99.5)	20711 (99.38)	22847 (99.59)	22607 (99.51)	25228 (99.42)	24154 (99.61)	28751 (99.6)	27081 (99.59)	23945 (99.63)	24094 (99.62)	20072 (99.69)	21145 (99.67)
**Congenital malformation**	Yes	1820 (0.6)	89 (0.48)	107 (0.51)	83 (0.36)	110 (0.48)	121 (0.48)	104 (0.43)	125 (0.43)	148 (0.54)	130 (0.54)	220 (0.91)	255 (1.27)	328 (1.55)
	No	278383 (99.4)	18362 (99.52)	20733 (99.49)	22858 (99.64)	22608 (99.52)	25255 (99.52)	24145 (99.57)	28741 (99.57)	27044 (99.46)	23905 (99.46)	23966 (99.09)	19880 (98.73)	20886 (98.45)

ART, assisted reproductive technology; GDM, gestational diabetes mellitus; HDP, hypertensive disorders in pregnancy; PROM, premature rupture of membranes; LGA, Large-for-gestational-age; SGA, small-for-gestational-age.

Table S2 The full-range average annual percent changes (AAPC) and segment annual percent changes (APC) in obstetrics characteristics and adverse events, 2010-2021

Disease	AAPC (95%CI)	Segment 1		Segment 2		Segment 3	
		Year	APC (95% CI)	Year	APC (95% CI)	Year	APC (95% CI)
Advanced maternal age (>35 years)	5.023 (3.93–6.26) *	2010-2014	-3.22 (-10.18–0.48)	2014-2017	24.82 (17.86–29.87) *	2017-2021	0.14 (-3.17–2.51)
ART intervention	13.71 (7.83–19.91) *	2010-2021	13.71 (7.83–19.91) *				
GDM	16.25 (14.62–18.71) *	2010-2014	33.32 (25.6–47.58) *	2014-2021	7.49 (5.47–9.4) *		
HDP	-1 (-2.24–0.29)	2010-2015	[-7.11 (-13.54– (-3.85))] *	2015-2021	4.4 (1.68–9.61) *		
Hypothyroidism	96.39 (72.27–171.4) *	2010-2015	330.38 (218.29–789.83) *	2015-2021	2.14 (-3.5–7.17)		
Hyperthyroidism	44.97 (30.18–130.62) *	2010-2014	150.96 (75.86–5915.73) *	2014-2021	5.94 (-9.87–15.51)		
Anemia	10.7 (7.04–18.17) *	2010-2015	24.72 (15.85–65.46) *	2015-2021	0.23 (-8.94–4.92)		
Thrombocytopenia	13.81 (10.74–17.53) *	2010-2015	8.7 (-13.38–17.56)	2015-2019	27.84 (21.43–41.52) *	2019-2021	1.18 (-11.7–13.61)
Polyhydramnios	-0.99 (-3.1–1.25)	2010-2015	0.23 (-8.94–4.93)	2015-2021	10.83 (5.38–22) *		
Oligohydramnios	2.96 (1.39–4.72) *	2010-2021	2.96 (1.39–4.72) *				
PROM	-0.57 (-1.66–0.58)	2010-2016	1.5 (0.12–6.73) *	2016-2021	[-2.99 (-8.27– (-1.25))] *		
Placenta previa	-3.51 (-6.5–-0.82) *	2010-2016	0.83 (-2–13.77)	2016-2021	[-8.47 (-23.15– (-4.75))] *		
Placental abruption	6.15 (2.96–10.24) *	2010-2021	6.15 (2.96–10.24) *				
Cesarean section	0.79 (-0.06–1.6)	2010-2013	-2.37 (-8.11–1.4)	2013-2017	5.5 (3.44–9.16) *	2017-2021	-1.39 (-5.31–0.31)
Perinatal asphyxia	0.11 (-2.48–2.85)	2010-2021	0.11 (-2.48–2.85)				
LGA	[-3.11 (-5.17– (-0.4))] *	2010-2019	-1.13 (-3.15–10.98)	2019-2021	[-11.54 (-22.66– (-1.7))] *		
SGA	8.76 (2.86–17.1) *	2010-2017	25.13 (17.05–48.32) *	2017-2021	[-14.9 (-35.53– (-2.55))] *		
Postpartum hemorrhage	0.41 (-2.91–4)	2010-2015	19.76 (11.05–35.33) *	2015-2021	[-13.3 (-20.09– (-8.7))] *		
Premature	-1.35 (-3.14–0.48)	2010-2018	1.53 (0.06–5.97) *	2018-2021	[-8.65 (-20.09– (-2.63))] *		
Stillbirth	[-4.89 (-7.52– (-2.39))] *	2010-2021	[-4.89 (-7.52– (-2.39))] *				
Congenital malformation	11.02 (8.71–14.1) *	2010-2016	-2.95 (-11.57–3.45)	2016-2021	30.46 (23.59–44.05) *		

ART, assisted reproductive technology; GDM, gestational diabetes mellitus; HDP, hypertensive disorders in pregnancy; PROM, premature rupture of

membranes; LGA, Large-for-gestational-age; SGA, small-for-gestational-age.

The asterisk denotes a statistically significant difference.

The number of joinpoints were selected by the optimal models from joinpoint regression analysis.

Table S3 The selected ARIMA models and prediction of future obstetric events

Pregnancy complications and adverse perinatal outcomes	ARIMA (p, d, q)	AIC	P value of White-noise test	Year	Predicted incidence rate	75%CI	95%CI
**ART**	ARIMA (0, 1, 0)	23.44	0.797	2022	4.99	(4.17–5.8)	(3.6–6.37)
				2023	5.03	(3.89–6.17)	(3.09–6.98)
				2024	5.21	(3.61–6.81)	(2.48–7.94)
				2025	5.33	(3.27–7.4)	(1.82–8.85)
				2026	5.48	(2.9–8.06)	(1.09–9.88)
				2027	5.62	(2.49–8.74)	(0.29–10.94)
**GDM**	ARIMA (1, 2, 0)	39.07	0.203	2022	21.44	(19.84–23.05)	(18.71–24.18)
				2023	22.41	(19.01–25.8)	(16.63–28.19)
				2024	23.38	(17.82–28.93)	(13.9–32.85)
				2025	24.34	(16.3–32.38)	(10.64–38.04)
				2026	25.31	(14.5–36.12)	(6.89–43.73)
				2027	26.28	(12.44–40.11)	(2.71–49.85)
**HDP**	ARIMA (1, 0, 0)	22.71	0.649	2022	6.38	(5.68–7.08)	(5.19–7.57)
				2023	6.22	(4.87–7.57)	(3.92–8.53)
				2024	6.10	(3.93–8.27)	(2.4–9.8)
				2025	5.97	(2.87–9.07)	(0.68–11.25)
				2026	5.84	(1.7–9.97)	(-1.21–12.88)
				2027	5.71	(0.44–10.97)	(-3.26–14.68)
**Anemia**	ARIMA (1, 2, 1)	60.84	0.578	2022	26.83	(22.76–30.91)	(19.89–33.78)
				2023	27.97	(21.55–34.38)	(17.03–38.9)
				2024	29.11	(20.64–37.58)	(14.68–43.54)
				2025	30.26	(19.85–40.67)	(12.52–48)
				2026	31.41	(19.09–43.73)	(10.42–52.4)
				2027	32.56	(18.34–46.78)	(8.33–56.78)
**Thrombocytopenia**	ARIMA (1, 2, 1)	24.11	0.887	2022	4.69	(4.06–5.31)	(3.62–5.75)
				2023	5.00	(4.17–5.83)	(3.58–6.42)
				2024	5.31	(4.28–6.34)	(3.56–7.07)
				2025	5.63	(4.41–6.84)	(3.56–7.7)
				2026	5.94	(4.55–7.33)	(3.57–8.31)

				2027	6.25	(4.69–7.82)	(3.59–8.92)
**Polyhydramnios**	ARIMA (1, 2, 0)	5.15	0.685	2022	2.23	(2.02–2.45)	(1.87–2.6)
				2023	2.36	(2.02–2.71)	(1.77–2.95)
				2024	2.55	(1.98–3.11)	(1.59–3.51)
				2025	2.69	(1.91–3.46)	(1.37–4.01)
				2026	2.86	(1.83–3.89)	(1.1–4.62)
				2027	3.01	(1.72–4.31)	(0.8–5.22)
**Oligohydramnios**	ARIMA (0, 2, 0)	15.53	0.527	2022	6.02	(5.47–6.56)	(5.08–6.95)
				2023	5.59	(4.38–6.8)	(3.53–7.66)
				2024	5.17	(3.15–7.19)	(1.72–8.61)
				2025	4.74	(1.79–7.7)	(-0.29–9.78)
				2026	4.32	(0.33–8.31)	(-2.48–11.12)
				2027	3.90	(-1.24–9.03)	(-4.85–12.64)
**PROM**	ARIMA (0, 0, 0)	41.29	0.070	2022	15.68	(14.24–17.13)	(13.22–18.14)
				2023	14.78	(12.34–17.22)	(10.62–18.94)
				2024	13.94	(10.01–17.86)	(7.25–20.63)
				2025	13.05	(7.58–18.53)	(3.73–22.38)
				2026	12.20	(4.92–19.47)	(-0.19–24.59)
				2027	11.32	(2.14–20.51)	(-4.33–26.97)
**Placenta previa**	ARIMA (0, 2, 0)	15.30	0.111	2022	2.18	(1.76–2.6)	(1.47–2.89)
				2023	2.17	(1.45–2.89)	(0.95–3.4)
				2024	2.16	(1.01–3.32)	(0.19–4.13)
				2025	2.16	(0.54–3.78)	(-0.6–4.92)
				2026	2.15	(0–4.3)	(-1.52–5.82)
				2027	2.14	(-0.58–4.86)	(-2.49–6.78)
**Placental abruption**	ARIMA (0, 2, 0)	5.24	0.563	2022	1.74	(1.48–1.99)	(1.3–2.17)
				2023	1.70	(1.29–2.11)	(1–2.4)
				2024	1.49	(0.93–2.06)	(0.53–2.45)
				2025	1.48	(0.69–2.28)	(0.13–2.83)
				2026	1.42	(0.4–2.45)	(-0.32–3.17)
				2027	1.29	(0.03–2.56)	(-0.86–3.45)
**Cesarean**	ARIMA (0, 2, 0)	53.11	0.545	2022	44.67	(41.95–47.39)	(40.04–49.3)
				2023	45.16	(40.91–49.41)	(37.92–52.4)
				2024	45.64	(40.11–51.17)	(36.21–55.06)
				2025	46.12	(39.43–52.81)	(34.72–57.51)

				2026	46.60	(38.82–54.37)	(33.35–59.84)
				2027	47.08	(38.26–55.89)	(32.05–62.1)
**Perinatal asphyxia**	ARIMA (0, 2, 0)	27.81	0.070	2022	5.02	(4.29–5.74)	(3.78–6.25)
				2023	4.59	(3.23–5.95)	(2.28–6.9)
				2024	4.00	(2.17–5.83)	(0.88–7.12)
				2025	3.65	(1.18–6.12)	(-0.56–7.86)
				2026	3.30	(0.03–6.57)	(-2.28–8.88)
				2027	2.80	(-1.26–6.86)	(-4.12–9.71)
**LGA**	ARIMA (0, 2, 0)	20.88	0.394	2022	3.99	(3.49–4.48)	(3.15–4.82)
				2023	3.59	(2.84–4.35)	(2.31–4.88)
				2024	2.88	(1.86–3.89)	(1.15–4.6)
				2025	2.61	(1.17–4.06)	(0.16–5.07)
				2026	2.18	(0.33–4.03)	(-0.97–5.33)
				2027	1.59	(-0.67–3.86)	(-2.26–5.45)
**SGA**	ARIMA (1, 2, 1)	39.87	0.791	2022	3.22	(1.77–4.67)	(0.75–5.68)
				2023	3.00	(0.76–5.24)	(-0.82–6.82)
				2024	2.79	(-0.27–5.84)	(-2.42–7.99)
				2025	2.57	(-1.33–6.48)	(-4.08–9.23)
				2026	2.36	(-2.44–7.17)	(-5.82–10.55)
				2027	2.15	(-3.6–7.89)	(-7.64–11.94)
**Postpartum hemorrhage**	ARIMA (1, 2, 0)	50.35	0.745	2022	6.77	(3.98–9.57)	(2.01–11.53)
				2023	6.90	(1.76–12.03)	(-1.85–15.65)
				2024	6.98	(-1.23–15.19)	(-7.01–20.97)
				2025	7.08	(-4.55–18.71)	(-12.74–26.9)
				2026	7.17	(-8.3–22.64)	(-19.19–33.53)
				2027	7.27	(-12.37–26.91)	(-26.19–40.73)
**Premature**	ARIMA (0, 1, 0)	55.62	0.926	2022	8.88	(5.16–12.61)	(2.54–15.23)
				2023	6.77	(0.54–12.99)	(-3.84–17.37)
				2024	5.72	(-4.32–15.76)	(-11.39–22.82)
				2025	3.96	(-9.99–17.91)	(-19.81–27.73)
				2026	2.67	(-15.87–21.22)	(-28.92–34.27)
				2027	1.07	(-22.32–24.47)	(-38.79–40.94)
**Stillbirth**	ARIMA (0, 1, 0)	16.83	0.073	2022	0.29	(0.01–0.57)	(0.13–0.46)
				2023	0.29	(-0.18–0.76)	(0.01–0.57)

				2024	0.26	(-0.49–1.02)	(-0.18–0.71)
				2025	0.26	(-0.8–1.31)	(-0.36–0.87)
				2026	0.24	(-1.17–1.64)	(-0.59–1.06)
				2027	0.22	(-1.55–1.99)	(-0.82–1.26)
**Congenital malformation**	ARIMA (0, 2, 0)	4.95	0.416	2022	1.83	(1.63–2.03)	(1.49–2.17)
				2023	2.11	(1.67–2.55)	(1.36–2.86)
				2024	2.39	(1.65–3.13)	(1.14–3.64)
				2025	2.67	(1.59–3.75)	(0.83–4.51)
				2026	2.95	(1.49–4.41)	(0.46–5.44)
				2027	3.23	(1.35–5.11)	(0.03–6.43)

ART, assisted reproductive technology; GDM, gestational diabetes mellitus; HDP, hypertensive disorders in pregnancy; PROM, premature rupture of membranes; LGA, Large-for-gestational-age; SGA, small-for-gestational-age.

Table S4 Sensitivity analysis of the selected ARIMA models and prediction of future obstetric events using data from 2010-2020

Pregnancy complications and adverse perinatal outcomes	ARIMA (p, d, q)	AIC	P value of White-noise test	Year	Predicted incidence rate	75%CI	95%CI
ART	ARIMA (1, 1, 0)	25.18	0.179	2021	4.59	(3.68–5.49)	(3.05–6.12)
				2022	4.58	(3.25–5.91)	(2.32–6.85)
				2023	4.58	(2.93–6.24)	(1.77–7.4)
				2024	4.58	(2.66–6.51)	(1.31–7.86)
				2025	4.58	(2.42–6.74)	(0.9–8.26)
				2026	4.58	(2.21–6.96)	(0.54–8.63)
GDM	ARIMA (1, 2, 0)	35.53	0.921	2021	21.95	(20.35–23.56)	(19.22–24.68)
				2022	24.23	(20.69–27.78)	(18.19–30.28)
				2023	26.52	(20.61–32.42)	(16.45–36.58)
				2024	28.80	(20.17–37.42)	(14.1–43.5)
				2025	31.08	(19.42–42.74)	(11.21–50.95)
				2026	33.36	(18.38–48.35)	(7.83–58.89)
HDP	ARIMA (1, 0, 0)	22.01	0.199	2021	6.73	(6.17–7.28)	(5.78–7.67)
				2022	6.67	(5.97–7.37)	(5.48–7.86)
				2023	6.62	(5.85–7.4)	(5.3–7.94)
				2024	6.59	(5.77–7.41)	(5.2–7.98)
				2025	6.56	(5.72–7.4)	(5.13–8)
				2026	6.54	(5.69–7.4)	(5.09–8)
Anemia	ARIMA (1, 2, 0)	55.99	0.351	2021	26.35	(21.17–31.53)	(17.52–35.18)
				2022	27.00	(16.98–37.02)	(9.93–44.07)
				2023	27.35	(11.26–43.44)	(-0.06–54.76)
				2024	27.81	(4.84–50.77)	(-11.32–66.93)
				2025	28.23	(-2.39–58.85)	(-23.94–80.4)
				2026	28.66	(-10.31–67.62)	(-37.73–95.05)
Thrombocytopenia	ARIMA (1, 1, 0)	23.05	0.213	2021	4.04	(3.32–4.76)	(2.81–5.27)
				2022	4.04	(2.99–5.09)	(2.25–5.83)
				2023	4.04	(2.74–5.34)	(1.82–6.26)
				2024	4.04	(2.53–5.55)	(1.46–6.61)
				2025	4.04	(2.34–5.73)	(1.15–6.93)
				2026	4.04	(2.18–5.9)	(0.87–7.21)

Polyhydramnios	ARIMA (1, 2, 1)	1.99	0.477	2021	2.26	(2.05–2.47)	(1.9–2.62)
				2022	2.51	(2.15–2.87)	(1.89–3.13)
				2023	2.83	(2.22–3.44)	(1.79–3.87)
				2024	3.09	(2.24–3.94)	(1.65–4.53)
				2025	3.40	(2.26–4.55)	(1.45–5.36)
				2026	3.67	(2.23–5.11)	(1.21–6.13)
Oligohydramnios	ARIMA (0, 2, 0)	14.83	0.813	2021	6.71	(6.14–7.28)	(5.76–7.69)
				2022	6.55	(5.28–7.82)	(4.48–8.69)
				2023	6.39	(4.27–8.51)	(2.95–9.93)
				2024	6.23	(3.12–9.34)	(1.21–11.37)
				2025	6.07	(1.86–10.28)	(-0.7–13)
				2026	5.91	(0.49–11.33)	(-2.79–14.8)
PROM	ARIMA (1, 1, 0)	32.09	0.906	2021	17.78	(16.66–18.9)	(15.87–19.69)
				2022	17.63	(16.35–18.9)	(15.46–19.8)
				2023	17.70	(16.17–19.23)	(15.09–20.3)
				2024	17.67	(15.97–19.36)	(14.78–20.56)
				2025	17.68	(15.81–19.55)	(14.5–20.86)
				2026	17.67	(15.66–19.69)	(14.23–21.11)
Placenta previa	ARIMA (1, 2, 0)	11.73	0.165	2021	1.81	(1.4–2.22)	(1.1–2.51)
				2022	1.67	(0.98–2.36)	(0.5–2.84)
				2023	1.37	(0.26–2.48)	(-0.53–3.26)
				2024	1.18	(-0.37–2.72)	(-1.46–3.81)
				2025	0.91	(-1.14–2.96)	(-2.59–4.41)
				2026	0.69	(-1.9–3.28)	(-3.72–5.11)
Placental abruption	ARIMA (1, 2, 0)	1.45	0.297	2021	2.23	(2–2.47)	(1.83–2.63)
				2022	2.31	(1.92–2.71)	(1.64–2.98)
				2023	2.48	(1.84–3.11)	(1.39–3.56)
				2024	2.59	(1.7–3.47)	(1.08–4.09)
				2025	2.73	(1.56–3.91)	(0.73–4.73)
				2026	2.86	(1.37–4.34)	(0.33–5.38)
Cesarean	ARIMA (0, 1, 0)	46.84	0.879	2021	42.99	(40.37–45.61)	(38.53–47.45)
				2022	42.99	(39.29–46.69)	(36.68–49.3)
				2023	42.99	(38.45–47.53)	(35.26–50.72)
				2024	42.99	(37.75–48.23)	(34.06–51.92)
				2025	42.99	(37.13–48.85)	(33.01–52.97)

				2026	42.99	(36.57–49.41)	(32.06–53.92)
Perinatal asphyxia	ARIMA (0, 2, 0)	21.14	0.604	2021	5.76	(5.21–6.31)	(4.82–6.7)
				2022	5.76	(5.18–6.33)	(4.77–6.74)
				2023	5.75	(5.17–6.33)	(4.77–6.74)
				2024	5.75	(5.17–6.33)	(4.76–6.74)
				2025	5.75	(5.17–6.33)	(4.76–6.74)
				2026	5.75	(5.17–6.33)	(4.76–6.74)
LGA	ARIMA (1,1,0)	14.32	0.647	2021	5.40	(5.04–5.77)	(4.78–6.03)
				2022	5.54	(5.15–5.93)	(4.87–6.21)
				2023	5.59	(5.19–5.99)	(4.92–6.27)
				2024	5.61	(5.21–6.01)	(4.93–6.29)
				2025	5.62	(5.22–6.02)	(4.94–6.3)
				2026	5.62	(5.23–6.02)	(4.95–6.3)
SGA	ARIMA (1, 1,0)	35.73	0.847	2021	3.85	(2.49–5.21)	(1.53–6.17)
				2022	3.85	(1.95–5.75)	(0.61–7.09)
				2023	3.85	(1.53–6.17)	(-0.1–7.81)
				2024	3.85	(1.18–6.53)	(-0.71–8.41)
				2025	3.85	(0.86–6.84)	(-1.24–8.94)
				2026	3.85	(0.58–7.12)	(-1.72–9.43)
Postpartum hemorrhage	ARIMA (1, 2, 0)	46.60	0.800	2021	5.98	(3.05–8.91)	(0.99–10.97)
				2022	5.61	(0.23–10.99)	(-3.56–14.77)
				2023	5.16	(-3.44–13.77)	(-9.5–19.82)
				2024	4.75	(-7.44–16.94)	(-16.02–25.52)
				2025	4.32	(-11.89–20.53)	(-23.29–31.93)
				2026	3.90	(-16.67–24.47)	(-31.15–38.95)
Premature	ARIMA (0, 1, 0)	50.62	0.968	2021	12.33	(9.17–15.49)	(6.94–17.72)
				2022	12.33	(7.85–16.81)	(4.7–19.96)
				2023	12.33	(6.85–17.81)	(2.99–21.67)
				2024	12.33	(6–18.66)	(1.55–23.11)
				2025	12.33	(5.25–19.41)	(0.27–24.39)
				2026	12.33	(4.58–20.08)	(-0.88–5.54)
Stillbirth	ARIMA (0, 1, 0)	-14.20	0.053	2021	0.31	(0.19–0.43)	(0.1–0.52)
				2022	0.31	(0.13–0.49)	(0.01–0.61)
				2023	0.31	(0.1–0.52)	(-0.06–0.68)
				2024	0.31	(0.06–0.56)	(-0.11–0.73)

				2025	0.31	(0.03–0.59)	(-0.16–0.78)
				2026	0.31	(0.01–0.61)	(-0.21–0.83)
Congenital malformation	ARIMA (0, 2, 0)	-3.50	0.056	2021	1.63	(1.42–1.84)	(1.28–1.98)
				2022	1.99	(1.53–2.45)	(1.21–2.77)
				2023	2.35	(1.58–3.12)	(1.04–3.66)
				2024	2.71	(1.59–3.83)	(0.8–4.62)
				2025	3.07	(1.55–4.59)	(0.48–5.66)
				2026	3.43	(1.47–5.39)	(0.1–6.76)

ART, assisted reproductive technology; GDM, gestational diabetes mellitus; HDP, hypertensive disorders in pregnancy; PROM, premature rupture of membranes; LGA, Large-for-gestational-age; SGA, small-for-gestational-age.

Table S5 The characteristics and adverse obstetric events of pregnant women in different age group (years)

Characteristics	<20 (n=7,036)	20-24 (n=46,802)	25-29 (n=119,964)	30-34 (n=73,167)	35-39 (n=27,821)	≥40 (n=5,409)	*P* trend
ART intervention	/	0.34 (0.29–0.39)	2.17 (2.09–2.25)	6.38 (6.2–6.56)	6.21 (5.93–6.5)	6.21 (5.57–6.86)	<0.05
HBV infection	0.75 (0.55–0.96)	1.21 (1.11–1.3)	1.47 (1.41–1.54)	1.62 (1.53–1.71)	1.39 (1.26–1.53)	1.89 (1.52–2.25)	<0.05
Twin or multiple gestations	4.39 (3.91–4.87)	4.42 (4.23–4.6)	5.58 (5.45–5.71)	8.47 (8.27–8.67)	7.42 (7.11–7.72)	6.16 (5.52–6.8)	<0.05
Hypothyroidism	5.24 (4.72–5.77)	6.32 (6.1–6.54)	8.58 (8.42–8.74)	11 (10.78–11.23)	11.56 (11.19–11.94)	11.96 (11.1–12.83)	<0.05
Hyperthyroidism	0.14 (0.05–0.23)	0.28 (0.23–0.32)	0.41 (0.38–0.45)	0.45 (0.4–0.5)	0.41 (0.33–0.48)	0.24 (0.11–0.37)	<0.05
GDM	3.08 (2.68–3.49)	5.81 (5.59–6.02)	10.92 (10.75–11.1)	17.35 (17.08–17.63)	24 (23.5–24.5)	28.8 (27.6–30.01)	<0.05
HDP	6.21 (5.65–6.78)	5.01 (4.81–5.21)	4.89 (4.77–5.01)	6.65 (6.47–6.83)	9.69 (9.34–10.03)	15.25 (14.29–16.21)	<0.05
Anemia	26.01 (24.98–27.03)	22.93 (22.55–23.31)	20.21 (19.98–20.43)	21.87 (21.57–22.17)	23.16 (22.66–23.65)	25.7 (24.53–26.86)	<0.05
Thrombocytopenia	1.12 (0.88–1.37)	1.46 (1.35–1.57)	1.85 (1.78–1.93)	2.57 (2.46–2.69)	2.72 (2.53–2.92)	2.5 (2.08–2.91)	<0.05
Polyhydramnios	0.81 (0.6–1.02)	1.02 (0.93–1.11)	1.39 (1.32–1.45)	1.96 (1.86–2.06)	2.24 (2.07–2.41)	3.36 (2.88–3.85)	<0.05
Oligohydramnios	6.55 (5.97–7.13)	6.48 (6.26–6.71)	6.14 (6–6.27)	5.56 (5.39–5.73)	5.51 (5.24–5.78)	5.56 (4.95–6.18)	<0.05
PROM	16.17 (15.31–17.03)	18.17 (17.82–18.52)	19.51 (19.28–19.73)	17.46 (17.18–17.73)	17.16 (16.71–17.6)	17.3 (16.3–18.31)	<0.05
Placenta previa	0.99 (0.76–1.23)	1.39 (1.28–1.49)	2.18 (2.1–2.26)	3.62 (3.49–3.76)	5.59 (5.32–5.86)	7.47 (6.77–8.17)	<0.05
Placental abruption	2.05 (1.72–2.38)	1.36 (1.26–1.47)	1.31 (1.25–1.38)	1.64 (1.55–1.74)	1.97 (1.81–2.14)	2.22 (1.83–2.61)	<0.05
Cesarean section	20.24 (19.3–21.18)	28.1 (27.69–28.51)	37.06 (36.79–37.34)	51.37 (51.01–51.73)	60.98 (60.4–61.55)	60.88 (59.58–62.18)	<0.05
Postpartum hemorrhage	6.11 (5.55–6.67)	6.58 (6.36–6.81)	7.93 (7.78–8.08)	10.1 (9.88–10.32)	11.77 (11.39–12.15)	12.68 (11.8–13.57)	<0.05
LGA	3.28 (2.87–3.7)	4.93 (4.73–5.12)	5.7 (5.57–5.83)	5.74 (5.57–5.91)	5.59 (5.32–5.86)	5.69 (5.08–6.31)	<0.05
SGA	0.55 (0.38–0.73)	0.38 (0.33–0.44)	0.33 (0.3–0.36)	0.36 (0.32–0.4)	0.42 (0.35–0.5)	0.31 (0.17–0.46)	<0.05
Premature	11.98 (11.22–12.74)	9.16 (8.9–9.43)	9.31 (9.15–9.48)	12.27 (12.04–12.51)	14.36 (13.95–14.78)	16.01 (15.03–16.99)	<0.05
Perinatal asphyxia	7.18 (6.57–7.78)	6.13 (5.91–6.35)	5.92 (5.79–6.05)	4.77 (4.61–4.92)	4.45 (4.21–4.7)	5.47 (4.87–6.08)	<0.05
Congenital malformation	0.74 (0.54–0.94)	0.63 (0.56–0.71)	0.59 (0.55–0.63)	0.66 (0.6–0.72)	0.79 (0.69–0.89)	1.13 (0.85–1.41)	<0.05
Stillbirth	0.72 (0.53–0.92)	0.48 (0.42–0.54)	0.37 (0.33–0.4)	0.44 (0.39–0.49)	0.51 (0.43–0.6)	0.57 (0.37–0.77)	<0.05

The data were presented as prevalence (95% CI).

ART, assisted reproductive technology; HBV, hepatitis B virus; GDM, gestational diabetes mellitus; HDP, hypertensive disorders in pregnancy; PROM,

premature rupture of membranes; LGA, Large-for-gestational-age; SGA, small-for-gestational-age.

Table S6 The risk of pregnancy complications and adverse perinatal outcomes in different age groups

**Disease**	Age group	PRR (95%CI)	*P*
**Pregnancy complications**			
Hypothyroidism	20-24	Reference	
	<20	0.83 (0.72–0.94)	<0.001
	25-29	1.36 (1.32–1.4)	<0.001
	30-34	1.74 (1.7–1.78)	<0.001
	35-39	1.83 (1.78–1.88)	<0.001
	⩾40	1.89 (1.81–1.98)	<0.001
Hyperthyroidism	20-24	Reference	
	<20	0.52 (-0.13–1.16)	0.044
	25-29	1.5 (1.3–1.69)	<0.001
	30-34	1.64 (1.43–1.84)	<0.001
	35-39	1.47 (1.22–1.73)	0.003
	⩾40	0.87 (0.3–1.44)	0.638
GDM	20-24	Reference	
	<20	0.53 (0.39–0.67)	<0.001
	25-29	1.88 (1.84–1.92)	<0.001
	30-34	2.99 (2.95–3.03)	<0.001
	35-39	4.13 (4.09–4.18)	<0.001
	⩾40	4.96 (4.9–5.02)	<0.001
HDP	20-24	Reference	
	<20	1.24 (1.14–1.34)	<0.001
	25-29	0.98 (0.93–1.02)	0.337
	30-34	1.33 (1.28–1.38)	<0.001

	35-39	1.93 (1.88–1.99)	<0.001
	⩾40	3.05 (2.97–3.12)	<0.001
Anemia	20-24	Reference	
	<20	1.13 (1.08–1.18)	<0.001
	25-29	0.88 (0.86–0.9)	<0.001
	30-34	0.95 (0.93–0.98)	<0.001
	35-39	1.01 (0.98–1.04)	0.529
	⩾40	1.12 (1.07–1.18)	<0.001
Thrombocytopenia	20-24	Reference	
	<20	0.77 (0.54–1)	0.027
	25-29	1.27 (1.18–1.35)	<0.001
	30-34	1.76 (1.67–1.85)	<0.001
	35-39	1.86 (1.76–1.97)	<0.001
	⩾40	1.71 (1.52–1.89)	<0.001
**Adverse perinatal outcomes**			
Polyhydramnios	20-24	Reference	
	<20	0.79 (0.52–1.07)	0.095 .
	25-29	1.36 (1.25–1.46)	<0.001
	30-34	1.92 (1.81–2.02)	<0.001
	35-39	2.19 (2.07–2.31)	<0.001
	⩾40	3.29 (3.12–3.46)	<0.001
Oligohydramnios	20-24	Reference	
	<20	1.01 (0.91–1.11)	0.831
	25-29	0.95 (0.9–0.99)	0.012
	30-34	0.86 (0.81–0.9)	<0.001

	35-39	0.85 (0.79–0.91)	<0.001
	⩾40	0.86 (0.74–0.98)	0.012
PROM	20-24	Reference	
	<20	0.89 (0.83–0.95)	<0.001
	25-29	1.07 (1.05–1.1)	<0.001
	30-34	0.96 (0.93–0.99)	0.004
	35-39	0.94 (0.91–0.98)	0.001
	⩾40	0.95 (0.88–1.02)	0.156
Placenta previa	20-24	Reference	
	<20	0.72 (0.47–0.96)	0.008
	25-29	1.57 (1.49–1.66)	<0.001
	30-34	2.61 (2.53–2.7)	<0.001
	35-39	4.03 (3.94–4.12)	<0.001
	⩾40	5.39 (5.26–5.51)	<0.001
Placental abruption	20-24	Reference	
	<20	1.5 (1.32–1.68)	<0.001
	25-29	0.96 (0.87–1.05)	0.415
	30-34	1.21 (1.11–1.3)	<0.001
	35-39	1.45 (1.33–1.56)	<0.001
	⩾40	1.63 (1.43–1.82)	<0.001
Cesarean section	20-24	Reference	
	<20	0.72 (0.67–0.77)	<0.001
	25-29	1.32 (1.3–1.34)	<0.001
	30-34	1.83 (1.81–1.85)	<0.001
	35-39	2.17 (2.15–2.19)	<0.001

	⩾40	2.17 (2.13–2.2)	<0.001
Postpartum hemorrhage	20-24	Reference	
	<20	0.93 (0.83–1.03)	0.149
	25-29	1.2 (1.16–1.25)	<0.001
	30-34	1.53 (1.49–1.58)	<0.001
	35-39	1.79 (1.74–1.84)	<0.001
	⩾40	1.93 (1.84–2.01)	<0.001
Perinatal asphyxia	20-24	Reference	
	<20	1.17 (1.08–1.27)	0.001
	25-29	0.97 (0.92–1.01)	0.111
	30-34	0.78 (0.73–0.83)	<0.001
	35-39	0.73 (0.66–0.79)	<0.001
	⩾40	0.89 (0.77–1.01)	0.062
LGA	20-24	Reference	
	<20	0.67 (0.53–0.8)	<0.001
	25-29	1.16 (1.11–1.2)	<0.001
	30-34	1.17 (1.11–1.22)	<0.001
	35-39	1.13 (1.07–1.2)	<0.001
	⩾40	1.16 (1.04–1.27)	0.017
SGA	20-24	Reference	
	<20	1.37 (1.23–1.51)	<0.001
	25-29	1.12 (1.05–1.19)	0.001
	30-34	1.48 (1.41–1.55)	<0.001
	35-39	1.62 (1.54–1.71)	<0.001
	⩾40	1.88 (1.74–2.02)	<0.001

Premature	20-24	Reference	
	<20	1.31 (1.23–1.38)	<0.001
	25-29	1.02 (0.98–1.05)	0.371
	30-34	1.34 (1.3–1.38)	<0.001
	35-39	1.57 (1.52–1.61)	<0.001
	⩾40	1.75 (1.67–1.82)	<0.001
Stillbirth	20-24	Reference	
	<20	1.51 (1.2–1.81)	0.008
	25-29	0.76 (0.6–0.92)	0.001
	30-34	0.91 (0.74–1.08)	0.277
	35-39	1.07 (0.86–1.28)	0.532
	⩾40	1.19 (0.82–1.57)	0.359
Congenital malformation	20-24	Reference	
	<20	1.16 (0.87–1.46)	0.311
	25-29	0.93 (0.79–1.07)	0.294
	30-34	1.04 (0.89–1.18)	0.612
	35-39	1.25 (1.07–1.42)	0.013
	⩾40	1.78 (1.5–2.05)	<0.001

PRR, prevalence rate ratio; GDM, gestational diabetes mellitus; HDP, hypertensive disorders in pregnancy; PROM, premature rupture of membranes; LGA, Large-for-gestational-age; SGA, small-for-gestational-age.

The Poisson regression models were used to examine the association between maternal age and pregnancy complications as well as adverse perinatal outcomes.

Table S7 The risk of different maternal age (15-49 years) for pregnancy complications, adverse perinatal outcomes and ART interventions

Maternal age	Hypothyroidism	Hyperthyroidism	GDM	HDP	Anemia	Thrombocytopenia	Polyhydramnios	Oligohydramnios	Premature rupture of membranes	Placenta previa	Placental abruption
	OR(95% CI)	OR(95% CI)	OR(95% CI)	OR(95% CI)	OR(95% CI)	OR(95% CI)	OR(95% CI)	OR(95% CI)	OR(95% CI)	OR(95% CI)	OR(95% CI)
15.00	0.45 (0.41–0.51)	0.25 (0.14–0.45)	0.13 (0.12–0.15)	1.4 (1.24–1.57)	1.76 (1.65–1.87)	0.4 (0.31–0.5）	0.47 (0.36–0.62)	1.08 (0.96–1.2)	0.69 (0.64–0.74)	0.31 (0.25–0.4)	1.8 (1.46–2.22)
15.17	0.46 (0.41–0.51)	0.26 (0.15–0.45)	0.13 (0.12–0.15)	1.38 (1.23–1.55)	1.74 (1.64–1.85)	0.4 (0.32–0.5）	0.47 (0.36–0.62)	1.08 (0.96–1.2)	0.69 (0.64–0.74)	0.32 (0.25–0.4)	1.78 (1.44–2.19)
15.34	0.46 (0.41–0.52)	0.26 (0.15–0.46)	0.14 (0.12–0.16)	1.37 (1.23–1.54)	1.72 (1.62–1.83)	0.4 (0.32–0.51）	0.48 (0.37–0.62)	1.08 (0.97–1.2)	0.7 (0.65–0.75)	0.32 (0.26–0.41)	1.76 (1.43–2.15)
15.51	0.47 (0.42–0.52)	0.27 (0.15–0.46)	0.14 (0.13–0.16)	1.36 (1.22–1.52)	1.71 (1.61–1.81)	0.41 (0.33–0.51）	0.48 (0.37–0.63)	1.08 (0.97–1.2)	0.7 (0.65–0.75)	0.33 (0.26–0.41)	1.74 (1.42–2.12)
15.68	0.47 (0.42–0.53)	0.27 (0.16–0.47)	0.15 (0.13–0.17)	1.35 (1.21–1.51)	1.69 (1.59–1.79)	0.41 (0.33–0.51）	0.49 (0.38–0.63)	1.08 (0.97–1.19)	0.71 (0.66–0.76)	0.33 (0.26–0.42)	1.71 (1.41–2.09)
15.85	0.48 (0.43–0.53)	0.28 (0.16–0.47)	0.15 (0.13–0.17)	1.34 (1.21–1.5)	1.67 (1.58–1.77)	0.42 (0.34–0.52）	0.49 (0.38–0.63)	1.08 (0.97–1.19)	0.71 (0.67–0.76)	0.34 (0.27–0.42)	1.69 (1.4–2.05)
16.03	0.48 (0.43–0.53)	0.28 (0.17–0.48)	0.15 (0.14–0.17)	1.33 (1.2–1.48)	1.66 (1.57–1.75)	0.42 (0.34–0.52）	0.5 (0.39–0.64)	1.08 (0.97–1.19)	0.72 (0.67–0.76)	0.34 (0.27–0.42)	1.67 (1.39–2.02)
16.20	0.49 (0.44–0.54)	0.29 (0.17–0.48)	0.16 (0.14–0.18)	1.32 (1.19–1.47)	1.64 (1.55–1.73)	0.43 (0.35–0.52）	0.5 (0.39–0.64)	1.08 (0.98–1.19)	0.72 (0.68–0.77)	0.35 (0.28–0.43)	1.65 (1.37–1.99)
16.37	0.49 (0.44–0.54)	0.3 (0.18–0.49)	0.16 (0.15–0.18)	1.31 (1.19–1.45)	1.62 (1.54–1.71)	0.43 (0.35–0.53）	0.51 (0.4–0.64)	1.08 (0.98–1.18)	0.73 (0.68–0.77)	0.35 (0.28–0.43)	1.64 (1.36–1.96)
16.54	0.49 (0.45–0.55)	0.3 (0.19–0.49)	0.17 (0.15–0.19)	1.3 (1.18–1.44)	1.61 (1.52–1.7)	0.44 (0.36–0.53）	0.51 (0.4–0.64)	1.08 (0.98–1.18)	0.73 (0.69–0.78)	0.35 (0.29–0.44)	1.62 (1.35–1.93)
16.71	0.5 (0.45–0.55)	0.31 (0.19–0.5)	0.17 (0.16–0.19)	1.29 (1.17–1.42)	1.59 (1.51–1.68)	0.44 (0.36–0.53）	0.51 (0.41–0.65)	1.08 (0.98–1.18)	0.74 (0.7–0.78)	0.36 (0.29–0.44)	1.6 (1.34–1.9)
16.88	0.5 (0.46–0.55)	0.31 (0.2–0.5)	0.18 (0.16–0.2)	1.28 (1.17–1.41)	1.58 (1.5–1.66)	0.45 (0.37–0.54）	0.52 (0.42–0.65)	1.08 (0.98–1.18)	0.74 (0.7–0.79)	0.36 (0.3–0.44)	1.58 (1.33–1.87)
17.05	0.51 (0.46–0.56)	0.32 (0.2–0.51)	0.18 (0.16–0.2)	1.27 (1.16–1.4)	1.56 (1.49–1.64)	0.45 (0.37–0.54）	0.52 (0.42–0.65)	1.08 (0.99–1.18)	0.75 (0.71–0.79)	0.37 (0.3–0.45)	1.56 (1.32–1.84)
17.22	0.51 (0.47–0.56)	0.33 (0.21–0.51)	0.19 (0.17–0.21)	1.26 (1.15–1.38)	1.55 (1.47–1.62)	0.46 (0.38–0.55）	0.53 (0.43–0.66)	1.08 (0.99–1.17)	0.76 (0.71–0.8)	0.37 (0.31–0.45)	1.54 (1.31–1.81)
17.39	0.52 (0.48–0.57)	0.33 (0.22–0.52)	0.19 (0.17–0.21)	1.25 (1.15–1.37)	1.53 (1.46–1.61)	0.46 (0.39–0.55）	0.53 (0.43–0.66)	1.08 (0.99–1.17)	0.76 (0.72–0.8)	0.38 (0.32–0.46)	1.52 (1.3–1.79)
17.56	0.52 (0.48–0.57)	0.34 (0.22–0.53)	0.2 (0.18–0.22)	1.24 (1.14–1.36)	1.52 (1.45–1.59)	0.47 (0.39–0.55）	0.54 (0.44–0.66)	1.08 (0.99–1.17)	0.77 (0.73–0.81)	0.38 (0.32–0.46)	1.5 (1.29–1.76)
17.73	0.53 (0.49–0.57)	0.35 (0.23–0.53)	0.2 (0.19–0.22)	1.23 (1.13–1.34)	1.5 (1.43–1.57)	0.47 (0.4–0.56）	0.54 (0.45–0.66)	1.08 (0.99–1.17)	0.77 (0.73–0.81)	0.39 (0.33–0.47)	1.49 (1.28–1.73)
17.90	0.53 (0.49–0.58)	0.36 (0.24–0.54)	0.21 (0.19–0.23)	1.23 (1.13–1.33)	1.49 (1.42–1.55)	0.48 (0.4–0.56）	0.55 (0.45–0.67)	1.08 (1–1.16)	0.78 (0.74–0.82)	0.4 (0.33–0.47)	1.47 (1.27–1.7)
18.08	0.54 (0.5–0.58)	0.36 (0.24–0.54)	0.22 (0.2–0.24)	1.22 (1.12–1.32)	1.47 (1.41–1.54)	0.48 (0.41–0.56）	0.55 (0.46–0.67)	1.08 (1–1.16)	0.79 (0.75–0.83)	0.4 (0.34–0.47)	1.45 (1.26–1.68)
18.25	0.54 (0.5–0.59)	0.37 (0.25–0.55)	0.22 (0.2–0.24)	1.21 (1.12–1.31)	1.46 (1.4–1.52)	0.49 (0.42–0.57）	0.56 (0.46–0.67)	1.08 (1–1.16)	0.79 (0.75–0.83)	0.41 (0.34–0.48)	1.44 (1.25–1.65)
18.42	0.55 (0.51–0.59)	0.38 (0.26–0.55)	0.23 (0.21–0.25)	1.2 (1.11–1.29)	1.44 (1.38–1.5)	0.49 (0.42–0.57）	0.56 (0.47–0.68)	1.08 (1–1.16)	0.8 (0.76–0.84)	0.41 (0.35–0.48)	1.42 (1.24–1.63)
18.59	0.55 (0.51–0.6)	0.39 (0.27–0.56)	0.23 (0.21–0.25)	1.19 (1.1–1.28)	1.43 (1.37–1.49)	0.5 (0.43–0.58）	0.57 (0.48–0.68)	1.08 (1–1.16)	0.8 (0.77–0.84)	0.42 (0.36–0.49)	1.4 (1.23–1.6)
18.76	0.56 (0.52–0.6)	0.39 (0.27–0.57)	0.24 (0.22–0.26)	1.18 (1.1–1.27)	1.41 (1.36–1.47)	0.5 (0.43–0.58）	0.58 (0.48–0.68)	1.08 (1.01–1.15)	0.81 (0.77–0.85)	0.42 (0.36–0.49)	1.38 (1.22–1.58)

18.93	0.56 (0.53–0.6)	0.4 (0.28–0.57)	0.25 (0.23–0.27)	1.17 (1.09–1.26)	1.4 (1.35–1.45)	0.51 (0.44–0.58)	0.58 (0.49–0.69)	1.08 (1.01–1.15)	0.82 (0.78–0.85)	0.43 (0.37–0.5)	1.37 (1.21–1.55)
19.10	0.57 (0.53–0.61)	0.41 (0.29–0.58)	0.25 (0.23–0.27)	1.16 (1.08–1.24)	1.39 (1.34–1.44)	0.51 (0.45–0.59)	0.59 (0.5–0.69)	1.08 (1.01–1.15)	0.82 (0.79–0.86)	0.43 (0.38–0.5)	1.35 (1.2–1.53)
19.27	0.57 (0.54–0.61)	0.42 (0.3–0.58)	0.26 (0.24–0.28)	1.15 (1.08–1.23)	1.37 (1.33–1.42)	0.52 (0.45–0.59)	0.59 (0.51–0.69)	1.08 (1.01–1.15)	0.83 (0.79–0.86)	0.44 (0.38–0.51)	1.34 (1.19–1.51)
19.44	0.58 (0.54–0.62)	0.43 (0.31–0.59)	0.27 (0.25–0.29)	1.14 (1.07–1.22)	1.36 (1.31–1.41)	0.52 (0.46–0.6)	0.6 (0.51–0.7)	1.08 (1.01–1.15)	0.83 (0.8–0.87)	0.45 (0.39–0.51)	1.32 (1.18–1.48)
19.61	0.59 (0.55–0.62)	0.44 (0.32–0.6)	0.28 (0.26–0.3)	1.14 (1.07–1.21)	1.35 (1.3–1.39)	0.53 (0.47–0.6)	0.6 (0.52–0.7)	1.08 (1.02–1.14)	0.84 (0.81–0.87)	0.45 (0.4–0.52)	1.31 (1.17–1.46)
19.78	0.59 (0.56–0.63)	0.45 (0.33–0.6)	0.28 (0.26–0.3)	1.13 (1.06–1.2)	1.33 (1.29–1.38)	0.54 (0.47–0.6)	0.61 (0.53–0.7)	1.08 (1.02–1.14)	0.85 (0.82–0.88)	0.46 (0.4–0.52)	1.29 (1.16–1.44)
19.95	0.6 (0.56–0.63)	0.45 (0.34–0.61)	0.29 (0.27–0.31)	1.12 (1.05–1.19)	1.32 (1.28–1.36)	0.54 (0.48–0.61)	0.61 (0.53–0.71)	1.08 (1.02–1.14)	0.85 (0.82–0.88)	0.47 (0.41–0.53)	1.27 (1.15–1.42)
20.13	0.6 (0.57–0.64)	0.46 (0.35–0.62)	0.3 (0.28–0.32)	1.11 (1.05–1.17)	1.31 (1.27–1.35)	0.55 (0.49–0.61)	0.62 (0.54–0.71)	1.08 (1.02–1.14)	0.86 (0.83–0.89)	0.47 (0.42–0.53)	1.26 (1.14–1.39)
20.30	0.61 (0.58–0.64)	0.47 (0.36–0.62)	0.31 (0.29–0.33)	1.1 (1.04–1.16)	1.29 (1.26–1.33)	0.55 (0.5–0.62)	0.63 (0.55–0.71)	1.08 (1.02–1.14)	0.87 (0.84–0.9)	0.48 (0.43–0.54)	1.24 (1.13–1.37)
20.47	0.61 (0.58–0.65)	0.48 (0.37–0.63)	0.32 (0.3–0.34)	1.09 (1.04–1.15)	1.28 (1.25–1.32)	0.56 (0.5–0.62)	0.63 (0.56–0.72)	1.08 (1.03–1.13)	0.87 (0.84–0.9)	0.48 (0.43–0.54)	1.23 (1.12–1.35)
20.64	0.62 (0.59–0.65)	0.49 (0.38–0.64)	0.33 (0.31–0.34)	1.08 (1.03–1.14)	1.27 (1.24–1.3)	0.56 (0.51–0.63)	0.64 (0.56–0.72)	1.08 (1.03–1.13)	0.88 (0.85–0.91)	0.49 (0.44–0.55)	1.22 (1.11–1.33)
20.81	0.63 (0.6–0.66)	0.5 (0.39–0.64)	0.33 (0.32–0.35)	1.08 (1.02–1.13)	1.26 (1.22–1.29)	0.57 (0.52–0.63)	0.64 (0.57–0.72)	1.08 (1.03–1.13)	0.89 (0.86–0.91)	0.5 (0.45–0.55)	1.2 (1.1–1.31)
20.98	0.63 (0.6–0.66)	0.51 (0.41–0.65)	0.34 (0.33–0.36)	1.07 (1.02–1.12)	1.24 (1.21–1.28)	0.58 (0.53–0.63)	0.65 (0.58–0.73)	1.08 (1.03–1.13)	0.89 (0.87–0.92)	0.5 (0.46–0.56)	1.19 (1.09–1.29)
21.15	0.64 (0.61–0.67)	0.52 (0.42–0.66)	0.35 (0.34–0.37)	1.06 (1.01–1.11)	1.23 (1.2–1.26)	0.58 (0.53–0.64)	0.65 (0.59–0.73)	1.08 (1.03–1.13)	0.9 (0.87–0.93)	0.51 (0.46–0.56)	1.17 (1.08–1.27)
21.32	0.64 (0.62–0.67)	0.53 (0.43–0.66)	0.36 (0.35–0.38)	1.05 (1.01–1.1)	1.22 (1.19–1.25)	0.59 (0.54–0.64)	0.66 (0.6–0.73)	1.08 (1.04–1.13)	0.91 (0.88–0.93)	0.52 (0.47–0.57)	1.16 (1.07–1.25)
21.49	0.65 (0.62–0.68)	0.55 (0.44–0.67)	0.37 (0.36–0.39)	1.04 (1–1.09)	1.21 (1.18–1.24)	0.6 (0.55–0.65)	0.67 (0.6–0.74)	1.08 (1.04–1.12)	0.91 (0.89–0.94)	0.53 (0.48–0.57)	1.15 (1.06–1.23)
21.66	0.66 (0.63–0.68)	0.56 (0.46–0.68)	0.38 (0.37–0.4)	1.04 (1–1.08)	1.2 (1.17–1.22)	0.6 (0.56–0.65)	0.67 (0.61–0.74)	1.08 (1.04–1.12)	0.92 (0.9–0.94)	0.53 (0.49–0.58)	1.13 (1.05–1.22)
21.83	0.66 (0.64–0.69)	0.57 (0.47–0.69)	0.39 (0.38–0.41)	1.03 (0.99–1.07)	1.19 (1.16–1.21)	0.61 (0.56–0.66)	0.68 (0.62–0.74)	1.08 (1.04–1.12)	0.93 (0.91–0.95)	0.54 (0.5–0.58)	1.12 (1.04–1.2)
22.01	0.67 (0.64–0.69)	0.58 (0.48–0.69)	0.4 (0.39–0.42)	1.02 (0.98–1.06)	1.17 (1.15–1.2)	0.62 (0.57–0.66)	0.69 (0.63–0.75)	1.08 (1.04–1.12)	0.93 (0.91–0.96)	0.55 (0.51–0.59)	1.11 (1.04–1.18)
22.18	0.67 (0.65–0.7)	0.59 (0.5–0.7)	0.42 (0.4–0.43)	1.01 (0.98–1.05)	1.16 (1.14–1.18)	0.62 (0.58–0.67)	0.69 (0.64–0.75)	1.08 (1.04–1.12)	0.94 (0.92–0.96)	0.55 (0.52–0.6)	1.09 (1.03–1.17)
22.35	0.68 (0.66–0.7)	0.6 (0.51–0.71)	0.43 (0.41–0.44)	1.01 (0.97–1.04)	1.15 (1.13–1.17)	0.63 (0.59–0.67)	0.7 (0.65–0.76)	1.08 (1.05–1.12)	0.95 (0.93–0.97)	0.56 (0.52–0.6)	1.08 (1.02–1.15)
22.52	0.69 (0.67–0.71)	0.62 (0.53–0.72)	0.44 (0.42–0.46)	1 (0.97–1.03)	1.14 (1.12–1.16)	0.64 (0.6–0.68)	0.71 (0.66–0.76)	1.08 (1.05–1.11)	0.95 (0.94–0.97)	0.57 (0.53–0.61)	1.07 (1.01–1.13)
22.69	0.69 (0.67–0.71)	0.63 (0.54–0.73)	0.45 (0.44–0.47)	0.99 (0.96–1.02)	1.13 (1.11–1.15)	0.64 (0.61–0.68)	0.71 (0.66–0.76)	1.08 (1.05–1.11)	0.96 (0.94–0.98)	0.58 (0.54–0.62)	1.06 (1–1.12)
22.86	0.7 (0.68–0.72)	0.64 (0.56–0.74)	0.46 (0.45–0.48)	0.99 (0.96–1.02)	1.12 (1.1–1.14)	0.65 (0.62–0.69)	0.72 (0.67–0.77)	1.08 (1.05–1.11)	0.97 (0.95–0.98)	0.59 (0.55–0.62)	1.05 (0.99–1.1)
23.03	0.71 (0.69–0.73)	0.65 (0.57–0.74)	0.48 (0.46–0.49)	0.98 (0.95–1.01)	1.11 (1.09–1.13)	0.66 (0.62–0.69)	0.73 (0.68–0.77)	1.08 (1.05–1.11)	0.97 (0.96–0.99)	0.59 (0.56–0.63)	1.04 (0.99–1.09)
23.20	0.72 (0.7–0.73)	0.66 (0.59–0.75)	0.49 (0.48–0.5)	0.97 (0.95–1)	1.1 (1.09–1.12)	0.67 (0.63–0.7)	0.73 (0.69–0.78)	1.08 (1.05–1.11)	0.98 (0.96–1)	0.6 (0.57–0.64)	1.03 (0.98–1.08)
23.37	0.72 (0.71–0.74)	0.68 (0.6–0.76)	0.5 (0.49–0.52)	0.97 (0.94–0.99)	1.09 (1.08–1.11)	0.67 (0.64–0.71)	0.74 (0.7–0.78)	1.08 (1.05–1.11)	0.98 (0.97–1)	0.61 (0.58–0.64)	1.02 (0.97–1.07)

23.54	0.73 (0.71–0.75)	0.69 (0.62–0.77)	0.52 (0.5–0.53)	0.96 (0.94–0.99)	1.08 (1.07–1.1)	0.68 (0.65–0.71)	0.75 (0.71–0.79)	1.08 (1.05–1.1)	0.99 (0.98–1.01)	0.62 (0.59–0.65)	1.01 (0.96–1.06)
23.71	0.74 (0.72–0.75)	0.7 (0.63–0.78)	0.53 (0.52–0.54)	0.96 (0.94–0.98)	1.07 (1.06–1.09)	0.69 (0.66–0.72)	0.75 (0.72–0.79)	1.08 (1.05–1.1)	1 (0.98–1.01)	0.63 (0.6–0.66)	1 (0.96–1.04)
23.88	0.74 (0.73–0.76)	0.72 (0.65–0.79)	0.55 (0.53–0.56)	0.95 (0.93–0.98)	1.07 (1.05–1.08)	0.7 (0.67–0.73)	0.76 (0.73–0.8)	1.08 (1.05–1.1)	1 (0.99–1.02)	0.64 (0.61–0.67)	0.99 (0.95–1.03)
24.06	0.75 (0.74–0.77)	0.73 (0.66–0.8)	0.56 (0.55–0.57)	0.95 (0.93–0.97)	1.06 (1.05–1.07)	0.71 (0.68–0.74)	0.77 (0.74–0.81)	1.07 (1.05–1.1)	1.01 (0.99–1.02)	0.65 (0.63–0.68)	0.98 (0.95–1.02)
24.23	0.76 (0.75–0.77)	0.74 (0.68–0.81)	0.58 (0.56–0.59)	0.95 (0.93–0.97)	1.05 (1.04–1.06)	0.72 (0.69–0.74)	0.78 (0.74–0.81)	1.07 (1.05–1.1)	1.01 (1–1.02)	0.66 (0.64–0.68)	0.98 (0.94–1.02)
24.40	0.77 (0.76–0.78)	0.76 (0.69–0.82)	0.59 (0.58–0.6)	0.94 (0.92–0.96)	1.04 (1.03–1.06)	0.73 (0.7–0.75)	0.79 (0.75–0.82)	1.07 (1.05–1.09)	1.02 (1–1.03)	0.67 (0.65–0.69)	0.97 (0.94–1.01)
24.57	0.78 (0.76–0.79)	0.77 (0.71–0.83)	0.61 (0.6–0.62)	0.94 (0.92–0.96)	1.04 (1.03–1.05)	0.73 (0.71–0.76)	0.79 (0.76–0.83)	1.07 (1.05–1.09)	1.02 (1.01–1.03)	0.68 (0.66–0.7)	0.97 (0.93–1)
24.74	0.79 (0.77–0.8)	0.78 (0.72–0.85)	0.62 (0.61–0.63)	0.94 (0.92–0.95)	1.03 (1.02–1.04)	0.74 (0.72–0.77)	0.8 (0.77–0.83)	1.07 (1.05–1.09)	1.02 (1.01–1.03)	0.69 (0.67–0.71)	0.96 (0.93–0.99)
24.91	0.79 (0.78–0.81)	0.8 (0.74–0.86)	0.64 (0.63–0.65)	0.94 (0.92–0.95)	1.03 (1.01–1.04)	0.75 (0.73–0.78)	0.81 (0.78–0.84)	1.07 (1.05–1.09)	1.03 (1.01–1.04)	0.7 (0.68–0.72)	0.96 (0.92–0.99)
25.08	0.8 (0.79–0.82)	0.81 (0.75–0.87)	0.66 (0.65–0.67)	0.93 (0.92–0.95)	1.02 (1.01–1.03)	0.76 (0.74–0.79)	0.82 (0.79–0.85)	1.07 (1.05–1.08)	1.03 (1.02–1.04)	0.72 (0.7–0.74)	0.95 (0.92–0.98)
25.25	0.81 (0.8–0.82)	0.82 (0.77–0.88)	0.67 (0.66–0.68)	0.93 (0.92–0.95)	1.01 (1–1.02)	0.77 (0.75–0.8)	0.83 (0.8–0.86)	1.06 (1.04–1.08)	1.03 (1.02–1.04)	0.73 (0.71–0.75)	0.95 (0.92–0.98)
25.42	0.82 (0.81–0.83)	0.83 (0.78–0.89)	0.69 (0.68–0.7)	0.93 (0.92–0.95)	1.01 (1–1.02)	0.79 (0.76–0.81)	0.84 (0.81–0.86)	1.06 (1.04–1.08)	1.03 (1.02–1.04)	0.74 (0.72–0.76)	0.95 (0.92–0.98)
25.59	0.83 (0.82–0.84)	0.85 (0.8–0.9)	0.71 (0.7–0.72)	0.93 (0.92–0.95)	1.01 (1–1.02)	0.8 (0.78–0.82)	0.85 (0.82–0.87)	1.06 (1.04–1.08)	1.04 (1.02–1.05)	0.75 (0.74–0.77)	0.94 (0.91–0.97)
25.76	0.84 (0.83–0.85)	0.86 (0.81–0.91)	0.73 (0.72–0.74)	0.93 (0.92–0.95)	1 (0.99–1.01)	0.81 (0.79–0.83)	0.85 (0.83–0.88)	1.06 (1.04–1.07)	1.04 (1.03–1.05)	0.77 (0.75–0.78)	0.94 (0.91–0.97)
25.93	0.85 (0.84–0.86)	0.87 (0.82–0.92)	0.75 (0.74–0.75)	0.93 (0.92–0.95)	1 (0.99–1.01)	0.82 (0.8–0.84)	0.86 (0.84–0.89)	1.05 (1.04–1.07)	1.04 (1.03–1.05)	0.78 (0.77–0.8)	0.94 (0.92–0.97)
26.11	0.86 (0.85–0.87)	0.88 (0.84–0.93)	0.77 (0.76–0.77)	0.94 (0.92–0.95)	1 (0.99–1)	0.83 (0.81–0.85)	0.87 (0.85–0.9)	1.05 (1.03–1.06)	1.04 (1.03–1.05)	0.8 (0.78–0.81)	0.94 (0.92–0.97)
26.28	0.87 (0.86–0.88)	0.9 (0.85–0.94)	0.78 (0.78–0.79)	0.94 (0.93–0.95)	0.99 (0.99–1)	0.84 (0.83–0.86)	0.88 (0.86–0.91)	1.05 (1.03–1.06)	1.04 (1.03–1.04)	0.81 (0.8–0.83)	0.94 (0.92–0.97)
26.45	0.88 (0.87–0.89)	0.91 (0.87–0.95)	0.8 (0.8–0.81)	0.94 (0.93–0.95)	0.99 (0.99–1)	0.86 (0.84–0.87)	0.89 (0.87–0.92)	1.04 (1.03–1.06)	1.04 (1.03–1.04)	0.83 (0.81–0.84)	0.94 (0.92–0.97)
26.62	0.9 (0.89–0.9)	0.92 (0.88–0.96)	0.82 (0.82–0.83)	0.94 (0.93–0.95)	0.99 (0.98–1)	0.87 (0.86–0.89)	0.91 (0.89–0.92)	1.04 (1.03–1.05)	1.03 (1.03–1.04)	0.84 (0.83–0.86)	0.95 (0.93–0.97)
26.79	0.91 (0.9–0.91)	0.93 (0.9–0.97)	0.84 (0.84–0.85)	0.95 (0.94–0.96)	0.99 (0.98–1)	0.89 (0.87–0.9)	0.92 (0.9–0.93)	1.03 (1.02–1.05)	1.03 (1.02–1.04)	0.86 (0.85–0.87)	0.95 (0.93–0.97)
26.96	0.92 (0.91–0.93)	0.94 (0.91–0.98)	0.87 (0.86–0.87)	0.95 (0.95–0.96)	0.99 (0.98–1)	0.9 (0.89–0.91)	0.93 (0.91–0.94)	1.03 (1.02–1.04)	1.03 (1.02–1.03)	0.88 (0.87–0.89)	0.95 (0.94–0.97)
27.13	0.93 (0.93–0.94)	0.95 (0.92–0.98)	0.89 (0.88–0.89)	0.96 (0.95–0.97)	0.99 (0.99–0.99)	0.92 (0.9–0.93)	0.94 (0.92–0.95)	1.03 (1.02–1.03)	1.03 (1.02–1.03)	0.9 (0.89–0.91)	0.96 (0.95–0.97)
27.30	0.94 (0.94–0.95)	0.96 (0.94–0.99)	0.91 (0.9–0.91)	0.97 (0.96–0.97)	0.99 (0.99–0.99)	0.93 (0.92–0.94)	0.95 (0.94–0.96)	1.02 (1.01–1.03)	1.02 (1.02–1.03)	0.91 (0.91–0.92)	0.97 (0.95–0.98)
27.47	0.96 (0.95–0.96)	0.97 (0.95–0.99)	0.93 (0.93–0.93)	0.97 (0.97–0.98)	0.99 (0.99–1)	0.95 (0.94–0.95)	0.96 (0.95–0.97)	1.02 (1.01–1.02)	1.02 (1.01–1.02)	0.93 (0.93–0.94)	0.97 (0.96–0.98)
27.64	0.97 (0.97–0.97)	0.98 (0.97–1)	0.95 (0.95–0.95)	0.98 (0.98–0.98)	0.99 (0.99–1)	0.96 (0.96–0.97)	0.97 (0.97–0.98)	1.01 (1.01–1.01)	1.01 (1.01–1.01)	0.96 (0.95–0.96)	0.98 (0.97–0.99)
27.81	0.98 (0.98–0.99)	0.99 (0.98–1)	0.98 (0.97–0.98)	0.99 (0.99–0.99)	1 (1–1)	0.98 (0.98–0.98)	0.99 (0.98–0.99)	1.01 (1–1.01)	1.01 (1.01–1.01)	0.98 (0.97–0.98)	0.99 (0.99–0.99)
27.98	1 (1–1)	1 (1–1)	1 (1–1)	1 (1–1)	1 (1–1)	1 (1–1)	1 (1–1)	1 (1–1)	1 (1–1)	1 (1–1)	1 (1–1)

**28.00**	**Reference**	**Reference**	**Reference**	**Reference**	**Reference**	**Reference**	**Reference**	**Reference**	**Reference**	**Reference**	**Reference**
28.16	1.01 (1.01–1.01)	1.01 (1–1.01)	1.02 (1.02–1.02)	1.01 (1.01–1.01)	1 (1–1)	1.02 (1.01–1.02)	1.01 (1.01–1.01)	1 (0.99–1)	0.99 (0.99–1)	1.02 (1.02–1.02)	1.01 (1.01–1.01)
28.33	1.03 (1.02–1.03)	1.01 (1–1.03)	1.04 (1.04–1.05)	1.02 (1.02–1.02)	1.01 (1–1.01)	1.03 (1.03–1.04)	1.02 (1.02–1.03)	0.99 (0.99–0.99)	0.99 (0.99–0.99)	1.04 (1.04–1.05)	1.02 (1.01–1.03)
28.50	1.04 (1.04–1.05)	1.02 (1–1.04)	1.07 (1.06–1.07)	1.03 (1.03–1.04)	1.01 (1.01–1.01)	1.05 (1.04–1.06)	1.04 (1.03–1.05)	0.98 (0.98–0.99)	0.98 (0.98–0.99)	1.07 (1.06–1.08)	1.03 (1.02–1.04)
28.67	1.06 (1.05–1.06)	1.03 (1–1.06)	1.09 (1.09–1.1)	1.04 (1.04–1.05)	1.01 (1.01–1.02)	1.07 (1.06–1.08)	1.05 (1.04–1.07)	0.98 (0.97–0.99)	0.98 (0.97–0.98)	1.09 (1.08–1.1)	1.04 (1.03–1.06)
28.84	1.07 (1.06–1.08)	1.04 (1–1.07)	1.12 (1.11–1.12)	1.05 (1.05–1.06)	1.02 (1.01–1.02)	1.09 (1.07–1.1)	1.06 (1.05–1.08)	0.97 (0.97–0.98)	0.97 (0.96–0.97)	1.12 (1.1–1.13)	1.05 (1.04–1.07)
29.01	1.08 (1.08–1.09)	1.04 (1–1.08)	1.14 (1.13–1.15)	1.07 (1.06–1.08)	1.02 (1.02–1.03)	1.11 (1.09–1.13)	1.08 (1.06–1.1)	0.97 (0.96–0.98)	0.96 (0.96–0.97)	1.14 (1.12–1.16)	1.07 (1.05–1.09)
29.18	1.1 (1.09–1.11)	1.05 (1–1.1)	1.16 (1.15–1.17)	1.08 (1.07–1.09)	1.03 (1.02–1.03)	1.12 (1.1–1.15)	1.09 (1.07–1.12)	0.96 (0.95–0.98)	0.96 (0.95–0.96)	1.17 (1.15–1.19)	1.08 (1.05–1.11)
29.35	1.11 (1.1–1.13)	1.05 (1–1.11)	1.19 (1.18–1.2)	1.1 (1.08–1.11)	1.03 (1.02–1.04)	1.14 (1.12–1.17)	1.11 (1.08–1.14)	0.96 (0.94–0.97)	0.95 (0.94–0.96)	1.19 (1.17–1.22)	1.09 (1.06–1.12)
29.52	1.13 (1.11–1.14)	1.06 (0.99–1.12)	1.21 (1.2–1.23)	1.11 (1.09–1.13)	1.04 (1.03–1.05)	1.16 (1.13–1.19)	1.12 (1.09–1.15)	0.95 (0.94–0.97)	0.94 (0.93–0.95)	1.22 (1.19–1.25)	1.11 (1.07–1.14)
29.69	1.14 (1.12–1.16)	1.06 (0.99–1.13)	1.24 (1.22–1.25)	1.12 (1.11–1.14)	1.04 (1.03–1.05)	1.18 (1.15–1.21)	1.13 (1.1–1.17)	0.95 (0.93–0.97)	0.94 (0.93–0.95)	1.25 (1.21–1.28)	1.12 (1.08–1.16)
29.86	1.15 (1.14–1.17)	1.06 (0.99–1.14)	1.26 (1.25–1.28)	1.14 (1.12–1.16)	1.05 (1.04–1.06)	1.19 (1.16–1.23)	1.15 (1.11–1.19)	0.94 (0.93–0.96)	0.93 (0.92–0.94)	1.27 (1.24–1.31)	1.13 (1.09–1.17)
30.04	1.17 (1.15–1.19)	1.06 (0.98–1.15)	1.29 (1.27–1.31)	1.16 (1.13–1.18)	1.05 (1.04–1.06)	1.21 (1.17–1.25)	1.16 (1.12–1.21)	0.94 (0.92–0.96)	0.93 (0.91–0.94)	1.3 (1.26–1.34)	1.14 (1.1–1.19)
30.21	1.18 (1.16–1.2)	1.07 (0.98–1.16)	1.31 (1.29–1.33)	1.17 (1.15–1.2)	1.06 (1.04–1.07)	1.23 (1.18–1.27)	1.17 (1.13–1.22)	0.94 (0.92–0.96)	0.92 (0.91–0.93)	1.33 (1.28–1.37)	1.16 (1.11–1.21)
30.38	1.19 (1.17–1.21)	1.07 (0.98–1.17)	1.34 (1.31–1.36)	1.19 (1.16–1.21)	1.06 (1.05–1.08)	1.24 (1.2–1.29)	1.19 (1.14–1.24)	0.93 (0.91–0.95)	0.92 (0.9–0.93)	1.35 (1.31–1.4)	1.17 (1.12–1.22)
30.55	1.2 (1.18–1.22)	1.07 (0.98–1.17)	1.36 (1.34–1.39)	1.2 (1.18–1.23)	1.07 (1.05–1.08)	1.25 (1.21–1.3)	1.2 (1.15–1.26)	0.93 (0.91–0.95)	0.91 (0.9–0.93)	1.38 (1.33–1.43)	1.18 (1.13–1.24)
30.72	1.21 (1.19–1.24)	1.07 (0.97–1.18)	1.39 (1.36–1.41)	1.22 (1.19–1.25)	1.07 (1.06–1.09)	1.27 (1.22–1.32)	1.22 (1.16–1.27)	0.93 (0.9–0.95)	0.91 (0.89–0.92)	1.41 (1.36–1.46)	1.19 (1.14–1.25)
30.89	1.22 (1.2–1.25)	1.07 (0.97–1.18)	1.41 (1.38–1.44)	1.24 (1.21–1.27)	1.08 (1.06–1.09)	1.28 (1.23–1.33)	1.23 (1.17–1.29)	0.92 (0.9–0.95)	0.9 (0.89–0.92)	1.43 (1.38–1.49)	1.21 (1.15–1.27)
31.06	1.23 (1.2–1.26)	1.07 (0.97–1.19)	1.44 (1.41–1.46)	1.26 (1.22–1.29)	1.08 (1.06–1.1)	1.29 (1.24–1.35)	1.24 (1.18–1.31)	0.92 (0.9–0.95)	0.9 (0.89–0.91)	1.46 (1.4–1.52)	1.22 (1.16–1.28)
31.23	1.24 (1.21–1.27)	1.07 (0.96–1.19)	1.46 (1.43–1.49)	1.27 (1.24–1.31)	1.08 (1.07–1.1)	1.3 (1.25–1.36)	1.26 (1.19–1.32)	0.92 (0.89–0.94)	0.9 (0.88–0.91)	1.49 (1.43–1.55)	1.23 (1.17–1.29)
31.40	1.25 (1.22–1.28)	1.07 (0.96–1.19)	1.48 (1.45–1.52)	1.29 (1.26–1.33)	1.09 (1.07–1.11)	1.31 (1.26–1.37)	1.27 (1.2–1.34)	0.92 (0.89–0.94)	0.89 (0.88–0.91)	1.51 (1.45–1.58)	1.24 (1.17–1.31)
31.57	1.26 (1.23–1.29)	1.07 (0.96–1.19)	1.51 (1.48–1.54)	1.31 (1.28–1.35)	1.09 (1.08–1.11)	1.32 (1.27–1.38)	1.28 (1.21–1.35)	0.91 (0.89–0.94)	0.89 (0.88–0.91)	1.54 (1.48–1.61)	1.25 (1.18–1.32)
31.74	1.26 (1.23–1.29)	1.07 (0.95–1.19)	1.53 (1.5–1.57)	1.33 (1.29–1.37)	1.1 (1.08–1.12)	1.33 (1.27–1.4)	1.3 (1.23–1.37)	0.91 (0.89–0.94)	0.89 (0.87–0.9)	1.57 (1.5–1.64)	1.26 (1.19–1.33)
31.91	1.27 (1.24–1.3)	1.06 (0.95–1.19)	1.56 (1.52–1.59)	1.35 (1.31–1.39)	1.1 (1.08–1.12)	1.34 (1.28–1.4)	1.31 (1.24–1.38)	0.91 (0.88–0.94)	0.89 (0.87–0.9)	1.6 (1.53–1.67)	1.27 (1.2–1.34)
32.09	1.28 (1.25–1.31)	1.06 (0.95–1.19)	1.58 (1.55–1.62)	1.37 (1.33–1.41)	1.11 (1.09–1.12)	1.35 (1.29–1.41)	1.32 (1.25–1.4)	0.91 (0.88–0.94)	0.88 (0.87–0.9)	1.62 (1.55–1.7)	1.28 (1.21–1.36)
32.26	1.28 (1.25–1.32)	1.06 (0.94–1.19)	1.61 (1.57–1.64)	1.39 (1.35–1.43)	1.11 (1.09–1.13)	1.36 (1.29–1.42)	1.33 (1.26–1.41)	0.91 (0.88–0.93)	0.88 (0.86–0.9)	1.65 (1.58–1.73)	1.29 (1.22–1.37)
32.43	1.29 (1.26–1.32)	1.05 (0.94–1.18)	1.63 (1.59–1.67)	1.41 (1.37–1.46)	1.11 (1.1–1.13)	1.36 (1.3–1.43)	1.35 (1.27–1.43)	0.91 (0.88–0.93)	0.88 (0.86–0.89)	1.68 (1.6–1.75)	1.3 (1.23–1.38)

32.60	1.3 (1.26–1.33)	1.05 (0.93–1.18)	1.65 (1.62–1.69)	1.43 (1.39–1.48)	1.12 (1.1–1.14)	1.37 (1.3–1.44)	1.36 (1.28–1.44)	0.9 (0.88–0.93)	0.88 (0.86–0.89)	1.7 (1.63–1.78)	1.31 (1.24–1.39)
32.77	1.3 (1.27–1.33)	1.05 (0.93–1.18)	1.68 (1.64–1.72)	1.46 (1.41–1.5)	1.12 (1.1–1.14)	1.37 (1.31–1.44)	1.37 (1.29–1.46)	0.9 (0.88–0.93)	0.88 (0.86–0.89)	1.73 (1.65–1.81)	1.32 (1.25–1.4)
32.94	1.31 (1.27–1.34)	1.04 (0.93–1.17)	1.7 (1.66–1.74)	1.48 (1.43–1.52)	1.13 (1.11–1.15)	1.38 (1.31–1.45)	1.39 (1.31–1.47)	0.9 (0.88–0.93)	0.87 (0.86–0.89)	1.76 (1.68–1.84)	1.33 (1.25–1.41)
33.11	1.31 (1.28–1.34)	1.04 (0.92–1.17)	1.73 (1.69–1.77)	1.5 (1.46–1.55)	1.13 (1.11–1.15)	1.38 (1.32–1.45)	1.4 (1.32–1.49)	0.9 (0.87–0.93)	0.87 (0.86–0.89)	1.78 (1.7–1.87)	1.34 (1.26–1.42)
33.28	1.31 (1.28–1.35)	1.03 (0.92–1.16)	1.75 (1.71–1.79)	1.52 (1.48–1.57)	1.13 (1.11–1.15)	1.39 (1.32–1.46)	1.41 (1.33–1.5)	0.9 (0.87–0.93)	0.87 (0.86–0.89)	1.81 (1.73–1.9)	1.35 (1.27–1.43)
33.45	1.32 (1.28–1.35)	1.03 (0.91–1.16)	1.77 (1.73–1.81)	1.55 (1.5–1.6)	1.14 (1.12–1.16)	1.39 (1.32–1.46)	1.43 (1.34–1.51)	0.9 (0.87–0.93)	0.87 (0.85–0.89)	1.84 (1.75–1.93)	1.36 (1.28–1.44)
33.62	1.32 (1.29–1.35)	1.02 (0.91–1.15)	1.8 (1.76–1.84)	1.57 (1.52–1.62)	1.14 (1.12–1.16)	1.39 (1.33–1.46)	1.44 (1.35–1.53)	0.9 (0.87–0.93)	0.87 (0.85–0.89)	1.87 (1.78–1.96)	1.37 (1.29–1.45)
33.79	1.32 (1.29–1.36)	1.02 (0.9–1.15)	1.82 (1.78–1.86)	1.6 (1.55–1.65)	1.15 (1.13–1.17)	1.39 (1.33–1.47)	1.45 (1.37–1.54)	0.9 (0.87–0.93)	0.87 (0.85–0.89)	1.89 (1.81–1.98)	1.37 (1.29–1.46)
33.96	1.32 (1.29–1.36)	1.01 (0.89–1.14)	1.84 (1.8–1.89)	1.62 (1.57–1.67)	1.15 (1.13–1.17)	1.4 (1.33–1.47)	1.46 (1.38–1.55)	0.9 (0.87–0.93)	0.87 (0.85–0.89)	1.92 (1.83–2.01)	1.38 (1.3–1.47)
34.14	1.33 (1.29–1.36)	1.01 (0.89–1.14)	1.87 (1.83–1.91)	1.65 (1.6–1.7)	1.15 (1.13–1.18)	1.4 (1.33–1.47)	1.48 (1.39–1.57)	0.9 (0.87–0.93)	0.87 (0.85–0.89)	1.95 (1.86–2.04)	1.39 (1.31–1.48)
34.31	1.33 (1.29–1.36)	1 (0.88–1.13)	1.89 (1.85–1.94)	1.67 (1.62–1.73)	1.16 (1.14–1.18)	1.4 (1.33–1.47)	1.49 (1.4–1.58)	0.9 (0.87–0.93)	0.87 (0.85–0.89)	1.98 (1.88–2.07)	1.4 (1.32–1.49)
34.48	1.33 (1.3–1.37)	0.99 (0.88–1.13)	1.92 (1.87–1.96)	1.7 (1.65–1.75)	1.16 (1.14–1.18)	1.4 (1.33–1.47)	1.5 (1.41–1.6)	0.9 (0.87–0.93)	0.87 (0.85–0.89)	2 (1.91–2.1)	1.41 (1.32–1.5)
34.65	1.33 (1.3–1.37)	0.99 (0.87–1.12)	1.94 (1.9–1.99)	1.73 (1.67–1.78)	1.17 (1.14–1.19)	1.4 (1.33–1.47)	1.51 (1.42–1.61)	0.9 (0.87–0.93)	0.87 (0.85–0.89)	2.03 (1.94–2.13)	1.41 (1.33–1.5)
34.82	1.33 (1.3–1.37)	0.98 (0.86–1.12)	1.96 (1.92–2.01)	1.75 (1.7–1.81)	1.17 (1.15–1.19)	1.4 (1.33–1.47)	1.53 (1.44–1.62)	0.9 (0.87–0.93)	0.87 (0.85–0.89)	2.06 (1.96–2.16)	1.42 (1.34–1.51)
34.99	1.33 (1.3–1.37)	0.97 (0.85–1.11)	1.99 (1.94–2.04)	1.78 (1.73–1.84)	1.17 (1.15–1.2)	1.4 (1.33–1.47)	1.54 (1.45–1.64)	0.9 (0.87–0.93)	0.87 (0.85–0.89)	2.09 (1.99–2.19)	1.43 (1.34–1.52)
35.16	1.33 (1.3–1.37)	0.97 (0.85–1.11)	2.01 (1.96–2.06)	1.81 (1.75–1.87)	1.18 (1.15–1.2)	1.4 (1.33–1.47)	1.55 (1.46–1.65)	0.9 (0.87–0.93)	0.87 (0.85–0.89)	2.11 (2.02–2.22)	1.44 (1.35–1.53)
35.33	1.33 (1.3–1.37)	0.96 (0.84–1.1)	2.04 (1.99–2.08)	1.84 (1.78–1.9)	1.18 (1.16–1.2)	1.4 (1.32–1.47)	1.57 (1.47–1.67)	0.9 (0.87–0.93)	0.87 (0.85–0.89)	2.14 (2.04–2.25)	1.45 (1.36–1.54)
35.50	1.33 (1.3–1.37)	0.95 (0.83–1.1)	2.06 (2.01–2.11)	1.87 (1.81–1.93)	1.18 (1.16–1.21)	1.39 (1.32–1.47)	1.58 (1.48–1.68)	0.9 (0.87–0.94)	0.87 (0.85–0.89)	2.17 (2.07–2.28)	1.45 (1.36–1.55)
35.67	1.33 (1.3–1.37)	0.95 (0.82–1.09)	2.08 (2.03–2.14)	1.9 (1.84–1.96)	1.19 (1.16–1.21)	1.39 (1.32–1.47)	1.59 (1.49–1.7)	0.9 (0.87–0.94)	0.87 (0.85–0.89)	2.2 (2.1–2.31)	1.46 (1.37–1.56)
35.84	1.33 (1.3–1.37)	0.94 (0.81–1.09)	2.11 (2.06–2.16)	1.93 (1.87–1.99)	1.19 (1.17–1.22)	1.39 (1.32–1.47)	1.6 (1.5–1.71)	0.9 (0.87–0.94)	0.87 (0.85–0.89)	2.23 (2.12–2.34)	1.47 (1.37–1.57)
36.02	1.33 (1.29–1.37)	0.93 (0.8–1.08)	2.13 (2.08–2.19)	1.96 (1.9–2.03)	1.2 (1.17–1.22)	1.39 (1.31–1.47)	1.62 (1.52–1.73)	0.9 (0.87–0.94)	0.87 (0.85–0.89)	2.26 (2.15–2.37)	1.47 (1.38–1.58)
36.19	1.33 (1.29–1.37)	0.93 (0.79–1.08)	2.16 (2.1–2.21)	1.99 (1.93–2.06)	1.2 (1.17–1.23)	1.39 (1.31–1.47)	1.63 (1.53–1.74)	0.91 (0.87–0.94)	0.87 (0.85–0.89)	2.29 (2.18–2.4)	1.48 (1.38–1.59)
36.36	1.33 (1.29–1.38)	0.92 (0.79–1.08)	2.18 (2.13–2.24)	2.02 (1.96–2.09)	1.2 (1.18–1.23)	1.39 (1.31–1.47)	1.64 (1.54–1.76)	0.91 (0.87–0.94)	0.87 (0.85–0.89)	2.32 (2.21–2.43)	1.49 (1.39–1.6)
36.53	1.33 (1.29–1.38)	0.91 (0.78–1.07)	2.21 (2.15–2.27)	2.06 (1.99–2.13)	1.21 (1.18–1.23)	1.38 (1.3–1.47)	1.66 (1.55–1.77)	0.91 (0.87–0.95)	0.87 (0.85–0.89)	2.35 (2.23–2.47)	1.5 (1.39–1.61)
36.70	1.33 (1.29–1.38)	0.91 (0.77–1.07)	2.23 (2.18–2.29)	2.09 (2.02–2.17)	1.21 (1.18–1.24)	1.38 (1.3–1.47)	1.67 (1.56–1.79)	0.91 (0.87–0.95)	0.87 (0.85–0.9)	2.38 (2.26–2.5)	1.5 (1.4–1.62)
36.87	1.33 (1.29–1.38)	0.9 (0.76–1.07)	2.26 (2.2–2.32)	2.12 (2.05–2.2)	1.21 (1.18–1.24)	1.38 (1.29–1.47)	1.68 (1.57–1.81)	0.91 (0.87–0.95)	0.87 (0.85–0.9)	2.41 (2.29–2.53)	1.51 (1.4–1.63)
37.04	1.33 (1.29–1.38)	0.89 (0.75–1.06)	2.29 (2.22–2.35)	2.16 (2.08–2.24)	1.22 (1.19–1.25)	1.38 (1.29–1.47)	1.7 (1.58–1.82)	0.91 (0.87–0.95)	0.87 (0.85–0.9)	2.44 (2.32–2.57)	1.52 (1.4–1.64)

37.21	1.33 (1.28–1.38)	0.89 (0.74–1.06)	2.31 (2.25–2.38)	2.19 (2.11–2.28)	1.22 (1.19–1.25)	1.37 (1.29–1.47)	1.71 (1.59–1.84)	0.91 (0.87–0.95)	0.87 (0.85–0.9)	2.47 (2.34–2.6)	1.53 (1.41–1.65)
37.38	1.33 (1.28–1.38)	0.88 (0.73–1.06)	2.34 (2.27–2.4)	2.23 (2.15–2.31)	1.22 (1.19–1.26)	1.37 (1.28–1.47)	1.72 (1.6–1.86)	0.91 (0.87–0.96)	0.87 (0.85–0.9)	2.5 (2.37–2.64)	1.53 (1.41–1.66)
37.55	1.33 (1.28–1.38)	0.87 (0.72–1.06)	2.36 (2.3–2.43)	2.27 (2.18–2.35)	1.23 (1.2–1.26)	1.37 (1.28–1.47)	1.74 (1.61–1.88)	0.91 (0.87–0.96)	0.88 (0.85–0.9)	2.53 (2.4–2.67)	1.54 (1.42–1.67)
37.72	1.33 (1.28–1.38)	0.87 (0.71–1.05)	2.39 (2.32–2.46)	2.3 (2.21–2.39)	1.23 (1.2–1.27)	1.37 (1.27–1.47)	1.75 (1.62–1.89)	0.91 (0.87–0.96)	0.88 (0.85–0.9)	2.57 (2.43–2.71)	1.55 (1.42–1.68)
37.89	1.33 (1.28–1.38)	0.86 (0.7–1.05)	2.42 (2.35–2.49)	2.34 (2.25–2.43)	1.24 (1.2–1.27)	1.36 (1.27–1.47)	1.77 (1.63–1.91)	0.91 (0.87–0.96)	0.88 (0.85–0.9)	2.6 (2.46–2.75)	1.55 (1.42–1.7)
38.07	1.33 (1.28–1.38)	0.85 (0.69–1.05)	2.45 (2.37–2.52)	2.38 (2.28–2.48)	1.24 (1.2–1.28)	1.36 (1.26–1.47)	1.78 (1.64–1.93)	0.91 (0.87–0.96)	0.88 (0.85–0.91)	2.63 (2.49–2.79)	1.56 (1.43–1.71)
38.24	1.33 (1.27–1.38)	0.85 (0.68–1.05)	2.48 (2.4–2.55)	2.42 (2.32–2.52)	1.24 (1.21–1.28)	1.36 (1.26–1.47)	1.79 (1.65–1.95)	0.92 (0.87–0.97)	0.88 (0.85–0.91)	2.67 (2.52–2.83)	1.57 (1.43–1.72)
38.41	1.33 (1.27–1.38)	0.84 (0.67–1.04)	2.5 (2.43–2.58)	2.45 (2.35–2.56)	1.25 (1.21–1.29)	1.36 (1.26–1.47)	1.81 (1.66–1.97)	0.92 (0.87–0.97)	0.88 (0.85–0.91)	2.7 (2.55–2.86)	1.58 (1.44–1.73)
38.58	1.33 (1.27–1.38)	0.83 (0.67–1.04)	2.53 (2.45–2.62)	2.49 (2.39–2.61)	1.25 (1.21–1.29)	1.35 (1.25–1.47)	1.82 (1.67–1.99)	0.92 (0.87–0.97)	0.88 (0.85–0.91)	2.74 (2.58–2.9)	1.58 (1.44–1.74)
38.75	1.33 (1.27–1.39)	0.83 (0.66–1.04)	2.56 (2.48–2.65)	2.54 (2.42–2.65)	1.26 (1.22–1.3)	1.35 (1.25–1.47)	1.84 (1.68–2.01)	0.92 (0.86–0.97)	0.88 (0.85–0.91)	2.77 (2.61–2.95)	1.59 (1.44–1.76)
38.92	1.33 (1.27–1.39)	0.82 (0.65–1.04)	2.59 (2.5–2.68)	2.58 (2.46–2.7)	1.26 (1.22–1.3)	1.35 (1.24–1.47)	1.85 (1.69–2.03)	0.92 (0.86–0.98)	0.88 (0.85–0.91)	2.81 (2.64–2.99)	1.6 (1.45–1.77)
39.09	1.32 (1.27–1.39)	0.81 (0.64–1.04)	2.62 (2.53–2.71)	2.62 (2.5–2.74)	1.26 (1.22–1.31)	1.35 (1.24–1.47)	1.87 (1.7–2.05)	0.92 (0.86–0.98)	0.88 (0.85–0.91)	2.84 (2.67–3.03)	1.61 (1.45–1.78)
39.26	1.32 (1.26–1.39)	0.81 (0.63–1.04)	2.65 (2.56–2.75)	2.66 (2.54–2.79)	1.27 (1.22–1.31)	1.34 (1.23–1.47)	1.88 (1.71–2.07)	0.92 (0.86–0.98)	0.88 (0.85–0.92)	2.88 (2.7–3.07)	1.62 (1.45–1.8)
39.43	1.32 (1.26–1.39)	0.8 (0.62–1.03)	2.68 (2.59–2.78)	2.7 (2.58–2.84)	1.27 (1.23–1.32)	1.34 (1.23–1.47)	1.9 (1.72–2.09)	0.92 (0.86–0.98)	0.88 (0.85–0.92)	2.92 (2.73–3.12)	1.62 (1.46–1.81)
39.60	1.32 (1.26–1.39)	0.8 (0.61–1.03)	2.71 (2.61–2.81)	2.75 (2.61–2.89)	1.27 (1.23–1.32)	1.34 (1.22–1.47)	1.91 (1.73–2.11)	0.92 (0.86–0.99)	0.88 (0.85–0.92)	2.95 (2.76–3.16)	1.63 (1.46–1.82)
39.77	1.32 (1.26–1.39)	0.79 (0.61–1.03)	2.74 (2.64–2.85)	2.79 (2.65–2.94)	1.28 (1.23–1.33)	1.34 (1.22–1.47)	1.93 (1.74–2.13)	0.92 (0.86–0.99)	0.88 (0.85–0.92)	2.99 (2.79–3.2)	1.64 (1.46–1.84)
39.94	1.32 (1.26–1.39)	0.78 (0.6–1.03)	2.77 (2.67–2.88)	2.84 (2.69–2.99)	1.28 (1.23–1.33)	1.34 (1.21–1.47)	1.94 (1.75–2.15)	0.92 (0.86–0.99)	0.88 (0.85–0.92)	3.03 (2.82–3.25)	1.65 (1.47–1.85)
40.12	1.32 (1.25–1.39)	0.78 (0.59–1.03)	2.81 (2.7–2.92)	2.88 (2.73–3.04)	1.29 (1.24–1.34)	1.33 (1.21–1.47)	1.96 (1.76–2.18)	0.92 (0.86–0.99)	0.88 (0.85–0.92)	3.07 (2.86–3.3)	1.65 (1.47–1.86)
40.29	1.32 (1.25–1.39)	0.77 (0.58–1.03)	2.84 (2.73–2.95)	2.93 (2.78–3.09)	1.29 (1.24–1.34)	1.33 (1.2–1.47)	1.97 (1.77–2.2)	0.93 (0.86–1)	0.88 (0.85–0.93)	3.11 (2.89–3.34)	1.66 (1.47–1.88)
40.46	1.32 (1.25–1.4)	0.77 (0.57–1.03)	2.87 (2.76–2.99)	2.98 (2.82–3.15)	1.29 (1.24–1.35)	1.33 (1.2–1.47)	1.99 (1.78–2.22)	0.93 (0.86–1)	0.89 (0.85–0.93)	3.15 (2.92–3.39)	1.67 (1.48–1.89)
40.63	1.32 (1.25–1.4)	0.76 (0.56–1.03)	2.9 (2.79–3.03)	3.02 (2.86–3.2)	1.3 (1.24–1.35)	1.33 (1.19–1.47)	2.01 (1.79–2.24)	0.93 (0.86–1)	0.89 (0.85–0.93)	3.19 (2.95–3.44)	1.68 (1.48–1.9)
40.80	1.32 (1.25–1.4)	0.75 (0.56–1.02)	2.94 (2.81–3.07)	3.07 (2.9–3.26)	1.3 (1.25–1.36)	1.32 (1.19–1.47)	2.02 (1.8–2.27)	0.93 (0.86–1)	0.89 (0.85–0.93)	3.23 (2.99–3.49)	1.69 (1.48–1.92)
40.97	1.32 (1.24–1.4)	0.75 (0.55–1.02)	2.97 (2.84–3.1)	3.12 (2.95–3.31)	1.31 (1.25–1.36)	1.32 (1.18–1.47)	2.04 (1.81–2.29)	0.93 (0.86–1.01)	0.89 (0.85–0.93)	3.27 (3.02–3.54)	1.69 (1.49–1.93)
41.14	1.32 (1.24–1.4)	0.74 (0.54–1.02)	3.01 (2.87–3.14)	3.17 (2.99–3.37)	1.31 (1.25–1.37)	1.32 (1.18–1.48)	2.06 (1.82–2.32)	0.93 (0.86–1.01)	0.89 (0.84–0.93)	3.31 (3.06–3.59)	1.7 (1.49–1.95)
41.31	1.32 (1.24–1.4)	0.74 (0.53–1.02)	3.04 (2.91–3.18)	3.23 (3.03–3.43)	1.31 (1.26–1.37)	1.32 (1.18–1.48)	2.07 (1.83–2.34)	0.93 (0.86–1.01)	0.89 (0.84–0.94)	3.35 (3.09–3.64)	1.71 (1.49–1.96)
41.48	1.32 (1.24–1.4)	0.73 (0.53–1.02)	3.07 (2.94–3.22)	3.28 (3.08–3.49)	1.32 (1.26–1.38)	1.31 (1.17–1.48)	2.09 (1.84–2.36)	0.93 (0.85–1.01)	0.89 (0.84–0.94)	3.4 (3.13–3.69)	1.72 (1.49–1.98)
41.65	1.32 (1.24–1.4)	0.73 (0.52–1.02)	3.11 (2.97–3.26)	3.33 (3.13–3.55)	1.32 (1.26–1.39)	1.31 (1.17–1.48)	2.11 (1.86–2.39)	0.93 (0.85–1.02)	0.89 (0.84–0.94)	3.44 (3.16–3.75)	1.73 (1.5–1.99)

41.82	1.32 (1.23–1.4)	0.72 (0.51–1.02)	3.15 (3–3.3)	3.38 (3.17–3.61)	1.33 (1.26–1.39)	1.31 (1.16–1.48)	2.12 (1.87–2.41)	0.93 (0.85–1.02)	0.89 (0.84–0.94)	3.49 (3.2–3.8)	1.74 (1.5–2.01)
41.99	1.32 (1.23–1.41)	0.72 (0.5–1.02)	3.18 (3.03–3.34)	3.44 (3.22–3.67)	1.33 (1.27–1.4)	1.31 (1.16–1.48)	2.14 (1.88–2.44)	0.93 (0.85–1.02)	0.89 (0.84–0.94)	3.53 (3.23–3.86)	1.74 (1.5–2.02)
42.17	1.31 (1.23–1.41)	0.71 (0.5–1.02)	3.22 (3.06–3.38)	3.5 (3.27–3.74)	1.33 (1.27–1.4)	1.31 (1.15–1.48)	2.16 (1.89–2.46)	0.93 (0.85–1.03)	0.89 (0.84–0.94)	3.58 (3.27–3.91)	1.75 (1.51–2.04)
42.34	1.31 (1.23–1.41)	0.7 (0.49–1.01)	3.26 (3.09–3.42)	3.55 (3.32–3.8)	1.34 (1.27–1.41)	1.3 (1.15–1.48)	2.17 (1.9–2.49)	0.94 (0.85–1.03)	0.89 (0.84–0.94)	3.62 (3.31–3.97)	1.76 (1.51–2.05)
42.51	1.31 (1.23–1.41)	0.7 (0.48–1.01)	3.29 (3.13–3.47)	3.61 (3.37–3.87)	1.34 (1.27–1.41)	1.3 (1.14–1.48)	2.19 (1.91–2.52)	0.94 (0.85–1.03)	0.89 (0.84–0.95)	3.67 (3.34–4.03)	1.77 (1.51–2.07)
42.68	1.31 (1.22–1.41)	0.69 (0.48–1.01)	3.33 (3.16–3.51)	3.67 (3.42–3.94)	1.35 (1.28–1.42)	1.3 (1.14–1.48)	2.21 (1.92–2.54)	0.94 (0.85–1.03)	0.89 (0.84–0.95)	3.72 (3.38–4.08)	1.78 (1.52–2.09)
42.85	1.31 (1.22–1.41)	0.69 (0.47–1.01)	3.37 (3.19–3.55)	3.73 (3.47–4)	1.35 (1.28–1.42)	1.3 (1.13–1.48)	2.23 (1.93–2.57)	0.94 (0.85–1.04)	0.89 (0.84–0.95)	3.76 (3.42–4.14)	1.79 (1.52–2.1)
43.02	1.31 (1.22–1.41)	0.68 (0.46–1.01)	3.41 (3.23–3.6)	3.79 (3.52–4.07)	1.35 (1.28–1.43)	1.29 (1.13–1.48)	2.24 (1.94–2.6)	0.94 (0.85–1.04)	0.89 (0.84–0.95)	3.81 (3.46–4.2)	1.8 (1.52–2.12)
43.19	1.31 (1.22–1.41)	0.68 (0.46–1.01)	3.45 (3.26–3.64)	3.85 (3.57–4.15)	1.36 (1.29–1.44)	1.29 (1.12–1.49)	2.26 (1.95–2.62)	0.94 (0.85–1.04)	0.9 (0.84–0.95)	3.86 (3.49–4.26)	1.8 (1.52–2.13)
43.36	1.31 (1.21–1.41)	0.67 (0.45–1.01)	3.49 (3.3–3.69)	3.91 (3.63–4.22)	1.36 (1.29–1.44)	1.29 (1.12–1.49)	2.28 (1.96–2.65)	0.94 (0.85–1.04)	0.9 (0.84–0.95)	3.91 (3.53–4.33)	1.81 (1.53–2.15)
43.53	1.31 (1.21–1.42)	0.67 (0.44–1.01)	3.53 (3.33–3.73)	3.97 (3.68–4.29)	1.37 (1.29–1.45)	1.29 (1.11–1.49)	2.3 (1.97–2.68)	0.94 (0.85–1.05)	0.9 (0.84–0.96)	3.96 (3.57–4.39)	1.82 (1.53–2.17)
43.70	1.31 (1.21–1.42)	0.66 (0.44–1.01)	3.57 (3.37–3.78)	4.04 (3.73–4.37)	1.37 (1.29–1.45)	1.29 (1.11–1.49)	2.32 (1.98–2.71)	0.94 (0.85–1.05)	0.9 (0.84–0.96)	4.01 (3.61–4.45)	1.83 (1.53–2.18)
43.87	1.31 (1.21–1.42)	0.66 (0.43–1.01)	3.61 (3.4–3.83)	4.1 (3.79–4.44)	1.38 (1.3–1.46)	1.28 (1.11–1.49)	2.34 (1.99–2.74)	0.94 (0.84–1.05)	0.9 (0.84–0.96)	4.06 (3.65–4.52)	1.84 (1.54–2.2)
44.05	1.31 (1.21–1.42)	0.65 (0.42–1.01)	3.65 (3.44–3.87)	4.17 (3.85–4.52)	1.38 (1.3–1.47)	1.28 (1.1–1.49)	2.35 (2–2.77)	0.94 (0.84–1.06)	0.9 (0.84–0.96)	4.11 (3.69–4.58)	1.85 (1.54–2.22)
44.22	1.31 (1.2–1.42)	0.65 (0.42–1.01)	3.69 (3.47–3.92)	4.24 (3.9–4.6)	1.38 (1.3–1.47)	1.28 (1.1–1.49)	2.37 (2.01–2.79)	0.94 (0.84–1.06)	0.9 (0.84–0.96)	4.17 (3.74–4.65)	1.86 (1.54–2.23)
44.39	1.31 (1.2–1.42)	0.64 (0.41–1)	3.73 (3.51–3.97)	4.31 (3.96–4.68)	1.39 (1.3–1.48)	1.28 (1.09–1.49)	2.39 (2.03–2.82)	0.95 (0.84–1.06)	0.9 (0.84–0.96)	4.22 (3.78–4.72)	1.87 (1.55–2.25)
44.56	1.31 (1.2–1.42)	0.64 (0.41–1)	3.78 (3.55–4.02)	4.38 (4.02–4.76)	1.39 (1.31–1.48)	1.27 (1.09–1.49)	2.41 (2.04–2.85)	0.95 (0.84–1.06)	0.9 (0.84–0.97)	4.28 (3.82–4.79)	1.87 (1.55–2.27)
44.73	1.31 (1.2–1.42)	0.63 (0.4–1)	3.82 (3.58–4.07)	4.45 (4.08–4.85)	1.4 (1.31–1.49)	1.27 (1.08–1.49)	2.43 (2.05–2.88)	0.95 (0.84–1.07)	0.9 (0.84–0.97)	4.33 (3.86–4.86)	1.88 (1.55–2.29)
44.90	1.31 (1.2–1.43)	0.63 (0.39–1)	3.86 (3.62–4.12)	4.52 (4.14–4.93)	1.4 (1.31–1.49)	1.27 (1.08–1.49)	2.45 (2.06–2.92)	0.95 (0.84–1.07)	0.9 (0.84–0.97)	4.39 (3.9–4.93)	1.89 (1.55–2.3)
45.07	1.31 (1.19–1.43)	0.62 (0.39–1)	3.91 (3.66–4.17)	4.59 (4.2–5.02)	1.4 (1.32–1.5)	1.27 (1.07–1.5)	2.47 (2.07–2.95)	0.95 (0.84–1.07)	0.9 (0.84–0.97)	4.44 (3.95–5)	1.9 (1.56–2.32)
45.24	1.31 (1.19–1.43)	0.62 (0.38–1)	3.95 (3.7–4.22)	4.67 (4.27–5.1)	1.41 (1.32–1.51)	1.26 (1.07–1.5)	2.49 (2.08–2.98)	0.95 (0.84–1.08)	0.9 (0.84–0.97)	4.5 (3.99–5.07)	1.91 (1.56–2.34)
45.41	1.3 (1.19–1.43)	0.61 (0.38–1)	4 (3.74–4.28)	4.74 (4.33–5.19)	1.41 (1.32–1.51)	1.26 (1.06–1.5)	2.51 (2.09–3.01)	0.95 (0.84–1.08)	0.9 (0.84–0.98)	4.56 (4.04–5.15)	1.92 (1.56–2.36)
45.58	1.3 (1.19–1.43)	0.61 (0.37–1)	4.04 (3.78–4.33)	4.82 (4.39–5.28)	1.42 (1.32–1.52)	1.26 (1.06–1.5)	2.53 (2.1–3.04)	0.95 (0.84–1.08)	0.9 (0.84–0.98)	4.62 (4.08–5.22)	1.93 (1.57–2.38)
45.75	1.3 (1.19–1.43)	0.61 (0.37–1)	4.09 (3.82–4.38)	4.9 (4.46–5.38)	1.42 (1.33–1.53)	1.26 (1.06–1.5)	2.55 (2.11–3.07)	0.95 (0.84–1.08)	0.9 (0.84–0.98)	4.67 (4.13–5.3)	1.94 (1.57–2.39)
45.92	1.3 (1.18–1.43)	0.6 (0.36–1)	4.14 (3.86–4.44)	4.98 (4.53–5.47)	1.43 (1.33–1.53)	1.26 (1.05–1.5)	2.57 (2.13–3.11)	0.95 (0.84–1.09)	0.91 (0.84–0.98)	4.73 (4.17–5.37)	1.95 (1.57–2.41)
46.10	1.3 (1.18–1.44)	0.6 (0.36–1)	4.18 (3.9–4.49)	5.06 (4.59–5.57)	1.43 (1.33–1.54)	1.25 (1.05–1.5)	2.59 (2.14–3.14)	0.95 (0.84–1.09)	0.91 (0.84–0.98)	4.8 (4.22–5.45)	1.96 (1.57–2.43)
46.27	1.3 (1.18–1.44)	0.59 (0.35–1)	4.23 (3.94–4.55)	5.14 (4.66–5.66)	1.44 (1.33–1.54)	1.25 (1.04–1.5)	2.61 (2.15–3.17)	0.95 (0.83–1.09)	0.91 (0.84–0.98)	4.86 (4.26–5.53)	1.97 (1.58–2.45)

46.44	1.3 (1.18–1.44)	0.59 (0.35–1)	4.28 (3.98–4.61)	5.22 (4.73–5.76)	1.44 (1.34–1.55)	1.25 (1.04–1.5)	2.63 (2.16–3.21)	0.96 (0.83–1.1)	0.91 (0.84–0.99)	4.92 (4.31–5.61)	1.98 (1.58–2.47)
46.61	1.3 (1.18–1.44)	0.58 (0.34–1)	4.33 (4.02–4.66)	5.31 (4.8–5.87)	1.44 (1.34–1.56)	1.25 (1.03–1.51)	2.65 (2.17–3.24)	0.96 (0.83–1.1)	0.91 (0.83–0.99)	4.98 (4.36–5.69)	1.99 (1.58–2.49)
46.78	1.3 (1.17–1.44)	0.58 (0.34–0.99)	4.38 (4.06–4.72)	5.39 (4.87–5.97)	1.45 (1.34–1.56)	1.25 (1.03–1.51)	2.67 (2.18–3.28)	0.96 (0.83–1.1)	0.91 (0.83–0.99)	5.05 (4.41–5.78)	1.99 (1.59–2.51)
46.95	1.3 (1.17–1.44)	0.57 (0.33–0.99)	4.43 (4.1–4.78)	5.48 (4.94–6.07)	1.45 (1.35–1.57)	1.24 (1.03–1.51)	2.7 (2.2–3.31)	0.96 (0.83–1.1)	0.91 (0.83–0.99)	5.11 (4.46–5.86)	2 (1.59–2.53)
47.12	1.3 (1.17–1.44)	0.57 (0.33–0.99)	4.48 (4.15–4.84)	5.57 (5.02–6.18)	1.46 (1.35–1.58)	1.24 (1.02–1.51)	2.72 (2.21–3.35)	0.96 (0.83–1.11)	0.91 (0.83–0.99)	5.18 (4.51–5.95)	2.01 (1.59–2.55)
47.29	1.3 (1.17–1.44)	0.57 (0.32–0.99)	4.53 (4.19–4.9)	5.66 (5.09–6.29)	1.46 (1.35–1.58)	1.24 (1.02–1.51)	2.74 (2.22–3.38)	0.96 (0.83–1.11)	0.91 (0.83–0.99)	5.24 (4.56–6.04)	2.02 (1.6–2.57)
47.46	1.3 (1.17–1.45)	0.56 (0.32–0.99)	4.58 (4.24–4.96)	5.75 (5.17–6.4)	1.47 (1.35–1.59)	1.24 (1.01–1.51)	2.76 (2.23–3.42)	0.96 (0.83–1.11)	0.91 (0.83–1)	5.31 (4.61–6.12)	2.03 (1.6–2.59)
47.63	1.3 (1.16–1.45)	0.56 (0.31–0.99)	4.64 (4.28–5.02)	5.84 (5.24–6.51)	1.47 (1.36–1.59)	1.23 (1.01–1.51)	2.78 (2.24–3.45)	0.96 (0.83–1.12)	0.91 (0.83–1)	5.38 (4.66–6.21)	2.04 (1.6–2.61)
47.80	1.3 (1.16–1.45)	0.55 (0.31–0.99)	4.69 (4.32–5.09)	5.94 (5.32–6.62)	1.48 (1.36–1.6)	1.23 (1–1.51)	2.81 (2.26–3.49)	0.96 (0.83–1.12)	0.91 (0.83–1)	5.45 (4.71–6.3)	2.05 (1.6–2.63)
47.97	1.3 (1.16–1.45)	0.55 (0.3–0.99)	4.74 (4.37–5.15)	6.03 (5.4–6.74)	1.48 (1.36–1.61)	1.23 (1–1.51)	2.83 (2.27–3.53)	0.96 (0.83–1.12)	0.91 (0.83–1)	5.52 (4.76–6.4)	2.06 (1.61–2.65)
48.15	1.3 (1.16–1.45)	0.54 (0.3–0.99)	4.8 (4.42–5.21)	6.13 (5.48–6.86)	1.48 (1.37–1.61)	1.23 (1–1.51)	2.85 (2.28–3.57)	0.96 (0.83–1.13)	0.91 (0.83–1)	5.59 (4.81–6.49)	2.07 (1.61–2.67)
48.32	1.3 (1.16–1.45)	0.54 (0.3–0.99)	4.85 (4.46–5.28)	6.23 (5.56–6.98)	1.49 (1.37–1.62)	1.23 (0.99–1.52)	2.87 (2.29–3.6)	0.97 (0.83–1.13)	0.91 (0.83–1.01)	5.66 (4.87–6.59)	2.08 (1.61–2.69)
48.49	1.3 (1.15–1.45)	0.54 (0.29–0.99)	4.91 (4.51–5.35)	6.33 (5.64–7.1)	1.49 (1.37–1.63)	1.22 (0.99–1.52)	2.9 (2.3–3.64)	0.97 (0.82–1.13)	0.91 (0.83–1.01)	5.73 (4.92–6.68)	2.09 (1.62–2.71)
48.66	1.29 (1.15–1.45)	0.53 (0.29–0.99)	4.97 (4.56–5.41)	6.43 (5.73–7.23)	1.5 (1.37–1.63)	1.22 (0.98–1.52)	2.92 (2.32–3.68)	0.97 (0.82–1.13)	0.92 (0.83–1.01)	5.81 (4.97–6.78)	2.1 (1.62–2.73)
48.83	1.29 (1.15–1.46)	0.53 (0.28–0.99)	5.02 (4.6–5.48)	6.54 (5.81–7.35)	1.5 (1.38–1.64)	1.22 (0.98–1.52)	2.94 (2.33–3.72)	0.97 (0.82–1.14)	0.92 (0.83–1.01)	5.88 (5.03–6.88)	2.11 (1.62–2.75)
49.00	1.29 (1.15–1.46)	0.52 (0.28–0.99)	5.08 (4.65–5.55)	6.64 (5.9–7.48)	1.51 (1.38–1.65)	1.22 (0.97–1.52)	2.97 (2.34–3.76)	0.97 (0.82–1.14)	0.92 (0.83–1.01)	5.96 (5.08–6.98)	2.12 (1.62–2.78)

Continuation of Table S7

Maternal age	Cesarean section	Postpartum hemorrhage	Perinatal asphyxia	Large-for-gestational-age infants	Small-for-gestational-age infants	Premature	Stillbirth	Congenital malformation	ART
	OR(95% CI)	OR(95% CI)	OR(95% CI)	OR(95% CI)	OR(95% CI)	OR(95% CI)	OR(95% CI)	OR(95% CI)	OR(95% CI)
15.00	0.33 (0.31–0.36)	0.64 (0.57–0.72)	1.28 (1.15–1.43)	0.49 (0.42–0.56)	1.2 (1.1–1.5)	1.41 (1.29–1.54)	2.65 (1.86–3.78)	1.34 (0.96–1.86)	0.001699 (0.000895–0.003224)
15.17	0.34 (0.32–0.36)	0.64 (0.58–0.72)	1.28 (1.15–1.43)	0.49 (0.43–0.56)	1.2 (1.1–1.5)	1.4 (1.28–1.52)	2.6 (1.84–3.69)	1.33 (0.96–1.84)	0.001856 (0.000988–0.003485)
15.34	0.34 (0.32–0.36)	0.65 (0.58–0.72)	1.28 (1.15–1.42)	0.5 (0.44–0.57)	1.2 (1–1.4)	1.38 (1.27–1.51)	2.56 (1.82–3.61)	1.33 (0.97–1.82)	0.002028 (0.001091–0.003768)
15.51	0.35 (0.33–0.37)	0.65 (0.59–0.72)	1.28 (1.15–1.42)	0.5 (0.44–0.57)	1.2 (1–1.4)	1.37 (1.26–1.49)	2.52 (1.8–3.52)	1.32 (0.97–1.8)	0.002215 (0.001205–0.004074)
15.68	0.35 (0.33–0.37)	0.65 (0.59–0.72)	1.27 (1.15–1.41)	0.51 (0.45–0.58)	1.2 (1–1.4)	1.36 (1.25–1.48)	2.48 (1.78–3.44)	1.31 (0.97–1.78)	0.00242 (0.00133–0.004404)
15.85	0.35 (0.33–0.37)	0.66 (0.59–0.72)	1.27 (1.15–1.4)	0.51 (0.45–0.58)	1.2 (1–1.4)	1.35 (1.24–1.46)	2.44 (1.76–3.37)	1.31 (0.97–1.77)	0.002644 (0.001469–0.004761)
16.03	0.36 (0.34–0.38)	0.66 (0.6–0.73)	1.27 (1.15–1.4)	0.52 (0.46–0.59)	1.2 (1–1.4)	1.34 (1.24–1.45)	2.4 (1.74–3.29)	1.3 (0.97–1.75)	0.002889 (0.001621–0.005148)
16.20	0.36 (0.34–0.38)	0.66 (0.6–0.73)	1.27 (1.15–1.39)	0.53 (0.47–0.59)	1.2 (1–1.4)	1.33 (1.23–1.43)	2.36 (1.73–3.21)	1.29 (0.97–1.73)	0.003156 (0.00179–0.005565)
16.37	0.37 (0.35–0.39)	0.66 (0.6–0.73)	1.26 (1.15–1.39)	0.53 (0.47–0.6)	1.2 (1–1.4)	1.31 (1.22–1.42)	2.32 (1.71–3.14)	1.29 (0.97–1.71)	0.003448 (0.001976–0.006017)
16.54	0.37 (0.35–0.39)	0.67 (0.61–0.73)	1.26 (1.15–1.38)	0.54 (0.48–0.6)	1.2 (1–1.3)	1.3 (1.21–1.4)	2.28 (1.69–3.07)	1.28 (0.97–1.69)	0.003767 (0.002182–0.006505)
16.71	0.38 (0.36–0.4)	0.67 (0.61–0.73)	1.26 (1.15–1.38)	0.55 (0.49–0.61)	1.2 (1–1.3)	1.29 (1.2–1.39)	2.24 (1.67–3)	1.28 (0.97–1.67)	0.004116 (0.002408–0.007033)
16.88	0.38 (0.36–0.4)	0.67 (0.62–0.74)	1.26 (1.15–1.37)	0.55 (0.49–0.62)	1.2 (1–1.3)	1.28 (1.19–1.38)	2.2 (1.66–2.93)	1.27 (0.97–1.66)	0.004496 (0.002659–0.007603)
17.05	0.38 (0.37–0.4)	0.68 (0.62–0.74)	1.25 (1.15–1.37)	0.56 (0.5–0.62)	1.1 (1–1.3)	1.27 (1.18–1.36)	2.17 (1.64–2.87)	1.26 (0.97–1.64)	0.004912 (0.002936–0.00822)
17.22	0.39 (0.37–0.41)	0.68 (0.62–0.74)	1.25 (1.15–1.36)	0.56 (0.51–0.63)	1.1 (1–1.3)	1.26 (1.18–1.35)	2.13 (1.62–2.8)	1.26 (0.98–1.62)	0.005367 (0.003241–0.008887)
17.39	0.39 (0.38–0.41)	0.68 (0.63–0.74)	1.25 (1.15–1.36)	0.57 (0.52–0.63)	1.1 (1–1.3)	1.25 (1.17–1.33)	2.1 (1.61–2.74)	1.25 (0.98–1.6)	0.005863 (0.003578–0.009608)
17.56	0.4 (0.38–0.42)	0.69 (0.63–0.74)	1.25 (1.15–1.35)	0.58 (0.52–0.64)	1.1 (1–1.3)	1.24 (1.16–1.32)	2.06 (1.59–2.68)	1.25 (0.98–1.59)	0.006406 (0.00395–0.010388)
17.73	0.4 (0.39–0.42)	0.69 (0.64–0.75)	1.24 (1.15–1.35)	0.58 (0.53–0.64)	1.1 (1–1.2)	1.23 (1.15–1.31)	2.03 (1.57–2.62)	1.24 (0.98–1.57)	0.006998 (0.004361–0.011231)
17.90	0.41 (0.39–0.43)	0.69 (0.64–0.75)	1.24 (1.15–1.34)	0.59 (0.54–0.65)	1.1 (1–1.2)	1.22 (1.14–1.29)	1.99 (1.56–2.56)	1.23 (0.98–1.55)	0.007646 (0.004814–0.012142)
18.08	0.41 (0.4–0.43)	0.69 (0.64–0.75)	1.24 (1.15–1.34)	0.6 (0.55–0.66)	1.1 (1–1.2)	1.2 (1.13–1.28)	1.96 (1.54–2.5)	1.23 (0.98–1.54)	0.008353 (0.005315–0.013127)
18.25	0.42 (0.4–0.44)	0.7 (0.65–0.75)	1.24 (1.15–1.33)	0.61 (0.55–0.66)	1.1 (1–1.2)	1.19 (1.13–1.27)	1.93 (1.52–2.44)	1.22 (0.98–1.52)	0.009126 (0.005868–0.014192)
18.42	0.42 (0.41–0.44)	0.7 (0.65–0.75)	1.23 (1.15–1.33)	0.61 (0.56–0.67)	1.1 (1–1.2)	1.18 (1.12–1.25)	1.9 (1.51–2.39)	1.22 (0.98–1.51)	0.00997 (0.006478–0.015344)
18.59	0.43 (0.41–0.45)	0.7 (0.66–0.76)	1.23 (1.15–1.32)	0.62 (0.57–0.68)	1.1 (1–1.2)	1.17 (1.11–1.24)	1.87 (1.49–2.34)	1.21 (0.98–1.49)	0.010892 (0.007152–0.016589)
18.76	0.43 (0.42–0.45)	0.71 (0.66–0.76)	1.23 (1.15–1.32)	0.63 (0.58–0.68)	1.1 (1–1.2)	1.16 (1.1–1.23)	1.84 (1.48–2.28)	1.2 (0.98–1.48)	0.0119 (0.007895–0.017935)

18.93	0.44 (0.42–0.45)	0.71 (0.67–0.76)	1.23 (1.15–1.31)	0.63 (0.58–0.69)	1.1 (1–1.2)	1.15 (1.09–1.22)	1.81 (1.46–2.23)	1.2 (0.98–1.46)	0.013001 (0.008716–0.019391)
19.10	0.44 (0.43–0.46)	0.71 (0.67–0.76)	1.23 (1.15–1.31)	0.64 (0.59–0.7)	1 (1–1.2)	1.14 (1.09–1.2)	1.78 (1.44–2.18)	1.19 (0.98–1.44)	0.014203 (0.009623–0.020965)
19.27	0.45 (0.43–0.46)	0.72 (0.67–0.76)	1.22 (1.15–1.3)	0.65 (0.6–0.7)	1 (0.9–1.1)	1.13 (1.08–1.19)	1.75 (1.43–2.13)	1.19 (0.98–1.43)	0.02 (0.01–0.02)
19.44	0.45 (0.44–0.47)	0.72 (0.68–0.77)	1.22 (1.15–1.3)	0.66 (0.61–0.71)	1 (0.9–1.1)	1.12 (1.07–1.18)	1.72 (1.41–2.08)	1.18 (0.99–1.42)	0.02 (0.01–0.02)
19.61	0.46 (0.44–0.47)	0.72 (0.68–0.77)	1.22 (1.15–1.29)	0.66 (0.62–0.71)	1 (0.9–1.1)	1.11 (1.06–1.17)	1.69 (1.4–2.04)	1.18 (0.99–1.4)	0.02 (0.01–0.03)
19.78	0.46 (0.45–0.48)	0.73 (0.69–0.77)	1.22 (1.15–1.29)	0.67 (0.63–0.72)	1 (0.9–1.1)	1.1 (1.05–1.16)	1.66 (1.38–1.99)	1.17 (0.99–1.39)	0.02 (0.01–0.03)
19.95	0.47 (0.46–0.49)	0.73 (0.69–0.77)	1.21 (1.15–1.28)	0.68 (0.64–0.73)	1 (0.9–1.1)	1.09 (1.05–1.14)	1.63 (1.37–1.95)	1.16 (0.99–1.37)	0.02 (0.02–0.03)
20.13	0.48 (0.46–0.49)	0.73 (0.7–0.77)	1.21 (1.15–1.28)	0.69 (0.64–0.74)	1 (0.9–1.1)	1.09 (1.04–1.13)	1.61 (1.35–1.91)	1.16 (0.99–1.36)	0.02 (0.02–0.03)
20.30	0.48 (0.47–0.5)	0.74 (0.7–0.78)	1.21 (1.15–1.27)	0.7 (0.65–0.74)	1 (0.9–1.1)	1.08 (1.03–1.12)	1.58 (1.34–1.86)	1.15 (0.99–1.34)	0.03 (0.02–0.04)
20.47	0.49 (0.47–0.5)	0.74 (0.7–0.78)	1.21 (1.15–1.27)	0.7 (0.66–0.75)	1 (0.9–1.1)	1.07 (1.02–1.11)	1.55 (1.33–1.82)	1.15 (0.99–1.33)	0.03 (0.02–0.04)
20.64	0.49 (0.48–0.51)	0.74 (0.71–0.78)	1.2 (1.15–1.26)	0.71 (0.67–0.76)	1 (0.9–1.1)	1.06 (1.02–1.1)	1.53 (1.31–1.78)	1.14 (0.99–1.32)	0.03 (0.02–0.04)
20.81	0.5 (0.49–0.51)	0.75 (0.71–0.78)	1.2 (1.15–1.26)	0.72 (0.68–0.76)	1 (0.9–1)	1.05 (1.01–1.09)	1.5 (1.3–1.74)	1.14 (0.99–1.3)	0.03 (0.03–0.05)
20.98	0.5 (0.49–0.52)	0.75 (0.72–0.78)	1.2 (1.15–1.25)	0.73 (0.69–0.77)	1 (0.9–1)	1.04 (1–1.08)	1.48 (1.28–1.7)	1.13 (0.99–1.29)	0.04 (0.03–0.05)
21.15	0.51 (0.5–0.52)	0.75 (0.72–0.79)	1.2 (1.15–1.25)	0.74 (0.7–0.78)	1 (0.9–1)	1.03 (1–1.07)	1.45 (1.27–1.67)	1.13 (0.99–1.28)	0.04 (0.03–0.05)
21.32	0.52 (0.5–0.53)	0.76 (0.73–0.79)	1.19 (1.15–1.24)	0.75 (0.71–0.79)	1 (0.9–1)	1.02 (0.99–1.05)	1.43 (1.25–1.63)	1.12 (0.99–1.27)	0.04 (0.03–0.06)
21.49	0.52 (0.51–0.53)	0.76 (0.73–0.79)	1.19 (1.15–1.24)	0.76 (0.72–0.79)	0.9 (0.9–1)	1.01 (0.98–1.04)	1.41 (1.24–1.6)	1.11 (0.99–1.25)	0.05 (0.04–0.06)
21.66	0.53 (0.52–0.54)	0.76 (0.74–0.79)	1.19 (1.15–1.23)	0.76 (0.73–0.8)	0.9 (0.9–1)	1 (0.97–1.03)	1.38 (1.23–1.56)	1.11 (0.99–1.24)	0.05 (0.04–0.07)
21.83	0.54 (0.52–0.55)	0.77 (0.74–0.8)	1.19 (1.15–1.23)	0.77 (0.74–0.81)	0.9 (0.9–1)	1 (0.97–1.02)	1.36 (1.21–1.53)	1.1 (0.99–1.23)	0.06 (0.05–0.07)
22.01	0.54 (0.53–0.55)	0.77 (0.75–0.8)	1.18 (1.14–1.23)	0.78 (0.75–0.82)	0.9 (0.9–1)	0.99 (0.96–1.01)	1.34 (1.2–1.5)	1.1 (0.99–1.22)	0.06 (0.05–0.08)
22.18	0.55 (0.54–0.56)	0.77 (0.75–0.8)	1.18 (1.14–1.22)	0.79 (0.76–0.82)	0.9 (0.9–1)	0.98 (0.95–1)	1.32 (1.18–1.47)	1.09 (0.99–1.21)	0.07 (0.06–0.09)
22.35	0.56 (0.55–0.57)	0.78 (0.75–0.8)	1.18 (1.14–1.22)	0.8 (0.77–0.83)	0.9 (0.9–1)	0.97 (0.95–1)	1.3 (1.17–1.44)	1.09 (0.99–1.2)	0.08 (0.06–0.09)
22.52	0.56 (0.55–0.57)	0.78 (0.76–0.81)	1.18 (1.14–1.21)	0.81 (0.78–0.84)	0.9 (0.9–1)	0.96 (0.94–0.99)	1.28 (1.16–1.41)	1.08 (0.99–1.19)	0.08 (0.07–0.1)
22.69	0.57 (0.56–0.58)	0.79 (0.76–0.81)	1.17 (1.14–1.21)	0.82 (0.79–0.85)	0.9 (0.9–0.9)	0.96 (0.93–0.98)	1.26 (1.14–1.38)	1.08 (0.99–1.18)	0.09 (0.08–0.11)
22.86	0.58 (0.57–0.59)	0.79 (0.77–0.81)	1.17 (1.14–1.2)	0.83 (0.8–0.86)	0.9 (0.9–0.9)	0.95 (0.93–0.97)	1.24 (1.13–1.36)	1.07 (0.99–1.17)	0.1 (0.08–0.12)
23.03	0.58 (0.58–0.59)	0.79 (0.77–0.81)	1.17 (1.14–1.2)	0.84 (0.81–0.86)	0.9 (0.9–0.9)	0.94 (0.92–0.96)	1.22 (1.12–1.33)	1.07 (0.99–1.16)	0.11 (0.09–0.13)
23.20	0.59 (0.58–0.6)	0.8 (0.78–0.82)	1.17 (1.14–1.2)	0.85 (0.82–0.87)	0.9 (0.9–0.9)	0.94 (0.92–0.96)	1.2 (1.1–1.31)	1.06 (0.99–1.15)	0.12 (0.1–0.14)
23.37	0.6 (0.59–0.61)	0.8 (0.78–0.82)	1.16 (1.13–1.19)	0.85 (0.83–0.88)	0.9 (0.9–0.9)	0.93 (0.91–0.95)	1.18 (1.09–1.29)	1.06 (0.99–1.14)	0.13 (0.11–0.15)

23.54	0.61 (0.6–0.62)	0.81 (0.79–0.82)	1.16 (1.13–1.19)	0.86 (0.84–0.89)	0.9 (0.9–0.9)	0.93 (0.91–0.94)	1.17 (1.08–1.26)	1.06 (0.98–1.13)	0.14 (0.12–0.16)
23.71	0.62 (0.61–0.62)	0.81 (0.79–0.83)	1.16 (1.13–1.18)	0.87 (0.85–0.89)	0.9 (0.9–0.9)	0.92 (0.9–0.94)	1.15 (1.07–1.24)	1.05 (0.98–1.12)	0.15 (0.13–0.17)
23.88	0.63 (0.62–0.63)	0.82 (0.8–0.83)	1.15 (1.13–1.18)	0.88 (0.86–0.9)	0.9 (0.9–0.9)	0.92 (0.9–0.93)	1.14 (1.06–1.23)	1.05 (0.98–1.12)	0.17 (0.15–0.18)
24.06	0.63 (0.63–0.64)	0.82 (0.81–0.84)	1.15 (1.12–1.17)	0.89 (0.87–0.91)	0.9 (0.8–0.9)	0.91 (0.9–0.93)	1.12 (1.04–1.21)	1.04 (0.98–1.11)	0.18 (0.16–0.2)
24.23	0.64 (0.64–0.65)	0.83 (0.81–0.84)	1.14 (1.12–1.17)	0.9 (0.88–0.92)	0.9 (0.8–0.9)	0.91 (0.89–0.92)	1.11 (1.03–1.19)	1.04 (0.98–1.1)	0.2 (0.18–0.21)
24.40	0.65 (0.65–0.66)	0.83 (0.82–0.85)	1.14 (1.12–1.16)	0.9 (0.89–0.92)	0.9 (0.8–0.9)	0.91 (0.89–0.92)	1.1 (1.02–1.17)	1.04 (0.98–1.1)	0.21 (0.19–0.23)
24.57	0.66 (0.66–0.67)	0.84 (0.82–0.85)	1.14 (1.11–1.16)	0.91 (0.89–0.93)	0.9 (0.8–0.9)	0.9 (0.89–0.92)	1.08 (1.01–1.16)	1.03 (0.98–1.09)	0.23 (0.21–0.25)
24.74	0.67 (0.67–0.68)	0.84 (0.83–0.86)	1.13 (1.11–1.15)	0.92 (0.9–0.94)	0.9 (0.8–0.9)	0.9 (0.89–0.91)	1.07 (1–1.15)	1.03 (0.97–1.09)	0.25 (0.23–0.27)
24.91	0.69 (0.68–0.69)	0.85 (0.83–0.86)	1.13 (1.1–1.15)	0.93 (0.91–0.95)	0.9 (0.8–0.9)	0.9 (0.89–0.91)	1.06 (1–1.13)	1.02 (0.97–1.08)	0.27 (0.25–0.29)
25.08	0.7 (0.69–0.7)	0.85 (0.84–0.87)	1.12 (1.1–1.14)	0.93 (0.92–0.95)	0.9 (0.8–0.9)	0.9 (0.89–0.91)	1.05 (0.99–1.12)	1.02 (0.97–1.08)	0.29 (0.28–0.31)
25.25	0.71 (0.7–0.72)	0.86 (0.85–0.87)	1.12 (1.1–1.14)	0.94 (0.92–0.96)	0.9 (0.9–0.9)	0.9 (0.89–0.91)	1.04 (0.98–1.11)	1.02 (0.97–1.07)	0.32 (0.3–0.33)
25.42	0.72 (0.72–0.73)	0.87 (0.85–0.88)	1.11 (1.09–1.13)	0.95 (0.93–0.96)	0.9 (0.9–0.9)	0.9 (0.89–0.91)	1.03 (0.97–1.1)	1.02 (0.97–1.07)	0.34 (0.33–0.36)
25.59	0.73 (0.73–0.74)	0.87 (0.86–0.88)	1.1 (1.09–1.12)	0.95 (0.94–0.97)	0.9 (0.9–0.9)	0.9 (0.89–0.91)	1.03 (0.97–1.09)	1.01 (0.97–1.06)	0.37 (0.36–0.39)
25.76	0.75 (0.74–0.75)	0.88 (0.87–0.89)	1.1 (1.08–1.12)	0.96 (0.95–0.98)	0.9 (0.9–0.9)	0.9 (0.89–0.91)	1.02 (0.96–1.08)	1.01 (0.97–1.06)	0.4 (0.39–0.42)
25.93	0.76 (0.76–0.77)	0.89 (0.88–0.9)	1.09 (1.08–1.11)	0.97 (0.95–0.98)	0.9 (0.9–0.9)	0.9 (0.89–0.91)	1.01 (0.96–1.07)	1.01 (0.97–1.05)	0.43 (0.42–0.45)
26.11	0.78 (0.77–0.78)	0.89 (0.88–0.9)	1.09 (1.07–1.1)	0.97 (0.96–0.99)	0.9 (0.9–0.9)	0.91 (0.9–0.92)	1.01 (0.96–1.06)	1.01 (0.97–1.05)	0.47 (0.45–0.48)
26.28	0.79 (0.79–0.8)	0.9 (0.89–0.91)	1.08 (1.07–1.09)	0.98 (0.96–0.99)	0.9 (0.9–0.9)	0.91 (0.9–0.92)	1 (0.96–1.05)	1.01 (0.97–1.04)	0.5 (0.49–0.52)
26.45	0.81 (0.8–0.81)	0.91 (0.9–0.92)	1.07 (1.06–1.09)	0.98 (0.97–0.99)	0.9 (0.9–0.9)	0.92 (0.91–0.92)	1 (0.95–1.04)	1 (0.97–1.04)	0.54 (0.53–0.55)
26.62	0.83 (0.82–0.83)	0.92 (0.91–0.93)	1.07 (1.05–1.08)	0.98 (0.97–1)	0.9 (0.9–0.9)	0.92 (0.91–0.93)	0.99 (0.95–1.04)	1 (0.97–1.04)	0.58 (0.57–0.59)
26.79	0.85 (0.84–0.85)	0.93 (0.92–0.94)	1.06 (1.05–1.07)	0.99 (0.98–1)	0.9 (0.9–0.9)	0.93 (0.92–0.93)	0.99 (0.96–1.03)	1 (0.97–1.03)	0.62 (0.61–0.63)
26.96	0.86 (0.86–0.87)	0.94 (0.93–0.94)	1.05 (1.04–1.06)	0.99 (0.98–1)	0.9 (0.9–0.9)	0.94 (0.93–0.94)	0.99 (0.96–1.02)	1 (0.97–1.03)	0.67 (0.66–0.68)
27.13	0.88 (0.88–0.89)	0.95 (0.94–0.95)	1.04 (1.03–1.05)	0.99 (0.99–1)	0.9 (0.9–0.9)	0.94 (0.94–0.95)	0.99 (0.96–1.02)	1 (0.98–1.02)	0.72 (0.71–0.73)
27.30	0.9 (0.9–0.91)	0.96 (0.95–0.96)	1.03 (1.03–1.04)	1 (0.99–1)	0.9 (0.9–1)	0.95 (0.95–0.96)	0.99 (0.97–1.01)	1 (0.98–1.02)	0.77 (0.76–0.77)
27.47	0.93 (0.92–0.93)	0.97 (0.96–0.97)	1.03 (1.02–1.03)	1 (0.99–1)	1 (1–1)	0.96 (0.96–0.97)	0.99 (0.97–1.01)	1 (0.98–1.01)	0.82 (0.81–0.83)
27.64	0.95 (0.95–0.95)	0.98 (0.97–0.98)	1.02 (1.01–1.02)	1 (1–1)	1 (1–1)	0.97 (0.97–0.98)	0.99 (0.98–1.01)	1 (0.99–1.01)	0.88 (0.87–0.88)
27.81	0.97 (0.97–0.97)	0.99 (0.99–0.99)	1.01 (1.01–1.01)	1 (1–1)	1 (1–1)	0.99 (0.98–0.99)	1 (0.99–1)	1 (0.99–1)	0.93 (0.93–0.94)
27.98	1 (1–1)	1 (1–1)	1 (1–1)	1 (1–1)	1 (1–1)	1 (1–1)	1 (1–1)	1 (1–1)	0.99 (0.99–0.99)

**28.00**	**Reference**	**Reference**	**Reference**	**Reference**	**Reference**	**Reference**	**Reference**	**Reference**	**Reference**
28.16	1.02 (1.02–1.02)	1.01 (1.01–1.01)	0.99 (0.99–0.99)	1 (1–1)	1 (1–1)	1.01 (1.01–1.01)	1 (1–1.01)	1 (1–1.01)	1.06 (1.05–1.06)
28.33	1.05 (1.05–1.05)	1.02 (1.02–1.02)	0.98 (0.98–0.99)	1 (1–1)	1 (1–1)	1.03 (1.02–1.03)	1.01 (1–1.02)	1 (0.99–1.01)	1.12 (1.12–1.13)
28.50	1.08 (1.07–1.08)	1.03 (1.03–1.04)	0.97 (0.97–0.98)	1 (0.99–1.01)	1 (1–1.1)	1.04 (1.04–1.04)	1.01 (0.99–1.03)	1 (0.99–1.02)	1.19 (1.18–1.2)
28.67	1.1 (1.1–1.11)	1.05 (1.04–1.05)	0.97 (0.96–0.97)	1 (0.99–1.01)	1.1 (1.1–1.1)	1.06 (1.05–1.06)	1.02 (0.99–1.04)	1 (0.98–1.03)	1.26 (1.25–1.27)
28.84	1.13 (1.13–1.14)	1.06 (1.05–1.07)	0.96 (0.95–0.97)	1 (0.99–1.01)	1.1 (1.1–1.1)	1.07 (1.06–1.08)	1.02 (0.99–1.06)	1.01 (0.98–1.03)	1.33 (1.31–1.35)
29.01	1.16 (1.15–1.17)	1.07 (1.06–1.08)	0.95 (0.94–0.96)	1 (0.99–1.01)	1.1 (1.1–1.1)	1.09 (1.08–1.1)	1.03 (0.99–1.07)	1.01 (0.98–1.04)	1.4 (1.38–1.42)
29.18	1.19 (1.18–1.2)	1.08 (1.07–1.09)	0.94 (0.93–0.95)	1 (0.98–1.01)	1.1 (1.1–1.1)	1.1 (1.09–1.11)	1.04 (0.99–1.08)	1.01 (0.97–1.05)	1.47 (1.45–1.5)
29.35	1.22 (1.21–1.23)	1.09 (1.08–1.11)	0.93 (0.92–0.95)	0.99 (0.98–1.01)	1.1 (1.1–1.2)	1.12 (1.11–1.13)	1.04 (0.99–1.1)	1.01 (0.97–1.06)	1.55 (1.51–1.58)
29.52	1.25 (1.24–1.26)	1.11 (1.09–1.12)	0.93 (0.91–0.94)	0.99 (0.98–1.01)	1.2 (1.1–1.2)	1.14 (1.12–1.15)	1.05 (0.99–1.11)	1.02 (0.97–1.06)	1.62 (1.58–1.66)
29.69	1.28 (1.27–1.29)	1.12 (1.1–1.13)	0.92 (0.9–0.94)	0.99 (0.97–1.01)	1.2 (1.1–1.2)	1.15 (1.14–1.17)	1.06 (1–1.13)	1.02 (0.97–1.07)	1.69 (1.65–1.74)
29.86	1.31 (1.3–1.32)	1.13 (1.11–1.15)	0.91 (0.9–0.93)	0.99 (0.97–1.01)	1.2 (1.2–1.2)	1.17 (1.15–1.19)	1.07 (1–1.14)	1.02 (0.97–1.08)	1.77 (1.72–1.82)
30.04	1.34 (1.32–1.35)	1.14 (1.12–1.16)	0.91 (0.89–0.93)	0.99 (0.97–1.01)	1.2 (1.2–1.2)	1.18 (1.17–1.2)	1.07 (1–1.15)	1.03 (0.97–1.09)	1.84 (1.78–1.9)
30.21	1.37 (1.35–1.38)	1.15 (1.13–1.17)	0.9 (0.88–0.92)	0.99 (0.97–1.01)	1.2 (1.2–1.3)	1.2 (1.18–1.22)	1.08 (1–1.17)	1.03 (0.97–1.1)	1.91 (1.84–1.98)
30.38	1.4 (1.38–1.41)	1.16 (1.14–1.19)	0.89 (0.87–0.92)	0.99 (0.96–1.01)	1.2 (1.2–1.3)	1.22 (1.2–1.24)	1.09 (1–1.18)	1.03 (0.97–1.11)	1.98 (1.91–2.05)
30.55	1.42 (1.41–1.44)	1.18 (1.15–1.2)	0.89 (0.87–0.91)	0.99 (0.96–1.01)	1.2 (1.2–1.3)	1.23 (1.21–1.25)	1.1 (1.01–1.19)	1.04 (0.97–1.11)	2.04 (1.97–2.12)
30.72	1.45 (1.44–1.47)	1.19 (1.16–1.21)	0.88 (0.86–0.91)	0.99 (0.96–1.01)	1.3 (1.2–1.3)	1.25 (1.22–1.27)	1.1 (1.01–1.21)	1.04 (0.97–1.12)	2.11 (2.02–2.19)
30.89	1.48 (1.46–1.5)	1.2 (1.17–1.22)	0.88 (0.86–0.9)	0.98 (0.96–1.01)	1.3 (1.2–1.3)	1.26 (1.24–1.29)	1.11 (1.02–1.22)	1.05 (0.97–1.13)	2.17 (2.08–2.26)
31.06	1.51 (1.49–1.53)	1.21 (1.18–1.23)	0.88 (0.85–0.9)	0.98 (0.96–1.01)	1.3 (1.2–1.3)	1.27 (1.25–1.3)	1.12 (1.02–1.23)	1.05 (0.98–1.14)	2.23 (2.13–2.32)
31.23	1.54 (1.52–1.56)	1.22 (1.19–1.25)	0.87 (0.85–0.9)	0.98 (0.95–1.01)	1.3 (1.3–1.4)	1.29 (1.26–1.32)	1.13 (1.02–1.24)	1.06 (0.98–1.15)	2.28 (2.18–2.38)
31.40	1.56 (1.54–1.58)	1.23 (1.2–1.26)	0.87 (0.84–0.89)	0.98 (0.95–1.01)	1.3 (1.3–1.4)	1.3 (1.27–1.33)	1.14 (1.03–1.25)	1.07 (0.98–1.16)	2.33 (2.23–2.44)
31.57	1.59 (1.57–1.61)	1.24 (1.21–1.27)	0.86 (0.84–0.89)	0.98 (0.95–1.01)	1.3 (1.3–1.4)	1.32 (1.29–1.34)	1.14 (1.03–1.26)	1.07 (0.99–1.16)	2.38 (2.27–2.5)
31.74	1.62 (1.59–1.64)	1.25 (1.22–1.28)	0.86 (0.84–0.89)	0.98 (0.95–1.01)	1.3 (1.3–1.4)	1.33 (1.3–1.36)	1.15 (1.04–1.28)	1.08 (0.99–1.17)	2.43 (2.31–2.54)
31.91	1.64 (1.62–1.67)	1.26 (1.23–1.29)	0.86 (0.83–0.88)	0.98 (0.95–1.01)	1.3 (1.3–1.4)	1.34 (1.31–1.37)	1.16 (1.05–1.29)	1.08 (1–1.18)	2.47 (2.35–2.59)
32.09	1.67 (1.64–1.69)	1.27 (1.23–1.3)	0.85 (0.83–0.88)	0.98 (0.95–1.01)	1.4 (1.3–1.4)	1.35 (1.32–1.38)	1.17 (1.05–1.3)	1.09 (1–1.19)	2.51 (2.38–2.63)
32.26	1.69 (1.67–1.72)	1.27 (1.24–1.31)	0.85 (0.83–0.88)	0.98 (0.95–1.01)	1.4 (1.3–1.4)	1.37 (1.33–1.4)	1.18 (1.06–1.31)	1.1 (1.01–1.2)	2.54 (2.42–2.67)
32.43	1.72 (1.69–1.74)	1.28 (1.25–1.32)	0.85 (0.82–0.88)	0.98 (0.95–1.01)	1.4 (1.3–1.4)	1.38 (1.35–1.41)	1.18 (1.06–1.32)	1.11 (1.01–1.21)	2.57 (2.44–2.7)

32.60	1.74 (1.72–1.77)	1.29 (1.26–1.33)	0.85 (0.82–0.87)	0.98 (0.95–1.01)	1.4 (1.3–1.4)	1.39 (1.36–1.42)	1.19 (1.07–1.33)	1.11 (1.02–1.22)	2.6 (2.47–2.73)
32.77	1.76 (1.74–1.79)	1.3 (1.27–1.33)	0.85 (0.82–0.87)	0.98 (0.95–1.01)	1.4 (1.3–1.5)	1.4 (1.37–1.43)	1.2 (1.08–1.34)	1.12 (1.03–1.23)	2.62 (2.49–2.76)
32.94	1.79 (1.76–1.82)	1.31 (1.27–1.34)	0.84 (0.82–0.87)	0.98 (0.94–1.01)	1.4 (1.3–1.5)	1.41 (1.38–1.44)	1.21 (1.08–1.35)	1.13 (1.03–1.24)	2.64 (2.51–2.78)
33.11	1.81 (1.78–1.84)	1.32 (1.28–1.35)	0.84 (0.82–0.87)	0.97 (0.94–1.01)	1.4 (1.3–1.5)	1.42 (1.39–1.45)	1.22 (1.09–1.36)	1.14 (1.04–1.25)	2.66 (2.52–2.8)
33.28	1.83 (1.81–1.86)	1.32 (1.29–1.36)	0.84 (0.81–0.87)	0.97 (0.94–1.01)	1.4 (1.4–1.5)	1.43 (1.4–1.47)	1.22 (1.1–1.37)	1.15 (1.05–1.25)	2.67 (2.53–2.81)
33.45	1.86 (1.83–1.88)	1.33 (1.3–1.37)	0.84 (0.81–0.87)	0.97 (0.94–1.01)	1.4 (1.4–1.5)	1.44 (1.41–1.48)	1.23 (1.1–1.38)	1.16 (1.06–1.26)	2.68 (2.54–2.82)
33.62	1.88 (1.85–1.91)	1.34 (1.3–1.37)	0.84 (0.81–0.87)	0.97 (0.94–1.01)	1.4 (1.4–1.5)	1.45 (1.42–1.49)	1.24 (1.11–1.39)	1.16 (1.06–1.28)	2.68 (2.55–2.83)
33.79	1.9 (1.87–1.93)	1.35 (1.31–1.38)	0.84 (0.81–0.87)	0.97 (0.94–1.01)	1.4 (1.4–1.5)	1.46 (1.43–1.5)	1.25 (1.12–1.4)	1.17 (1.07–1.29)	2.69 (2.55–2.83)
33.96	1.92 (1.89–1.95)	1.35 (1.32–1.39)	0.84 (0.81–0.87)	0.97 (0.94–1.01)	1.4 (1.4–1.5)	1.47 (1.43–1.51)	1.26 (1.13–1.41)	1.18 (1.08–1.3)	2.69 (2.55–2.83)
34.14	1.94 (1.91–1.97)	1.36 (1.33–1.4)	0.84 (0.81–0.86)	0.97 (0.94–1.01)	1.4 (1.4–1.5)	1.48 (1.44–1.51)	1.27 (1.13–1.42)	1.19 (1.09–1.31)	2.68 (2.55–2.83)
34.31	1.96 (1.93–1.99)	1.37 (1.33–1.4)	0.84 (0.81–0.86)	0.97 (0.94–1.01)	1.4 (1.4–1.5)	1.49 (1.45–1.52)	1.28 (1.14–1.43)	1.2 (1.1–1.32)	2.68 (2.54–2.82)
34.48	1.98 (1.95–2.01)	1.37 (1.34–1.41)	0.84 (0.81–0.86)	0.97 (0.94–1.01)	1.4 (1.4–1.5)	1.5 (1.46–1.53)	1.28 (1.15–1.44)	1.21 (1.11–1.33)	2.67 (2.54–2.81)
34.65	2 (1.97–2.03)	1.38 (1.34–1.42)	0.84 (0.81–0.86)	0.97 (0.94–1.01)	1.4 (1.4–1.5)	1.5 (1.47–1.54)	1.29 (1.15–1.45)	1.22 (1.11–1.34)	2.66 (2.53–2.8)
34.82	2.02 (1.99–2.05)	1.39 (1.35–1.43)	0.84 (0.81–0.87)	0.97 (0.94–1.01)	1.4 (1.4–1.5)	1.51 (1.48–1.55)	1.3 (1.16–1.46)	1.23 (1.12–1.35)	2.65 (2.51–2.79)
34.99	2.04 (2.01–2.07)	1.39 (1.36–1.43)	0.84 (0.81–0.87)	0.97 (0.94–1.01)	1.4 (1.4–1.5)	1.52 (1.48–1.56)	1.31 (1.17–1.47)	1.24 (1.13–1.37)	2.63 (2.5–2.78)
35.16	2.06 (2.02–2.09)	1.4 (1.36–1.44)	0.84 (0.81–0.87)	0.97 (0.94–1.01)	1.4 (1.4–1.5)	1.53 (1.49–1.57)	1.32 (1.17–1.49)	1.25 (1.14–1.38)	2.62 (2.49–2.76)
35.33	2.08 (2.04–2.11)	1.41 (1.37–1.45)	0.84 (0.81–0.87)	0.97 (0.94–1.01)	1.5 (1.4–1.5)	1.54 (1.5–1.58)	1.33 (1.18–1.5)	1.27 (1.15–1.39)	2.6 (2.47–2.74)
35.50	2.1 (2.06–2.13)	1.41 (1.37–1.45)	0.84 (0.8–0.87)	0.97 (0.94–1.01)	1.5 (1.4–1.5)	1.54 (1.51–1.58)	1.34 (1.18–1.51)	1.28 (1.16–1.41)	2.58 (2.45–2.72)
35.67	2.12 (2.08–2.15)	1.42 (1.38–1.46)	0.84 (0.8–0.87)	0.97 (0.93–1.01)	1.5 (1.4–1.5)	1.55 (1.51–1.59)	1.35 (1.19–1.52)	1.29 (1.17–1.42)	2.56 (2.43–2.7)
35.84	2.13 (2.1–2.17)	1.43 (1.38–1.47)	0.84 (0.8–0.87)	0.97 (0.93–1.01)	1.5 (1.4–1.5)	1.56 (1.52–1.6)	1.36 (1.2–1.54)	1.3 (1.18–1.44)	2.54 (2.41–2.68)
36.02	2.15 (2.11–2.19)	1.43 (1.39–1.47)	0.84 (0.8–0.87)	0.97 (0.93–1.01)	1.5 (1.4–1.5)	1.57 (1.53–1.61)	1.37 (1.2–1.55)	1.31 (1.18–1.45)	2.52 (2.39–2.66)
36.19	2.17 (2.13–2.21)	1.44 (1.39–1.48)	0.84 (0.8–0.87)	0.97 (0.93–1.01)	1.5 (1.4–1.5)	1.57 (1.53–1.62)	1.37 (1.21–1.57)	1.32 (1.19–1.47)	2.5 (2.37–2.64)
36.36	2.19 (2.15–2.23)	1.44 (1.4–1.49)	0.84 (0.8–0.87)	0.97 (0.93–1.01)	1.5 (1.4–1.5)	1.58 (1.54–1.63)	1.38 (1.21–1.58)	1.34 (1.2–1.49)	2.48 (2.35–2.61)
36.53	2.21 (2.16–2.25)	1.45 (1.4–1.5)	0.84 (0.8–0.88)	0.97 (0.93–1.01)	1.5 (1.4–1.5)	1.59 (1.54–1.64)	1.39 (1.22–1.6)	1.35 (1.21–1.5)	2.45 (2.32–2.59)
36.70	2.23 (2.18–2.27)	1.46 (1.41–1.5)	0.84 (0.8–0.88)	0.97 (0.93–1.01)	1.5 (1.4–1.5)	1.6 (1.55–1.64)	1.4 (1.22–1.61)	1.36 (1.22–1.52)	2.43 (2.3–2.57)
36.87	2.25 (2.2–2.29)	1.46 (1.41–1.51)	0.84 (0.8–0.88)	0.97 (0.93–1.01)	1.5 (1.4–1.5)	1.6 (1.56–1.65)	1.41 (1.22–1.63)	1.37 (1.23–1.54)	2.41 (2.28–2.54)
37.04	2.26 (2.21–2.31)	1.47 (1.42–1.52)	0.84 (0.8–0.88)	0.97 (0.93–1.01)	1.5 (1.4–1.5)	1.61 (1.56–1.66)	1.42 (1.23–1.64)	1.38 (1.23–1.55)	2.38 (2.25–2.52)

37.21	2.28 (2.23–2.33)	1.47 (1.42–1.53)	0.84 (0.8–0.88)	0.97 (0.92–1.02)	1.5 (1.4–1.5)	1.62 (1.57–1.67)	1.43 (1.23–1.66)	1.4 (1.24–1.57)	2.36 (2.23–2.5)
37.38	2.3 (2.25–2.36)	1.48 (1.43–1.53)	0.84 (0.8–0.88)	0.97 (0.92–1.02)	1.5 (1.4–1.6)	1.63 (1.57–1.68)	1.44 (1.24–1.68)	1.41 (1.25–1.59)	2.34 (2.21–2.48)
37.55	2.32 (2.27–2.38)	1.49 (1.43–1.54)	0.84 (0.8–0.89)	0.97 (0.92–1.02)	1.5 (1.4–1.6)	1.63 (1.58–1.69)	1.45 (1.24–1.7)	1.42 (1.26–1.61)	2.32 (2.18–2.45)
37.72	2.34 (2.28–2.4)	1.49 (1.44–1.55)	0.84 (0.8–0.89)	0.97 (0.92–1.02)	1.5 (1.4–1.6)	1.64 (1.59–1.7)	1.46 (1.24–1.71)	1.44 (1.26–1.63)	2.29 (2.16–2.43)
37.89	2.36 (2.3–2.42)	1.5 (1.44–1.56)	0.84 (0.8–0.89)	0.97 (0.92–1.02)	1.5 (1.4–1.6)	1.65 (1.59–1.71)	1.47 (1.25–1.73)	1.45 (1.27–1.65)	2.27 (2.14–2.41)
38.07	2.38 (2.32–2.44)	1.5 (1.45–1.56)	0.84 (0.8–0.89)	0.97 (0.92–1.02)	1.5 (1.4–1.6)	1.66 (1.6–1.72)	1.48 (1.25–1.75)	1.46 (1.28–1.67)	2.25 (2.12–2.39)
38.24	2.4 (2.34–2.46)	1.51 (1.45–1.57)	0.84 (0.8–0.89)	0.97 (0.92–1.02)	1.5 (1.4–1.6)	1.66 (1.6–1.72)	1.49 (1.25–1.77)	1.48 (1.29–1.69)	2.23 (2.09–2.37)
38.41	2.42 (2.35–2.49)	1.52 (1.46–1.58)	0.84 (0.8–0.9)	0.97 (0.91–1.02)	1.5 (1.4–1.6)	1.67 (1.61–1.73)	1.5 (1.26–1.79)	1.49 (1.29–1.71)	2.21 (2.07–2.35)
38.58	2.44 (2.37–2.51)	1.52 (1.46–1.59)	0.84 (0.79–0.9)	0.97 (0.91–1.03)	1.5 (1.4–1.6)	1.68 (1.61–1.74)	1.51 (1.26–1.81)	1.5 (1.3–1.73)	2.18 (2.05–2.33)
38.75	2.46 (2.39–2.53)	1.53 (1.46–1.6)	0.85 (0.79–0.9)	0.97 (0.91–1.03)	1.5 (1.4–1.6)	1.69 (1.62–1.75)	1.52 (1.26–1.83)	1.52 (1.31–1.76)	2.16 (2.03–2.31)
38.92	2.48 (2.41–2.55)	1.54 (1.47–1.6)	0.85 (0.79–0.9)	0.97 (0.91–1.03)	1.5 (1.4–1.6)	1.69 (1.63–1.76)	1.53 (1.26–1.85)	1.53 (1.32–1.78)	2.14 (2–2.29)
39.09	2.5 (2.43–2.58)	1.54 (1.47–1.61)	0.85 (0.79–0.9)	0.97 (0.91–1.03)	1.5 (1.4–1.6)	1.7 (1.63–1.77)	1.54 (1.27–1.87)	1.54 (1.32–1.8)	2.12 (1.98–2.27)
39.26	2.52 (2.45–2.6)	1.55 (1.48–1.62)	0.85 (0.79–0.91)	0.97 (0.91–1.03)	1.5 (1.4–1.6)	1.71 (1.64–1.78)	1.55 (1.27–1.9)	1.56 (1.33–1.82)	2.1 (1.96–2.25)
39.43	2.54 (2.46–2.62)	1.55 (1.48–1.63)	0.85 (0.79–0.91)	0.97 (0.91–1.03)	1.5 (1.4–1.6)	1.72 (1.64–1.79)	1.56 (1.27–1.92)	1.57 (1.34–1.85)	2.08 (1.94–2.23)
39.60	2.56 (2.48–2.65)	1.56 (1.49–1.64)	0.85 (0.79–0.91)	0.97 (0.9–1.03)	1.5 (1.4–1.6)	1.72 (1.65–1.8)	1.57 (1.27–1.94)	1.59 (1.35–1.87)	2.06 (1.92–2.22)
39.77	2.59 (2.5–2.67)	1.57 (1.49–1.65)	0.85 (0.79–0.91)	0.97 (0.9–1.04)	1.5 (1.4–1.6)	1.73 (1.66–1.81)	1.58 (1.28–1.96)	1.6 (1.35–1.9)	2.04 (1.9–2.2)
39.94	2.61 (2.52–2.7)	1.57 (1.5–1.66)	0.85 (0.79–0.92)	0.97 (0.9–1.04)	1.5 (1.3–1.6)	1.74 (1.66–1.82)	1.59 (1.28–1.99)	1.62 (1.36–1.92)	2.02 (1.88–2.18)
40.12	2.63 (2.54–2.72)	1.58 (1.5–1.66)	0.85 (0.79–0.92)	0.97 (0.9–1.04)	1.5 (1.3–1.6)	1.75 (1.67–1.83)	1.6 (1.28–2.01)	1.63 (1.37–1.95)	2 (1.86–2.16)
40.29	2.65 (2.56–2.75)	1.59 (1.51–1.67)	0.85 (0.79–0.92)	0.97 (0.9–1.04)	1.5 (1.3–1.6)	1.76 (1.67–1.84)	1.62 (1.28–2.03)	1.65 (1.37–1.97)	1.98 (1.84–2.14)
40.46	2.67 (2.58–2.77)	1.59 (1.51–1.68)	0.85 (0.78–0.92)	0.97 (0.9–1.04)	1.5 (1.3–1.6)	1.76 (1.68–1.85)	1.63 (1.29–2.06)	1.66 (1.38–2)	1.96 (1.81–2.13)
40.63	2.69 (2.6–2.8)	1.6 (1.51–1.69)	0.85 (0.78–0.92)	0.97 (0.89–1.04)	1.5 (1.3–1.6)	1.77 (1.69–1.86)	1.64 (1.29–2.08)	1.68 (1.39–2.02)	1.95 (1.79–2.11)
40.80	2.72 (2.62–2.82)	1.61 (1.52–1.7)	0.85 (0.78–0.93)	0.97 (0.89–1.04)	1.5 (1.3–1.6)	1.78 (1.69–1.87)	1.65 (1.29–2.11)	1.69 (1.4–2.05)	1.93 (1.77–2.09)
40.97	2.74 (2.64–2.85)	1.61 (1.52–1.71)	0.85 (0.78–0.93)	0.97 (0.89–1.05)	1.5 (1.3–1.6)	1.79 (1.7–1.88)	1.66 (1.29–2.13)	1.71 (1.4–2.08)	1.91 (1.75–2.07)
41.14	2.76 (2.66–2.87)	1.62 (1.53–1.72)	0.85 (0.78–0.93)	0.97 (0.89–1.05)	1.5 (1.3–1.6)	1.8 (1.7–1.9)	1.67 (1.29–2.16)	1.72 (1.41–2.1)	1.89 (1.74–2.06)
41.31	2.79 (2.68–2.9)	1.63 (1.53–1.73)	0.85 (0.78–0.93)	0.97 (0.89–1.05)	1.5 (1.3–1.6)	1.8 (1.71–1.91)	1.68 (1.3–2.18)	1.74 (1.42–2.13)	1.87 (1.72–2.04)
41.48	2.81 (2.7–2.93)	1.63 (1.54–1.74)	0.85 (0.78–0.94)	0.97 (0.89–1.05)	1.5 (1.3–1.6)	1.81 (1.71–1.92)	1.69 (1.3–2.21)	1.75 (1.42–2.16)	1.85 (1.7–2.03)
41.65	2.83 (2.72–2.95)	1.64 (1.54–1.75)	0.85 (0.78–0.94)	0.97 (0.88–1.05)	1.5 (1.3–1.6)	1.82 (1.72–1.93)	1.71 (1.3–2.24)	1.77 (1.43–2.19)	1.84 (1.68–2.01)

41.82	2.86 (2.74–2.98)	1.65 (1.55–1.75)	0.85 (0.78–0.94)	0.96 (0.88–1.05)	1.5 (1.3–1.6)	1.83 (1.73–1.94)	1.72 (1.3–2.26)	1.79 (1.44–2.22)	1.82 (1.66–1.99)
41.99	2.88 (2.76–3.01)	1.65 (1.55–1.76)	0.86 (0.78–0.94)	0.96 (0.88–1.06)	1.5 (1.3–1.6)	1.84 (1.73–1.95)	1.73 (1.3–2.29)	1.8 (1.45–2.25)	1.8 (1.64–1.98)
42.17	2.9 (2.78–3.03)	1.66 (1.55–1.77)	0.86 (0.78–0.94)	0.96 (0.88–1.06)	1.5 (1.3–1.7)	1.85 (1.74–1.96)	1.74 (1.31–2.32)	1.82 (1.45–2.28)	1.78 (1.62–1.96)
42.34	2.93 (2.8–3.06)	1.67 (1.56–1.78)	0.86 (0.77–0.95)	0.96 (0.88–1.06)	1.5 (1.3–1.7)	1.85 (1.74–1.97)	1.75 (1.31–2.35)	1.84 (1.46–2.31)	1.77 (1.6–1.95)
42.51	2.95 (2.82–3.09)	1.67 (1.56–1.79)	0.86 (0.77–0.95)	0.96 (0.88–1.06)	1.5 (1.3–1.7)	1.86 (1.75–1.98)	1.76 (1.31–2.37)	1.85 (1.47–2.34)	1.75 (1.58–1.93)
42.68	2.98 (2.84–3.12)	1.68 (1.57–1.8)	0.86 (0.77–0.95)	0.96 (0.87–1.06)	1.5 (1.3–1.7)	1.87 (1.76–1.99)	1.78 (1.31–2.4)	1.87 (1.47–2.37)	1.73 (1.57–1.92)
42.85	3 (2.86–3.15)	1.69 (1.57–1.81)	0.86 (0.77–0.95)	0.96 (0.87–1.06)	1.5 (1.3–1.7)	1.88 (1.76–2.01)	1.79 (1.31–2.43)	1.89 (1.48–2.4)	1.72 (1.55–1.9)
43.02	3.03 (2.88–3.18)	1.69 (1.58–1.82)	0.86 (0.77–0.96)	0.96 (0.87–1.07)	1.5 (1.3–1.7)	1.89 (1.77–2.02)	1.8 (1.32–2.46)	1.9 (1.49–2.44)	1.7 (1.53–1.89)
43.19	3.05 (2.9–3.21)	1.7 (1.58–1.83)	0.86 (0.77–0.96)	0.96 (0.87–1.07)	1.5 (1.3–1.7)	1.9 (1.77–2.03)	1.81 (1.32–2.49)	1.92 (1.5–2.47)	1.68 (1.51–1.87)
43.36	3.08 (2.93–3.23)	1.71 (1.59–1.84)	0.86 (0.77–0.96)	0.96 (0.87–1.07)	1.5 (1.3–1.7)	1.91 (1.78–2.04)	1.82 (1.32–2.52)	1.94 (1.5–2.5)	1.67 (1.5–1.86)
43.53	3.1 (2.95–3.26)	1.72 (1.59–1.85)	0.86 (0.77–0.96)	0.96 (0.87–1.07)	1.5 (1.3–1.7)	1.91 (1.79–2.05)	1.84 (1.32–2.55)	1.96 (1.51–2.53)	1.65 (1.48–1.84)
43.70	3.13 (2.97–3.29)	1.72 (1.59–1.86)	0.86 (0.77–0.97)	0.96 (0.86–1.07)	1.5 (1.3–1.7)	1.92 (1.79–2.06)	1.85 (1.32–2.59)	1.97 (1.52–2.57)	1.63 (1.46–1.83)
43.87	3.15 (2.99–3.32)	1.73 (1.6–1.87)	0.86 (0.77–0.97)	0.96 (0.86–1.07)	1.5 (1.3–1.7)	1.93 (1.8–2.08)	1.86 (1.32–2.62)	1.99 (1.52–2.6)	1.62 (1.45–1.81)
44.05	3.18 (3.02–3.35)	1.74 (1.6–1.88)	0.86 (0.77–0.97)	0.96 (0.86–1.08)	1.5 (1.3–1.7)	1.94 (1.8–2.09)	1.87 (1.33–2.65)	2.01 (1.53–2.64)	1.6 (1.43–1.8)
44.22	3.21 (3.04–3.39)	1.74 (1.61–1.89)	0.86 (0.76–0.97)	0.96 (0.86–1.08)	1.5 (1.3–1.7)	1.95 (1.81–2.1)	1.89 (1.33–2.68)	2.03 (1.54–2.67)	1.59 (1.41–1.78)
44.39	3.23 (3.06–3.42)	1.75 (1.61–1.9)	0.86 (0.76–0.98)	0.96 (0.86–1.08)	1.5 (1.3–1.7)	1.96 (1.82–2.11)	1.9 (1.33–2.72)	2.05 (1.55–2.71)	1.57 (1.4–1.77)
44.56	3.26 (3.08–3.45)	1.76 (1.62–1.91)	0.86 (0.76–0.98)	0.96 (0.86–1.08)	1.5 (1.3–1.7)	1.97 (1.82–2.12)	1.91 (1.33–2.75)	2.07 (1.55–2.75)	1.56 (1.38–1.76)
44.73	3.29 (3.11–3.48)	1.77 (1.62–1.92)	0.86 (0.76–0.98)	0.96 (0.85–1.08)	1.5 (1.3–1.7)	1.98 (1.83–2.14)	1.93 (1.33–2.78)	2.08 (1.56–2.78)	1.54 (1.37–1.74)
44.90	3.32 (3.13–3.51)	1.77 (1.63–1.93)	0.86 (0.76–0.98)	0.96 (0.85–1.09)	1.5 (1.3–1.7)	1.99 (1.84–2.15)	1.94 (1.33–2.82)	2.1 (1.57–2.82)	1.53 (1.35–1.73)
45.07	3.34 (3.15–3.54)	1.78 (1.63–1.94)	0.87 (0.76–0.99)	0.96 (0.85–1.09)	1.5 (1.3–1.7)	1.99 (1.84–2.16)	1.95 (1.34–2.85)	2.12 (1.58–2.86)	1.51 (1.33–1.72)
45.24	3.37 (3.18–3.58)	1.79 (1.64–1.95)	0.87 (0.76–0.99)	0.96 (0.85–1.09)	1.5 (1.3–1.7)	2 (1.85–2.17)	1.96 (1.34–2.89)	2.14 (1.58–2.9)	1.5 (1.32–1.7)
45.41	3.4 (3.2–3.61)	1.79 (1.64–1.96)	0.87 (0.76–0.99)	0.96 (0.85–1.09)	1.5 (1.3–1.7)	2.01 (1.85–2.18)	1.98 (1.34–2.92)	2.16 (1.59–2.94)	1.48 (1.3–1.69)
45.58	3.43 (3.22–3.64)	1.8 (1.65–1.97)	0.87 (0.76–0.99)	0.96 (0.85–1.09)	1.5 (1.3–1.7)	2.02 (1.86–2.2)	1.99 (1.34–2.96)	2.18 (1.6–2.98)	1.47 (1.29–1.68)
45.75	3.46 (3.25–3.68)	1.81 (1.65–1.98)	0.87 (0.76–1)	0.96 (0.85–1.09)	1.5 (1.3–1.7)	2.03 (1.87–2.21)	2 (1.34–3)	2.2 (1.61–3.02)	1.46 (1.27–1.66)
45.92	3.48 (3.27–3.71)	1.82 (1.65–2)	0.87 (0.76–1)	0.96 (0.84–1.1)	1.5 (1.3–1.7)	2.04 (1.87–2.22)	2.02 (1.34–3.03)	2.22 (1.61–3.06)	1.44 (1.26–1.65)
46.10	3.51 (3.3–3.74)	1.82 (1.66–2.01)	0.87 (0.75–1)	0.96 (0.84–1.1)	1.5 (1.3–1.7)	2.05 (1.88–2.24)	2.03 (1.34–3.07)	2.24 (1.62–3.1)	1.43 (1.25–1.64)
46.27	3.54 (3.32–3.78)	1.83 (1.66–2.02)	0.87 (0.75–1)	0.96 (0.84–1.1)	1.5 (1.3–1.8)	2.06 (1.89–2.25)	2.05 (1.35–3.11)	2.26 (1.63–3.14)	1.41 (1.23–1.62)

46.44	3.57 (3.35–3.81)	1.84 (1.67–2.03)	0.87 (0.75–1.01)	0.96 (0.84–1.1)	1.5 (1.3–1.8)	2.07 (1.89–2.26)	2.06 (1.35–3.15)	2.28 (1.64–3.19)	1.4 (1.22–1.61)
46.61	3.6 (3.37–3.85)	1.85 (1.67–2.04)	0.87 (0.75–1.01)	0.96 (0.84–1.1)	1.5 (1.3–1.8)	2.08 (1.9–2.27)	2.07 (1.35–3.19)	2.3 (1.64–3.23)	1.39 (1.2–1.6)
46.78	3.63 (3.4–3.88)	1.85 (1.68–2.05)	0.87 (0.75–1.01)	0.96 (0.84–1.1)	1.5 (1.3–1.8)	2.09 (1.91–2.29)	2.09 (1.35–3.23)	2.33 (1.65–3.27)	1.37 (1.19–1.59)
46.95	3.66 (3.42–3.92)	1.86 (1.68–2.06)	0.87 (0.75–1.01)	0.96 (0.83–1.11)	1.5 (1.3–1.8)	2.1 (1.91–2.3)	2.1 (1.35–3.27)	2.35 (1.66–3.32)	1.36 (1.18–1.57)
47.12	3.69 (3.45–3.96)	1.87 (1.69–2.07)	0.87 (0.75–1.02)	0.96 (0.83–1.11)	1.5 (1.3–1.8)	2.11 (1.92–2.31)	2.12 (1.35–3.31)	2.37 (1.67–3.36)	1.35 (1.16–1.56)
47.29	3.72 (3.47–3.99)	1.88 (1.69–2.08)	0.87 (0.75–1.02)	0.96 (0.83–1.11)	1.5 (1.3–1.8)	2.12 (1.92–2.33)	2.13 (1.36–3.35)	2.39 (1.68–3.41)	1.33 (1.15–1.55)
47.46	3.75 (3.5–4.03)	1.89 (1.7–2.09)	0.87 (0.75–1.02)	0.96 (0.83–1.11)	1.5 (1.2–1.8)	2.13 (1.93–2.34)	2.14 (1.36–3.39)	2.41 (1.68–3.46)	1.32 (1.14–1.54)
47.63	3.79 (3.52–4.07)	1.89 (1.7–2.11)	0.87 (0.75–1.02)	0.96 (0.83–1.11)	1.5 (1.2–1.8)	2.14 (1.94–2.35)	2.16 (1.36–3.43)	2.43 (1.69–3.5)	1.31 (1.12–1.53)
47.80	3.82 (3.55–4.1)	1.9 (1.71–2.12)	0.87 (0.74–1.03)	0.96 (0.83–1.12)	1.5 (1.2–1.8)	2.15 (1.94–2.37)	2.17 (1.36–3.47)	2.46 (1.7–3.55)	1.3 (1.11–1.51)
47.97	3.85 (3.58–4.14)	1.91 (1.71–2.13)	0.87 (0.74–1.03)	0.96 (0.82–1.12)	1.5 (1.2–1.8)	2.15 (1.95–2.38)	2.19 (1.36–3.52)	2.48 (1.71–3.6)	1.28 (1.1–1.5)
48.15	3.88 (3.6–4.18)	1.92 (1.72–2.14)	0.88 (0.74–1.03)	0.96 (0.82–1.12)	1.5 (1.2–1.8)	2.16 (1.96–2.39)	2.2 (1.36–3.56)	2.5 (1.71–3.65)	1.27 (1.08–1.49)
48.32	3.91 (3.63–4.22)	1.92 (1.72–2.15)	0.88 (0.74–1.03)	0.96 (0.82–1.12)	1.5 (1.2–1.8)	2.17 (1.96–2.41)	2.22 (1.36–3.61)	2.52 (1.72–3.7)	1.26 (1.07–1.48)
48.49	3.95 (3.66–4.26)	1.93 (1.73–2.16)	0.88 (0.74–1.04)	0.96 (0.82–1.12)	1.5 (1.2–1.8)	2.18 (1.97–2.42)	2.23 (1.37–3.65)	2.55 (1.73–3.75)	1.25 (1.06–1.47)
48.66	3.98 (3.69–4.29)	1.94 (1.73–2.18)	0.88 (0.74–1.04)	0.96 (0.82–1.13)	1.5 (1.2–1.8)	2.19 (1.98–2.44)	2.25 (1.37–3.7)	2.57 (1.74–3.8)	1.23 (1.05–1.46)
48.83	4.01 (3.71–4.33)	1.95 (1.74–2.19)	0.88 (0.74–1.04)	0.96 (0.82–1.13)	1.5 (1.2–1.8)	2.2 (1.98–2.45)	2.26 (1.37–3.74)	2.59 (1.75–3.85)	1.22 (1.04–1.45)
49.00	4.05 (3.74–4.37)	1.96 (1.74–2.2)	0.88 (0.74–1.04)	0.96 (0.81–1.13)	1.5 (1.2–1.8)	2.21 (1.99–2.46)	2.28 (1.37–3.79)	2.62 (1.75–3.9)	1.21 (1.02–1.43)

Table S8 The difference of characteristics between ART and SC women

Characteristics	Group	ART (n=9,494)	SC (n=270,709)	*P*
Maternal age (years)		31.77±3.91	28.4±4.85	<0.001
Age group n (%)	<20	/	7036 (2.6)	<0.001
	20-24	160 (1.63)	46642 (17.23)	<0.001
	25-29	2601 (26.56)	117363 (43.35)	
	30-34	4668 (47.66)	68499 (25.3)	
	35-39	1729 (17.65)	26092 (9.64)	
	≥40	336 (3.43)	5073 (1.87)	
Gravidity n (%)	One	4236 (50.52)	59916 (37.2)	<0.001
	Two	2191 (26.13)	44244 (27.47)	
	Three or more	1958 (23.35)	56903 (35.33)	
Parity n (%)	Primipara	5112 (60.97)	89404 (55.63)	<0.001
	Multipara	3273 (39.03)	71304 (44.37)	
Twin or multiple gestations n (%)	Yes	5243 (53.53)	12415 (4.59)	<0.001
	No	4251 (43.4)	258294 (95.41)	

The differences of characteristics between ART and SC women were evaluated with unpaired Student's t-test and using the chi-square($\chi 2$) test.
ART, assisted reproductive technology; SC, spontaneous conception.

Table S9 The Stratification analysis of the ART intervention and twin or multiple gestations for the risk of pregnancy complications and adverse outcomes

Diseases	Stratification	OR (95% CI)	*P*
Hypothyroidism	SC & Singleton pregnancy	Reference	
	ART & Singleton pregnancy	1.36 (1.24–1.48)	<0.001
	SC & Twin or multiple gestations	1.2 (1.14–1.27)	<0.001
	ART & Twin or multiple gestations	1.71 (1.6–1.84)	<0.001
Hyperthyroidism	SC & Singleton pregnancy	Reference	
	ART & Singleton pregnancy	1.35 (0.85–2.13)	0.198
	SC & Twin or multiple gestations	1.98 (1.57–2.5)	<0.001
	ART & Twin or multiple gestations	2.63 (1.97–3.51)	<0.001
GDM	SC & Singleton pregnancy	Reference	
	ART & Singleton pregnancy	1.4 (1.29–1.52)	<0.001
	SC & Twin or multiple gestations	1.27 (1.21–1.34)	<0.001
	ART & Twin or multiple gestations	1.55 (1.45–1.66)	<0.001
HDP	SC & Singleton pregnancy	Reference	
	ART & Singleton pregnancy	1.44 (1.29–1.6)	<0.001
	SC & Twin or multiple gestations	2.83 (2.68–2.99)	<0.001
	ART & Twin or multiple gestations	2.72 (2.52–2.94)	<0.001
Thrombocytopenia	SC & Singleton pregnancy	Reference	
	ART & Singleton pregnancy	1.29 (1.07–1.57)	0.009
	SC & Twin or multiple gestations	2.16 (1.97–2.36)	<0.001
	ART & Twin or multiple gestations	1.86 (1.62–2.13)	<0.001
Anemia	SC & Singleton pregnancy	Reference	
	ART & Singleton pregnancy	1.4 (1.3–1.52)	<0.001
	SC & Twin or multiple gestations	1.46 (1.4–1.52)	<0.001

	ART & Twin or multiple gestations	1.78 (1.68–1.89)	<0.001
Polyhydramnios	SC & Singleton pregnancy	Reference	
	ART & Singleton pregnancy	1.36 (1.09–1.68)	0.005
	SC & Twin or multiple gestations	2.23 (2.02–2.47)	<0.001
	ART & Twin or multiple gestations	2.26 (1.96–2.6)	<0.001
Oligohydramnios	SC & Singleton pregnancy	Reference	
	ART & Singleton pregnancy	1.04 (0.91–1.18)	0.599
	SC & Twin or multiple gestations	0.24 (0.21–0.28)	<0.001
	ART & Twin or multiple gestations	0.17 (0.13–0.22)	<0.001
PROM	SC & Singleton pregnancy	Reference	
	ART & Singleton pregnancy	0.74 (0.68–0.81)	<0.001
	SC & Twin or multiple gestations	1.07 (1.02–1.13)	0.005
	ART & Twin or multiple gestations	0.74 (0.69–0.8)	<0.001
Placenta previa	SC & Singleton pregnancy	Reference	
	ART & Singleton pregnancy	1.35 (1.16–1.57)	<0.001
	SC & Twin or multiple gestations	1.48 (1.35–1.62)	<0.001
	ART & Twin or multiple gestations	1.64 (1.44–1.85)	<0.001
Placental abruption	SC & Singleton pregnancy	Reference	
	ART & Singleton pregnancy	1.01 (0.79–1.28)	0.933
	SC & Twin or multiple gestations	1.44 (1.29–1.62)	<0.001
	ART & Twin or multiple gestations	1.09 (0.91–1.32)	0.350
Cesarean section	SC & Singleton pregnancy	Reference	
	ART & Singleton pregnancy	2.24 (2.08–2.42)	<0.001
	SC & Twin or multiple gestations	10.88 (10.25–11.55)	<0.001
	ART & Twin or multiple gestations	23.71 (20.77–27.08)	<0.001
Perinatal asphyxia	SC & Singleton pregnancy	Reference	

	ART & Singleton pregnancy	1 (0.86–1.17)	0.954
	SC & Twin or multiple gestations	1 (0.91–1.1)	0.958
	ART & Twin or multiple gestations	0.73 (0.63–0.85)	<0.001
LGA	SC & Singleton pregnancy	Reference	
	ART & Singleton pregnancy	0.97 (0.84–1.13)	0.727
	SC & Twin or multiple gestations	0 (0–0.01)	<0.001
	ART & Twin or multiple gestations	0.02 (0.01–0.04)	<0.001
SGA	SC & Singleton pregnancy	Reference	
	ART & Singleton pregnancy	1.12 (0.93–1.34)	0.246
	SC & Twin or multiple gestations	10.7 (10.16–11.28)	<0.001
	ART & Twin or multiple gestations	12.01 (11.19–12.89)	<0.001
Postpartum hemorrhage	SC & Singleton pregnancy	Reference	
	ART & Singleton pregnancy	2.05 (1.87–2.25)	<0.001
	SC & Twin or multiple gestations	4.2 (4.02–4.4)	<0.001
	ART & Twin or multiple gestations	6.26 (5.88–6.65)	<0.001
Stillbirth	SC & Singleton pregnancy	Reference	
	ART & Singleton pregnancy	0.63 (0.31–1.27)	0.199
	SC & Twin or multiple gestations	5.6 (4.8–6.55)	<0.001
	ART & Twin or multiple gestations	2.19 (1.57–3.06)	<0.001
Premature	SC & Singleton pregnancy	Reference	
	ART & Singleton pregnancy	1.24 (1.12-1.38)	<0.001
	SC & Twin or multiple gestations	17.47 (16.73-18.24)	<0.001
	ART & Twin or multiple gestations	14.39 (13.54-15.30)	<0.001
Congenital malformation	SC & Singleton pregnancy	Reference	
	ART & Singleton pregnancy	0.95 (0.63–1.44)	0.820
	SC & Twin or multiple gestations	1.18 (0.95–1.47)	0.145

ART & Twin or multiple gestations	1.06 (0.75–1.49)	0.741

ART, assisted reproductive technology; GDM, gestational diabetes mellitus; HDP, hypertensive disorders in pregnancy; PROM, premature rupture of membranes; LGA, Large-for-gestational-age; SGA, small-for-gestational-age.

The maternal age, gravidity and parity were adjusted.

Table S10 The risk of different maternal age (15-49 years) for pregnancy complications and adverse perinatal outcomes in ART and SC women with singleton pregnancy (n=262,466)

Age	GDM		PHD		Hypothyroidism		Anemia		Thrombocytopenia	
	SC	ART	SC	ART	SC	ART	SC	ART	SC	ART
15.00	0.13 (0.12–0.14)	0.21 (0.19–0.24)	1.33 (1.2–1.46)	2.31 (2–2.67)	0.4 (0.37–0.44)	0.82 (0.73–0.93)	1.51 (1.44–1.59)	1.88 (1.72–2.05)	0.33 (0.28–0.4)	0.5 (0.39–0.63)
15.17	0.13 (0.12–0.14)	0.22 (0.2–0.25)	1.32 (1.2–1.45)	2.3 (2–2.65)	0.4 (0.37–0.44)	0.83 (0.74–0.94)	1.5 (1.43–1.58)	1.87 (1.71–2.04)	0.34 (0.28–0.4)	0.5 (0.39–0.64)
15.34	0.14 (0.12–0.15)	0.23 (0.2–0.25)	1.31 (1.2–1.44)	2.29 (1.99–2.64)	0.41 (0.38–0.45)	0.84 (0.75–0.95)	1.49 (1.42–1.57)	1.86 (1.7–2.03)	0.34 (0.29–0.41)	0.51 (0.4–0.65)
15.51	0.14 (0.13–0.15)	0.23 (0.21–0.26)	1.31 (1.19–1.43)	2.28 (1.98–2.62)	0.41 (0.38–0.45)	0.85 (0.76–0.96)	1.48 (1.41–1.56)	1.84 (1.69–2.01)	0.35 (0.29–0.41)	0.52 (0.41–0.66)
15.68	0.14 (0.13–0.16)	0.24 (0.21–0.27)	1.3 (1.19–1.42)	2.27 (1.97–2.61)	0.42 (0.39–0.46)	0.86 (0.77–0.97)	1.47 (1.4–1.55)	1.83 (1.68–2)	0.35 (0.3–0.42)	0.53 (0.42–0.67)
15.85	0.15 (0.14–0.16)	0.25 (0.22–0.28)	1.29 (1.18–1.41)	2.26 (1.96–2.59)	0.42 (0.39–0.46)	0.87 (0.78–0.98)	1.47 (1.4–1.54)	1.82 (1.67–1.99)	0.36 (0.3–0.42)	0.53 (0.42–0.68)
16.03	0.15 (0.14–0.16)	0.25 (0.23–0.28)	1.29 (1.18–1.41)	2.25 (1.96–2.58)	0.43 (0.4–0.47)	0.88 (0.79–0.99)	1.46 (1.39–1.53)	1.81 (1.66–1.98)	0.36 (0.31–0.43)	0.54 (0.43–0.69)
16.20	0.16 (0.14–0.17)	0.26 (0.23–0.29)	1.28 (1.17–1.4)	2.23 (1.95–2.56)	0.44 (0.4–0.47)	0.9 (0.8–1.01)	1.45 (1.38–1.52)	1.8 (1.65–1.96)	0.37 (0.31–0.43)	0.55 (0.44–0.7)
16.37	0.16 (0.15–0.17)	0.27 (0.24–0.3)	1.27 (1.17–1.39)	2.22 (1.94–2.55)	0.44 (0.41–0.48)	0.91 (0.81–1.02)	1.44 (1.38–1.51)	1.79 (1.64–1.95)	0.37 (0.32–0.44)	0.56 (0.44–0.71)
16.54	0.17 (0.15–0.18)	0.27 (0.25–0.31)	1.27 (1.17–1.38)	2.21 (1.93–2.53)	0.45 (0.41–0.48)	0.92 (0.82–1.03)	1.43 (1.37–1.5)	1.78 (1.63–1.94)	0.38 (0.33–0.44)	0.57 (0.45–0.71)
16.71	0.17 (0.16–0.18)	0.28 (0.25–0.31)	1.26 (1.16–1.37)	2.2 (1.93–2.52)	0.45 (0.42–0.49)	0.93 (0.83–1.04)	1.42 (1.36–1.49)	1.77 (1.62–1.92)	0.39 (0.33–0.45)	0.58 (0.46–0.72)
16.88	0.17 (0.16–0.19)	0.29 (0.26–0.32)	1.26 (1.16–1.36)	2.19 (1.92–2.5)	0.46 (0.43–0.49)	0.94 (0.84–1.05)	1.41 (1.35–1.48)	1.76 (1.61–1.91)	0.39 (0.34–0.46)	0.59 (0.47–0.73)
17.05	0.18 (0.17–0.19)	0.3 (0.27–0.33)	1.25 (1.15–1.35)	2.18 (1.91–2.49)	0.46 (0.43–0.5)	0.95 (0.85–1.07)	1.41 (1.35–1.47)	1.75 (1.61–1.9)	0.4 (0.34–0.46)	0.59 (0.48–0.74)
17.22	0.18 (0.17–0.2)	0.31 (0.28–0.34)	1.24 (1.15–1.34)	2.17 (1.9–2.47)	0.47 (0.44–0.51)	0.97 (0.86–1.08)	1.4 (1.34–1.46)	1.74 (1.6–1.89)	0.4 (0.35–0.47)	0.6 (0.48–0.75)
17.39	0.19 (0.18–0.2)	0.32 (0.28–0.35)	1.24 (1.14–1.34)	2.16 (1.89–2.46)	0.48 (0.44–0.51)	0.98 (0.88–1.09)	1.39 (1.33–1.45)	1.73 (1.59–1.88)	0.41 (0.36–0.47)	0.61 (0.49–0.76)
17.56	0.2 (0.18–0.21)	0.32 (0.29–0.36)	1.23 (1.14–1.33)	2.15 (1.89–2.44)	0.48 (0.45–0.52)	0.99 (0.89–1.1)	1.38 (1.33–1.44)	1.72 (1.58–1.86)	0.42 (0.36–0.48)	0.62 (0.5–0.77)
17.73	0.2 (0.19–0.21)	0.33 (0.3–0.37)	1.22 (1.14–1.32)	2.14 (1.88–2.43)	0.49 (0.46–0.52)	1 (0.9–1.12)	1.37 (1.32–1.43)	1.7 (1.57–1.85)	0.42 (0.37–0.48)	0.63 (0.51–0.78)
17.90	0.21 (0.19–0.22)	0.34 (0.31–0.38)	1.22 (1.13–1.31)	2.13 (1.87–2.42)	0.49 (0.46–0.53)	1.02 (0.91–1.13)	1.36 (1.31–1.42)	1.69 (1.56–1.84)	0.43 (0.37–0.49)	0.64 (0.52–0.8)
18.08	0.21 (0.2–0.23)	0.35 (0.32–0.39)	1.21 (1.13–1.3)	2.12 (1.86–2.4)	0.5 (0.47–0.53)	1.03 (0.92–1.14)	1.36 (1.3–1.41)	1.68 (1.55–1.83)	0.44 (0.38–0.5)	0.65 (0.53–0.81)
18.25	0.22 (0.2–0.23)	0.36 (0.33–0.4)	1.21 (1.12–1.29)	2.11 (1.86–2.39)	0.51 (0.48–0.54)	1.04 (0.94–1.16)	1.35 (1.3–1.4)	1.67 (1.54–1.82)	0.44 (0.39–0.5)	0.66 (0.53–0.82)
18.42	0.22 (0.21–0.24)	0.37 (0.34–0.41)	1.2 (1.12–1.29)	2.09 (1.85–2.37)	0.51 (0.48–0.55)	1.05 (0.95–1.17)	1.34 (1.29–1.39)	1.66 (1.53–1.81)	0.45 (0.39–0.51)	0.67 (0.54–0.83)

18.59	0.23 (0.22–0.25)	0.38 (0.35–0.42)	1.19 (1.12–1.28)	2.08 (1.84–2.36)	0.52 (0.49–0.55)	1.07 (0.96–1.18)	1.33 (1.28–1.38)	1.65 (1.53–1.79)	0.46 (0.4–0.52)	0.68 (0.55–0.84)
18.76	0.24 (0.22–0.25)	0.39 (0.36–0.43)	1.19 (1.11–1.27)	2.07 (1.83–2.35)	0.53 (0.5–0.56)	1.08 (0.98–1.2)	1.32 (1.28–1.37)	1.64 (1.52–1.78)	0.46 (0.41–0.52)	0.69 (0.56–0.85)
18.93	0.24 (0.23–0.26)	0.41 (0.37–0.45)	1.18 (1.11–1.26)	2.06 (1.82–2.33)	0.53 (0.5–0.57)	1.09 (0.99–1.21)	1.31 (1.27–1.36)	1.63 (1.51–1.77)	0.47 (0.42–0.53)	0.7 (0.57–0.86)
19.10	0.25 (0.24–0.27)	0.42 (0.38–0.46)	1.18 (1.1–1.25)	2.05 (1.82–2.32)	0.54 (0.51–0.57)	1.11 (1–1.23)	1.31 (1.26–1.35)	1.62 (1.5–1.76)	0.48 (0.42–0.54)	0.71 (0.58–0.87)
19.27	0.26 (0.24–0.27)	0.43 (0.39–0.47)	1.17 (1.1–1.25)	2.04 (1.81–2.31)	0.55 (0.52–0.58)	1.12 (1.02–1.24)	1.3 (1.26–1.34)	1.61 (1.49–1.75)	0.48 (0.43–0.54)	0.72 (0.59–0.89)
19.44	0.26 (0.25–0.28)	0.44 (0.4–0.48)	1.17 (1.1–1.24)	2.03 (1.8–2.29)	0.55 (0.52–0.59)	1.14 (1.03–1.26)	1.29 (1.25–1.33)	1.61 (1.48–1.74)	0.49 (0.44–0.55)	0.73 (0.6–0.9)
19.61	0.27 (0.26–0.29)	0.45 (0.41–0.5)	1.16 (1.09–1.23)	2.02 (1.79–2.28)	0.56 (0.53–0.59)	1.15 (1.04–1.27)	1.28 (1.24–1.32)	1.6 (1.47–1.73)	0.5 (0.45–0.56)	0.75 (0.61–0.91)
19.78	0.28 (0.26–0.3)	0.47 (0.43–0.51)	1.15 (1.09–1.22)	2.01 (1.79–2.27)	0.57 (0.54–0.6)	1.17 (1.06–1.29)	1.28 (1.24–1.32)	1.59 (1.47–1.72)	0.51 (0.45–0.56)	0.76 (0.62–0.92)
19.95	0.29 (0.27–0.3)	0.48 (0.44–0.52)	1.15 (1.08–1.22)	2 (1.78–2.26)	0.57 (0.55–0.61)	1.18 (1.07–1.3)	1.27 (1.23–1.31)	1.58 (1.46–1.7)	0.51 (0.46–0.57)	0.77 (0.63–0.93)
20.13	0.3 (0.28–0.31)	0.49 (0.45–0.54)	1.14 (1.08–1.21)	1.99 (1.77–2.24)	0.58 (0.55–0.61)	1.2 (1.08–1.32)	1.26 (1.22–1.3)	1.57 (1.45–1.69)	0.52 (0.47–0.58)	0.78 (0.64–0.95)
20.30	0.3 (0.29–0.32)	0.51 (0.46–0.55)	1.14 (1.08–1.2)	1.98 (1.76–2.23)	0.59 (0.56–0.62)	1.21 (1.1–1.33)	1.25 (1.22–1.29)	1.56 (1.44–1.68)	0.53 (0.48–0.59)	0.79 (0.65–0.96)
20.47	0.31 (0.3–0.33)	0.52 (0.48–0.57)	1.13 (1.07–1.19)	1.97 (1.76–2.22)	0.6 (0.57–0.63)	1.23 (1.11–1.35)	1.25 (1.21–1.28)	1.55 (1.43–1.67)	0.54 (0.49–0.59)	0.8 (0.66–0.97)
20.64	0.32 (0.31–0.34)	0.54 (0.49–0.58)	1.13 (1.07–1.18)	1.96 (1.75–2.2)	0.6 (0.58–0.63)	1.24 (1.13–1.37)	1.24 (1.2–1.27)	1.54 (1.42–1.66)	0.55 (0.5–0.6)	0.82 (0.67–0.99)
20.81	0.33 (0.32–0.35)	0.55 (0.5–0.6)	1.12 (1.07–1.18)	1.95 (1.74–2.19)	0.61 (0.58–0.64)	1.26 (1.14–1.38)	1.23 (1.2–1.26)	1.53 (1.42–1.65)	0.55 (0.51–0.61)	0.83 (0.69–1)
20.98	0.34 (0.32–0.36)	0.57 (0.52–0.62)	1.11 (1.06–1.17)	1.94 (1.73–2.18)	0.62 (0.59–0.65)	1.27 (1.16–1.4)	1.22 (1.19–1.25)	1.52 (1.41–1.64)	0.56 (0.51–0.62)	0.84 (0.7–1.02)
21.15	0.35 (0.33–0.37)	0.58 (0.53–0.63)	1.11 (1.06–1.16)	1.93 (1.73–2.17)	0.63 (0.6–0.66)	1.29 (1.17–1.42)	1.22 (1.19–1.25)	1.51 (1.4–1.63)	0.57 (0.52–0.62)	0.85 (0.71–1.03)
21.32	0.36 (0.34–0.37)	0.6 (0.55–0.65)	1.1 (1.05–1.16)	1.93 (1.72–2.16)	0.64 (0.61–0.66)	1.31 (1.19–1.43)	1.21 (1.18–1.24)	1.5 (1.39–1.62)	0.58 (0.53–0.63)	0.87 (0.72–1.04)
21.49	0.37 (0.35–0.38)	0.61 (0.57–0.67)	1.1 (1.05–1.15)	1.92 (1.71–2.14)	0.64 (0.62–0.67)	1.32 (1.21–1.45)	1.2 (1.17–1.23)	1.49 (1.38–1.61)	0.59 (0.54–0.64)	0.88 (0.73–1.06)
21.66	0.38 (0.36–0.4)	0.63 (0.58–0.69)	1.09 (1.05–1.14)	1.91 (1.7–2.13)	0.65 (0.63–0.68)	1.34 (1.22–1.47)	1.19 (1.17–1.22)	1.48 (1.38–1.6)	0.6 (0.55–0.65)	0.89 (0.74–1.07)
21.83	0.39 (0.38–0.41)	0.65 (0.6–0.71)	1.09 (1.04–1.13)	1.9 (1.7–2.12)	0.66 (0.64–0.69)	1.36 (1.24–1.48)	1.19 (1.16–1.21)	1.48 (1.37–1.59)	0.61 (0.56–0.66)	0.91 (0.76–1.09)
22.01	0.4 (0.39–0.42)	0.67 (0.62–0.73)	1.08 (1.04–1.13)	1.89 (1.69–2.11)	0.67 (0.64–0.69)	1.37 (1.25–1.5)	1.18 (1.15–1.21)	1.47 (1.36–1.58)	0.62 (0.57–0.66)	0.92 (0.77–1.1)
22.18	0.41 (0.4–0.43)	0.69 (0.63–0.75)	1.08 (1.04–1.12)	1.88 (1.68–2.1)	0.68 (0.65–0.7)	1.39 (1.27–1.52)	1.17 (1.15–1.2)	1.46 (1.35–1.57)	0.62 (0.58–0.67)	0.93 (0.78–1.12)
22.35	0.42 (0.41–0.44)	0.71 (0.65–0.77)	1.07 (1.03–1.11)	1.87 (1.67–2.09)	0.69 (0.66–0.71)	1.41 (1.29–1.54)	1.17 (1.14–1.19)	1.45 (1.34–1.56)	0.63 (0.59–0.68)	0.95 (0.79–1.13)
22.52	0.44 (0.42–0.45)	0.73 (0.67–0.79)	1.07 (1.03–1.11)	1.86 (1.67–2.07)	0.69 (0.67–0.72)	1.43 (1.31–1.56)	1.16 (1.14–1.18)	1.44 (1.34–1.55)	0.64 (0.6–0.69)	0.96 (0.81–1.15)
22.69	0.45 (0.43–0.46)	0.75 (0.69–0.81)	1.06 (1.02–1.1)	1.85 (1.66–2.06)	0.7 (0.68–0.73)	1.44 (1.32–1.58)	1.15 (1.13–1.17)	1.43 (1.33–1.54)	0.65 (0.61–0.7)	0.98 (0.82–1.17)
22.86	0.46 (0.45–0.48)	0.77 (0.71–0.83)	1.06 (1.02–1.09)	1.84 (1.65–2.05)	0.71 (0.69–0.73)	1.46 (1.34–1.6)	1.14 (1.12–1.17)	1.42 (1.32–1.53)	0.66 (0.62–0.71)	0.99 (0.83–1.18)
23.03	0.47 (0.46–0.49)	0.79 (0.73–0.85)	1.05 (1.02–1.08)	1.83 (1.65–2.04)	0.72 (0.7–0.74)	1.48 (1.36–1.62)	1.14 (1.12–1.16)	1.41 (1.31–1.52)	0.67 (0.63–0.72)	1.01 (0.85–1.2)

23.20	0.49 (0.47–0.5)	0.81 (0.75–0.88)	1.04 (1.01–1.08)	1.82 (1.64–2.03)	0.73 (0.71–0.75)	1.5 (1.38–1.63)	1.13 (1.11–1.15)	1.41 (1.31–1.51)	0.68 (0.65–0.72)	1.02 (0.86–1.22)
23.37	0.5 (0.49–0.52)	0.83 (0.77–0.9)	1.04 (1.01–1.07)	1.81 (1.63–2.02)	0.74 (0.72–0.76)	1.52 (1.39–1.65)	1.12 (1.11–1.14)	1.4 (1.3–1.5)	0.69 (0.66–0.73)	1.04 (0.87–1.23)
23.54	0.52 (0.5–0.53)	0.86 (0.79–0.93)	1.03 (1.01–1.06)	1.81 (1.62–2.01)	0.75 (0.73–0.77)	1.54 (1.41–1.67)	1.12 (1.1–1.13)	1.39 (1.29–1.49)	0.71 (0.67–0.74)	1.05 (0.89–1.25)
23.71	0.53 (0.52–0.54)	0.88 (0.82–0.95)	1.03 (1–1.06)	1.8 (1.62–2)	0.76 (0.74–0.78)	1.56 (1.43–1.7)	1.11 (1.1–1.13)	1.38 (1.28–1.49)	0.72 (0.68–0.75)	1.07 (0.9–1.27)
23.88	0.54 (0.53–0.56)	0.91 (0.84–0.98)	1.02 (1–1.05)	1.79 (1.61–1.99)	0.77 (0.75–0.79)	1.58 (1.45–1.72)	1.1 (1.09–1.12)	1.37 (1.28–1.48)	0.73 (0.69–0.76)	1.09 (0.92–1.29)
24.06	0.56 (0.55–0.57)	0.93 (0.86–1)	1.02 (1–1.04)	1.78 (1.6–1.98)	0.78 (0.76–0.79)	1.6 (1.47–1.74)	1.1 (1.08–1.11)	1.36 (1.27–1.47)	0.74 (0.71–0.77)	1.1 (0.93–1.31)
24.23	0.58 (0.56–0.59)	0.96 (0.89–1.03)	1.02 (0.99–1.04)	1.77 (1.6–1.97)	0.79 (0.77–0.8)	1.62 (1.49–1.76)	1.09 (1.08–1.1)	1.36 (1.26–1.46)	0.75 (0.72–0.78)	1.12 (0.95–1.32)
24.40	0.59 (0.58–0.6)	0.98 (0.91–1.06)	1.01 (0.99–1.03)	1.76 (1.59–1.96)	0.8 (0.78–0.81)	1.64 (1.5–1.78)	1.09 (1.07–1.1)	1.35 (1.25–1.45)	0.76 (0.73–0.79)	1.14 (0.96–1.34)
24.57	0.61 (0.6–0.62)	1.01 (0.94–1.09)	1.01 (0.99–1.03)	1.76 (1.58–1.95)	0.81 (0.79–0.82)	1.66 (1.52–1.8)	1.08 (1.07–1.09)	1.34 (1.25–1.44)	0.77 (0.74–0.8)	1.15 (0.97–1.36)
24.74	0.62 (0.61–0.64)	1.04 (0.96–1.12)	1 (0.98–1.02)	1.75 (1.58–1.94)	0.82 (0.8–0.83)	1.68 (1.54–1.82)	1.07 (1.06–1.08)	1.33 (1.24–1.43)	0.78 (0.76–0.81)	1.17 (0.99–1.38)
24.91	0.64 (0.63–0.65)	1.07 (0.99–1.15)	1 (0.98–1.02)	1.74 (1.57–1.93)	0.83 (0.81–0.84)	1.7 (1.56–1.84)	1.07 (1.06–1.08)	1.33 (1.23–1.43)	0.79 (0.77–0.82)	1.19 (1–1.4)
25.08	0.66 (0.65–0.67)	1.1 (1.02–1.18)	1 (0.98–1.01)	1.74 (1.57–1.93)	0.84 (0.82–0.85)	1.72 (1.58–1.86)	1.06 (1.05–1.07)	1.32 (1.23–1.42)	0.81 (0.78–0.83)	1.2 (1.02–1.42)
25.25	0.68 (0.67–0.69)	1.13 (1.05–1.21)	0.99 (0.98–1.01)	1.73 (1.56–1.92)	0.85 (0.83–0.86)	1.74 (1.6–1.89)	1.06 (1.05–1.06)	1.31 (1.22–1.41)	0.82 (0.79–0.84)	1.22 (1.03–1.44)
25.42	0.69 (0.69–0.7)	1.16 (1.07–1.24)	0.99 (0.98–1)	1.73 (1.56–1.91)	0.86 (0.84–0.87)	1.76 (1.62–1.91)	1.05 (1.04–1.06)	1.31 (1.22–1.4)	0.83 (0.81–0.85)	1.24 (1.05–1.46)
25.59	0.71 (0.7–0.72)	1.19 (1.1–1.28)	0.99 (0.98–1)	1.72 (1.56–1.91)	0.87 (0.86–0.88)	1.78 (1.64–1.93)	1.05 (1.04–1.05)	1.3 (1.21–1.4)	0.84 (0.82–0.86)	1.26 (1.07–1.48)
25.76	0.73 (0.72–0.74)	1.22 (1.13–1.31)	0.99 (0.97–1)	1.72 (1.55–1.9)	0.88 (0.87–0.89)	1.8 (1.66–1.95)	1.04 (1.03–1.05)	1.29 (1.2–1.39)	0.85 (0.83–0.87)	1.27 (1.08–1.5)
25.93	0.75 (0.74–0.76)	1.25 (1.16–1.34)	0.98 (0.97–0.99)	1.72 (1.55–1.9)	0.89 (0.88–0.89)	1.82 (1.68–1.97)	1.04 (1.03–1.04)	1.29 (1.2–1.38)	0.86 (0.85–0.88)	1.29 (1.1–1.52)
26.11	0.77 (0.76–0.78)	1.28 (1.19–1.38)	0.98 (0.97–0.99)	1.71 (1.55–1.9)	0.9 (0.89–0.9)	1.84 (1.7–2)	1.03 (1.03–1.04)	1.28 (1.19–1.38)	0.87 (0.86–0.89)	1.31 (1.11–1.54)
26.28	0.79 (0.78–0.8)	1.31 (1.22–1.41)	0.98 (0.97–0.99)	1.71 (1.55–1.9)	0.91 (0.9–0.91)	1.86 (1.72–2.02)	1.03 (1.02–1.03)	1.28 (1.19–1.37)	0.89 (0.87–0.9)	1.33 (1.13–1.56)
26.45	0.81 (0.8–0.82)	1.35 (1.25–1.45)	0.98 (0.97–0.99)	1.71 (1.55–1.89)	0.92 (0.91–0.92)	1.88 (1.73–2.04)	1.02 (1.02–1.03)	1.27 (1.19–1.37)	0.9 (0.89–0.91)	1.34 (1.14–1.58)
26.62	0.83 (0.82–0.83)	1.38 (1.29–1.48)	0.98 (0.97–0.99)	1.71 (1.55–1.89)	0.93 (0.92–0.93)	1.9 (1.75–2.06)	1.02 (1.02–1.02)	1.27 (1.18–1.36)	0.91 (0.9–0.92)	1.36 (1.16–1.6)
26.79	0.85 (0.85–0.85)	1.42 (1.32–1.52)	0.98 (0.98–0.99)	1.71 (1.55–1.89)	0.93 (0.93–0.94)	1.92 (1.77–2.08)	1.02 (1.01–1.02)	1.26 (1.18–1.36)	0.92 (0.91–0.93)	1.38 (1.17–1.62)
26.96	0.87 (0.87–0.87)	1.45 (1.35–1.56)	0.98 (0.98–0.99)	1.71 (1.55–1.9)	0.94 (0.94–0.95)	1.94 (1.79–2.1)	1.01 (1.01–1.02)	1.26 (1.17–1.35)	0.93 (0.92–0.94)	1.39 (1.19–1.64)
27.13	0.89 (0.89–0.89)	1.48 (1.38–1.59)	0.98 (0.98–0.99)	1.72 (1.55–1.9)	0.95 (0.95–0.96)	1.96 (1.81–2.12)	1.01 (1.01–1.01)	1.26 (1.17–1.35)	0.94 (0.94–0.95)	1.41 (1.2–1.66)
27.30	0.91 (0.91–0.92)	1.52 (1.42–1.63)	0.99 (0.98–0.99)	1.72 (1.55–1.9)	0.96 (0.96–0.97)	1.98 (1.83–2.14)	1.01 (1.01–1.01)	1.25 (1.17–1.35)	0.96 (0.95–0.96)	1.43 (1.22–1.68)
27.47	0.93 (0.93–0.94)	1.55 (1.45–1.67)	0.99 (0.99–0.99)	1.72 (1.56–1.91)	0.97 (0.97–0.97)	2 (1.84–2.16)	1.01 (1–1.01)	1.25 (1.16–1.34)	0.97 (0.96–0.97)	1.44 (1.23–1.7)
27.64	0.96 (0.95–0.96)	1.59 (1.48–1.71)	0.99 (0.99–0.99)	1.73 (1.56–1.91)	0.98 (0.98–0.98)	2.02 (1.86–2.18)	1 (1–1)	1.25 (1.16–1.34)	0.98 (0.97–0.98)	1.46 (1.24–1.72)

27.81	0.98 (0.98–0.98)	1.63 (1.51–1.75)	1 (0.99–1)	1.74 (1.57–1.92)	0.99 (0.99–0.99)	2.03 (1.88–2.2)	1 (1–1)	1.25 (1.16–1.34)	0.99 (0.99–0.99)	1.48 (1.26–1.74)
27.98	1 (1–1)	1.66 (1.55–1.78)	1 (1–1)	1.74 (1.58–1.93)	1 (1–1)	2.05 (1.9–2.22)	1 (1–1)	1.24 (1.16–1.33)	1 (1–1)	1.49 (1.27–1.75)
**28.00**	**Reference**	**Reference**	**Reference**	**Reference**	**Reference**	**Reference**	**Reference**	**Reference**	**Reference**	**Reference**
28.16	1.02 (1.02–1.02)	1.7 (1.58–1.82)	1 (1–1.01)	1.75 (1.59–1.94)	1.01 (1.01–1.01)	2.07 (1.91–2.24)	1 (1–1)	1.24 (1.16–1.33)	1.01 (1.01–1.01)	1.51 (1.29–1.77)
28.33	1.04 (1.04–1.04)	1.73 (1.61–1.86)	1.01 (1.01–1.01)	1.76 (1.59–1.95)	1.02 (1.02–1.02)	2.09 (1.93–2.26)	1 (1–1)	1.24 (1.16–1.33)	1.02 (1.02–1.02)	1.52 (1.3–1.79)
28.50	1.06 (1.06–1.06)	1.77 (1.65–1.9)	1.02 (1.02–1.02)	1.77 (1.61–1.96)	1.02 (1.02–1.03)	2.1 (1.94–2.28)	1 (1–1)	1.24 (1.16–1.33)	1.03 (1.03–1.03)	1.54 (1.31–1.81)
28.67	1.08 (1.08–1.09)	1.81 (1.68–1.94)	1.02 (1.02–1.03)	1.79 (1.62–1.98)	1.03 (1.03–1.03)	2.12 (1.96–2.3)	1 (1–1)	1.24 (1.16–1.33)	1.04 (1.04–1.04)	1.55 (1.32–1.83)
28.84	1.11 (1.1–1.11)	1.84 (1.72–1.98)	1.03 (1.03–1.03)	1.8 (1.63–1.99)	1.04 (1.04–1.04)	2.14 (1.97–2.31)	1 (1–1)	1.24 (1.16–1.33)	1.05 (1.04–1.06)	1.57 (1.34–1.84)
29.01	1.13 (1.12–1.13)	1.88 (1.75–2.01)	1.04 (1.04–1.04)	1.82 (1.64–2.01)	1.05 (1.04–1.05)	2.15 (1.99–2.33)	1 (1–1)	1.24 (1.16–1.33)	1.06 (1.05–1.07)	1.58 (1.35–1.86)
29.18	1.15 (1.15–1.15)	1.91 (1.78–2.05)	1.05 (1.05–1.05)	1.83 (1.66–2.02)	1.06 (1.05–1.06)	2.17 (2–2.35)	1 (1–1)	1.24 (1.16–1.33)	1.07 (1.06–1.08)	1.6 (1.36–1.87)
29.35	1.17 (1.17–1.18)	1.95 (1.82–2.09)	1.06 (1.05–1.06)	1.85 (1.67–2.04)	1.06 (1.06–1.07)	2.18 (2.02–2.36)	1 (1–1)	1.24 (1.16–1.33)	1.08 (1.07–1.09)	1.61 (1.37–1.89)
29.52	1.19 (1.19–1.2)	1.98 (1.85–2.13)	1.07 (1.06–1.08)	1.87 (1.69–2.06)	1.07 (1.06–1.07)	2.2 (2.03–2.38)	1 (1–1)	1.24 (1.16–1.33)	1.09 (1.08–1.1)	1.62 (1.38–1.91)
29.69	1.21 (1.21–1.22)	2.02 (1.88–2.17)	1.08 (1.08–1.09)	1.89 (1.71–2.09)	1.08 (1.07–1.08)	2.21 (2.04–2.39)	1 (1–1.01)	1.25 (1.16–1.34)	1.1 (1.08–1.11)	1.64 (1.4–1.92)
29.86	1.24 (1.23–1.24)	2.06 (1.92–2.21)	1.09 (1.09–1.1)	1.91 (1.73–2.11)	1.08 (1.08–1.09)	2.23 (2.05–2.41)	1 (1–1.01)	1.25 (1.16–1.34)	1.1 (1.09–1.12)	1.65 (1.41–1.94)
30.04	1.26 (1.25–1.26)	2.09 (1.95–2.25)	1.11 (1.1–1.11)	1.93 (1.75–2.13)	1.09 (1.08–1.1)	2.24 (2.07–2.42)	1.01 (1–1.01)	1.25 (1.16–1.34)	1.11 (1.1–1.13)	1.66 (1.42–1.95)
30.21	1.28 (1.27–1.29)	2.13 (1.98–2.28)	1.12 (1.11–1.13)	1.95 (1.77–2.16)	1.1 (1.09–1.1)	2.25 (2.08–2.44)	1.01 (1–1.01)	1.25 (1.17–1.34)	1.12 (1.11–1.13)	1.67 (1.43–1.97)
30.38	1.3 (1.29–1.31)	2.16 (2.02–2.32)	1.13 (1.12–1.14)	1.98 (1.79–2.18)	1.1 (1.09–1.11)	2.26 (2.09–2.45)	1.01 (1.01–1.01)	1.26 (1.17–1.35)	1.13 (1.11–1.14)	1.69 (1.44–1.98)
30.55	1.32 (1.31–1.33)	2.2 (2.05–2.36)	1.15 (1.14–1.16)	2 (1.81–2.21)	1.11 (1.1–1.12)	2.28 (2.1–2.46)	1.01 (1.01–1.02)	1.26 (1.17–1.35)	1.14 (1.12–1.15)	1.7 (1.45–1.99)
30.72	1.34 (1.33–1.35)	2.24 (2.08–2.4)	1.16 (1.15–1.17)	2.03 (1.84–2.24)	1.11 (1.11–1.12)	2.29 (2.11–2.48)	1.02 (1.01–1.02)	1.26 (1.18–1.35)	1.14 (1.13–1.16)	1.71 (1.46–2.01)
30.89	1.36 (1.35–1.37)	2.27 (2.12–2.44)	1.18 (1.17–1.19)	2.06 (1.86–2.27)	1.12 (1.11–1.13)	2.3 (2.12–2.49)	1.02 (1.01–1.02)	1.27 (1.18–1.36)	1.15 (1.13–1.17)	1.72 (1.47–2.02)
31.06	1.39 (1.38–1.4)	2.31 (2.15–2.48)	1.2 (1.18–1.21)	2.09 (1.89–2.3)	1.13 (1.12–1.14)	2.31 (2.14–2.5)	1.02 (1.02–1.03)	1.27 (1.18–1.36)	1.16 (1.14–1.18)	1.73 (1.48–2.03)
31.23	1.41 (1.4–1.42)	2.34 (2.18–2.51)	1.21 (1.2–1.22)	2.12 (1.91–2.34)	1.13 (1.12–1.14)	2.32 (2.15–2.51)	1.03 (1.02–1.03)	1.27 (1.19–1.37)	1.17 (1.15–1.19)	1.74 (1.49–2.05)
31.40	1.43 (1.42–1.44)	2.38 (2.22–2.55)	1.23 (1.22–1.24)	2.15 (1.94–2.37)	1.14 (1.12–1.15)	2.33 (2.16–2.53)	1.03 (1.02–1.04)	1.28 (1.19–1.37)	1.17 (1.15–1.2)	1.75 (1.49–2.06)
31.57	1.45 (1.44–1.46)	2.41 (2.25–2.59)	1.25 (1.24–1.26)	2.18 (1.97–2.41)	1.14 (1.13–1.15)	2.34 (2.17–2.54)	1.03 (1.02–1.04)	1.28 (1.2–1.38)	1.18 (1.16–1.2)	1.76 (1.5–2.07)
31.74	1.47 (1.46–1.49)	2.45 (2.28–2.63)	1.27 (1.25–1.28)	2.21 (2–2.45)	1.15 (1.13–1.16)	2.35 (2.17–2.55)	1.04 (1.03–1.04)	1.29 (1.2–1.38)	1.19 (1.16–1.21)	1.77 (1.51–2.08)
31.91	1.49 (1.48–1.51)	2.49 (2.32–2.67)	1.29 (1.27–1.31)	2.25 (2.03–2.49)	1.15 (1.14–1.16)	2.36 (2.18–2.56)	1.04 (1.03–1.05)	1.29 (1.21–1.39)	1.19 (1.17–1.22)	1.78 (1.52–2.09)
32.09	1.52 (1.5–1.53)	2.52 (2.35–2.71)	1.31 (1.29–1.33)	2.29 (2.07–2.53)	1.16 (1.14–1.17)	2.37 (2.19–2.57)	1.05 (1.04–1.05)	1.3 (1.21–1.39)	1.2 (1.17–1.23)	1.79 (1.53–2.1)

32.26	1.54 (1.52–1.55)	2.56 (2.38–2.75)	1.33 (1.31–1.35)	2.32 (2.1–2.57)	1.16 (1.15–1.18)	2.38 (2.2–2.58)	1.05 (1.04–1.06)	1.3 (1.22–1.4)	1.21 (1.18–1.24)	1.8 (1.54–2.12)
32.43	1.56 (1.54–1.58)	2.59 (2.42–2.78)	1.35 (1.33–1.37)	2.36 (2.14–2.61)	1.17 (1.15–1.18)	2.39 (2.21–2.59)	1.05 (1.04–1.06)	1.31 (1.22–1.41)	1.21 (1.18–1.24)	1.81 (1.54–2.13)
32.60	1.58 (1.56–1.6)	2.63 (2.45–2.82)	1.38 (1.36–1.4)	2.4 (2.17–2.65)	1.17 (1.15–1.19)	2.4 (2.22–2.6)	1.06 (1.05–1.07)	1.32 (1.23–1.41)	1.22 (1.19–1.25)	1.82 (1.55–2.14)
32.77	1.6 (1.58–1.62)	2.67 (2.48–2.86)	1.4 (1.38–1.42)	2.44 (2.21–2.7)	1.17 (1.16–1.19)	2.41 (2.23–2.61)	1.06 (1.05–1.08)	1.32 (1.23–1.42)	1.22 (1.19–1.26)	1.83 (1.56–2.15)
32.94	1.62 (1.6–1.64)	2.7 (2.52–2.9)	1.42 (1.4–1.45)	2.49 (2.25–2.75)	1.18 (1.16–1.2)	2.42 (2.24–2.62)	1.07 (1.06–1.08)	1.33 (1.24–1.43)	1.23 (1.19–1.27)	1.84 (1.57–2.16)
33.11	1.65 (1.62–1.67)	2.74 (2.55–2.94)	1.45 (1.42–1.48)	2.53 (2.29–2.8)	1.18 (1.16–1.2)	2.43 (2.24–2.63)	1.07 (1.06–1.09)	1.34 (1.24–1.43)	1.24 (1.2–1.28)	1.85 (1.57–2.17)
33.28	1.67 (1.65–1.69)	2.78 (2.59–2.98)	1.48 (1.45–1.5)	2.57 (2.33–2.85)	1.19 (1.17–1.21)	2.44 (2.25–2.64)	1.08 (1.07–1.09)	1.34 (1.25–1.44)	1.24 (1.2–1.28)	1.86 (1.58–2.18)
33.45	1.69 (1.67–1.71)	2.81 (2.62–3.02)	1.5 (1.47–1.53)	2.62 (2.37–2.9)	1.19 (1.17–1.21)	2.45 (2.26–2.65)	1.08 (1.07–1.1)	1.35 (1.26–1.45)	1.25 (1.21–1.29)	1.87 (1.59–2.19)
33.62	1.71 (1.69–1.74)	2.85 (2.65–3.06)	1.53 (1.5–1.56)	2.67 (2.41–2.95)	1.19 (1.17–1.22)	2.45 (2.27–2.66)	1.09 (1.08–1.11)	1.36 (1.26–1.46)	1.25 (1.21–1.3)	1.87 (1.6–2.2)
33.79	1.74 (1.71–1.76)	2.89 (2.69–3.1)	1.56 (1.52–1.59)	2.72 (2.46–3)	1.2 (1.18–1.22)	2.46 (2.27–2.67)	1.1 (1.08–1.11)	1.36 (1.27–1.46)	1.26 (1.21–1.31)	1.88 (1.6–2.21)
33.96	1.76 (1.73–1.79)	2.93 (2.72–3.14)	1.59 (1.55–1.62)	2.77 (2.5–3.06)	1.2 (1.18–1.23)	2.47 (2.28–2.68)	1.1 (1.09–1.12)	1.37 (1.28–1.47)	1.27 (1.22–1.31)	1.89 (1.61–2.22)
34.14	1.78 (1.75–1.81)	2.96 (2.76–3.18)	1.61 (1.58–1.65)	2.82 (2.55–3.12)	1.21 (1.18–1.23)	2.48 (2.29–2.69)	1.11 (1.09–1.12)	1.38 (1.28–1.48)	1.27 (1.22–1.32)	1.9 (1.62–2.23)
34.31	1.8 (1.77–1.83)	3 (2.79–3.23)	1.64 (1.61–1.68)	2.87 (2.59–3.18)	1.21 (1.18–1.24)	2.49 (2.29–2.7)	1.11 (1.1–1.13)	1.38 (1.29–1.49)	1.28 (1.22–1.33)	1.91 (1.62–2.24)
34.48	1.83 (1.79–1.86)	3.04 (2.83–3.27)	1.68 (1.63–1.72)	2.92 (2.64–3.23)	1.21 (1.19–1.24)	2.5 (2.3–2.71)	1.12 (1.1–1.14)	1.39 (1.3–1.49)	1.28 (1.23–1.34)	1.92 (1.63–2.25)
34.65	1.85 (1.82–1.88)	3.08 (2.87–3.31)	1.71 (1.66–1.75)	2.98 (2.69–3.3)	1.22 (1.19–1.25)	2.5 (2.31–2.71)	1.13 (1.11–1.15)	1.4 (1.3–1.5)	1.29 (1.23–1.35)	1.92 (1.64–2.27)
34.82	1.87 (1.84–1.91)	3.12 (2.9–3.35)	1.74 (1.69–1.79)	3.03 (2.74–3.36)	1.22 (1.19–1.25)	2.51 (2.31–2.72)	1.13 (1.11–1.15)	1.41 (1.31–1.51)	1.29 (1.23–1.35)	1.93 (1.64–2.28)
34.99	1.9 (1.86–1.94)	3.16 (2.94–3.4)	1.77 (1.72–1.82)	3.09 (2.79–3.42)	1.23 (1.19–1.26)	2.52 (2.32–2.73)	1.14 (1.12–1.16)	1.41 (1.32–1.52)	1.3 (1.24–1.36)	1.94 (1.65–2.29)
35.16	1.92 (1.88–1.96)	3.2 (2.98–3.44)	1.8 (1.75–1.86)	3.15 (2.84–3.49)	1.23 (1.2–1.26)	2.53 (2.33–2.74)	1.14 (1.12–1.17)	1.42 (1.32–1.53)	1.3 (1.24–1.37)	1.95 (1.65–2.3)
35.33	1.95 (1.91–1.99)	3.24 (3.01–3.48)	1.84 (1.78–1.89)	3.21 (2.9–3.55)	1.23 (1.2–1.27)	2.53 (2.33–2.75)	1.15 (1.13–1.17)	1.43 (1.33–1.54)	1.31 (1.24–1.38)	1.96 (1.66–2.31)
35.50	1.97 (1.93–2.02)	3.28 (3.05–3.53)	1.87 (1.82–1.93)	3.27 (2.95–3.62)	1.24 (1.2–1.27)	2.54 (2.34–2.76)	1.16 (1.13–1.18)	1.44 (1.34–1.54)	1.32 (1.25–1.39)	1.97 (1.67–2.32)
35.67	2 (1.95–2.04)	3.32 (3.09–3.58)	1.91 (1.85–1.97)	3.33 (3–3.69)	1.24 (1.21–1.28)	2.55 (2.35–2.77)	1.16 (1.14–1.19)	1.44 (1.34–1.55)	1.32 (1.25–1.39)	1.98 (1.67–2.33)
35.84	2.02 (1.98–2.07)	3.37 (3.13–3.62)	1.94 (1.88–2.01)	3.39 (3.06–3.76)	1.25 (1.21–1.28)	2.56 (2.36–2.78)	1.17 (1.14–1.19)	1.45 (1.35–1.56)	1.33 (1.25–1.4)	1.98 (1.68–2.34)
36.02	2.05 (2–2.1)	3.41 (3.17–3.67)	1.98 (1.91–2.05)	3.45 (3.12–3.83)	1.25 (1.21–1.29)	2.57 (2.36–2.79)	1.17 (1.15–1.2)	1.46 (1.36–1.57)	1.33 (1.26–1.41)	1.99 (1.69–2.35)
36.19	2.08 (2.03–2.13)	3.45 (3.21–3.72)	2.02 (1.95–2.09)	3.52 (3.17–3.9)	1.25 (1.21–1.29)	2.57 (2.37–2.8)	1.18 (1.15–1.21)	1.47 (1.36–1.58)	1.34 (1.26–1.42)	2 (1.69–2.36)
36.36	2.1 (2.05–2.15)	3.5 (3.25–3.77)	2.05 (1.98–2.13)	3.58 (3.23–3.98)	1.26 (1.22–1.3)	2.58 (2.38–2.81)	1.19 (1.16–1.22)	1.48 (1.37–1.59)	1.34 (1.26–1.43)	2.01 (1.7–2.37)
36.53	2.13 (2.07–2.18)	3.54 (3.29–3.81)	2.09 (2.02–2.17)	3.65 (3.29–4.05)	1.26 (1.22–1.3)	2.59 (2.38–2.82)	1.19 (1.16–1.22)	1.48 (1.38–1.6)	1.35 (1.27–1.44)	2.02 (1.71–2.39)
36.70	2.16 (2.1–2.21)	3.59 (3.33–3.86)	2.13 (2.05–2.21)	3.72 (3.35–4.13)	1.27 (1.22–1.31)	2.6 (2.39–2.83)	1.2 (1.17–1.23)	1.49 (1.39–1.61)	1.36 (1.27–1.45)	2.03 (1.71–2.4)

36.87	2.18 (2.13–2.24)	3.64 (3.37–3.92)	2.17 (2.09–2.26)	3.79 (3.41–4.21)	1.27 (1.22–1.32)	2.61 (2.4–2.84)	1.21 (1.18–1.24)	1.5 (1.39–1.61)	1.36 (1.27–1.45)	2.04 (1.72–2.41)
37.04	2.21 (2.15–2.27)	3.68 (3.42–3.97)	2.21 (2.13–2.3)	3.86 (3.48–4.29)	1.27 (1.23–1.32)	2.62 (2.4–2.85)	1.21 (1.18–1.25)	1.51 (1.4–1.62)	1.37 (1.28–1.46)	2.04 (1.73–2.42)
37.21	2.24 (2.18–2.3)	3.73 (3.46–4.02)	2.25 (2.17–2.35)	3.93 (3.54–4.37)	1.28 (1.23–1.33)	2.62 (2.41–2.86)	1.22 (1.19–1.25)	1.52 (1.41–1.63)	1.37 (1.28–1.47)	2.05 (1.73–2.43)
37.38	2.27 (2.2–2.34)	3.78 (3.5–4.07)	2.3 (2.2–2.39)	4.01 (3.61–4.45)	1.28 (1.23–1.33)	2.63 (2.42–2.87)	1.23 (1.19–1.26)	1.52 (1.42–1.64)	1.38 (1.28–1.48)	2.06 (1.74–2.44)
37.55	2.3 (2.23–2.37)	3.83 (3.55–4.12)	2.34 (2.24–2.44)	4.08 (3.67–4.54)	1.29 (1.24–1.34)	2.64 (2.42–2.88)	1.23 (1.2–1.27)	1.53 (1.42–1.65)	1.39 (1.29–1.49)	2.07 (1.75–2.46)
37.72	2.33 (2.26–2.4)	3.87 (3.59–4.18)	2.38 (2.28–2.49)	4.16 (3.74–4.62)	1.29 (1.24–1.34)	2.65 (2.43–2.89)	1.24 (1.2–1.28)	1.54 (1.43–1.66)	1.39 (1.29–1.5)	2.08 (1.75–2.47)
37.89	2.36 (2.28–2.43)	3.92 (3.64–4.23)	2.43 (2.32–2.54)	4.24 (3.81–4.71)	1.29 (1.24–1.35)	2.66 (2.44–2.9)	1.25 (1.21–1.28)	1.55 (1.44–1.67)	1.4 (1.3–1.51)	2.09 (1.76–2.48)
38.07	2.39 (2.31–2.46)	3.97 (3.68–4.29)	2.47 (2.37–2.59)	4.31 (3.88–4.8)	1.3 (1.24–1.35)	2.67 (2.44–2.91)	1.25 (1.22–1.29)	1.56 (1.44–1.68)	1.4 (1.3–1.52)	2.1 (1.76–2.49)
38.24	2.42 (2.34–2.5)	4.03 (3.73–4.34)	2.52 (2.41–2.64)	4.4 (3.95–4.89)	1.3 (1.25–1.36)	2.67 (2.45–2.92)	1.26 (1.22–1.3)	1.57 (1.45–1.69)	1.41 (1.3–1.52)	2.11 (1.77–2.51)
38.41	2.45 (2.37–2.53)	4.08 (3.78–4.4)	2.57 (2.45–2.69)	4.48 (4.02–4.99)	1.31 (1.25–1.36)	2.68 (2.46–2.93)	1.27 (1.23–1.31)	1.57 (1.46–1.7)	1.42 (1.31–1.53)	2.12 (1.78–2.52)
38.58	2.48 (2.4–2.57)	4.13 (3.82–4.46)	2.61 (2.49–2.74)	4.56 (4.1–5.08)	1.31 (1.25–1.37)	2.69 (2.46–2.94)	1.27 (1.23–1.31)	1.58 (1.47–1.71)	1.42 (1.31–1.54)	2.12 (1.78–2.53)
38.75	2.51 (2.43–2.6)	4.18 (3.87–4.52)	2.66 (2.54–2.8)	4.65 (4.17–5.18)	1.31 (1.26–1.37)	2.7 (2.47–2.95)	1.28 (1.24–1.32)	1.59 (1.47–1.72)	1.43 (1.31–1.55)	2.13 (1.79–2.54)
38.92	2.54 (2.46–2.64)	4.24 (3.92–4.58)	2.71 (2.58–2.85)	4.73 (4.25–5.28)	1.32 (1.26–1.38)	2.71 (2.48–2.96)	1.29 (1.24–1.33)	1.6 (1.48–1.73)	1.43 (1.32–1.56)	2.14 (1.8–2.56)
39.09	2.58 (2.49–2.67)	4.29 (3.97–4.64)	2.76 (2.63–2.91)	4.82 (4.32–5.38)	1.32 (1.26–1.39)	2.72 (2.48–2.97)	1.29 (1.25–1.34)	1.61 (1.49–1.74)	1.44 (1.32–1.57)	2.15 (1.8–2.57)
39.26	2.61 (2.52–2.71)	4.34 (4.02–4.7)	2.82 (2.67–2.96)	4.91 (4.4–5.48)	1.33 (1.26–1.39)	2.72 (2.49–2.98)	1.3 (1.26–1.35)	1.62 (1.5–1.75)	1.45 (1.32–1.58)	2.16 (1.81–2.58)
39.43	2.64 (2.55–2.74)	4.4 (4.07–4.76)	2.87 (2.72–3.02)	5 (4.48–5.59)	1.33 (1.27–1.4)	2.73 (2.5–2.99)	1.31 (1.26–1.35)	1.63 (1.51–1.76)	1.45 (1.33–1.59)	2.17 (1.82–2.59)
39.60	2.68 (2.58–2.78)	4.46 (4.12–4.82)	2.92 (2.77–3.08)	5.1 (4.57–5.69)	1.33 (1.27–1.4)	2.74 (2.5–3)	1.31 (1.27–1.36)	1.63 (1.51–1.77)	1.46 (1.33–1.6)	2.18 (1.82–2.61)
39.77	2.71 (2.61–2.82)	4.51 (4.17–4.88)	2.98 (2.82–3.14)	5.19 (4.65–5.8)	1.34 (1.27–1.41)	2.75 (2.51–3.01)	1.32 (1.27–1.37)	1.64 (1.52–1.78)	1.46 (1.33–1.61)	2.19 (1.83–2.62)
39.94	2.75 (2.64–2.86)	4.57 (4.22–4.95)	3.03 (2.87–3.2)	5.29 (4.74–5.91)	1.34 (1.28–1.41)	2.76 (2.52–3.02)	1.33 (1.28–1.38)	1.65 (1.53–1.79)	1.47 (1.34–1.62)	2.2 (1.83–2.63)
40.12	2.78 (2.67–2.9)	4.63 (4.28–5.01)	3.09 (2.92–3.27)	5.39 (4.82–6.03)	1.35 (1.28–1.42)	2.77 (2.52–3.04)	1.34 (1.29–1.39)	1.66 (1.54–1.8)	1.48 (1.34–1.63)	2.21 (1.84–2.65)
40.29	2.82 (2.7–2.94)	4.69 (4.33–5.08)	3.15 (2.97–3.33)	5.49 (4.91–6.14)	1.35 (1.28–1.43)	2.78 (2.53–3.05)	1.34 (1.29–1.4)	1.67 (1.54–1.81)	1.48 (1.34–1.64)	2.22 (1.85–2.66)
40.46	2.85 (2.74–2.97)	4.75 (4.38–5.15)	3.21 (3.03–3.4)	5.59 (5–6.26)	1.36 (1.28–1.43)	2.79 (2.54–3.06)	1.35 (1.3–1.4)	1.68 (1.55–1.82)	1.49 (1.35–1.65)	2.23 (1.85–2.68)
40.63	2.89 (2.77–3.02)	4.81 (4.44–5.21)	3.27 (3.08–3.46)	5.7 (5.09–6.38)	1.36 (1.29–1.44)	2.79 (2.54–3.07)	1.36 (1.3–1.41)	1.69 (1.56–1.83)	1.5 (1.35–1.66)	2.24 (1.86–2.69)
40.80	2.93 (2.8–3.06)	4.87 (4.49–5.28)	3.33 (3.13–3.53)	5.81 (5.18–6.5)	1.36 (1.29–1.44)	2.8 (2.55–3.08)	1.36 (1.31–1.42)	1.7 (1.57–1.84)	1.5 (1.35–1.67)	2.25 (1.87–2.7)
40.97	2.96 (2.84–3.1)	4.94 (4.55–5.35)	3.39 (3.19–3.6)	5.91 (5.28–6.63)	1.37 (1.29–1.45)	2.81 (2.56–3.09)	1.37 (1.32–1.43)	1.71 (1.58–1.85)	1.51 (1.36–1.68)	2.26 (1.87–2.72)
41.14	3 (2.87–3.14)	5 (4.61–5.42)	3.45 (3.25–3.67)	6.02 (5.38–6.75)	1.37 (1.3–1.45)	2.82 (2.56–3.1)	1.38 (1.32–1.44)	1.71 (1.58–1.86)	1.52 (1.36–1.69)	2.27 (1.88–2.73)
41.31	3.04 (2.91–3.18)	5.06 (4.66–5.49)	3.52 (3.3–3.75)	6.14 (5.47–6.88)	1.38 (1.3–1.46)	2.83 (2.57–3.11)	1.39 (1.33–1.45)	1.72 (1.59–1.87)	1.52 (1.36–1.7)	2.28 (1.89–2.75)

41.48	3.08 (2.94–3.22)	5.13 (4.72–5.57)	3.58 (3.36–3.82)	6.25 (5.57–7.01)	1.38 (1.3–1.47)	2.84 (2.58–3.12)	1.39 (1.34–1.46)	1.73 (1.6–1.88)	1.53 (1.37–1.71)	2.29 (1.89–2.76)
41.65	3.12 (2.98–3.27)	5.19 (4.78–5.64)	3.65 (3.42–3.89)	6.37 (5.67–7.15)	1.39 (1.3–1.47)	2.85 (2.58–3.14)	1.4 (1.34–1.46)	1.74 (1.61–1.89)	1.54 (1.37–1.72)	2.29 (1.9–2.77)
41.82	3.16 (3.01–3.31)	5.26 (4.84–5.72)	3.72 (3.48–3.97)	6.49 (5.78–7.29)	1.39 (1.31–1.48)	2.86 (2.59–3.15)	1.41 (1.35–1.47)	1.75 (1.62–1.9)	1.54 (1.38–1.73)	2.3 (1.91–2.79)
41.99	3.2 (3.05–3.36)	5.33 (4.9–5.79)	3.79 (3.54–4.05)	6.61 (5.88–7.43)	1.39 (1.31–1.48)	2.87 (2.6–3.16)	1.42 (1.35–1.48)	1.76 (1.62–1.91)	1.55 (1.38–1.74)	2.31 (1.91–2.8)
42.17	3.24 (3.09–3.4)	5.4 (4.96–5.87)	3.86 (3.61–4.13)	6.73 (5.99–7.57)	1.4 (1.31–1.49)	2.87 (2.61–3.17)	1.42 (1.36–1.49)	1.77 (1.63–1.92)	1.56 (1.38–1.75)	2.32 (1.92–2.82)
42.34	3.28 (3.12–3.45)	5.46 (5.02–5.94)	3.93 (3.67–4.21)	6.86 (6.1–7.72)	1.4 (1.32–1.5)	2.88 (2.61–3.18)	1.43 (1.37–1.5)	1.78 (1.64–1.93)	1.56 (1.39–1.76)	2.33 (1.92–2.83)
42.51	3.32 (3.16–3.5)	5.53 (5.09–6.02)	4.01 (3.74–4.29)	6.99 (6.21–7.86)	1.41 (1.32–1.5)	2.89 (2.62–3.19)	1.44 (1.37–1.51)	1.79 (1.65–1.94)	1.57 (1.39–1.77)	2.34 (1.93–2.85)
42.68	3.37 (3.2–3.54)	5.61 (5.15–6.1)	4.08 (3.8–4.38)	7.12 (6.32–8.02)	1.41 (1.32–1.51)	2.9 (2.63–3.21)	1.45 (1.38–1.52)	1.8 (1.66–1.95)	1.58 (1.39–1.78)	2.35 (1.94–2.86)
42.85	3.41 (3.24–3.59)	5.68 (5.21–6.18)	4.16 (3.87–4.46)	7.25 (6.44–8.17)	1.42 (1.32–1.52)	2.91 (2.63–3.22)	1.46 (1.39–1.53)	1.81 (1.67–1.97)	1.58 (1.4–1.79)	2.36 (1.94–2.88)
43.02	3.45 (3.28–3.64)	5.75 (5.28–6.27)	4.23 (3.94–4.55)	7.39 (6.55–8.33)	1.42 (1.33–1.52)	2.92 (2.64–3.23)	1.46 (1.39–1.54)	1.82 (1.67–1.98)	1.59 (1.4–1.8)	2.38 (1.95–2.89)
43.19	3.5 (3.32–3.69)	5.82 (5.34–6.35)	4.31 (4.01–4.64)	7.53 (6.67–8.49)	1.43 (1.33–1.53)	2.93 (2.65–3.24)	1.47 (1.4–1.55)	1.83 (1.68–1.99)	1.6 (1.4–1.81)	2.39 (1.96–2.91)
43.36	3.54 (3.36–3.74)	5.9 (5.41–6.43)	4.39 (4.08–4.73)	7.67 (6.8–8.65)	1.43 (1.33–1.53)	2.94 (2.65–3.25)	1.48 (1.41–1.56)	1.84 (1.69–2)	1.6 (1.41–1.82)	2.4 (1.96–2.92)
43.53	3.59 (3.4–3.79)	5.97 (5.48–6.52)	4.48 (4.15–4.83)	7.81 (6.92–8.82)	1.43 (1.34–1.54)	2.95 (2.66–3.27)	1.49 (1.41–1.57)	1.85 (1.7–2.01)	1.61 (1.41–1.84)	2.41 (1.97–2.94)
43.70	3.63 (3.44–3.84)	6.05 (5.54–6.6)	4.56 (4.22–4.92)	7.96 (7.04–8.99)	1.44 (1.34–1.55)	2.96 (2.67–3.28)	1.5 (1.42–1.57)	1.86 (1.71–2.02)	1.62 (1.42–1.85)	2.42 (1.98–2.95)
43.87	3.68 (3.48–3.89)	6.13 (5.61–6.69)	4.65 (4.3–5.02)	8.11 (7.17–9.16)	1.44 (1.34–1.55)	2.97 (2.67–3.29)	1.5 (1.43–1.58)	1.87 (1.72–2.03)	1.62 (1.42–1.86)	2.43 (1.98–2.97)
44.05	3.73 (3.52–3.95)	6.21 (5.68–6.78)	4.73 (4.38–5.12)	8.26 (7.3–9.34)	1.45 (1.35–1.56)	2.98 (2.68–3.3)	1.51 (1.43–1.59)	1.88 (1.72–2.05)	1.63 (1.42–1.87)	2.44 (1.99–2.99)
44.22	3.78 (3.57–4)	6.29 (5.75–6.87)	4.82 (4.45–5.22)	8.41 (7.43–9.52)	1.45 (1.35–1.56)	2.98 (2.69–3.32)	1.52 (1.44–1.6)	1.89 (1.73–2.06)	1.64 (1.43–1.88)	2.45 (1.99–3)
44.39	3.82 (3.61–4.05)	6.37 (5.82–6.96)	4.91 (4.53–5.32)	8.57 (7.57–9.7)	1.46 (1.35–1.57)	2.99 (2.69–3.33)	1.53 (1.45–1.61)	1.9 (1.74–2.07)	1.64 (1.43–1.89)	2.46 (2–3.02)
44.56	3.87 (3.65–4.11)	6.45 (5.9–7.05)	5 (4.61–5.43)	8.73 (7.71–9.89)	1.46 (1.35–1.58)	3 (2.7–3.34)	1.54 (1.45–1.62)	1.91 (1.75–2.08)	1.65 (1.43–1.9)	2.47 (2.01–3.03)
44.73	3.92 (3.7–4.16)	6.53 (5.97–7.15)	5.1 (4.69–5.53)	8.89 (7.85–10.08)	1.47 (1.36–1.58)	3.01 (2.71–3.35)	1.54 (1.46–1.63)	1.92 (1.76–2.09)	1.66 (1.44–1.91)	2.48 (2.01–3.05)
44.90	3.97 (3.74–4.22)	6.62 (6.04–7.24)	5.19 (4.78–5.64)	9.06 (7.99–10.28)	1.47 (1.36–1.59)	3.02 (2.71–3.37)	1.55 (1.47–1.64)	1.93 (1.77–2.11)	1.67 (1.44–1.93)	2.49 (2.02–3.07)
45.07	4.02 (3.79–4.28)	6.7 (6.12–7.34)	5.29 (4.86–5.75)	9.23 (8.13–10.48)	1.48 (1.36–1.6)	3.03 (2.72–3.38)	1.56 (1.47–1.65)	1.94 (1.78–2.12)	1.67 (1.44–1.94)	2.5 (2.03–3.08)
45.24	4.08 (3.83–4.34)	6.79 (6.19–7.44)	5.39 (4.95–5.87)	9.4 (8.28–10.68)	1.48 (1.37–1.6)	3.04 (2.73–3.39)	1.57 (1.48–1.66)	1.95 (1.79–2.13)	1.68 (1.45–1.95)	2.51 (2.03–3.1)
45.41	4.13 (3.88–4.39)	6.87 (6.27–7.53)	5.49 (5.04–5.98)	9.58 (8.43–10.88)	1.49 (1.37–1.61)	3.05 (2.73–3.4)	1.58 (1.49–1.67)	1.96 (1.79–2.14)	1.69 (1.45–1.96)	2.52 (2.04–3.12)
45.58	4.18 (3.93–4.45)	6.96 (6.35–7.63)	5.59 (5.13–6.1)	9.76 (8.58–11.09)	1.49 (1.37–1.62)	3.06 (2.74–3.42)	1.59 (1.5–1.68)	1.97 (1.8–2.15)	1.69 (1.46–1.97)	2.53 (2.05–3.13)
45.75	4.23 (3.97–4.51)	7.05 (6.43–7.74)	5.7 (5.22–6.22)	9.94 (8.74–11.31)	1.49 (1.38–1.62)	3.07 (2.75–3.43)	1.59 (1.5–1.69)	1.98 (1.81–2.17)	1.7 (1.46–1.98)	2.54 (2.05–3.15)
45.92	4.29 (4.02–4.57)	7.14 (6.5–7.84)	5.8 (5.31–6.34)	10.13 (8.9–11.53)	1.5 (1.38–1.63)	3.08 (2.76–3.44)	1.6 (1.51–1.7)	1.99 (1.82–2.18)	1.71 (1.46–2)	2.55 (2.06–3.17)

46.10	4.34 (4.07–4.64)	7.23 (6.58–7.94)	5.91 (5.4–6.47)	10.32 (9.06–11.75)	1.5 (1.38–1.64)	3.09 (2.76–3.46)	1.61 (1.52–1.71)	2 (1.83–2.19)	1.72 (1.47–2.01)	2.57 (2.07–3.19)
46.27	4.4 (4.12–4.7)	7.32 (6.67–8.05)	6.02 (5.5–6.6)	10.51 (9.22–11.98)	1.51 (1.39–1.64)	3.1 (2.77–3.47)	1.62 (1.52–1.72)	2.01 (1.84–2.21)	1.72 (1.47–2.02)	2.58 (2.07–3.2)
46.44	4.46 (4.17–4.76)	7.42 (6.75–8.16)	6.14 (5.6–6.73)	10.71 (9.39–12.21)	1.51 (1.39–1.65)	3.11 (2.78–3.48)	1.63 (1.53–1.73)	2.03 (1.85–2.22)	1.73 (1.47–2.03)	2.59 (2.08–3.22)
46.61	4.51 (4.22–4.83)	7.51 (6.83–8.26)	6.25 (5.7–6.86)	10.91 (9.56–12.45)	1.52 (1.39–1.66)	3.12 (2.78–3.5)	1.64 (1.54–1.74)	2.04 (1.86–2.23)	1.74 (1.48–2.04)	2.6 (2.09–3.24)
46.78	4.57 (4.27–4.89)	7.61 (6.92–8.37)	6.37 (5.8–6.99)	11.11 (9.73–12.69)	1.52 (1.39–1.66)	3.13 (2.79–3.51)	1.65 (1.55–1.75)	2.05 (1.87–2.24)	1.75 (1.48–2.06)	2.61 (2.09–3.26)
46.95	4.63 (4.32–4.96)	7.71 (7–8.49)	6.49 (5.9–7.13)	11.32 (9.91–12.93)	1.53 (1.4–1.67)	3.14 (2.8–3.52)	1.66 (1.55–1.77)	2.06 (1.88–2.26)	1.75 (1.49–2.07)	2.62 (2.1–3.27)
47.12	4.69 (4.37–5.03)	7.81 (7.09–8.6)	6.61 (6–7.27)	11.53 (10.08–13.19)	1.53 (1.4–1.68)	3.15 (2.8–3.54)	1.66 (1.56–1.78)	2.07 (1.89–2.27)	1.76 (1.49–2.08)	2.63 (2.1–3.29)
47.29	4.75 (4.43–5.09)	7.91 (7.17–8.71)	6.73 (6.11–7.42)	11.75 (10.27–13.44)	1.54 (1.4–1.68)	3.16 (2.81–3.55)	1.67 (1.57–1.79)	2.08 (1.9–2.28)	1.77 (1.49–2.09)	2.64 (2.11–3.31)
47.46	4.81 (4.48–5.16)	8.01 (7.26–8.83)	6.86 (6.22–7.56)	11.97 (10.45–13.7)	1.54 (1.41–1.69)	3.17 (2.82–3.56)	1.68 (1.57–1.8)	2.09 (1.9–2.3)	1.78 (1.5–2.11)	2.66 (2.12–3.33)
47.63	4.87 (4.54–5.23)	8.11 (7.35–8.95)	6.99 (6.33–7.71)	12.19 (10.64–13.97)	1.55 (1.41–1.7)	3.18 (2.82–3.58)	1.69 (1.58–1.81)	2.1 (1.91–2.31)	1.78 (1.5–2.12)	2.67 (2.12–3.35)
47.80	4.93 (4.59–5.3)	8.21 (7.44–9.07)	7.12 (6.44–7.86)	12.42 (10.83–14.24)	1.55 (1.41–1.7)	3.19 (2.83–3.59)	1.7 (1.59–1.82)	2.11 (1.92–2.32)	1.79 (1.51–2.13)	2.68 (2.13–3.37)
47.97	5 (4.65–5.38)	8.32 (7.53–9.19)	7.25 (6.56–8.02)	12.65 (11.03–14.51)	1.56 (1.42–1.71)	3.2 (2.84–3.6)	1.71 (1.6–1.83)	2.13 (1.93–2.34)	1.8 (1.51–2.14)	2.69 (2.14–3.38)
48.15	5.06 (4.7–5.45)	8.43 (7.63–9.31)	7.39 (6.67–8.18)	12.89 (11.23–14.8)	1.56 (1.42–1.72)	3.21 (2.85–3.62)	1.72 (1.6–1.84)	2.14 (1.94–2.35)	1.81 (1.51–2.16)	2.7 (2.14–3.4)
48.32	5.13 (4.76–5.52)	8.53 (7.72–9.43)	7.52 (6.79–8.34)	13.13 (11.43–15.08)	1.57 (1.42–1.72)	3.22 (2.85–3.63)	1.73 (1.61–1.85)	2.15 (1.95–2.36)	1.81 (1.52–2.17)	2.71 (2.15–3.42)
48.49	5.19 (4.82–5.6)	8.64 (7.82–9.56)	7.67 (6.91–8.5)	13.38 (11.63–15.38)	1.57 (1.43–1.73)	3.23 (2.86–3.65)	1.74 (1.62–1.86)	2.16 (1.96–2.38)	1.82 (1.52–2.18)	2.72 (2.16–3.44)
48.66	5.26 (4.87–5.67)	8.75 (7.91–9.69)	7.81 (7.03–8.67)	13.63 (11.84–15.67)	1.58 (1.43–1.74)	3.24 (2.87–3.66)	1.75 (1.63–1.87)	2.17 (1.97–2.39)	1.83 (1.53–2.2)	2.74 (2.16–3.46)
48.83	5.33 (4.93–5.75)	8.87 (8.01–9.82)	7.96 (7.16–8.84)	13.88 (12.06–15.98)	1.58 (1.43–1.75)	3.25 (2.87–3.67)	1.76 (1.64–1.89)	2.18 (1.98–2.41)	1.84 (1.53–2.21)	2.75 (2.17–3.48)
49.00	5.39 (4.99–5.83)	8.98 (8.11–9.95)	8.1 (7.28–9.02)	14.14 (12.28–16.29)	1.59 (1.44–1.75)	3.26 (2.88–3.69)	1.77 (1.64–1.9)	2.19 (1.99–2.42)	1.85 (1.53–2.22)	2.76 (2.18–3.5)

Continuation of Table S10

Age	Polyhydramnios		Placenta previa		Cesarean section		Postpartum hemorrhage		Premature	
	SC	ART	SC	ART	SC	ART	SC	ART	SC	ART
15.00	0.39 (0.32–0.49)	0.53 (0.39–0.73)	0.25 (0.21–0.3)	0.35 (0.28–0.45)	0.25 (0.24–0.26)	0.55 (0.5–0.6)	0.67 (0.61–0.73)	1.4 (1.23–1.59)	1.53 (1.41–1.65)	2.13 (1.87–2.43)
15.17	0.4 (0.32–0.5)	0.54 (0.4–0.73)	0.26 (0.22–0.3)	0.36 (0.29–0.45)	0.25 (0.24–0.27)	0.56 (0.51–0.61)	0.67 (0.62–0.73)	1.41 (1.24–1.6)	1.51 (1.4–1.64)	2.12 (1.86–2.41)
15.34	0.4 (0.33–0.5)	0.55 (0.41–0.74)	0.26 (0.22–0.31)	0.37 (0.29–0.46)	0.26 (0.25–0.27)	0.57 (0.52–0.62)	0.68 (0.62–0.74)	1.42 (1.25–1.61)	1.51 (1.39–1.63)	2.1 (1.85–2.4)
15.51	0.41 (0.33–0.51)	0.56 (0.41–0.75)	0.27 (0.22–0.31)	0.37 (0.3–0.47)	0.26 (0.25–0.28)	0.58 (0.53–0.63)	0.68 (0.62–0.74)	1.42 (1.26–1.61)	1.5 (1.38–1.62)	2.09 (1.84–2.38)
15.68	0.41 (0.34–0.51)	0.56 (0.42–0.76)	0.27 (0.23–0.32)	0.38 (0.3–0.48)	0.27 (0.26–0.28)	0.59 (0.54–0.64)	0.68 (0.63–0.74)	1.43 (1.26–1.62)	1.49 (1.38–1.6)	2.08 (1.83–2.36)
15.85	0.42 (0.34–0.51)	0.57 (0.42–0.76)	0.28 (0.23–0.32)	0.39 (0.31–0.48)	0.27 (0.26–0.29)	0.6 (0.55–0.65)	0.69 (0.63–0.75)	1.44 (1.27–1.63)	1.48 (1.37–1.59)	2.06 (1.82–2.34)
16.03	0.43 (0.35–0.52)	0.58 (0.43–0.77)	0.28 (0.24–0.33)	0.39 (0.32–0.49)	0.28 (0.27–0.29)	0.61 (0.57–0.67)	0.69 (0.64–0.75)	1.45 (1.28–1.63)	1.47 (1.36–1.58)	2.05 (1.81–2.33)
16.20	0.43 (0.35–0.52)	0.58 (0.44–0.78)	0.29 (0.24–0.33)	0.4 (0.32–0.5)	0.28 (0.27–0.3)	0.62 (0.58–0.68)	0.69 (0.64–0.75)	1.45 (1.29–1.64)	1.46 (1.35–1.57)	2.04 (1.79–2.31)
16.37	0.44 (0.36–0.53)	0.59 (0.44–0.79)	0.29 (0.25–0.34)	0.41 (0.33–0.51)	0.29 (0.28–0.3)	0.64 (0.59–0.69)	0.7 (0.64–0.75)	1.46 (1.3–1.65)	1.45 (1.35–1.55)	2.02 (1.78–2.29)
16.54	0.44 (0.36–0.53)	0.6 (0.45–0.8)	0.3 (0.25–0.35)	0.42 (0.34–0.52)	0.29 (0.28–0.31)	0.65 (0.6–0.7)	0.7 (0.65–0.76)	1.47 (1.3–1.65)	1.44 (1.34–1.54)	2.01 (1.77–2.28)
16.71	0.45 (0.37–0.54)	0.61 (0.46–0.8)	0.3 (0.26–0.35)	0.42 (0.34–0.52)	0.3 (0.29–0.31)	0.66 (0.61–0.72)	0.7 (0.65–0.76)	1.48 (1.31–1.66)	1.43 (1.33–1.53)	2 (1.76–2.26)
16.88	0.45 (0.38–0.54)	0.61 (0.46–0.81)	0.31 (0.26–0.36)	0.43 (0.35–0.53)	0.31 (0.29–0.32)	0.67 (0.62–0.73)	0.71 (0.66–0.76)	1.48 (1.32–1.67)	1.42 (1.32–1.52)	1.98 (1.75–2.24)
17.05	0.46 (0.38–0.55)	0.62 (0.47–0.82)	0.31 (0.27–0.36)	0.44 (0.36–0.54)	0.31 (0.3–0.32)	0.69 (0.63–0.74)	0.71 (0.66–0.77)	1.49 (1.33–1.68)	1.41 (1.32–1.51)	1.97 (1.74–2.23)
17.22	0.46 (0.39–0.55)	0.63 (0.48–0.83)	0.32 (0.28–0.37)	0.45 (0.37–0.55)	0.32 (0.3–0.33)	0.7 (0.65–0.75)	0.72 (0.66–0.77)	1.5 (1.34–1.68)	1.4 (1.31–1.49)	1.96 (1.73–2.21)
17.39	0.47 (0.39–0.56)	0.64 (0.48–0.84)	0.32 (0.28–0.37)	0.46 (0.37–0.56)	0.32 (0.31–0.34)	0.71 (0.66–0.77)	0.72 (0.67–0.77)	1.51 (1.34–1.69)	1.39 (1.3–1.48)	1.94 (1.72–2.2)
17.56	0.48 (0.4–0.56)	0.64 (0.49–0.85)	0.33 (0.29–0.38)	0.47 (0.38–0.57)	0.33 (0.32–0.34)	0.72 (0.67–0.78)	0.72 (0.67–0.78)	1.51 (1.35–1.7)	1.38 (1.3–1.47)	1.93 (1.71–2.18)
17.73	0.48 (0.41–0.57)	0.65 (0.5–0.85)	0.34 (0.29–0.39)	0.47 (0.39–0.58)	0.33 (0.32–0.35)	0.74 (0.68–0.8)	0.73 (0.68–0.78)	1.52 (1.36–1.7)	1.37 (1.29–1.46)	1.92 (1.7–2.16)
17.90	0.49 (0.41–0.58)	0.66 (0.5–0.86)	0.34 (0.3–0.39)	0.48 (0.4–0.59)	0.34 (0.33–0.35)	0.75 (0.7–0.81)	0.73 (0.68–0.78)	1.53 (1.37–1.71)	1.36 (1.28–1.45)	1.91 (1.69–2.15)
18.08	0.49 (0.42–0.58)	0.67 (0.51–0.87)	0.35 (0.31–0.4)	0.49 (0.4–0.6)	0.35 (0.33–0.36)	0.77 (0.71–0.83)	0.73 (0.69–0.78)	1.54 (1.38–1.72)	1.35 (1.27–1.44)	1.89 (1.68–2.13)
18.25	0.5 (0.43–0.59)	0.68 (0.52–0.88)	0.36 (0.31–0.4)	0.5 (0.41–0.61)	0.35 (0.34–0.37)	0.78 (0.72–0.84)	0.74 (0.69–0.79)	1.55 (1.38–1.73)	1.35 (1.27–1.43)	1.88 (1.67–2.12)
18.42	0.51 (0.43–0.59)	0.68 (0.53–0.89)	0.36 (0.32–0.41)	0.51 (0.42–0.62)	0.36 (0.35–0.37)	0.79 (0.74–0.86)	0.74 (0.7–0.79)	1.55 (1.39–1.73)	1.34 (1.26–1.42)	1.87 (1.66–2.1)
18.59	0.51 (0.44–0.6)	0.69 (0.53–0.9)	0.37 (0.33–0.42)	0.52 (0.43–0.63)	0.37 (0.35–0.38)	0.81 (0.75–0.87)	0.75 (0.7–0.79)	1.56 (1.4–1.74)	1.33 (1.25–1.4)	1.86 (1.65–2.09)
18.76	0.52 (0.45–0.6)	0.7 (0.54–0.91)	0.38 (0.33–0.42)	0.53 (0.44–0.64)	0.37 (0.36–0.39)	0.82 (0.76–0.89)	0.75 (0.7–0.8)	1.57 (1.41–1.75)	1.32 (1.25–1.39)	1.84 (1.64–2.07)
18.93	0.52 (0.45–0.61)	0.71 (0.55–0.92)	0.38 (0.34–0.43)	0.54 (0.45–0.65)	0.38 (0.37–0.39)	0.84 (0.78–0.9)	0.75 (0.71–0.8)	1.58 (1.42–1.76)	1.31 (1.24–1.38)	1.83 (1.63–2.06)

19.10	0.53 (0.46–0.61)	0.72 (0.56–0.93)	0.39 (0.35–0.44)	0.55 (0.46–0.66)	0.39 (0.38–0.4)	0.85 (0.79–0.92)	0.76 (0.71–0.8)	1.59 (1.43–1.77)	1.3 (1.23–1.37)	1.82 (1.62–2.04)
19.27	0.54 (0.47–0.62)	0.73 (0.57–0.94)	0.4 (0.35–0.44)	0.56 (0.46–0.67)	0.4 (0.38–0.41)	0.87 (0.81–0.94)	0.76 (0.72–0.81)	1.6 (1.44–1.77)	1.29 (1.23–1.36)	1.81 (1.61–2.03)
19.44	0.54 (0.47–0.63)	0.74 (0.57–0.95)	0.4 (0.36–0.45)	0.57 (0.47–0.68)	0.4 (0.39–0.42)	0.89 (0.82–0.95)	0.77 (0.72–0.81)	1.6 (1.44–1.78)	1.28 (1.22–1.35)	1.8 (1.6–2.01)
19.61	0.55 (0.48–0.63)	0.75 (0.58–0.96)	0.41 (0.37–0.46)	0.58 (0.48–0.69)	0.41 (0.4–0.42)	0.9 (0.84–0.97)	0.77 (0.73–0.81)	1.61 (1.45–1.79)	1.28 (1.21–1.34)	1.78 (1.59–2)
19.78	0.56 (0.49–0.64)	0.76 (0.59–0.97)	0.42 (0.38–0.47)	0.59 (0.49–0.71)	0.42 (0.41–0.43)	0.92 (0.85–0.99)	0.77 (0.73–0.82)	1.62 (1.46–1.8)	1.27 (1.21–1.33)	1.77 (1.58–1.98)
19.95	0.56 (0.5–0.64)	0.77 (0.6–0.98)	0.43 (0.38–0.47)	0.6 (0.5–0.72)	0.43 (0.41–0.44)	0.94 (0.87–1.01)	0.78 (0.74–0.82)	1.63 (1.47–1.81)	1.26 (1.2–1.32)	1.76 (1.57–1.97)
20.13	0.57 (0.5–0.65)	0.77 (0.61–0.99)	0.43 (0.39–0.48)	0.61 (0.51–0.73)	0.43 (0.42–0.45)	0.95 (0.89–1.03)	0.78 (0.74–0.82)	1.64 (1.48–1.81)	1.25 (1.19–1.31)	1.75 (1.56–1.96)
20.30	0.58 (0.51–0.65)	0.78 (0.62–1)	0.44 (0.4–0.49)	0.62 (0.52–0.74)	0.44 (0.43–0.45)	0.97 (0.9–1.04)	0.79 (0.75–0.83)	1.65 (1.49–1.82)	1.24 (1.19–1.3)	1.74 (1.55–1.94)
20.47	0.59 (0.52–0.66)	0.79 (0.62–1.01)	0.45 (0.41–0.5)	0.63 (0.53–0.75)	0.45 (0.44–0.46)	0.99 (0.92–1.06)	0.79 (0.75–0.83)	1.65 (1.5–1.83)	1.23 (1.18–1.29)	1.73 (1.54–1.93)
20.64	0.59 (0.53–0.67)	0.8 (0.63–1.02)	0.46 (0.42–0.5)	0.65 (0.54–0.77)	0.46 (0.45–0.47)	1.01 (0.94–1.08)	0.79 (0.76–0.83)	1.66 (1.5–1.84)	1.23 (1.17–1.28)	1.71 (1.54–1.92)
20.81	0.6 (0.54–0.67)	0.81 (0.64–1.03)	0.47 (0.43–0.51)	0.66 (0.55–0.78)	0.47 (0.45–0.48)	1.03 (0.96–1.1)	0.8 (0.76–0.84)	1.67 (1.51–1.85)	1.22 (1.17–1.27)	1.7 (1.53–1.9)
20.98	0.61 (0.54–0.68)	0.82 (0.65–1.04)	0.48 (0.44–0.52)	0.67 (0.57–0.79)	0.48 (0.46–0.49)	1.05 (0.97–1.12)	0.8 (0.77–0.84)	1.68 (1.52–1.85)	1.21 (1.16–1.26)	1.69 (1.52–1.89)
21.15	0.62 (0.55–0.69)	0.83 (0.66–1.05)	0.49 (0.44–0.53)	0.68 (0.58–0.81)	0.48 (0.47–0.5)	1.07 (0.99–1.14)	0.81 (0.77–0.84)	1.69 (1.53–1.86)	1.2 (1.16–1.25)	1.68 (1.51–1.87)
21.32	0.62 (0.56–0.69)	0.84 (0.67–1.07)	0.49 (0.45–0.54)	0.7 (0.59–0.82)	0.49 (0.48–0.5)	1.09 (1.01–1.16)	0.81 (0.78–0.85)	1.7 (1.54–1.87)	1.19 (1.15–1.24)	1.67 (1.5–1.86)
21.49	0.63 (0.57–0.7)	0.86 (0.68–1.08)	0.5 (0.46–0.55)	0.71 (0.6–0.84)	0.5 (0.49–0.51)	1.11 (1.03–1.19)	0.81 (0.78–0.85)	1.71 (1.55–1.88)	1.19 (1.14–1.23)	1.66 (1.49–1.85)
21.66	0.64 (0.58–0.7)	0.87 (0.69–1.09)	0.51 (0.47–0.55)	0.72 (0.61–0.85)	0.51 (0.5–0.52)	1.13 (1.05–1.21)	0.82 (0.79–0.85)	1.72 (1.56–1.89)	1.18 (1.14–1.22)	1.65 (1.48–1.84)
21.83	0.65 (0.59–0.71)	0.88 (0.7–1.1)	0.52 (0.48–0.56)	0.73 (0.62–0.86)	0.52 (0.51–0.53)	1.15 (1.07–1.23)	0.82 (0.79–0.86)	1.72 (1.57–1.9)	1.17 (1.13–1.21)	1.64 (1.47–1.82)
22.01	0.66 (0.6–0.72)	0.89 (0.71–1.11)	0.53 (0.49–0.57)	0.75 (0.64–0.88)	0.53 (0.52–0.54)	1.17 (1.09–1.25)	0.83 (0.8–0.86)	1.73 (1.58–1.91)	1.16 (1.12–1.2)	1.63 (1.46–1.81)
22.18	0.66 (0.61–0.72)	0.9 (0.72–1.13)	0.54 (0.5–0.58)	0.76 (0.65–0.89)	0.54 (0.53–0.55)	1.19 (1.11–1.28)	0.83 (0.8–0.86)	1.74 (1.59–1.92)	1.16 (1.12–1.19)	1.62 (1.45–1.8)
22.35	0.67 (0.62–0.73)	0.91 (0.73–1.14)	0.55 (0.51–0.59)	0.78 (0.66–0.91)	0.55 (0.54–0.56)	1.21 (1.13–1.3)	0.84 (0.81–0.87)	1.75 (1.59–1.92)	1.15 (1.11–1.18)	1.61 (1.44–1.78)
22.52	0.68 (0.63–0.74)	0.92 (0.74–1.15)	0.56 (0.53–0.6)	0.79 (0.67–0.93)	0.56 (0.55–0.57)	1.23 (1.15–1.32)	0.84 (0.81–0.87)	1.76 (1.6–1.93)	1.14 (1.11–1.18)	1.59 (1.43–1.77)
22.69	0.69 (0.64–0.75)	0.93 (0.75–1.16)	0.57 (0.54–0.61)	0.8 (0.69–0.94)	0.57 (0.56–0.58)	1.26 (1.17–1.35)	0.84 (0.82–0.87)	1.77 (1.61–1.94)	1.13 (1.1–1.17)	1.58 (1.43–1.76)
22.86	0.7 (0.65–0.75)	0.94 (0.76–1.18)	0.58 (0.55–0.62)	0.82 (0.7–0.96)	0.58 (0.57–0.59)	1.28 (1.2–1.37)	0.85 (0.82–0.88)	1.78 (1.62–1.95)	1.13 (1.09–1.16)	1.57 (1.42–1.75)
23.03	0.71 (0.66–0.76)	0.96 (0.77–1.19)	0.59 (0.56–0.63)	0.83 (0.72–0.97)	0.59 (0.58–0.6)	1.3 (1.22–1.4)	0.85 (0.83–0.88)	1.79 (1.63–1.96)	1.12 (1.09–1.15)	1.56 (1.41–1.74)
23.20	0.71 (0.67–0.77)	0.97 (0.78–1.2)	0.6 (0.57–0.64)	0.85 (0.73–0.99)	0.6 (0.59–0.61)	1.33 (1.24–1.42)	0.86 (0.83–0.88)	1.8 (1.64–1.97)	1.11 (1.08–1.14)	1.55 (1.4–1.72)
23.37	0.72 (0.68–0.77)	0.98 (0.79–1.22)	0.62 (0.58–0.65)	0.87 (0.74–1.01)	0.61 (0.61–0.62)	1.35 (1.27–1.45)	0.86 (0.84–0.89)	1.81 (1.65–1.98)	1.1 (1.08–1.13)	1.54 (1.39–1.71)
23.54	0.73 (0.69–0.78)	0.99 (0.8–1.23)	0.63 (0.6–0.66)	0.88 (0.76–1.03)	0.63 (0.62–0.64)	1.38 (1.29–1.48)	0.87 (0.84–0.89)	1.82 (1.66–1.99)	1.1 (1.07–1.12)	1.53 (1.38–1.7)

23.71	0.74 (0.7–0.79)	1 (0.81–1.24)	0.64 (0.61–0.67)	0.9 (0.77–1.04)	0.64 (0.63–0.65)	1.4 (1.31–1.5)	0.87 (0.85–0.89)	1.83 (1.67–2)	1.09 (1.06–1.11)	1.52 (1.37–1.69)
23.88	0.75 (0.71–0.8)	1.02 (0.82–1.26)	0.65 (0.62–0.68)	0.91 (0.79–1.06)	0.65 (0.64–0.66)	1.43 (1.34–1.53)	0.88 (0.86–0.9)	1.84 (1.68–2.01)	1.08 (1.06–1.11)	1.51 (1.36–1.68)
24.06	0.76 (0.72–0.8)	1.03 (0.83–1.27)	0.66 (0.63–0.69)	0.93 (0.8–1.08)	0.66 (0.65–0.67)	1.46 (1.36–1.56)	0.88 (0.86–0.9)	1.84 (1.69–2.02)	1.08 (1.05–1.1)	1.5 (1.36–1.67)
24.23	0.77 (0.73–0.81)	1.04 (0.84–1.29)	0.67 (0.65–0.7)	0.95 (0.82–1.1)	0.67 (0.67–0.68)	1.48 (1.39–1.59)	0.88 (0.87–0.9)	1.85 (1.7–2.03)	1.07 (1.05–1.09)	1.49 (1.35–1.66)
24.40	0.78 (0.74–0.82)	1.05 (0.85–1.3)	0.69 (0.66–0.71)	0.97 (0.83–1.12)	0.69 (0.68–0.69)	1.51 (1.41–1.62)	0.89 (0.87–0.91)	1.86 (1.71–2.04)	1.06 (1.04–1.08)	1.49 (1.34–1.65)
24.57	0.79 (0.75–0.83)	1.07 (0.87–1.32)	0.7 (0.67–0.73)	0.98 (0.85–1.14)	0.7 (0.69–0.71)	1.54 (1.44–1.65)	0.89 (0.88–0.91)	1.87 (1.72–2.05)	1.06 (1.04–1.07)	1.48 (1.33–1.64)
24.74	0.8 (0.76–0.83)	1.08 (0.88–1.33)	0.71 (0.69–0.74)	1 (0.87–1.16)	0.71 (0.71–0.72)	1.57 (1.47–1.68)	0.9 (0.88–0.91)	1.88 (1.73–2.06)	1.05 (1.03–1.07)	1.47 (1.33–1.63)
24.91	0.81 (0.78–0.84)	1.09 (0.89–1.35)	0.73 (0.7–0.75)	1.02 (0.88–1.18)	0.73 (0.72–0.73)	1.6 (1.49–1.71)	0.9 (0.89–0.92)	1.89 (1.73–2.07)	1.04 (1.03–1.06)	1.46 (1.32–1.62)
25.08	0.82 (0.79–0.85)	1.11 (0.9–1.36)	0.74 (0.72–0.76)	1.04 (0.9–1.2)	0.74 (0.73–0.74)	1.63 (1.52–1.74)	0.91 (0.89–0.92)	1.9 (1.74–2.08)	1.04 (1.02–1.05)	1.45 (1.31–1.61)
25.25	0.83 (0.8–0.86)	1.12 (0.91–1.38)	0.75 (0.73–0.77)	1.06 (0.92–1.22)	0.75 (0.75–0.76)	1.66 (1.55–1.77)	0.91 (0.9–0.93)	1.91 (1.75–2.09)	1.03 (1.02–1.05)	1.45 (1.31–1.6)
25.42	0.84 (0.81–0.86)	1.13 (0.92–1.4)	0.77 (0.75–0.79)	1.08 (0.93–1.24)	0.77 (0.76–0.77)	1.69 (1.58–1.8)	0.92 (0.91–0.93)	1.92 (1.76–2.1)	1.03 (1.02–1.04)	1.44 (1.3–1.59)
25.59	0.85 (0.82–0.87)	1.15 (0.93–1.41)	0.78 (0.76–0.8)	1.1 (0.95–1.27)	0.78 (0.77–0.78)	1.72 (1.61–1.83)	0.92 (0.91–0.93)	1.94 (1.77–2.11)	1.02 (1.01–1.03)	1.43 (1.29–1.58)
25.76	0.86 (0.84–0.88)	1.16 (0.95–1.43)	0.79 (0.78–0.81)	1.12 (0.97–1.29)	0.79 (0.79–0.8)	1.75 (1.64–1.87)	0.93 (0.92–0.94)	1.95 (1.78–2.12)	1.02 (1.01–1.03)	1.43 (1.29–1.58)
25.93	0.87 (0.85–0.89)	1.18 (0.96–1.45)	0.81 (0.79–0.82)	1.14 (0.99–1.31)	0.81 (0.8–0.81)	1.78 (1.67–1.9)	0.93 (0.92–0.94)	1.96 (1.79–2.13)	1.02 (1.01–1.02)	1.42 (1.28–1.57)
26.11	0.88 (0.86–0.9)	1.19 (0.97–1.46)	0.82 (0.81–0.84)	1.16 (1–1.33)	0.82 (0.82–0.83)	1.81 (1.7–1.93)	0.94 (0.93–0.95)	1.97 (1.8–2.14)	1.01 (1–1.02)	1.42 (1.28–1.56)
26.28	0.89 (0.87–0.91)	1.21 (0.98–1.48)	0.84 (0.82–0.85)	1.18 (1.02–1.36)	0.84 (0.83–0.84)	1.84 (1.73–1.97)	0.94 (0.94–0.95)	1.98 (1.81–2.16)	1.01 (1–1.02)	1.41 (1.28–1.56)
26.45	0.9 (0.88–0.92)	1.22 (0.99–1.5)	0.85 (0.84–0.86)	1.2 (1.04–1.38)	0.85 (0.85–0.86)	1.88 (1.76–2.01)	0.95 (0.94–0.96)	1.99 (1.82–2.17)	1.01 (1–1.01)	1.41 (1.27–1.55)
26.62	0.91 (0.9–0.92)	1.23 (1.01–1.51)	0.87 (0.86–0.88)	1.22 (1.06–1.41)	0.87 (0.87–0.87)	1.91 (1.79–2.04)	0.95 (0.95–0.96)	2 (1.84–2.18)	1 (1–1.01)	1.4 (1.27–1.55)
26.79	0.92 (0.91–0.93)	1.25 (1.02–1.53)	0.88 (0.87–0.89)	1.24 (1.08–1.43)	0.88 (0.88–0.89)	1.95 (1.82–2.08)	0.96 (0.95–0.96)	2.01 (1.85–2.19)	1 (1–1.01)	1.4 (1.27–1.55)
26.96	0.93 (0.92–0.94)	1.26 (1.03–1.55)	0.9 (0.89–0.91)	1.26 (1.1–1.46)	0.9 (0.9–0.9)	1.98 (1.86–2.11)	0.97 (0.96–0.97)	2.02 (1.86–2.2)	1 (1–1)	1.4 (1.26–1.54)
27.13	0.94 (0.94–0.95)	1.28 (1.04–1.57)	0.92 (0.91–0.92)	1.29 (1.12–1.48)	0.92 (0.91–0.92)	2.02 (1.89–2.15)	0.97 (0.97–0.97)	2.03 (1.87–2.22)	1 (1–1)	1.4 (1.26–1.54)
27.30	0.95 (0.95–0.96)	1.29 (1.06–1.58)	0.93 (0.93–0.94)	1.31 (1.14–1.51)	0.93 (0.93–0.93)	2.05 (1.92–2.19)	0.98 (0.97–0.98)	2.05 (1.88–2.23)	1 (1–1)	1.4 (1.26–1.54)
27.47	0.97 (0.96–0.97)	1.31 (1.07–1.6)	0.95 (0.94–0.95)	1.33 (1.16–1.53)	0.95 (0.95–0.95)	2.09 (1.96–2.23)	0.98 (0.98–0.98)	2.06 (1.89–2.24)	1 (1–1)	1.39 (1.26–1.54)
27.64	0.98 (0.97–0.98)	1.32 (1.08–1.62)	0.96 (0.96–0.97)	1.36 (1.18–1.56)	0.96 (0.96–0.97)	2.12 (1.99–2.27)	0.99 (0.99–0.99)	2.07 (1.9–2.25)	1 (1–1)	1.4 (1.26–1.54)
27.81	0.99 (0.99–0.99)	1.34 (1.09–1.64)	0.98 (0.98–0.98)	1.38 (1.2–1.59)	0.98 (0.98–0.98)	2.16 (2.02–2.31)	0.99 (0.99–0.99)	2.08 (1.91–2.27)	1 (1–1)	1.4 (1.26–1.54)
27.98	1 (1–1)	1.35 (1.11–1.66)	1 (1–1)	1.4 (1.22–1.62)	1 (1–1)	2.2 (2.06–2.35)	1 (1–1)	2.09 (1.92–2.28)	1 (1–1)	1.4 (1.27–1.54)
**28.00**	**Reference**	**Reference**	**Reference**	**Reference**	**Reference**	**Reference**	**Reference**	**Reference**	**Reference**	**Reference**

28.16	1.01 (1.01–1.01)	1.37 (1.12–1.68)	1.02 (1.01–1.02)	1.43 (1.24–1.64)	1.02 (1.02–1.02)	2.24 (2.1–2.39)	1.01 (1–1.01)	2.11 (1.94–2.29)	1 (1–1)	1.4 (1.27–1.55)
28.33	1.02 (1.02–1.02)	1.38 (1.13–1.69)	1.03 (1.03–1.04)	1.45 (1.27–1.67)	1.03 (1.03–1.03)	2.28 (2.13–2.43)	1.01 (1.01–1.01)	2.12 (1.95–2.31)	1 (1–1.01)	1.4 (1.27–1.55)
28.50	1.03 (1.03–1.04)	1.4 (1.14–1.71)	1.05 (1.05–1.05)	1.48 (1.29–1.7)	1.05 (1.05–1.05)	2.31 (2.17–2.47)	1.02 (1.02–1.02)	2.13 (1.96–2.32)	1.01 (1.01–1.01)	1.41 (1.28–1.56)
28.67	1.04 (1.04–1.05)	1.42 (1.16–1.73)	1.07 (1.06–1.07)	1.5 (1.31–1.73)	1.07 (1.07–1.07)	2.35 (2.21–2.51)	1.02 (1.02–1.03)	2.15 (1.97–2.34)	1.01 (1.01–1.01)	1.41 (1.28–1.56)
28.84	1.06 (1.05–1.06)	1.43 (1.17–1.75)	1.09 (1.08–1.09)	1.53 (1.33–1.76)	1.09 (1.09–1.09)	2.39 (2.24–2.55)	1.03 (1.03–1.03)	2.16 (1.98–2.35)	1.02 (1.01–1.02)	1.42 (1.29–1.57)
29.01	1.07 (1.06–1.08)	1.45 (1.18–1.77)	1.11 (1.1–1.11)	1.56 (1.35–1.79)	1.11 (1.1–1.11)	2.43 (2.28–2.6)	1.04 (1.03–1.04)	2.17 (2–2.37)	1.02 (1.02–1.02)	1.43 (1.29–1.57)
29.18	1.08 (1.07–1.09)	1.46 (1.2–1.79)	1.13 (1.12–1.13)	1.58 (1.38–1.82)	1.12 (1.12–1.13)	2.47 (2.32–2.64)	1.04 (1.04–1.05)	2.19 (2.01–2.38)	1.02 (1.02–1.03)	1.43 (1.3–1.58)
29.35	1.09 (1.08–1.1)	1.48 (1.21–1.81)	1.14 (1.14–1.15)	1.61 (1.4–1.85)	1.14 (1.14–1.15)	2.52 (2.36–2.68)	1.05 (1.05–1.05)	2.2 (2.02–2.4)	1.03 (1.03–1.03)	1.44 (1.31–1.59)
29.52	1.1 (1.09–1.11)	1.49 (1.22–1.83)	1.16 (1.15–1.17)	1.64 (1.43–1.88)	1.16 (1.16–1.16)	2.56 (2.4–2.73)	1.06 (1.05–1.06)	2.22 (2.04–2.41)	1.04 (1.03–1.04)	1.45 (1.31–1.6)
29.69	1.11 (1.1–1.13)	1.51 (1.23–1.85)	1.18 (1.17–1.19)	1.66 (1.45–1.91)	1.18 (1.18–1.18)	2.6 (2.44–2.77)	1.06 (1.06–1.07)	2.23 (2.05–2.43)	1.04 (1.04–1.05)	1.46 (1.32–1.61)
29.86	1.13 (1.11–1.14)	1.53 (1.25–1.87)	1.2 (1.19–1.22)	1.69 (1.47–1.95)	1.2 (1.2–1.2)	2.64 (2.48–2.82)	1.07 (1.06–1.08)	2.24 (2.06–2.44)	1.05 (1.05–1.06)	1.47 (1.33–1.62)
30.04	1.14 (1.12–1.15)	1.54 (1.26–1.89)	1.22 (1.21–1.24)	1.72 (1.5–1.98)	1.22 (1.22–1.22)	2.69 (2.52–2.87)	1.08 (1.07–1.08)	2.26 (2.08–2.46)	1.06 (1.05–1.06)	1.48 (1.34–1.63)
30.21	1.15 (1.13–1.17)	1.56 (1.27–1.91)	1.24 (1.23–1.26)	1.75 (1.52–2.01)	1.24 (1.24–1.24)	2.73 (2.56–2.91)	1.08 (1.08–1.09)	2.27 (2.09–2.47)	1.07 (1.06–1.07)	1.49 (1.35–1.65)
30.38	1.16 (1.14–1.18)	1.58 (1.29–1.93)	1.26 (1.25–1.28)	1.78 (1.55–2.04)	1.26 (1.26–1.27)	2.78 (2.6–2.96)	1.09 (1.08–1.1)	2.29 (2.1–2.49)	1.08 (1.07–1.08)	1.5 (1.36–1.66)
30.55	1.18 (1.16–1.19)	1.59 (1.3–1.95)	1.29 (1.27–1.3)	1.81 (1.57–2.08)	1.28 (1.28–1.29)	2.82 (2.64–3.01)	1.1 (1.09–1.11)	2.3 (2.12–2.51)	1.08 (1.08–1.09)	1.52 (1.37–1.67)
30.72	1.19 (1.17–1.21)	1.61 (1.32–1.97)	1.31 (1.29–1.32)	1.84 (1.6–2.11)	1.3 (1.3–1.31)	2.87 (2.69–3.06)	1.11 (1.1–1.12)	2.32 (2.13–2.52)	1.09 (1.09–1.1)	1.53 (1.39–1.69)
30.89	1.2 (1.18–1.22)	1.63 (1.33–1.99)	1.33 (1.31–1.35)	1.87 (1.63–2.15)	1.32 (1.32–1.33)	2.91 (2.73–3.11)	1.11 (1.1–1.12)	2.33 (2.15–2.54)	1.1 (1.09–1.11)	1.54 (1.4–1.7)
31.06	1.21 (1.19–1.24)	1.64 (1.34–2.01)	1.35 (1.33–1.37)	1.9 (1.65–2.18)	1.34 (1.34–1.35)	2.96 (2.77–3.16)	1.12 (1.11–1.13)	2.35 (2.16–2.56)	1.11 (1.1–1.12)	1.56 (1.41–1.72)
31.23	1.22 (1.2–1.25)	1.66 (1.36–2.03)	1.37 (1.35–1.39)	1.93 (1.68–2.22)	1.37 (1.36–1.37)	3.01 (2.82–3.21)	1.13 (1.12–1.14)	2.37 (2.17–2.58)	1.13 (1.11–1.14)	1.57 (1.43–1.74)
31.40	1.24 (1.21–1.26)	1.68 (1.37–2.05)	1.4 (1.37–1.42)	1.96 (1.71–2.26)	1.39 (1.38–1.4)	3.05 (2.86–3.26)	1.14 (1.13–1.15)	2.38 (2.19–2.59)	1.14 (1.13–1.15)	1.59 (1.44–1.76)
31.57	1.25 (1.22–1.28)	1.69 (1.38–2.07)	1.42 (1.39–1.44)	2 (1.74–2.29)	1.41 (1.4–1.42)	3.1 (2.91–3.31)	1.14 (1.13–1.16)	2.4 (2.2–2.61)	1.15 (1.14–1.16)	1.61 (1.46–1.77)
31.74	1.26 (1.23–1.29)	1.71 (1.4–2.09)	1.44 (1.42–1.47)	2.03 (1.77–2.33)	1.43 (1.42–1.44)	3.15 (2.95–3.36)	1.15 (1.14–1.17)	2.42 (2.22–2.63)	1.16 (1.15–1.17)	1.62 (1.47–1.79)
31.91	1.28 (1.24–1.31)	1.73 (1.41–2.11)	1.47 (1.44–1.49)	2.06 (1.79–2.37)	1.45 (1.44–1.47)	3.2 (3–3.42)	1.16 (1.15–1.17)	2.43 (2.23–2.65)	1.17 (1.16–1.19)	1.64 (1.49–1.81)
32.09	1.29 (1.25–1.32)	1.75 (1.43–2.13)	1.49 (1.46–1.52)	2.09 (1.82–2.41)	1.48 (1.47–1.49)	3.25 (3.05–3.47)	1.17 (1.15–1.18)	2.45 (2.25–2.66)	1.19 (1.17–1.2)	1.66 (1.5–1.83)
32.26	1.3 (1.26–1.34)	1.76 (1.44–2.16)	1.51 (1.48–1.54)	2.13 (1.85–2.45)	1.5 (1.49–1.51)	3.31 (3.1–3.53)	1.18 (1.16–1.19)	2.47 (2.27–2.68)	1.2 (1.18–1.22)	1.68 (1.52–1.85)
32.43	1.31 (1.28–1.35)	1.78 (1.46–2.18)	1.54 (1.51–1.57)	2.16 (1.88–2.49)	1.52 (1.51–1.54)	3.36 (3.15–3.58)	1.18 (1.17–1.2)	2.48 (2.28–2.7)	1.21 (1.2–1.23)	1.7 (1.54–1.88)
32.60	1.33 (1.29–1.37)	1.8 (1.47–2.2)	1.56 (1.53–1.6)	2.2 (1.91–2.53)	1.55 (1.53–1.56)	3.41 (3.2–3.64)	1.19 (1.17–1.21)	2.5 (2.3–2.72)	1.23 (1.21–1.25)	1.72 (1.56–1.9)

32.77	1.34 (1.3–1.39)	1.82 (1.48–2.22)	1.59 (1.55–1.62)	2.23 (1.94–2.57)	1.57 (1.56–1.59)	3.46 (3.25–3.7)	1.2 (1.18–1.22)	2.52 (2.31–2.74)	1.24 (1.23–1.26)	1.74 (1.58–1.92)
32.94	1.35 (1.31–1.4)	1.83 (1.5–2.25)	1.61 (1.58–1.65)	2.27 (1.98–2.61)	1.6 (1.58–1.61)	3.52 (3.3–3.76)	1.21 (1.19–1.23)	2.54 (2.33–2.76)	1.26 (1.24–1.28)	1.76 (1.6–1.95)
33.11	1.37 (1.32–1.42)	1.85 (1.51–2.27)	1.64 (1.6–1.68)	2.31 (2.01–2.65)	1.62 (1.61–1.64)	3.57 (3.35–3.81)	1.22 (1.2–1.24)	2.55 (2.35–2.78)	1.28 (1.25–1.3)	1.78 (1.62–1.97)
33.28	1.38 (1.33–1.43)	1.87 (1.53–2.29)	1.67 (1.62–1.71)	2.34 (2.04–2.69)	1.65 (1.63–1.67)	3.63 (3.4–3.87)	1.23 (1.2–1.25)	2.57 (2.36–2.8)	1.29 (1.27–1.31)	1.81 (1.64–1.99)
33.45	1.4 (1.34–1.45)	1.89 (1.54–2.31)	1.69 (1.65–1.74)	2.38 (2.07–2.74)	1.67 (1.65–1.69)	3.69 (3.45–3.94)	1.24 (1.21–1.26)	2.59 (2.38–2.82)	1.31 (1.28–1.33)	1.83 (1.66–2.02)
33.62	1.41 (1.35–1.47)	1.91 (1.56–2.34)	1.72 (1.67–1.77)	2.42 (2.11–2.78)	1.7 (1.68–1.72)	3.74 (3.51–4)	1.24 (1.22–1.27)	2.61 (2.39–2.84)	1.32 (1.3–1.35)	1.85 (1.68–2.05)
33.79	1.42 (1.36–1.48)	1.93 (1.57–2.36)	1.75 (1.7–1.8)	2.46 (2.14–2.83)	1.73 (1.7–1.75)	3.8 (3.56–4.06)	1.25 (1.23–1.28)	2.63 (2.41–2.86)	1.34 (1.32–1.37)	1.88 (1.7–2.07)
33.96	1.44 (1.38–1.5)	1.95 (1.59–2.39)	1.78 (1.72–1.83)	2.5 (2.17–2.87)	1.75 (1.73–1.78)	3.86 (3.62–4.12)	1.26 (1.23–1.29)	2.64 (2.43–2.88)	1.36 (1.33–1.39)	1.9 (1.72–2.1)
34.14	1.45 (1.39–1.52)	1.97 (1.6–2.41)	1.8 (1.75–1.86)	2.54 (2.21–2.92)	1.78 (1.76–1.81)	3.92 (3.67–4.19)	1.27 (1.24–1.3)	2.66 (2.44–2.9)	1.38 (1.35–1.41)	1.93 (1.74–2.13)
34.31	1.47 (1.4–1.54)	1.99 (1.62–2.43)	1.83 (1.78–1.89)	2.58 (2.24–2.97)	1.81 (1.78–1.83)	3.98 (3.73–4.25)	1.28 (1.25–1.31)	2.68 (2.46–2.92)	1.4 (1.36–1.43)	1.95 (1.77–2.16)
34.48	1.48 (1.41–1.55)	2.01 (1.64–2.46)	1.86 (1.8–1.93)	2.62 (2.28–3.02)	1.84 (1.81–1.86)	4.04 (3.79–4.32)	1.29 (1.26–1.32)	2.7 (2.48–2.95)	1.41 (1.38–1.45)	1.98 (1.79–2.19)
34.65	1.49 (1.42–1.57)	2.03 (1.65–2.48)	1.89 (1.83–1.96)	2.66 (2.31–3.07)	1.87 (1.84–1.89)	4.11 (3.84–4.39)	1.3 (1.27–1.33)	2.72 (2.5–2.97)	1.43 (1.4–1.47)	2 (1.81–2.22)
34.82	1.51 (1.43–1.59)	2.05 (1.67–2.51)	1.92 (1.86–1.99)	2.71 (2.35–3.12)	1.89 (1.86–1.92)	4.17 (3.9–4.46)	1.31 (1.27–1.34)	2.74 (2.51–2.99)	1.45 (1.42–1.49)	2.03 (1.84–2.25)
34.99	1.52 (1.44–1.61)	2.07 (1.68–2.54)	1.95 (1.88–2.03)	2.75 (2.39–3.17)	1.92 (1.89–1.96)	4.24 (3.96–4.53)	1.32 (1.28–1.35)	2.76 (2.53–3.01)	1.47 (1.43–1.51)	2.06 (1.86–2.28)
35.16	1.54 (1.45–1.63)	2.09 (1.7–2.56)	1.99 (1.91–2.06)	2.79 (2.43–3.22)	1.95 (1.92–1.99)	4.3 (4.02–4.6)	1.33 (1.29–1.36)	2.78 (2.55–3.03)	1.49 (1.45–1.53)	2.09 (1.89–2.31)
35.33	1.55 (1.47–1.65)	2.11 (1.71–2.59)	2.02 (1.94–2.1)	2.84 (2.46–3.27)	1.98 (1.95–2.02)	4.37 (4.09–4.67)	1.34 (1.3–1.37)	2.8 (2.57–3.05)	1.51 (1.47–1.55)	2.11 (1.91–2.34)
35.50	1.57 (1.48–1.67)	2.13 (1.73–2.61)	2.05 (1.97–2.13)	2.88 (2.5–3.32)	2.01 (1.98–2.05)	4.44 (4.15–4.74)	1.35 (1.31–1.39)	2.82 (2.58–3.08)	1.53 (1.49–1.58)	2.14 (1.94–2.37)
35.67	1.59 (1.49–1.69)	2.15 (1.75–2.64)	2.08 (2–2.17)	2.93 (2.54–3.38)	2.05 (2.01–2.08)	4.5 (4.21–4.82)	1.35 (1.31–1.4)	2.84 (2.6–3.1)	1.55 (1.51–1.6)	2.17 (1.96–2.4)
35.84	1.6 (1.5–1.71)	2.17 (1.76–2.67)	2.12 (2.03–2.21)	2.98 (2.58–3.43)	2.08 (2.04–2.12)	4.57 (4.28–4.89)	1.36 (1.32–1.41)	2.86 (2.62–3.12)	1.57 (1.53–1.62)	2.2 (1.99–2.44)
36.02	1.62 (1.51–1.73)	2.19 (1.78–2.7)	2.15 (2.06–2.25)	3.02 (2.62–3.49)	2.11 (2.07–2.15)	4.65 (4.34–4.97)	1.37 (1.33–1.42)	2.88 (2.64–3.15)	1.59 (1.55–1.65)	2.23 (2.01–2.47)
36.19	1.63 (1.53–1.75)	2.21 (1.8–2.72)	2.18 (2.09–2.28)	3.07 (2.66–3.55)	2.14 (2.1–2.19)	4.72 (4.41–5.05)	1.38 (1.34–1.43)	2.9 (2.65–3.17)	1.62 (1.56–1.67)	2.26 (2.04–2.5)
36.36	1.65 (1.54–1.77)	2.23 (1.81–2.75)	2.22 (2.12–2.32)	3.12 (2.71–3.6)	2.18 (2.13–2.22)	4.79 (4.48–5.12)	1.39 (1.35–1.44)	2.92 (2.67–3.19)	1.64 (1.58–1.69)	2.29 (2.07–2.54)
36.53	1.66 (1.55–1.79)	2.26 (1.83–2.78)	2.26 (2.15–2.36)	3.17 (2.75–3.66)	2.21 (2.16–2.26)	4.87 (4.55–5.21)	1.4 (1.36–1.45)	2.94 (2.69–3.22)	1.66 (1.6–1.72)	2.32 (2.1–2.57)
36.70	1.68 (1.56–1.81)	2.28 (1.85–2.81)	2.29 (2.18–2.41)	3.22 (2.79–3.72)	2.24 (2.19–2.29)	4.94 (4.62–5.29)	1.41 (1.36–1.47)	2.96 (2.71–3.24)	1.68 (1.62–1.74)	2.35 (2.12–2.61)
36.87	1.7 (1.57–1.83)	2.3 (1.86–2.84)	2.33 (2.21–2.45)	3.28 (2.84–3.78)	2.28 (2.23–2.33)	5.02 (4.69–5.37)	1.42 (1.37–1.48)	2.98 (2.73–3.26)	1.7 (1.64–1.77)	2.38 (2.15–2.64)
37.04	1.71 (1.59–1.85)	2.32 (1.88–2.87)	2.37 (2.25–2.49)	3.33 (2.88–3.85)	2.31 (2.26–2.37)	5.1 (4.76–5.45)	1.43 (1.38–1.49)	3.01 (2.75–3.29)	1.73 (1.67–1.79)	2.42 (2.18–2.68)
37.21	1.73 (1.6–1.87)	2.35 (1.9–2.9)	2.4 (2.28–2.53)	3.38 (2.92–3.91)	2.35 (2.29–2.41)	5.17 (4.83–5.54)	1.44 (1.39–1.5)	3.03 (2.76–3.31)	1.75 (1.69–1.82)	2.45 (2.21–2.72)

37.38	1.75 (1.61–1.9)	2.37 (1.92–2.93)	2.44 (2.31–2.58)	3.44 (2.97–3.97)	2.39 (2.33–2.45)	5.25 (4.91–5.63)	1.45 (1.4–1.51)	3.05 (2.78–3.34)	1.77 (1.71–1.84)	2.48 (2.24–2.75)
37.55	1.77 (1.63–1.92)	2.39 (1.93–2.96)	2.48 (2.35–2.62)	3.49 (3.02–4.04)	2.42 (2.36–2.48)	5.34 (4.98–5.72)	1.46 (1.41–1.53)	3.07 (2.8–3.36)	1.8 (1.73–1.87)	2.51 (2.27–2.79)
37.72	1.78 (1.64–1.94)	2.42 (1.95–2.99)	2.52 (2.38–2.67)	3.55 (3.06–4.11)	2.46 (2.4–2.52)	5.42 (5.06–5.8)	1.48 (1.41–1.54)	3.09 (2.82–3.39)	1.82 (1.75–1.9)	2.55 (2.3–2.83)
37.89	1.8 (1.65–1.96)	2.44 (1.97–3.02)	2.56 (2.42–2.71)	3.6 (3.11–4.17)	2.5 (2.43–2.57)	5.5 (5.14–5.9)	1.49 (1.42–1.55)	3.11 (2.84–3.41)	1.85 (1.77–1.92)	2.58 (2.33–2.87)
38.07	1.82 (1.66–1.99)	2.46 (1.99–3.05)	2.6 (2.45–2.76)	3.66 (3.16–4.24)	2.54 (2.47–2.61)	5.59 (5.21–5.99)	1.5 (1.43–1.56)	3.14 (2.86–3.44)	1.87 (1.79–1.95)	2.62 (2.36–2.91)
38.24	1.84 (1.68–2.01)	2.49 (2.01–3.09)	2.64 (2.49–2.81)	3.72 (3.21–4.31)	2.58 (2.51–2.65)	5.68 (5.29–6.08)	1.51 (1.44–1.58)	3.16 (2.88–3.47)	1.9 (1.82–1.98)	2.65 (2.39–2.95)
38.41	1.85 (1.69–2.03)	2.51 (2.02–3.12)	2.69 (2.53–2.86)	3.78 (3.26–4.38)	2.62 (2.55–2.69)	5.76 (5.38–6.18)	1.52 (1.45–1.59)	3.18 (2.9–3.49)	1.92 (1.84–2.01)	2.69 (2.42–2.99)
38.58	1.87 (1.7–2.06)	2.54 (2.04–3.15)	2.73 (2.56–2.91)	3.84 (3.31–4.46)	2.66 (2.58–2.73)	5.85 (5.46–6.28)	1.53 (1.46–1.6)	3.2 (2.92–3.52)	1.95 (1.86–2.04)	2.72 (2.45–3.03)
38.75	1.89 (1.72–2.08)	2.56 (2.06–3.18)	2.77 (2.6–2.96)	3.9 (3.36–4.53)	2.7 (2.62–2.78)	5.94 (5.54–6.37)	1.54 (1.47–1.61)	3.23 (2.94–3.54)	1.97 (1.89–2.07)	2.76 (2.48–3.07)
38.92	1.91 (1.73–2.11)	2.59 (2.08–3.22)	2.82 (2.64–3.01)	3.96 (3.41–4.6)	2.74 (2.66–2.82)	6.04 (5.63–6.48)	1.55 (1.48–1.63)	3.25 (2.96–3.57)	2 (1.91–2.1)	2.8 (2.52–3.11)
39.09	1.93 (1.74–2.13)	2.61 (2.1–3.25)	2.86 (2.68–3.06)	4.03 (3.47–4.68)	2.78 (2.7–2.87)	6.13 (5.71–6.58)	1.56 (1.49–1.64)	3.27 (2.98–3.6)	2.03 (1.93–2.13)	2.84 (2.55–3.16)
39.26	1.95 (1.76–2.16)	2.64 (2.12–3.29)	2.91 (2.72–3.11)	4.09 (3.52–4.76)	2.83 (2.74–2.92)	6.22 (5.8–6.68)	1.57 (1.5–1.65)	3.3 (3–3.63)	2.05 (1.96–2.16)	2.87 (2.58–3.2)
39.43	1.97 (1.77–2.18)	2.66 (2.14–3.32)	2.96 (2.76–3.17)	4.16 (3.58–4.84)	2.87 (2.78–2.96)	6.32 (5.89–6.79)	1.58 (1.5–1.67)	3.32 (3.02–3.65)	2.08 (1.98–2.19)	2.91 (2.62–3.24)
39.60	1.99 (1.79–2.21)	2.69 (2.16–3.36)	3 (2.8–3.22)	4.23 (3.63–4.92)	2.92 (2.82–3.01)	6.42 (5.98–6.89)	1.6 (1.51–1.68)	3.34 (3.04–3.68)	2.11 (2.01–2.22)	2.95 (2.65–3.29)
39.77	2.01 (1.8–2.23)	2.72 (2.18–3.39)	3.05 (2.84–3.28)	4.29 (3.69–5)	2.96 (2.87–3.06)	6.52 (6.07–7)	1.61 (1.52–1.69)	3.37 (3.06–3.71)	2.14 (2.03–2.25)	2.99 (2.68–3.33)
39.94	2.03 (1.82–2.26)	2.74 (2.2–3.43)	3.1 (2.88–3.34)	4.36 (3.74–5.08)	3.01 (2.91–3.11)	6.62 (6.16–7.11)	1.62 (1.53–1.71)	3.39 (3.08–3.74)	2.17 (2.06–2.28)	3.03 (2.72–3.38)
40.12	2.05 (1.83–2.29)	2.77 (2.22–3.47)	3.15 (2.92–3.39)	4.43 (3.8–5.17)	3.05 (2.95–3.16)	6.72 (6.26–7.22)	1.63 (1.54–1.72)	3.41 (3.1–3.77)	2.2 (2.08–2.32)	3.07 (2.75–3.43)
40.29	2.07 (1.84–2.31)	2.8 (2.24–3.5)	3.2 (2.97–3.45)	4.5 (3.86–5.25)	3.1 (3–3.21)	6.83 (6.35–7.34)	1.64 (1.55–1.74)	3.44 (3.12–3.79)	2.23 (2.11–2.35)	3.11 (2.79–3.47)
40.46	2.09 (1.86–2.34)	2.83 (2.26–3.54)	3.25 (3.01–3.51)	4.58 (3.92–5.34)	3.15 (3.04–3.26)	6.93 (6.45–7.45)	1.65 (1.56–1.75)	3.46 (3.14–3.82)	2.26 (2.14–2.38)	3.15 (2.83–3.52)
40.63	2.11 (1.87–2.37)	2.85 (2.28–3.58)	3.3 (3.06–3.57)	4.65 (3.98–5.43)	3.2 (3.09–3.31)	7.04 (6.55–7.57)	1.66 (1.57–1.76)	3.49 (3.16–3.85)	2.29 (2.16–2.42)	3.2 (2.86–3.57)
40.80	2.13 (1.89–2.4)	2.88 (2.3–3.62)	3.36 (3.1–3.64)	4.72 (4.04–5.52)	3.25 (3.13–3.37)	7.15 (6.65–7.69)	1.68 (1.58–1.78)	3.51 (3.18–3.88)	2.32 (2.19–2.45)	3.24 (2.9–3.62)
40.97	2.15 (1.9–2.42)	2.91 (2.32–3.66)	3.41 (3.15–3.7)	4.8 (4.1–5.61)	3.3 (3.18–3.42)	7.26 (6.75–7.81)	1.69 (1.59–1.79)	3.54 (3.2–3.91)	2.35 (2.22–2.49)	3.28 (2.94–3.67)
41.14	2.17 (1.92–2.45)	2.94 (2.34–3.7)	3.47 (3.19–3.76)	4.88 (4.17–5.7)	3.35 (3.23–3.48)	7.37 (6.85–7.94)	1.7 (1.6–1.81)	3.56 (3.22–3.94)	2.38 (2.24–2.52)	3.33 (2.98–3.72)
41.31	2.19 (1.93–2.48)	2.97 (2.36–3.74)	3.52 (3.24–3.83)	4.95 (4.23–5.8)	3.4 (3.27–3.53)	7.49 (6.96–8.06)	1.71 (1.61–1.82)	3.59 (3.24–3.97)	2.41 (2.27–2.56)	3.37 (3.02–3.77)
41.48	2.21 (1.95–2.51)	3 (2.38–3.78)	3.58 (3.29–3.89)	5.03 (4.3–5.9)	3.45 (3.32–3.59)	7.6 (7.06–8.19)	1.72 (1.62–1.84)	3.61 (3.27–4)	2.44 (2.3–2.6)	3.42 (3.05–3.82)
41.65	2.23 (1.96–2.54)	3.03 (2.4–3.82)	3.64 (3.34–3.96)	5.11 (4.36–6)	3.51 (3.37–3.65)	7.72 (7.17–8.32)	1.74 (1.63–1.85)	3.64 (3.29–4.03)	2.48 (2.33–2.63)	3.46 (3.09–3.88)
41.82	2.26 (1.98–2.57)	3.06 (2.42–3.86)	3.69 (3.38–4.03)	5.2 (4.43–6.1)	3.56 (3.42–3.71)	7.84 (7.28–8.45)	1.75 (1.64–1.87)	3.67 (3.31–4.06)	2.51 (2.36–2.67)	3.51 (3.13–3.93)

41.99	2.28 (1.99–2.6)	3.09 (2.44–3.9)	3.75 (3.43–4.1)	5.28 (4.5–6.2)	3.62 (3.48–3.76)	7.96 (7.39–8.58)	1.76 (1.65–1.88)	3.69 (3.33–4.09)	2.54 (2.39–2.71)	3.56 (3.17–3.99)
42.17	2.3 (2.01–2.63)	3.12 (2.46–3.94)	3.81 (3.49–4.17)	5.37 (4.57–6.3)	3.67 (3.53–3.83)	8.09 (7.5–8.72)	1.77 (1.66–1.9)	3.72 (3.35–4.13)	2.58 (2.42–2.75)	3.6 (3.22–4.04)
42.34	2.32 (2.03–2.66)	3.15 (2.49–3.99)	3.87 (3.54–4.24)	5.45 (4.64–6.41)	3.73 (3.58–3.89)	8.21 (7.62–8.86)	1.79 (1.67–1.91)	3.75 (3.37–4.16)	2.61 (2.45–2.79)	3.65 (3.26–4.1)
42.51	2.35 (2.04–2.7)	3.18 (2.51–4.03)	3.94 (3.59–4.32)	5.54 (4.71–6.51)	3.79 (3.63–3.95)	8.34 (7.73–9)	1.8 (1.68–1.93)	3.77 (3.4–4.19)	2.65 (2.48–2.83)	3.7 (3.3–4.15)
42.68	2.37 (2.06–2.73)	3.21 (2.53–4.07)	4 (3.64–4.39)	5.63 (4.78–6.62)	3.85 (3.69–4.01)	8.47 (7.85–9.14)	1.81 (1.69–1.94)	3.8 (3.42–4.22)	2.68 (2.51–2.87)	3.75 (3.34–4.21)
42.85	2.39 (2.07–2.76)	3.24 (2.55–4.12)	4.06 (3.69–4.47)	5.72 (4.85–6.73)	3.91 (3.74–4.08)	8.6 (7.97–9.29)	1.83 (1.7–1.96)	3.83 (3.44–4.25)	2.72 (2.54–2.91)	3.8 (3.39–4.27)
43.02	2.42 (2.09–2.79)	3.27 (2.57–4.16)	4.13 (3.75–4.55)	5.81 (4.93–6.85)	3.97 (3.8–4.14)	8.74 (8.09–9.43)	1.84 (1.71–1.97)	3.85 (3.46–4.29)	2.76 (2.57–2.95)	3.85 (3.43–4.33)
43.19	2.44 (2.11–2.83)	3.31 (2.6–4.21)	4.2 (3.8–4.63)	5.9 (5–6.96)	4.03 (3.86–4.21)	8.87 (8.21–9.58)	1.85 (1.72–1.99)	3.88 (3.49–4.32)	2.79 (2.61–2.99)	3.91 (3.47–4.39)
43.36	2.46 (2.12–2.86)	3.34 (2.62–4.26)	4.26 (3.86–4.71)	6 (5.08–7.08)	4.09 (3.91–4.28)	9.01 (8.34–9.73)	1.86 (1.73–2.01)	3.91 (3.51–4.35)	2.83 (2.64–3.04)	3.96 (3.52–4.45)
43.53	2.49 (2.14–2.89)	3.37 (2.64–4.3)	4.33 (3.92–4.79)	6.09 (5.16–7.2)	4.15 (3.97–4.35)	9.15 (8.47–9.89)	1.88 (1.74–2.02)	3.94 (3.53–4.39)	2.87 (2.67–3.08)	4.01 (3.56–4.51)
43.70	2.51 (2.16–2.93)	3.41 (2.67–4.35)	4.4 (3.97–4.87)	6.19 (5.24–7.32)	4.22 (4.03–4.42)	9.29 (8.59–10.04)	1.89 (1.75–2.04)	3.96 (3.55–4.42)	2.91 (2.7–3.12)	4.06 (3.61–4.58)
43.87	2.54 (2.17–2.96)	3.44 (2.69–4.4)	4.47 (4.03–4.96)	6.29 (5.32–7.44)	4.28 (4.09–4.49)	9.44 (8.72–10.2)	1.9 (1.77–2.06)	3.99 (3.58–4.45)	2.95 (2.74–3.17)	4.12 (3.66–4.64)
44.05	2.56 (2.19–3)	3.47 (2.71–4.45)	4.54 (4.09–5.04)	6.39 (5.4–7.57)	4.35 (4.15–4.56)	9.58 (8.86–10.37)	1.92 (1.78–2.07)	4.02 (3.6–4.49)	2.98 (2.77–3.21)	4.17 (3.7–4.7)
44.22	2.59 (2.21–3.03)	3.51 (2.74–4.49)	4.62 (4.15–5.13)	6.49 (5.48–7.69)	4.42 (4.21–4.63)	9.73 (8.99–10.53)	1.93 (1.79–2.09)	4.05 (3.62–4.52)	3.02 (2.81–3.26)	4.23 (3.75–4.77)
44.39	2.61 (2.23–3.07)	3.54 (2.76–4.54)	4.69 (4.21–5.22)	6.6 (5.57–7.82)	4.49 (4.28–4.71)	9.88 (9.13–10.7)	1.95 (1.8–2.11)	4.08 (3.65–4.56)	3.07 (2.84–3.31)	4.29 (3.8–4.84)
44.56	2.64 (2.24–3.1)	3.58 (2.78–4.59)	4.76 (4.28–5.31)	6.7 (5.65–7.95)	4.56 (4.34–4.78)	10.03 (9.27–10.87)	1.96 (1.81–2.12)	4.11 (3.67–4.59)	3.11 (2.88–3.35)	4.34 (3.85–4.9)
44.73	2.67 (2.26–3.14)	3.61 (2.81–4.65)	4.84 (4.34–5.4)	6.81 (5.74–8.09)	4.63 (4.41–4.86)	10.19 (9.41–11.04)	1.97 (1.82–2.14)	4.14 (3.7–4.63)	3.15 (2.91–3.4)	4.4 (3.9–4.97)
44.90	2.69 (2.28–3.18)	3.65 (2.83–4.7)	4.92 (4.4–5.5)	6.92 (5.82–8.22)	4.7 (4.47–4.94)	10.35 (9.55–11.21)	1.99 (1.83–2.16)	4.17 (3.72–4.67)	3.19 (2.95–3.45)	4.46 (3.95–5.04)
45.07	2.72 (2.3–3.22)	3.68 (2.86–4.75)	5 (4.47–5.59)	7.03 (5.91–8.36)	4.77 (4.54–5.02)	10.51 (9.69–11.39)	2 (1.84–2.17)	4.2 (3.74–4.7)	3.23 (2.99–3.5)	4.52 (4–5.11)
45.24	2.74 (2.32–3.25)	3.72 (2.88–4.8)	5.08 (4.53–5.69)	7.14 (6–8.5)	4.85 (4.61–5.1)	10.67 (9.84–11.57)	2.02 (1.85–2.19)	4.23 (3.77–4.74)	3.28 (3.02–3.55)	4.58 (4.05–5.18)
45.41	2.77 (2.33–3.29)	3.76 (2.91–4.85)	5.16 (4.6–5.79)	7.26 (6.09–8.65)	4.92 (4.67–5.18)	10.84 (9.99–11.76)	2.03 (1.87–2.21)	4.26 (3.79–4.78)	3.32 (3.06–3.6)	4.64 (4.1–5.26)
45.58	2.8 (2.35–3.33)	3.79 (2.93–4.91)	5.24 (4.67–5.89)	7.38 (6.19–8.79)	5 (4.74–5.27)	11.01 (10.14–11.94)	2.05 (1.88–2.23)	4.29 (3.82–4.81)	3.37 (3.1–3.65)	4.71 (4.16–5.33)
45.75	2.83 (2.37–3.37)	3.83 (2.96–4.96)	5.33 (4.74–5.99)	7.49 (6.28–8.94)	5.08 (4.81–5.35)	11.18 (10.3–12.13)	2.06 (1.89–2.25)	4.32 (3.84–4.85)	3.41 (3.14–3.71)	4.77 (4.21–5.4)
45.92	2.85 (2.39–3.41)	3.87 (2.98–5.02)	5.41 (4.81–6.1)	7.61 (6.38–9.09)	5.15 (4.89–5.44)	11.35 (10.45–12.33)	2.07 (1.9–2.26)	4.35 (3.87–4.89)	3.46 (3.18–3.76)	4.83 (4.26–5.48)
46.10	2.88 (2.41–3.45)	3.91 (3.01–5.07)	5.5 (4.88–6.2)	7.74 (6.47–9.25)	5.23 (4.96–5.52)	11.53 (10.61–12.52)	2.09 (1.91–2.28)	4.38 (3.89–4.93)	3.5 (3.22–3.81)	4.9 (4.32–5.56)
46.27	2.91 (2.43–3.49)	3.94 (3.03–5.13)	5.59 (4.95–6.31)	7.86 (6.57–9.4)	5.32 (5.03–5.61)	11.71 (10.77–12.72)	2.1 (1.92–2.3)	4.41 (3.92–4.96)	3.55 (3.26–3.87)	4.96 (4.37–5.64)
46.44	2.94 (2.45–3.53)	3.98 (3.06–5.19)	5.68 (5.02–6.42)	7.99 (6.67–9.56)	5.4 (5.11–5.7)	11.89 (10.93–12.92)	2.12 (1.94–2.32)	4.44 (3.94–5)	3.6 (3.3–3.92)	5.03 (4.43–5.71)

46.61	2.97 (2.47–3.57)	4.02 (3.09–5.24)	5.77 (5.09–6.53)	8.12 (6.77–9.72)	5.48 (5.19–5.8)	12.07 (11.1–13.13)	2.13 (1.95–2.34)	4.47 (3.97–5.04)	3.65 (3.34–3.98)	5.1 (4.49–5.79)
46.78	3 (2.49–3.62)	4.06 (3.11–5.3)	5.86 (5.17–6.64)	8.25 (6.88–9.89)	5.57 (5.26–5.89)	12.26 (11.27–13.34)	2.15 (1.96–2.36)	4.5 (3.99–5.08)	3.69 (3.38–4.04)	5.17 (4.54–5.88)
46.95	3.03 (2.51–3.66)	4.1 (3.14–5.36)	5.95 (5.25–6.76)	8.38 (6.98–10.06)	5.65 (5.34–5.98)	12.45 (11.44–13.55)	2.16 (1.97–2.38)	4.54 (4.02–5.12)	3.74 (3.42–4.09)	5.24 (4.6–5.96)
47.12	3.06 (2.52–3.7)	4.14 (3.17–5.42)	6.05 (5.32–6.88)	8.51 (7.09–10.23)	5.74 (5.42–6.08)	12.64 (11.61–13.76)	2.18 (1.98–2.39)	4.57 (4.04–5.16)	3.79 (3.47–4.15)	5.31 (4.66–6.04)
47.29	3.09 (2.54–3.75)	4.18 (3.19–5.48)	6.15 (5.4–7)	8.65 (7.19–10.4)	5.83 (5.5–6.18)	12.84 (11.79–13.98)	2.2 (2–2.41)	4.6 (4.07–5.2)	3.85 (3.51–4.21)	5.38 (4.72–6.13)
47.46	3.12 (2.56–3.79)	4.22 (3.22–5.54)	6.25 (5.48–7.12)	8.79 (7.3–10.57)	5.92 (5.58–6.28)	13.04 (11.97–14.21)	2.21 (2.01–2.43)	4.63 (4.1–5.24)	3.9 (3.55–4.27)	5.45 (4.78–6.21)
47.63	3.15 (2.59–3.84)	4.27 (3.25–5.61)	6.35 (5.56–7.24)	8.93 (7.41–10.75)	6.01 (5.67–6.38)	13.24 (12.15–14.43)	2.23 (2.02–2.45)	4.67 (4.12–5.28)	3.95 (3.6–4.33)	5.52 (4.84–6.3)
47.80	3.18 (2.61–3.88)	4.31 (3.27–5.67)	6.45 (5.64–7.37)	9.07 (7.52–10.94)	6.11 (5.75–6.48)	13.45 (12.33–14.66)	2.24 (2.03–2.47)	4.7 (4.15–5.33)	4 (3.64–4.4)	5.6 (4.91–6.39)
47.97	3.21 (2.63–3.93)	4.35 (3.3–5.73)	6.55 (5.73–7.5)	9.22 (7.64–11.12)	6.2 (5.84–6.59)	13.66 (12.52–14.9)	2.26 (2.05–2.49)	4.73 (4.18–5.37)	4.06 (3.69–4.46)	5.67 (4.97–6.48)
48.15	3.24 (2.65–3.97)	4.39 (3.33–5.8)	6.66 (5.81–7.63)	9.36 (7.75–11.31)	6.3 (5.93–6.69)	13.87 (12.71–15.13)	2.27 (2.06–2.51)	4.77 (4.2–5.41)	4.11 (3.73–4.52)	5.75 (5.03–6.57)
48.32	3.27 (2.67–4.02)	4.44 (3.36–5.86)	6.76 (5.89–7.76)	9.52 (7.87–11.5)	6.4 (6.01–6.8)	14.08 (12.9–15.37)	2.29 (2.07–2.53)	4.8 (4.23–5.45)	4.17 (3.78–4.59)	5.83 (5.1–6.66)
48.49	3.31 (2.69–4.07)	4.48 (3.39–5.93)	6.87 (5.98–7.89)	9.67 (7.99–11.7)	6.49 (6.1–6.91)	14.3 (13.09–15.62)	2.31 (2.08–2.55)	4.84 (4.26–5.49)	4.22 (3.83–4.65)	5.9 (5.16–6.75)
48.66	3.34 (2.71–4.12)	4.52 (3.42–5.99)	6.98 (6.07–8.03)	9.82 (8.11–11.9)	6.6 (6.19–7.02)	14.52 (13.29–15.87)	2.32 (2.1–2.57)	4.87 (4.28–5.54)	4.28 (3.88–4.72)	5.98 (5.23–6.85)
48.83	3.37 (2.73–4.16)	4.57 (3.44–6.06)	7.09 (6.16–8.17)	9.98 (8.23–12.1)	6.7 (6.29–7.14)	14.75 (13.49–16.12)	2.34 (2.11–2.6)	4.91 (4.31–5.58)	4.34 (3.93–4.79)	6.06 (5.29–6.95)
49.00	3.41 (2.75–4.21)	4.61 (3.47–6.13)	7.21 (6.25–8.31)	10.14 (8.36–12.3)	6.8 (6.38–7.25)	14.98 (13.7–16.38)	2.36 (2.12–2.62)	4.94 (4.34–5.62)	4.39 (3.97–4.86)	6.15 (5.36–7.04)

Table S11 Interaction of AMA and ART treatment with adverse pregnancy outcomes

Adverse outcomes		AMA	ART	*OR* (95%*CI*)
**GDM**	OR00	No	No	Reference
	OR10	Yes	No	2.37 [2.28, 2.47]
	OR01	No	Yes	2.11 [1.91, 2.32]
	OR11	Yes	Yes	3.67 [3.21, 4.21]
	RERI (95%CI)	0.19 [-0.32, 0.76]		
	AP (95CI%)	0.05 [-0.1, 0.18]		
	SI (95CI%)	1.08 [0.88, 1.32]		
**HDP**	OR00	No	No	Reference
	OR10	Yes	No	2.16 [2.04, 2.28]
	OR01	No	Yes	1.88 [1.65, 2.14]
	OR11	Yes	Yes	2.27 [1.87, 2.75]
	RERI (95%CI)	-0.77 [-1.25, -0.23]		
	AP (95CI%)	-0.34 [-0.67, -0.1]		
	SI (95CI%)	0.62 [0.43, 0.89]		
**Anemia**	OR00	No	No	Reference
	OR10	Yes	No	1.04 [1, 1.08]
	OR01	No	Yes	1.37 [1.25, 1.5]
	OR11	Yes	Yes	1.41 [1.22, 1.62]
	RERI (95%CI)	0 [-0.23, 0.25]		
	AP (95CI%)	0 [-0.2, 0.14]		
	SI (95CI%)	1 [0.56, 1.78]		
**Thrombocytopenia**	OR00	No	No	Reference
	OR10	Yes	No	1.17 [1.06, 1.28]
	OR01	No	Yes	1.55 [1.24, 1.95]
	OR11	Yes	Yes	1.62 [1.15, 2.28]
	RERI (95%CI)	-0.1 [-0.71, 0.63]		
	AP (95CI%)	-0.06 [-0.64, 0.23]		
	SI (95CI%)	0.86 [0.31, 2.38]		
**Hypothyroidism**	OR00	No	No	Reference

	OR10	Yes	No	1.34 [1.28, 1.4]
	OR01	No	Yes	1.66 [1.5, 1.83]
	OR11	Yes	Yes	1.88 [1.6, 2.2]
	RERI (95%CI)	-0.12 [-0.45, 0.24]		
	AP (95CI%)	-0.06 [-0.28, 0.1]		
	SI (95CI%)	0.88 [0.6, 1.29]		
**Hyperthyroidism**	OR00	No	No	Reference
	OR10	Yes	No	0.95 [0.72, 1.25]
	OR01	No	Yes	1.64 [0.98, 2.75]
	OR11	Yes	Yes	1.43 [0.59, 3.47]
	RERI (95%CI)	-0.16 [-1.57, 1.98]		
	AP (95CI%)	-0.11 [-2.63, 0.28]		
	SI (95CI%)	0.73 [0.03, 19.25]		
**Polyhydramnios**	OR00	No	No	Reference
	OR10	Yes	No	1.31 [1.18, 1.45]
	OR01	No	Yes	1.52 [1.17, 1.98]
	OR11	Yes	Yes	1.97 [1.39, 2.81]
	RERI (95%CI)	0.14 [-0.6, 1.04]		
	AP (95CI%)	0.07 [-0.46, 0.33]		
	SI (95CI%)	1.17 [0.49, 2.77]		
**Oligohydramnios**	OR00	No	No	Reference
	OR10	Yes	No	1.15 [1.08, 1.24]
	OR01	No	Yes	1.09 [0.94, 1.26]
	OR11	Yes	Yes	1.08 [0.84, 1.37]
	RERI (95%CI)	-0.17 [-0.47, 0.17]		
	AP (95CI%)	-0.16 [-0.58, 0.08]		
	SI (95CI%)	0.31 [0.01, 10.35]		
**PROM**	OR00	No	No	Reference
	OR10	Yes	No	1.17 [1.12, 1.22]
	OR01	No	Yes	0.84 [0.76, 0.93]
	OR11	Yes	Yes	0.67 [0.56, 0.8]

	RERI (95%CI)	-0.34 [-0.49, -0.18]		
	AP (95CI%)	-0.5 [-0.88, -0.27]		
	SI (95CI%)	/		
**Placenta previa**	OR00	No	No	Reference
	OR10	Yes	No	1.73 [1.61, 1.86]
	OR01	No	Yes	1.68 [1.38, 2.03]
	OR11	Yes	Yes	2.33 [1.82, 2.97]
	RERI (95%CI)	-0.08 [-0.7, 0.62]		
	AP (95CI%)	-0.03 [-0.39, 0.19]		
	SI (95CI%)	0.94 [0.58, 1.53]		
**Placental abruption**	OR00	No	No	Reference
	OR10	Yes	No	1.28 [1.15, 1.42]
	OR01	No	Yes	1 [0.75, 1.32]
	OR11	Yes	Yes	1.05 [0.68, 1.63]
	RERI (95%CI)	-0.22 [-0.73, 0.41]		
	AP (95CI%)	-0.21 [-1.15, 0.11]		
	SI (95CI%)	0.19 [0, 1151]		
**Cesarean section**	OR00	No	No	Reference
	OR10	Yes	No	1.79 [1.73, 1.85]
	OR01	No	Yes	2.73 [2.5, 2.98]
	OR11	Yes	Yes	4.81 [4.09, 5.66]
	RERI (95%CI)	1.29 [0.53, 2.17]		
	AP (95CI%)	0.27 [0.12, 0.38]		
	SI (95CI%)	1.51 [1.21, 1.89]		
**LGA**	OR00	No	No	Reference
	OR10	Yes	No	0.9 [0.84, 0.96]
	OR01	No	Yes	1.01 [0.86, 1.2]
	OR11	Yes	Yes	0.89 [0.68, 1.18]
	RERI (95%CI)	-0.02 [-0.31, 0.31]		
	AP (95CI%)	-0.02 [-0.5, 0.21]		
	SI (95CI%)	/		

**SGA**	OR00	No	No	Reference
	OR10	Yes	No	1.14 [1.04, 1.24]
	OR01	No	Yes	1.03 [0.83, 1.27]
	OR11	Yes	Yes	0.8 [0.55, 1.16]
	RERI (95%CI)	-0.37 [-0.73, 0.05]		
	AP (95CI%)	-0.46 [-1.37, -0.1]		
	SI (95CI%)	/		
**Postpartum hemorrhage**	OR00	No	No	Reference
	OR10	Yes	No	1.29 [1.23, 1.36]
	OR01	No	Yes	2.36 [2.12, 2.64]
	OR11	Yes	Yes	1.9 [1.59, 2.27]
	RERI (95%CI)	-0.76 [-1.18, -0.31]		
	AP (95CI%)	-0.4 [-0.73, -0.16]		
	SI (95CI%)	0.54 [0.36, 0.81]		
**Congenital malformation**	OR00	No	No	Reference
	OR10	Yes	No	1.28 [1.07, 1.53]
	OR01	No	Yes	1.03 [0.64, 1.65]
	OR11	Yes	Yes	0.83 [0.37, 1.86]
	RERI (95%CI)	-0.48 [-1.28, 0.63]		
	AP (95CI%)	-0.57 [-3.64, -0.14]		
	SI (95CI%)	/		

AMA, advanced maternal age; ART, assisted reproductive technology; GDM, gestational diabetes mellitus; HDP, hypertensive disorders in pregnancy; PROM, premature rupture of membranes; LGA, Large-for-gestational-age; SGA, small-for-gestational-age.

RERI, relative excess risk due to interaction; AP, the attributable proportion due to interaction; SI, the synergy index.

Table S12 The effect of prior cesarean section on next pregnancy complications and adverse perinatal outcomes

**Adverse outcomes**	**Group**	**Multipara without prior cesarean section**	**Multipara with prior cesarean section**	***PRR (95%CI)***	***P***
Hypothyroidism	No	47026 (88.8)	18020 (82.7)	1.69 (1.62–1.77)	0.000
	Yes	5961 (11.2)	3773 (17.3)		
Hyperthyroidism	No	52788 (99.6)	21685 (99.5)	1.55 (1.21–1.98)	0.001
	Yes	199 (0.4)	108 (0.5)		
GDM	No	45177 (85.3)	16904 (77.6)	1.48 (1.42–1.55)	0.000
	Yes	7810 (14.7)	4889 (22.4)		
HDP	No	48442 (91.4)	20358 (93.4)	0.78 (0.73–0.83)	0.000
	Yes	4545 (8.6)	1435 (6.6)		
Thrombocytopenia	No	51454 (97.1)	21022 (96.5)	1.31 (1.19–1.43)	0.000
	Yes	1533 (2.9)	771 (3.5)		
Anemia	No	39942 (75.4)	14257 (65.4)	1.76 (1.7–1.83)	0.000
	Yes	13045 (24.6)	7536 (34.6)		
Polyhydramnios	No	51511 (97.2)	21237 (97.4)	0.95 (0.86–1.05)	0.324
	Yes	1476 (2.8)	556 (2.6)		
Oligohydramnios	No	50737 (95.8)	20732 (95.1)	1.01 (0.94–1.09)	0.769
	Yes	2250 (4.2)	1061 (4.9)		
PROM	No	42965 (81.1)	19759 (90.7)	0.42 (0.4–0.44)	0.000
	Yes	10022 (18.9)	2034 (9.3)		
Placenta previa	No	50083 (94.5)	21149 (97)	0.47 (0.43–0.51)	0.000
	Yes	2904 (5.5)	644 (3)		
Placental abruption	No	51559 (97.3)	21558 (98.9)	0.38 (0.33–0.44)	0.000
	Yes	1428 (2.7)	235 (1.1)		

Perinatal asphyxia	No	50927 (96.1)	21210 (97.3)	0.66 (0.6–0.72)	0.000
	Yes	2060 (3.9)	583 (2.7)		
LGA	No	49827 (94)	20722 (95.1)	0.69 (0.64–0.74)	0.000
	Yes	3160 (6)	1071 (4.9)		
SGA	No	49136 (92.7)	20745 (95.2)	1.07 (0.99–1.16)	0.069
	Yes	3851 (7.3)	1048 (4.8)		
Postpartum hemorrhage	No	45813 (86.5)	19199 (88.1)	1.05 (1–1.1)	0.068
	Yes	7174 (13.5)	2594 (11.9)		
Stillbirth	No	52600 (99.3)	21741 (99.8)	0.37 (0.27–0.5)	0.000
	Yes	387 (0.7)	52 (0.2)		
Premature	No	41190 (77.74)	188639 (85.53)	0.86 (0.82-0.90)	0.000
	Yes	11797 (22.26)	3154 (14.47)		
Congenital malformation	No	52605 (99.3)	21654 (99.4)	0.87 (0.71–1.06)	0.160
	Yes	382 (0.7)	139 (0.6)		

GDM, gestational diabetes mellitus; HDP, hypertensive disorders in pregnancy; PROM, premature rupture of membranes; LGA, Large-for-gestational-age; SGA, small-for-gestational-age.

The gravidity, parity, assisted reproductive technology intervention, and twin or multiple gestations were adjusted.

Table S13 The effect of prior cesarean section on next pregnancy complications and adverse perinatal outcomes in different age stratifications

Adverse outcomes	<35 years		≥35years	
	PRR (95%CI)	*P*	PRR (95%CI)	*P*
Hyperthyroidism	1.39 (1.04–1.85)	0.024	1.91 (1.14–3.2)	0.014
Hypothyroidism	1.66 (1.57–1.75)	0.000	1.71 (1.57–1.86)	0.000
GDM	1.46 (1.38–1.54)	0.000	1.49 (1.39–1.59)	0.000
HDP	0.87 (0.8–0.95)	0.001	0.7 (0.63–0.77)	0.000
Thrombocytopenia	1.25 (1.12–1.39)	0.000	1.34 (1.13–1.59)	0.001
Anemia	1.93 (1.85–2.01)	0.000	1.45 (1.35–1.55)	0.000
Polyhydramnios	0.87 (0.77–0.99)	0.038	1.13 (0.95–1.34)	0.181
Oligohydramnios	1.11 (1.01–1.21)	0.029	0.82 (0.71–0.94)	0.006
PROM	0.41 (0.39–0.44)	0.000	0.42 (0.38–0.46)	0.000
Placenta previa	0.5 (0.45–0.56)	0.000	0.4 (0.35–0.47)	0.000
Placental abruption	0.38 (0.32–0.45)	0.000	0.41 (0.32–0.51)	0.000
Perinatal asphyxia	0.74 (0.66–0.83)	0.000	0.53 (0.45–0.63)	0.000
LGA	0.67 (0.61–0.73)	0.000	0.7 (0.61–0.8)	0.000
SGA	1.13 (1.03–1.24)	0.012	1.02 (0.89–1.17)	0.761
Postpartum hemorrhage	1.13 (1.07–1.2)	0.000	0.88 (0.8–0.97)	0.007
Stillbirth	0.91 (0.86–0.97)	0.002	0.79 (0.72–0.86)	0.000
Premature	0.36 (0.25–0.52)	0.000	0.38 (0.23–0.65)	0.000
Congenital malformation	0.93 (0.73–1.18)	0.551	0.77 (0.53–1.1)	0.154

GDM, gestational diabetes mellitus; HDP, hypertensive disorders in pregnancy; PROM, premature rupture of membranes; LGA, Large-for-gestational-age; SGA, small-for-gestational-age.

The gravidity, parity, assisted reproductive technology intervention, and twin or multiple gestations were adjusted.

Table S14 The prevalence of comorbid pregnancy complications

The number of pregnancy complications	Total population	<20	20-24	25-29	30-34	35-39	≥40
None	161563 (57.66）	4552 (64.7）	30191 (64.51）	73282 (61.09）	38620 (52.78）	12797 (46）	2118 (39.16）
One complication	89934 (32.1）	2025 (28.78）	13461 (28.76）	36476 (30.41）	25330 (34.62）	10501 (37.74）	2140 (39.56）
Two complications	24498 (8.74）	409 (5.81）	2782 (5.94）	8870 (7.39）	7773 (10.62）	3743 (13.45）	921 (17.03）
Three or more complications	4208 (1.5）	50 (0.71）	368 (0.79）	1336 (1.11）	1444 (1.97）	780 (2.8）	230 (4.25）

Table S15 The risk of comorbid pregnancy complications for adverse perinatal outcomes

Adverse perinatal outcome	Comorbid pregnancy complications						
	No	One	*P*	Two	*P*	Three or more	*P*
Polyhydramnios	Reference	1.11 (1.02–1.2)	0.012	1.06 (0.94–1.2)	0.335	1.07 (0.83–1.38)	0.603
Oligohydramnios	Reference	1.11 (1.06–1.16)	0.000	1.08 (1.01–1.15)	0.027	1.07 (0.92–1.24)	0.366
PROM	Reference	0.98 (0.96–1.01)	0.208	0.94 (0.91–0.99)	0.009	0.83 (0.75–0.91)	0.000
Placenta previa	Reference	1.42 (1.33–1.51)	0.000	1.44 (1.32–1.57)	0.000	1.26 (1.04–1.51)	0.016
Placental abruption	Reference	1.48 (1.37–1.6)	0.000	1.9 (1.71–2.11)	0.000	2.74 (2.26–3.32)	0.000
Cesarean section	Reference	1.3 (1.27–1.33)	0.000	1.46 (1.41–1.52)	0.000	1.67 (1.54–1.8)	0.000
Perinatal asphyxia	Reference	1.27 (1.2–1.33)	0.000	1.34 (1.25–1.45)	0.000	1.52 (1.31–1.78)	0.000
LGA	Reference	1.1 (1.05–1.15)	0.000	1.16 (1.08–1.24)	0.000	1.3 (1.13–1.5)	0.000
SGA	Reference	1.79 (1.68–1.91)	0.000	2.45 (2.25–2.66)	0.000	3.69 (3.19–4.27)	0.000
Postpartum hemorrhage	Reference	2.17 (2.08–2.26)	0.000	3.02 (2.87–3.19)	0.000	3.62 (3.28–4)	0.000
Premature	Reference	1.28 (1.24–1.33)	0.000	1.57 (1.49–1.65)	0.000	1.98 (1.79–2.19)	0.000
Stillbirth	Reference	1.18 (0.99–1.41)	0.065	1.26 (0.97–1.63)	0.077	1.39 (0.81–2.38)	0.236
Congenital malformation	Reference	1.29 (1.13–1.48)	0.000	1.44 (1.19–1.74)	0.000	1.84 (1.28–2.64)	0.001

The maternal age, gravidity, parity and ART treatment were adjusted. ART, assisted reproductive technology; PROM, premature rupture of membranes; LGA, Large-for-gestational-age; SGA, small-for-gestational-age.
